# Serum S100B Level in the Management of Pediatric Minor Head Trauma: A Randomized Clinical Trial

**DOI:** 10.1001/jamanetworkopen.2024.2366

**Published:** 2024-03-19

**Authors:** Damien Bouvier, Aymeric Cantais, Alban Laspougeas, Fleur Lorton, Yannick Plenier, Maria Cottier, Philippe Fournier, Antoine Tran, Emilie Moreau, Julie Durif, Catherine Sarret, Charline Mourgues, Franck Sturtz, Jean-Baptiste Oudart, Juliette Raffort, Philippe Gonzalo, Jean-Paul Cristol, Damien Masson, Bruno Pereira, Vincent Sapin

**Affiliations:** 1 Department of Biochemistry and Molecular Genetics, Centre Hospitalier Universitaire (CHU) Clermont-Ferrand, Université Clermont Auvergne, Centre National de la Recherche Scientiﬁque (CNRS), Institut National de la Santé et de la Recherche Médicale (INSERM), Génétique, Reproduction et Développement, Clermont-Ferrand, France.; 2 Department of Pediatrics, CHU Saint-Etienne, Saint-Etienne, France; 3 Department of Pediatrics, CHU Limoges, Limoges, France; 4 Pediatric Emergency Department, Nantes Université, CHU Nantes, INSERM, Centre d’Investigation Clinique 1413, Nantes, France; 5 Department of Pediatrics, CHU Reims, Reims, France; 6 Department of Pediatrics, CHU Montpellier, Montpellier, France; 7 Department of Pediatrics, CHU Nîmes, Nîmes, France; 8 Department of Pediatrics, CHU Nice, Nice, France; 9 Department of Pediatrics, Assistance Publique–Hôpitaux de Marseille, Marseille, France; 10 Department of Biochemistry and Molecular Genetics, CHU Clermont-Ferrand, Clermont-Ferrand, France; 11 Department of Pediatrics, CHU Clermont-Ferrand, Université Clermont Auvergne, CNRS, SIGMA, Thérapies Guidées par l’Image, Clermont-Ferrand, France; 12 Biostatistics Unit (Délégation à la Recherche Clinique et à l’Innovation), CHU Clermont-Ferrand, Clermont-Ferrand, France; 13 Department of Biochemistry, CHU Limoges, Limoges, France; 14 Faculté de Médecine, Université de Reims Champagne-Ardenne, Matrice Extracellulaire et Dynamique Cellulaire Unit, UMR CNRS 7369, Reims, France; 15 Department of Biochemistry, CHU Nice, Nice, France; 16 Department of Biochemistry and Pharmacology, CHU Saint-Etienne, Saint-Etienne, France; 17 Department of Biochemistry, CHU Montpellier, Montpellier, France; 18 Department of Biochemistry, CHU Nantes, Nantes, France

## Abstract

**Importance:**

Minor head trauma (HT) is one of the most common causes of hospitalization in children. A diagnostic test could prevent unnecessary hospitalizations and cranial computed tomographic (CCT) scans.

**Objective:**

To evaluate the effectiveness of serum S100B values in reducing exposure to CCT scans and in-hospital observation in children with minor HT.

**Design, Setting, and Participants:**

This multicenter, unblinded, prospective, interventional randomized clinical trial used a stepped-wedge cluster design to compare S100B biomonitoring and control groups at 11 centers in France. Participants included children and adolescents 16 years or younger (hereinafter referred to as children) admitted to the emergency department with minor HT. The enrollment period was November 1, 2016, to October 31, 2021, with a follow-up period of 1 month for each patient. Data were analyzed from March 7 to May 29, 2023, based on the modified intention-to-treat and per protocol populations.

**Interventions:**

Children in the control group had CCT scans or were hospitalized according to current recommendations. In the S100B biomonitoring group, blood sampling took place within 3 hours after minor HT, and management depended on serum S100B protein levels. If the S100B level was within the reference range according to age, the children were discharged from the emergency department. Otherwise, children were treated as in the control group.

**Main Outcomes and Measures:**

Proportion of CCT scans performed (absence or presence of CCT scan for each patient) in the 48 hours following minor HT.

**Results:**

A total of 2078 children were included: 926 in the control group and 1152 in the S100B biomonitoring group (1235 [59.4%] boys; median age, 3.2 [IQR, 1.0-8.5] years). Cranial CT scans were performed in 299 children (32.3%) in the control group and 112 (9.7%) in the S100B biomonitoring group. This difference of 23% (95% CI, 19%-26%) was not statistically significant (*P* = .44) due to an intraclass correlation coefficient of 0.32. A statistically significant 50% reduction in hospitalizations (95% CI, 47%-53%) was observed in the S100B biomonitoring group (479 [41.6%] vs 849 [91.7%]; *P* < .001).

**Conclusions and Relevance:**

In this randomized clinical trial of effectiveness of the serum S100B level in the management of pediatric minor HT, S100B biomonitoring yielded a reduction in the number of CCT scans and in-hospital observation when measured in accordance with the conditions defined by a clinical decision algorithm.

**Trial Registration:**

ClinicalTrials.gov Identifier: NCT02819778

## Key Points

**Question:**

What is the value of measuring serum S100B level in reducing cranial computed tomography (CCT) exposure in minor head trauma?

**Findings:**

In this randomized clinical trial that included 2078 children, CCT scans were performed in 299 of 926 (32%) in the control group, and 112 of 1152 (10%) in the S100B biomonitoring group. The between-group difference of 23% was not statistically significant.

**Meaning:**

When measured in accordance with the conditions defined by a clinical decision algorithm for the management of pediatric minor head trauma, serum S100B level yielded a reduction in the number of CCT scans, thereby avoiding unnecessary radiation exposure.

## Teaser

This randomized clinical trial evaluates the effectiveness of serum S100B measurement to reduce exposure to cranial computed tomographic scans among children admitted to the emergency department with minor head trauma.

## Introduction

Head trauma (HT) constitutes a leading public health problem in children (691 per 100 000 in emergency departments).^1,2^ Mild HT, defined by a Glasgow Coma Scale (GCS) score of 13 to 15,^3^ is one of the most common causes of pediatric hospital admissions.^4,5,6^

Cranial computed tomography (CCT) is a standard diagnostic tool for adults with HT. In children, however, several recent large-scale epidemiologic studies have highlighted an increased risk of cancer related to the radiation exposure from CCT scans.^7,8,9^ Alternatively, admission for observation with CCT scanning only in the case of clinical deterioration would reduce x-ray exposure but would be more costly.^10,11^ In addition, 93% to 100% of these children do not develop intracerebral lesions.^12^ In this context, clinical decision rules were designed to help clinicians identify children at very low risk of developing intracerebral lesions.^13,14^ The Paediatric Emergency Care Applied Research Network algorithm for children with minor HT (GCS score, 14-15) classify children in 3 risk categories (very low, intermediate, and high) for developing clinically important brain injury to help CCT decision-making. The algorithm leads to more cost-effective care^15^ and has been reported to yield a 10% reduction of CCT scans performed for all 3 risk categories.^13^ The intermediate risk category (GCS score, 15) concerns about 30% of children and accounts for 0.9% of clinically important HT. For this group, observation and/or CCT scans are recommended without further defining observation.^14^

A diagnostic test for HT, although involving blood sampling, would prevent unnecessary hospitalizations and CCT scans for many children. Serum S100B protein is well established as a sensitive acute HT biomarker. It has a short half-life of about 30 to 100 minutes.^16,17,18^ The potential of serum S100B measurement as a means of reducing unnecessary CCT scans in adults with minor HT has been well established in many observational and interventional studies.^19,20,21,22,23,24,25,26^ Further efforts should focus on standardizing the interpretation of serum S100B values in the pediatric population using specific reference ranges.^27^ Some studies and a meta-analysis have reported data from children with minor HT.^28,29,30,31,32,33,34^

The primary objective of our study was to evaluate the reduction in CCT scan recommendations after measurement of serum S100B levels in the management of pediatric minor HT with an intermediate risk of clinically important brain injury. The secondary objectives were to evaluate the benefits of S100B-guided management in terms of length of stay in the emergency department, hospitalizations, radiation exposure, the detection of complications, the absence of late adverse effects, and lower management costs.

## Methods

### Study Design

This investigator-initiated, unblinded, multicenter randomized clinical trial used a stepped-wedge cluster design in 10 French university hospital centers (Clermont-Ferrand, Limoges, Lyon, Marseille, Montpellier, Nice, Reims, Saint-Etienne, Nantes, and Nîmes) and in 1 French hospital center (Vichy). This study followed the Consolidated Standards of Reporting Trials (CONSORT) reporting guideline for stepped-wedge cluster randomized trials. The enrollment period was November 1, 2016, to October 31, 2021, with a follow-up period of 1 month for each patient. The protocol was approved by the Sud-Est 6 ethics committee and the French Agency for the Safety of Health Products. Patients and/or the public were not involved in the research question and design of the study. The study protocol was published previously.^35^ The protocol submitted to the ethics committee and the statistical analysis plan are available in Supplement 1.

 Centers were randomly assigned to a step by the study statistician (B.P.) using a block randomization sequence generated in Stata software, version 13 (StataCorp LLC). Step composition was stratified according to the planned recruitment of each participating center. Fifteen 4-month intervals were defined over 60 months. The randomization consisted of 5 steps, 2 centers enrolled in each of steps 1 to 4 and 3 centers enrolled in step 5 (eFigure 1 in Supplement 2). Children in the control group received conventional treatment in accordance with the French Paediatric Society (Société Française de Pédiatrie [SFP]) recommendations (eFigure 2 in Supplement 2). A single micromethod venous blood sample (1 mL) was collected in patients in the S100B biomonitoring group for S100B determination within 3 hours of trauma, and their subsequent management depended on the results of S100B assay. Children with a positive test result received conventional treatment in accordance with the SFP recommendations.^36^ In the case of a negative test result, the children were discharged from the emergency department after 6 hours of observation, as suggested by the Sud-Est 6 ethics committee (eFigure 3 in Supplement 2).

### Participants

Children and adolescents 16 years or younger (hereinafter referred to as children) presenting to the emergency department with minor HT and a GCS score of 15 were included in 2 groups: a control group, in which patients received usual care (conventional treatment), and a biomonitoring group, in which patients received treatment that included the S100B protein assay (Figure).

**Figure.**
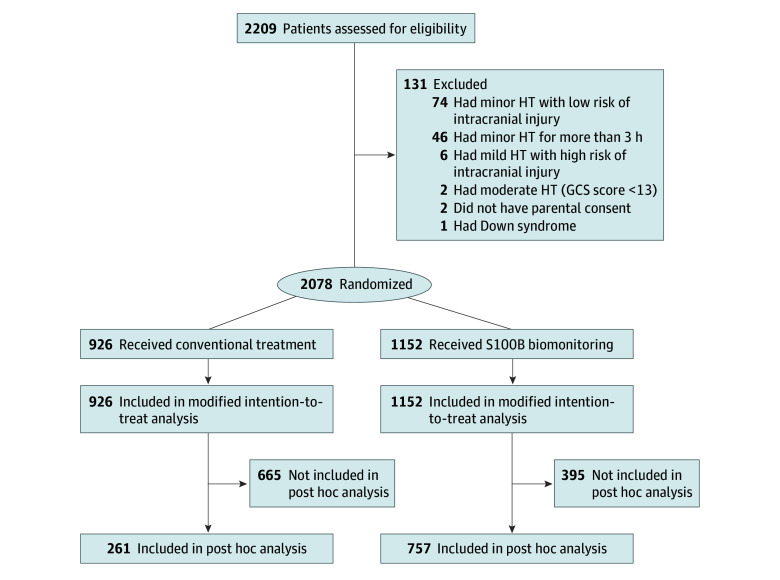
Study Flowchart GCS indicates Glasgow Coma Scale; HT, head trauma.

Children were enrolled in the study after obtaining signed parental consent. Research associates entered the data into the electronic case report form. The presence of persistent clinical signs 48 hours and 3 weeks after minor HT was monitored by telephone calls by the clinical research associates for both groups. Other hospitalizations were also sought in the medical records. The total duration of the study was 61 months (60 months of enrollment plus 1 month for follow-up of the last enrolled patients).

### Consent

Patients and their parents or legal guardians were fully informed, in understandable language, about the objectives and constraints of the study, the potential risks, the required monitoring and safety measures, and of their right to refuse to participate in the study and the possibility to revoke their consent at any time. The opinion of the Sud-Est 6 ethics committee was also communicated.

### Eligibility

The study population consisted of pediatric patients (aged ≤16 years) admitted to pediatric emergency departments for minor HT with a GCS score of 15 requiring hospitalization and/or a CCT scan as per SFP recommendations. The GCS was used to evaluate children 2 years and older while the pediatric GCS was used in nonverbal children younger than 2 years.^37^

Inclusion criteria were 16 years or younger, management within 3 hours after HT (for blood sampling), and a GCS score of 15 classically requiring hospitalization and/or a CCT scan as per SFP recommendations (eFigures 2 and 3 in Supplement 2).^36^ For children younger than 2 years, the criteria were parietal or occipital scalp hematoma, loss of consciousness for more than 5 seconds, trauma due to a severe accident (eFigures 2 and 3 in Supplement 2), and abnormal behavior according to the parents. For children 2 years and older, the criteria were loss of consciousness at the time of the accident, vomiting, trauma due to severe accident (eFigures 2 and 3 in Supplement 2), and severe headache. Exclusion criteria were enrollment in another therapeutic trial with drug administration, Down syndrome, melanoma, trauma occurring more than 3 hours prior to admission, GCS score of 13 or 14, signs of skull fracture or skull base lesions (CCT scan recommended), HT not requiring hospitalization and/or a CCT scan as per SFP recommendations, and refusal by the child, parents, or legal guardian (eFigures 2 and 3 in Supplement 2).^36^

### Outcome Measures

The primary end point was the proportion of CCT scans recommended (absence or presence of CCT scan for each patient) within 48 hours of HT, compared between the 2 groups. The secondary end points were duration of management defined by the time spent in the pediatric emergency department (time from admission to discharge), duration of hospitalization for observation, and cost of management. In addition, secondary end points also concerned the effective radiation dose for each CCT scan recorded on each patient’s x-ray report, the presence of intracranial injury on CCT scans identified by radiological examination, and the presence of persistent clinical signs at the telephone follow-up interviews (48 hours and 3 weeks after injury).

For discharged patients, the presence or persistence of clinical signs at 48 hours and 3 weeks after injury was evaluated through a standardized telephone interview conducted by a clinical research associate. The following items were collected and analyzed separately for all participating children: frequency of vomiting since returning home, problems or difficulties observed by parents related to arm or leg movements, convulsions, ocular discomfort, signs of facial paralysis, and parent’s opinion of child’s return to previsit health status or changes observed. Additional information on headache complaints was also collected for children 4 years or older.

In case of hospitalization, the follow-up was performed using medical records. Clinical signs sought were vomiting, facial paralysis, movement disorders, vertigo, pupillary light reflex disorder, seizure, progressive headache, or behavior change. Hospitalizations in the neurosurgery or the intensive care unit were also noted.

### S100B Assay

The analytical method used was based on an electrochemiluminescence assay (Roche Diagnostics instruments) in the central laboratory of each center. Results were available within 1 hour. The S100B serum assay finding was considered positive (for intracranial lesion) according to age as follows: 0 to 9 months, greater than 0.35 μg/L; 10 to 24 months, greater than 0.23 μg/L; and older than 24 months, greater than 0.18 μg/L.^38^

### Sample Size Estimation

The proportion of CCT scans ordered in conventional minor HT treatment is approximately 20% (range, 10%-30%). Thus, to detect an absolute difference of 6% between the 2 groups (ie, a relative reduction of 30% in the rate of CCT scans ordered in the S100B biomonitoring group compared with the control group), a sample of 615 participants per group is required using an individually randomized design for a 2-sided type I error at 0.05 at a statistical power of 80%. Furthermore, in light of findings by Calcagnile et al,^23^ we expected that 30% of physicians treating patients in the S100B biomonitoring group would not base their practice on the result of the serum S100B assay. Thus, 800 participants per group were needed to conserve a power of 80% with the hypotheses defined previously.^39^ Due to the stepped-wedge cluster design, the sample size was inflated to account for between- and within-center variability measured by an intraclass correlation coefficient (ICC). According to randomization sequence, time, mean number of patients per center, coefficient of variation of cluster size defined as the ratio of the SD of cluster sizes, ICC expected between 0.005 and 0.05, and loss to follow-up (around 5%), 4000 patients (2000 patients per group) were required.^40,41^ The Stata routine stepped-wedge program was used to estimate sample size.

After the enrollment of more than 2000 patients, a prespecified interim analysis was conducted. According to the rate of inclusions, expected and observed ICC values, observed absolute difference between randomized groups for the primary end point, and the inflated type I error, an independent committee recommended to stop the inclusions.

### Statistical Analysis

Data were analyzed from March 7 to May 29, 2023. The planned approach to statistical analysis was published previously.^35^ We analyzed data in the modified intention-to-treat population, which was designed to exclude patients whose inclusion was associated with 1 or more serious protocol violations. As recommended by the independent committee, we also performed an exploratory post hoc analysis for the primary end point in a population that was enrolled only from well-functioning centers (ie, when the S100B intervention was correctly implemented) defined in a modified statistical plan. The centers that did not comply with the decision algorithm for CCT scans and hospitalizations for over 20% of patients and/or included at least 60% fewer patients in the S100B biomonitoring group than in the control group were excluded from the post hoc analysis.

The primary outcome was compared between the 2 groups using generalized linear mixed models (robust Poisson distribution). Randomization groups, randomization steps, time, and their interactions were evaluated as fixed effects and center and time as random effects. Results were expressed as relative risks (RRs) and 95% CIs. The intracluster correlation and time effect from the fitted model were estimated. Adjusted analyses were performed using the model defined for the primary analysis with prespecified covariates determined according to clinically relevant parameters: age group distribution and causes of minor HT. Results are reported as absolute differences and adjusted RRs (ARRs) with 95% CIs.

Other binary outcomes were tested using the same statistical model to account for intercenter and intracenter variability. Results are reported as RRs with 95% CIs. For continuous end points, between-group comparisons were performed using a linear mixed model with the aforementioned fixed and random effects. Results are presented as between-group differences with 95% CIs for continuous data.

All analyses were performed using Stata software, version 15 (StataCorp LLC). Statistical adjustment for the interim analysis was required as analyses were conducted for half of the inclusions. Using O’Brien-Fleming estimates, the inflated type I error was 0.005. Thus, for the primary end point, a 2-sided *P* < .005 was considered statistically significant; for the secondary end points, a 2-sided *P* < .05 was considered statistically significant. No correction for multiple testing was applied to the secondary outcomes or subgroup analyses. There were no missing data for the primary end point. For secondary outcomes, the number of available data was reported. Statistical analyses were performed without an imputation data method.

### Economic Analysis

An economic analysis was drafted from the hospital point of view. The analysis compared the costs of hospitalization stay in the 2 groups (see statistical analysis plan in Supplement 1).

## Results

### Modified Intention-to-Treat Analysis

#### Cohort Description

A total of 2078 children (median age, 3.2 [IQR, 1.0-8.5] years) were included over a period of 60 months: 926 in the conventional treatment group and 1152 in the S100B biomonitoring group (Figure). The characteristics of the 2 groups are shown in Table 1. Participants included 1235 boys (59.4%) and 843 girls (40.6%).

**Table 1.** Characteristics of the Conventional Treatment Group and S100B Biomonitoring Group

Characteristic	Patient group^a^		*P* value
	Conventional treatment (n = 926)	S100B biomonitoring (n = 1152)	
Age, median (IQR), y	2.7 (0.9-7.7)	3.4 (1.1-9.2)	.02
Age group distribution, mo			
0-9	184/926 (19.9)	174/1152 (15.1)	.02
10-24	214/926 (23.1)	276/1152 (24.0)	
>24	528/926 (57.0)	702/1152 (60.9)	
Sex			
Boys	533/926 (57.6)	702/1152 (60.9)	.14
Girls	393/926 (42.4)	450/1152 (39.1)	
Weight, median (IQR), kg	14.0 (9.2-25.9)	15.0 (10.0-30.0)	.007
Height, median (IQR), cm	93 (74-128)	97 (74-137)	.04
Distance between home and hospital, median (IQR), km	13 (6-25)	12 (5-25)	.57
Direct mechanism of injury	910/926 (98.3)	1144/1152 (99.3)	.01
Causes of minor HT			
Domestic accident	648/926 (70.0)	753/1152 (65.4)	<.001
School accident	154/926 (16.6)	139/1152 (12.1)	
Sports related	74/926 (8.0)	163/1152 (14.1)	
Road accident	44/926 (4.8)	94/1152 (8.2)	
Intoxication	6/926 (0.6)	3/1152 (0.3)	
Inclusion criteria for children aged <2 y			
Parietal or occipital scalp hematoma	79/390 (20.3)	63/435 (14.5)	.10
Loss of consciousness >5s	42/391 (10.7)	46/435 (10.6)	.77
Trauma due to serious accident	278/391 (71.1)	333/435 (76.6)	.59
Abnormal behavior in parents’ opinion	74/390 (19.0)	66/435 (15.2)	.26
Inclusion criteria for children aged ≥2 y			
Loss of consciousness	196/534 (36.7)	260/710 (36.6)	.94
Vomiting	203/534 (38.0)	240/709 (33.9)	.15
Trauma due to serious accident	238/534 (44.6)	317/710 (44.7)	.42
Severe headache	136/534 (25.5)	156/710 (22.0)	.78

Abbreviation: HT, head trauma.

^a^ Unless otherwise indicated, data are expressed as No./total No. (%) of patients.

#### Primary End Point

A CCT scan was performed for 299 children (32.3%) in the control group (n = 926), and 112 (9.7%) in the S100B biomonitoring group (n = 1152). This difference of 23% (95% CI, 19%-26%) was not statistically significant (ARR, 0.87 [95% CI, 0.61-1.24]; *P* = .44) with an ICC of 0.32 (Table 2).

**Table 2.** Proportion of CCT Scans Recommended Within 48 Hours Following Minor HT Compared Between the Conventional Treatment Group and S100B Biomonitoring Group

Analysis type	No. of centers	Patient group, No. with CCT/total No. (%)		RR (95% CI)	*P* value	ARR (95% CI)^a^	*P* value	ICC
		Conventional treatment	S100B biomonitoring					
Modified intention-to-treat analysis	11	299/926 (32.3)	112/1152 (9.7)	0.95 (0.65-1.39)	.79	0.87 (0.61-1.24)	.44	0.32
Post hoc analysis	4	20/261 (7.7)	31/757 (4.1)	0.52 (0.28-0.96)	.04	0.49 (0.30-0.77)	.002	0.02

Abbreviations: ARR, adjusted relative risk (RR); CCT, cranial computed tomography; HT, head trauma; ICC, intraclass correlation coefficient.

^a^ Adjusted for age group distribution and minor HT causes.

#### Secondary End Points

Comparisons of secondary end points between the S100B biomonitoring group and the control group are presented in Table 3. Statistically significant reductions of hospitalizations (479 [41.6%] vs 849 [91.7%]; RR, 0.46 [95% CI, 0.39-0.51]; *P* < .001; absolute difference, 50% [95% CI, 47%-53%]), hospitalizations longer than 48 hours (50 [4.3%] vs 55 [5.9%]; RR,  0.33 [95% CI, 0.15-0.75]; *P* = .005), parental leave (49 of 935 [5.2%] vs 66 of 805 [8.2%]; RR, 0.50 [95% CI, 0.35-0.70]; *P* < .001), and median hospitalization costs (€181 [IQR, €181-€498] vs €498 [IQR, €498-€498]; *P* < .001; between-group difference, −€213 [95% CI, −€344 to −€84]) were observed in the S100B biomonitoring group.

**Table 3.** Secondary End Points Compared Between the Conventional Treatment Group and S100B Biomonitoring Group

Secondary end points	Patient group^a^		RR (95% CI)	*P* value
	Conventional management control group (n = 926)	S100B biomonitoring group (n = 1152)		
Hospitalization				
Hospital monitoring	849/926 (91.7)	479/1152 (41.6)	0.46 (0.39 to 0.51)	<.001
Hospitalization >48 h	55/926 (5.9)	50/1152 (4.3)	0.33 (0.15 to 0.75)	.005
Hospitalization in ICU	1/926 (0.1)	4/1152 (0.3)	3.12 (0.41 to 23.60)	.27
Hospitalization in neurosurgery department	10/926 (1.1)	6/1152 (0.5)	0.53 (0.11 to 2.51)	.43
CCT scan				
Effective radiation dose, median (IQR), mSv	400/926 (285-570)	526 (367-768)	121 (50 to 192)^b^	.01
Presence of intracranial injury on first CCT scan	66/299 (22.1)	33/112 (29.5)	1.35 (0.87 to 2.09)	.15
Presence of intracranial injury on every CCT scan	82/324 (25.3)	55/140 (39.3)	1.53 (0.97 to 2.41)	.07
Bone fracture	66/82 (80.5)	43/55 (78.2)	0.97 (0.78 to 1.20)	.79
Epidural hematoma	13/82 (15.9)	15/55 (27.3)	1.42 (0.01 to 184.40)	.89
Hemorrhagic contusion	11/82 (13.4)	2/55 (3.6)	0.27 (0.03 to 2.65)	.26
Subdural hematoma	6/82 (7.3)	7/55 (12.7)	1.74 (0.61 to 4.94)	.30
Pneumocephalus	11/82 (13.4)	11/55 (20.0)	1.49 (0.70 to 3.18)	.30
Subarachnoid hemorrhage	14/82 (17.1)	7/55 (12.7)	0.75 (0.32 to 1.71)	.49
Othematoma	9/82 (11.0)	3/55 (5.5)	0.50 (0.21 to 1.20)	.12
Persistent clinical signs at the telephone follow-up interview 48 h after the minor HT				
Presence of persistent clinical signs	195/795 (24.5)	235/977 (24.1)	0.98 (0.69 to 1.40)	.91
Vomiting, No. (%)	35/195 (17.9)	39/235 (16.6)	0.92 (0.68 to 1.25)	.61
Headache in children aged ≥2 y	100/195 (51.3)	120/235 (51.1)	1.00 (0.86 to 1.16)	.96
Motor deficit	0	0	NE	NE
Convulsion	1/195 (0.5)	0	NE	NE
Facial paralysis	0	1/235 (0.4)	NE	NE
Abnormal pupillary light reflex	46/195 (23.6)	47/235 (20.0)	0.85 (0.50 to 1.44)	.54
Abnormal behavior	85/195 (43.6)	128/235 (54.5)	1.10 (0.52 to 2.31)	.81
Persistent clinical signs at the telephone follow-up interview 3 wk after minor HT				
Presence of persistent clinical signs	90/808 (11.1)	87/945 (9.2)	0.73 (0.59 to 0.91)	.006
Vomiting	10/90 (11.1)	13/87 (14.9)	1.05 (0.22 to 5.02)	.95
Headache in children aged ≥2 y	47/90 (52.2)	36/87 (41.4)	0.79 (0.53 to 1.18)	.25
Motor deficit	0	0	NE	NE
Convulsions	1/90 (1.1)	1/87 (1.1)	1.03 (0.15 to 7.21)	.97
Facial paralysis	2/90 (2.2)	4/87 (4.6)	2.07 (0.63 to 6.76)	.23
Abnormal pupillary light reflex	8/90 (8.9)	11/87 (12.6)	1.42 (0.72 to 2.79)	.31
Abnormal behavior	44/90 (48.9)	54/87 (62.1)	0.78 (0.45 to 1.36)	.37
Hospitalized within 3 wk	3/808 (0.4)	5/945 (0.5)	1.36 (0.31 to 6.04)	.69
Cost of management				
Cost of hospitalization, median, median (IQR), €	498 (498-498)	181 (181-498)	−213 (−344 to −84)^b^	<.001
Parental leave	66/805 (8.2)	49/935 (5.2)	0.50 (0.35 to 0.70)	<.001

Abbreviations: CCT, cranial computed tomography; HT, head trauma; ICU, intensive care unit; NE, not evaluated; RR, relative risk.

^a^ Unless otherwise indicated, data are expressed as No./total No. (%) of patients.

^b^ Between-group differences instead of RR.

### Post Hoc Analysis

An exploratory post hoc analysis was performed excluding 7 centers (eTable 1 in Supplement 2). In the 4 centers included in the post hoc analysis, 1018 children were included (261 in the control group and 757 in the S100B biomonitoring group) (Figure and eTable 2 in Supplement 2). The post hoc analysis highlighted a significant reduction in CCT scan recommendations in the S100B biomonitoring group when compared with the control group (ARR, 0.49 [95% CI, 0.30-0.77]; *P* = .002) with an ICC of 0.02. The proportions were 31 of 757 (4.1%) and 20 of 261 (7.7%), respectively (Table 2).

## Discussion

We conducted a study in the largest cohort of children to date, to our knowledge, to evaluate serum S100B biomonitoring in the management of minor HT. A significant marginal difference was observed in the age comparison between the control group and the S100B biomonitoring group, which logically implied that children in the control group were smaller in height and weight, had more domestic accidents, and received a lower effective radiation dose.^42^ This may reflect a reluctance to collect blood samples from younger children. Therefore, the age distribution and causes of mild HT were used as adjustment variables in the primary end point analysis. In the control group, 32.3% of children had a CCT scan within 48 hours of minor HT, compared with 9.7% in the S100B biomonitoring group. This reduction of approximately two-thirds between the 2 groups was not significant due to the center effect. This effect is partly explained by the high intercenter heterogeneity in the proportion of CCT scans in the control groups (between 5% and 71%). This effect was exacerbated by the fact that some centers stopped enrolling at the time of the changeover. A better appropriation of the biomarker by physicians would increase adherence to the protocol and more accurately assess the effectiveness of S100B.

An exploratory post hoc analysis was conducted at 4 observant centers over the entire inclusion period and in compliance with the decision algorithm. This analysis showed that 7.7% of children in the control group had a CCT scan within 48 hours of minor HT compared with 4.1% in the S100B biomonitoring group. This reduction between the 2 groups was statistically significant, with no significant center effect. In the control group, 91.7% of children were hospitalized for monitoring compared with 41.6% in the S100B biomonitoring group. This notion of hospital monitoring, present in the French recommendations, includes observation during a short-term hospitalization (<24 hours) to conventional hospitalization and is not directly transposable to that mentioned in the North American recommendations, which is shorter (4-6 hours).

A meta-analysis highlighted the lack of a large multicenter study in the pediatric population.^33^ We also used age-adjusted reference ranges. In a before-and-after study conducted in 1062 children at intermediate risk of clinically important traumatic brain injury,^34^ the implementation of a modified Paediatric Emergency Care Applied Research Network rule including S100B assay significantly reduced the proportion of CCT scans and in-hospital observations for children with minor HT by 34% (95% CI, 10.3%-54.6%) and 45.2% (95% CI, 39.9%-51.3%), respectively. In our study, we found an RR of 0.49 (95% CI, 0.30-0.77) in the post hoc analysis for CCT scans and 0.46 (95% CI, 0.39-0.51) in the modified intention-to-treat analysis for in-hospital observations.

### Limitations

The limitations of our study are related to the difficulty of some centers to enroll in the 2 groups, which is consistent with the known challenges of recruiting pediatric populations in emergency settings.^43^ A prespecified interim analysis after enrollment of 2000 patients to estimate the statistical power based on the ICC and the observed absolute difference identified the impact of the center effect on the primary end point and led the independent committee (1) to decide to stop the trial and (2) to perform a post hoc exploratory analysis.

## Conclusions

In this randomized clinical trial of effectiveness of the serum S100B in the management of pediatric minor HT, we observed that S100B yielded a reduction in the number of CCT scans and in-hospital observations when measured in accordance with the conditions defined by a clinical decision algorithm. Learned societies in pediatrics and emergency medicine could issue recommendations along these lines.

## Notes

Supplement 1.
Trial Protocol

Supplement 2. **eFigure 1.** Stepped-Wedge Study Design **eFigure 2.** Decision Algorithm for CCT Scan or Hospitalization Indication for Children With Minor Head Trauma Management in the Conventional Treatment Group **eFigure 3.** Decision Algorithm for CCT Scan or Hospitalization Indication for Children With Minor Head Trauma Management in the S100B Biomonitoring Group **eTable 1.** Rationale for Exploratory Post Hoc Analysis **eTable 2.** Characteristics of the Conventional Treatment Group and S100B Biomonitoring Group: Exploratory Post Hoc Analysis on 4 Centers

Supplement 3.
Data Sharing Statement
