# Supplementary material for: Serum S100B Level in the Management of Pediatric Minor Head Trauma: A Randomized Clinical Trial
Source: JAMA Netw Open. 2024 Mar 19;7(3):e242366. doi: 10.1001/jamanetworkopen.2024.2366 (PMC10951739; doi:10.1001/jamanetworkopen.2024.2366)
Supplement: Supplement 1. — Trial Protocol [file jamanetwopen-e242366-s001.pdf]

This is a supplement to the manuscript entitled “Effectiveness of the serum S100B in the management of paediatric mild traumatic brain injury: a randomized clinical trial”

Study registration: ClinicalTrials.gov Identifier NCT02819778

This supplement contains the following items:

1. Summary of changes to the study protocol 2

2. Original protocol (version 2, accepted by the ethics committee on Jun 08, 2016) 3

3. Final version of the study protocol (version 8, accepted on Nov 11, 2019) 74

4. Study protocol (published in BMJ Open on May 24, 2019) 146

5. Statistical analysis plan 155

Protocols: Original (V2, May 02, 2016 and Final (V8, Oct 10, 2019)

Summary of changes to the Study Protocol

• Version V2 to V3 (Jul 05, 2016): Change Center following withdrawal. Added Philippe Fournier (Nimes University Hospital) to the Investigator list.

• Version V3 to V4 (Nov 07, 2016): Removed Vincent Guigonis (Limoges Hospital) to the Investigator list (retired) and added Aymeric Dallocchio (Limoges Hospital) to the Investigator list. Added Sarret Catherine, Dore Eric, Terral Daniel, Isfan Florentina, Merlin Etienne, Julian Valérie (Clermont-Ferrand University Hospital) ; Longis Bernard, Laguille Christine, Messager Véronique, Proust Sarah, Tahir Abdelilah (Limoges Hospital) ; Moreau Emilie, Boutin Aurélie, Caujolle Anaïs, Pailhous Sophie (Marseille Nord Hospital – AP-HM) ; Cottier Maria, Haquet Armelle, Blanc Brigitte, Maestracci Michel, Fournols Laura, Thibault Marielle, Ladet Séverine, Bidet Antoine (Montpellier University Hospital) ; Blanc Sibylle, Brun Jennifer, Butori Mathilde, Cabane Florence, Desaldeleer Cécile, Giannantonio Marie, Giurin Ida, Maillotte Anne-Marie, Montaudie-Dumas Isabelle, Kohsok Claire, Four Robert (Nice University Hospital) ; Filleron Anne, Gilton-Bott Lucie, Tran Tu-Anh (Nimes Hospital) ; Elmerich Florence, Medhioub Yasmine, Pons Aurélie, Minette Delphine, Thomas Emmanuelle, Bessaci Katia (Reims University Hospital) ; Desbree Aurélie, Cantais Aymeric, Chareyras Cécile, Colmant Richard Odile, Destombe Sylvie, Dupre Anne, Gay Claire, Latour Claire, Maluka Elona, Masse Magali, Molly Claudine, Mteirek Ahmad, Philbois Olivier, Rieu Valérie, Rigaudiere Philippe, Rolland Emmanuelle, Sevrez Chloé, Thuiller Charlotte, Tripodi Louise (Saint-Etienne University Hospital) to the Investigator list.

• Version V4 to V5 (Nov 03, 2017): Added Dall'Acqua David (Vichy Hospital); Champigny Marie-Alexandrine (Limoges Hospital); Salet Randa, Baron-Joly Sandrine, Catteau Natacha, Van Der Hende Kathleen, Walenda Carsten (Nimes Hospital) to the Investigator list. Update of the Study completion date from August 2018 to August 2019.

• Version V5 to V6 (Oct 12, 2018): Added Gras-Le Guen Christèle (Nantes University Hospital); Devos Caroline, Babe Philippe, Herisse Anne Laure, Demonchy Diane, Gignoux Laure, De Bieville Hubert, Berthet Stéphanie, Tran Antoine, Martin D'escrienne Marguerite (Nice University Hospital); Masson Alexandra (Limoges Hospital); Bouillon Jean Baptiste, Geneste Bruno, Boutry Morgane (Vichy Hospital) to the Investigator list. Update of the Study completion date from August 2019 to April 2020. Planned interim analysis after enrolment of 2000 patients. Change of Pediatrician on the independent monitoring committee, removed Gras-Le Guen Christèle and added Véronique Chasle (Rennes University Hospital)

• Version V6 to V7 (Dec 04, 2018): Added a biobank for panel of brain markers study.

• Version V7 to V8 (Oct 10, 2019): Removed Guyon Gael (Montpellier University Hospital) from the Investigator list (retired). Added Chloe Jullian, Tourniaire Gaelle and Jeziorski Eric (Montpellier University Hospital); Tronche Julie and Laspougeas Alban (Limoges Hospital); Meurice Laura, Cracco Ophélie, Gastaldi Margaux, Boucheron Adeline, Dumortier Morgane, Levieux Karine, Hubert-Dibon Gaëlle, Andre Sindy, Lorton Fleur, Ferraro Guillaume, Legallais Abel Elodie, Vrignaud Bénédicte (Nantes University Hospital) to the Investigator list. Study completion date from Avril 2020 to November 2021.

Interventional study assessing evaluation of the interest of serum S100B protein determination in the management of pediatric mild traumatic brain injury

Running title: PROS100B

Version: 2 **dated: 02/05/2016**

Sponsor Code	ANSM registration number
PHRC N 2015 BOUVIER	2016-A00195-46

Sponsor: **Clermont-Ferrand Teaching Hospital**
58 Rue de Montalembert
63003 Clermont-Ferrand Cedex 1
France

Coordinating investigator: ***Dr. Damien Bouvier (Physician-Biologist, Hosp.Practitioner assistant)***
Medical Biochemistry and Molecular Biology Clermont-Ferrand Teaching Hospital
58, Rue Montalembert 63003 Clermont-Ferrand cedex 1 France

Co-investigator: ***Prof. André Labbé (Pediatrician, Univ. Prof./Hosp.Practitioner, department head)***
Pediatrics Unit Hôpital Estaing Clermont-Ferrand Teaching Hospital
Place Lucie et Raymond Aubrac 63003 Clermont-Ferrand cedex 1 France

Scientific collaborator: ***Prof. Vincent Sapin (Biologist, Univ. Prof./Hosp.Practitioner, department head)***
Medical Biochemistry and Molecular Biology Clermont-Ferrand Teaching Hospital
58, Rue Montalembert 63003 Clermont-Ferrand cedex 1 France

Methodologist: ***Bruno Pereira (PhD)***
Clinical Research and Innovation Delegation Clermont-Ferrand Teaching Hospital
Villa annexe IFSI 58, rue Montalembert 63003 Clermont-Ferrand cedex 1 France

Economist: ***Charline Mourgues (MSc)***
Clinical Research and Innovation Delegation Clermont-Ferrand Teaching Hospital
Villa annexe IFSI 58, rue Montalembert 63003 Clermont-Ferrand cedex 1 France

ABSTRACT

Background: Mild traumatic brain injury (mTBI) (Glasgow Coma Scale score between 13 and 15) is a very common reason for presentation to pediatric emergency departments. So as not to overlook the risk of complications, which occur at a rate of 0-7%, measures such as cranial computed tomography (CCT-scan) and/or short inpatient observation are prescribed. Ultimately, the majority of these measures could be avoided (Homer and Kleinman, 1999) and a large Australian cohort shows that the risk of brain tumors is 2.44 times higher for children who had a CCT-scan (3.24 for age 1-4 years) (Mathews *et al.,* 2013). Assay of a sensitive biomarker in blood, such as the S100B protein, has the potential to reduce the number of these unnecessary measures (Bouvier *et al.,* 2012). Indeed, in this context, we have shown that this assay has a sensitivity of 100% (positive test associated with brain lesions in CCT-scan and/or the appearance of clinical complications) and a negative predictive value of 100% (negative test associated with the lack of brain damage to the CCT-scan and/or the occurrence of clinical complications). Thus, the benefit to the patient would be a support simplified compared to conventional care.

Primary objective*:* Evaluate the utility of serum S100B measurement in the management of pediatric mTBI by demonstrating a decrease in the proportion of CCT-scan prescribed in the "S100B management" intervention arm compared with the "conventional management" control arm, hypothesizing a 30% decrease in the number of CCT-scan between the intervention *versus* control arms.

Secondary objectives*:* Demonstrate the utility of serum S100B measurement with respect to:

- reduction in the time spent in the pediatric emergency room
- reduction in the duration of hospitalization
- reduction in radiation exposure (mSv)
- reduction in sedation and use of sedatives
- detection of complications (intracranial lesions) by CCT-scan which can occur at a rate of 0-7% in patients with mTBI (American Academy of Pediatrics, 1999)
- absence of intercurrent events at 48 hours and 3 weeks after mTBI
- compliance of emergency physicians with the S100B assay
- reduction of the cost of management

Type of study: The proposed protocol is a randomized, multicenter, open, prospective, interventional study (9 centers) using a stepped wedge cluster design, with two arms:

- Intervention group "S100B management"
- Control group "Conventional management"

This stepped wedge cluster randomization (stratified by cluster size) was chosen 1) to improve feasibility in emergency department and 2) to avoid the major risk of contamination bias in the control group.

Number of centers: 9

Clermont-Ferrand, Limoges, Lyon, Marseille, Montpellier, Nice, Reims, Saint-Etienne, Toulouse (Teaching Hospital - France)

Description of the study: Children in the “conventional management” control arm will have CCT-scan or be hospitalized according to the current recommendations of the French Society of Pediatry (SFP). In the “S100B management” intervention arm, blood sampling to determine serum S100B protein levels will take place within 3 hours after the mTBI and subsequent management will depend on the assay result (which is available 1 hour after arrival in the laboratory, dosing with the same technique of immunoassay in the 9 centers). If S100B is in normal range, the children will be discharged from the emergency department after 6 hours of observation. If the result is abnormal, CCT-scan or hospitalization will be prescribed in accordance with current SFP recommendations.

Primary outcome measure: The primary outcome measure will be the proportion of CCT-scans performed (absence/presence of CCT-scan for each patient) in the 48 hours following the mTBI, compared between the two arms (“S100B management” intervention *versus* “conventional management” control group).

Sample size: 4000 (2000 in each arm)

Inclusion criteria:

- Age ≤ 16 years
- Therapeutic management within 3 hours after the mTBI
- Glasgow Coma Scale score of 15 classically managed by hospitalization and/or CCT-scan as per SFP recommendations.

Non-inclusion criteria:

- Patient already enrolled in another therapeutic trial with drug administration
- Down syndrome
- Melanoma
- Refusal of child
- Refusal of parents or legal guardian
- Child with Glasgow Coma Scale ≤ 14
- Child with head injury and Glasgow Coma Scale score of 15 not requiring hospitalization and/or CCT-scan as per SFP recommendations.

Brief description of the products: This research protocol corresponds to a diagnostic study and no products will be administered to patients. Determination of serum S100B concentrations will be carried out by the medical biology laboratory at each center using the same automated immunoassay method (CE mark).

Conduct of the study: The study will run over a period of 24 months at 9 participating centers.

Summary of the benefits and foreseeable and known risks for subjects participating in the research: The risks to the patient are minimal since the probability of a false negative result is virtually nul. Furthermore, the serum assay of S100B protein is minimally invasive because it requires only a single blood sampling using a micro-method.

CONTENTS

ABBREVIATIONS **8**

1. GENERAL INFORMATION **9**

1.1. TITLE OF THE RESEARCH 9
1.2. SPONSOR CODE 9
1.3. SPONSOR 9
1.4. COORDINATION AND MONITORING 9
1.5. INVESTIGATORS 9
1.5.1. Coordinating investigator and collaborator *9*
1.5.2. Scientific collaborators and Co-investigators *10*
1.6. ASSOCIATE PARTNERS 12
1.7. STUDY SITES 12
1.8. DATA PROCESSING 13
1.9. ETHICS COMMITTEE 13
1.10. ESTIMATED TIME FRAME 13

2. STUDY RATIONALE / SCIENTIFIC JUSTIFICATION **14**

2.1. CURRENT STATE OF SCIENTIFIC KNOWLEDGE 14
2.2. HYPOTHESES AND OBJECTIVES 16
2.3. SUMMARY OF THE BENEFITS AND FORESEEABLE AND KNOWN RISKS FOR SUBJECTS PARTICIPATING IN THE RESEARCH 16
2.4. EXPECTED IMPACT 17
2.5. REFERENCES TO THE SCIENTIFIC LITERATURE AND TO PERTINENT DATA SERVING AS A BASIS FOR THE RESEARCH 17

3. STUDY OBJECTIVES **18**

3.1. PRIMARY OBJECTIVE 18
3.2. SECONDARY OBJECTIVES 18

4. DESCRIPTION OF THE STUDY **18**

4.1. TYPE OF STUDY 18
4.2. RESEARCH CATEGORY 19

5. STUDY POPULATION **19**

5.1. INCLUSION CRITERIA 20
5.2. NON-INCLUSION CRITERIA 20
5.3. PROCEDURE FOR PREMATURE TREATMENT DISCONTINUATION 21
5.4. EXCLUSION PERIOD AND PARTICIPATION IN ANOTHER STUDY 21
5.5. COMPENSATION OF SUBJECTS 21
5.6. RECRUITMENT MODALITIES 21

6. STUDY METHODOLOGY **22**

6.1. MEDICAL EVALUATION CRITERIA 22
6.1.1. Primary endpoint *22*
6.1.2. Secondary endpoints *22*
6.2. DESCRIPTION OF STUDY METHODOLOGY 22
6.3. DESCRIPTION OF THE MEASURES TAKEN TO REDUCE AND AVOID BIAS 25
6.3.1. Justification of randomization arms *25*
6.3.2. Blinding bias *25*

6.3.3. Randomization *26*
6.3.4. Reproducibility of the assay *26*
6.3.5. Control of attrition bias *26*

7. PRACTICAL CONDUCT OF THE STUDY 27

7.1. DETAILED DESCRIPTION OF PROCEDURES (DESCRIPTION OF EACH VISIT) 27
7.2. DESCRIPTION OF THE GENERAL LOGISTICAL ORGANIZATION OF THE STUDY 27
7.3. SAMPLES AND BIOLOGICAL ANALYSES 28
7.4. PLANNED DURATION OF PARTICIPATION AND STUDY TIME FRAME 28

8. STUDY PRODUCT 29

8.1. DESCRIPTION OF STUDY PRODUCT 29
8.2. PRESENTATION OF THE PRODUCTS 29
8.3. USE OF THE PRODUCT 29
8.4. AUTHORIZED AND UNAUTHORIZED MEDICINES AND TREATMENTS DURING THE STUDY 29

9. DATA COLLECTED 29

10. STATISTICAL CONSIDERATIONS 30

10.1. SAMPLE SIZE ESTIMATION 31
10.2. DATA ANALYSIS: GENERAL POINTS 32
10.3. CHARACTERISTICS OF PATIENTS AT BASELINE 32
10.4. PRIMARY ANALYSIS 32
10.5. SECONDARY ANALYSES 32
10.6. ECONOMIC ANALYSES 32
10.7. METHOD FOR MANAGING MISSING, UNUSED OR INVALID DATA 33
10.8. PERSONS IN CHARGE OF ANALYSIS 33

11. SAFETY ASSESSMENT – MANAGEMENT OF ADVERSE EVENTS 34

11.1. DEFINITIONS 34
11.2. SERIOUS ADVERSE EVENT REPORTING 35
11.3. MONITORING COMMITTEE 36
11.4. TERMINATION OF THE STUDY 37
11.5. FOLLOW-UP OF PATIENTS PRESENTING AN ADVERSE EVENT 37

12. RIGHT OF ACCESS TO SOURCE DOCUMENTS AND DATA 37

12.1. ACCESS TO DATA 37
12.2. SOURCE DATA 37
12.3. DATA CONFIDENTIALITY 37
12.4. REGISTRATION IN THE NATIONAL FILE OF BIOMEDICAL RESEARCH SUBJECTS 38

13. QUALITY CONTROL AND ASSURANCE 38

13.1. ENGAGEMENT OF THE INVESTIGATORS AND THE SPONSOR 38
13.2. QUALITY ASSURANCE 38
13.3. QUALITY CONTROL 38
13.4. CASE REPORT FORM 39

14. ETHICAL CONSIDERATIONS 39

14.1. ETHICS COMMITTEE 39
14.2. INFORMATION FOR PATIENTS AND WRITTEN INFORMED CONSENT FORM 39
14.3. PROTOCOL AMENDMENTS 40
14.4. MANAGEMENT RELATING TO THE RESEARCH 40

15. DATA PROCESSING AND STORAGE OF STUDY DOCUMENTS AND DATA 40

15.1. DATA ENTRY AND PROCESSING 40
15.2. CNIL 40
15.3. RECORD-KEEPING 40

16. FUNDING AND INSURANCE 41

16.1. STUDY BUDGET 41
16.2. INSURANCE 41

17. COMMUNICATION – RULES FOR PUBLICATION 41

18. FEASIBILITY OF THE STUDY 42

19. COMMENTS AND ANSWERS TO PREVIOUS EXAMINATIONS 42

20. BIBLIOGRAPHY 46

21. LIST OF APPENDIX 48

Abbreviations

ANSM: Agence Nationale de Sécurité du Médicament et des Produits de santé *(French Agency for the Safety of Medicines and Health Products)*

CCT-scan: cranial computed tomography

CNIL: Commission Nationale de l'Informatique et des Libertés *(French data protection authority)*

CT-scan: computed tomography

CRA: Clinical Research Associate

CRT: Clinical Research Technician

CSP: Code de la Santé Publique *(French Public Health Code)*

CST: Clinical Study Technician

CV: Coefficient of variation

DRCI: Délégation à la Recherche Clinique et à l'Innovation *(Clinical Research and Innovation Department)*

eCRF: electronic Case Report Form

GCS: Glasgow Coma Scale

kDa: kiloDalton

mGy.cm: milliGray.centimeter

mTBI: mild Traumatic Brain Injury

mSv: milliSievert

SAE: serious adverse events

SD: Standard deviation

SFMU: Société Française de Médecine d'Urgence *(French Society of Emergency Medicine)*

SFP: French Society of Pediatry

TBI: Traumatic Brain Injury

1. General information

1.1. Title of the research

Interventional study assessing evaluation of the interest of serum S100B protein determination in the management of pediatric mild traumatic brain injury

Running title: PROS100B

1.2. Sponsor code

Sponsor Code : PHRC N 2015 BOUVIER
N°IDRCB : 2016-A00195-46

1.3. Sponsor

Clermont-Ferrand Teaching Hospital
58 rue Montalembert
63003 Clermont-Ferrand cedex 1
France

Associate Directorate General – Clinical Research and Innovation Department
Tel: +33 4 73 75 11 95 Fax: +33 4 73 75 47 30

1.4. Coordination and monitoring

Clermont-Ferrand Teaching Hospital
58 rue Montalembert
63003 Clermont-Ferrand cedex 1
France

Associate Directorate General – Clinical Research and Innovation Department
Tel: +33 4 73 75 11 95 Fax: +33 4 73 75 47 30

1.5. Investigators

1.5.1. Coordinating investigator and collaborator

Coordinating investigator: ***Damien Bouvier (Physician-Biologist, Hosp.Practitioner assistant)***
Medical Biochemistry and Molecular Biology Clermont-Ferrand Teaching Hospital
58, Rue Montalembert 63003 Clermont-Ferrand cedex 1 France
Tel: +33 4 73 75 18 01 Fax: +33 4 73 75 18 55 dbouvier@chu-clermontferrand.fr

Co-investigator: *Prof. André Labbé (Pediatrician, Univ. Prof./Hosp. Practitioner, dept. head)*
Pediatrics Unit Estaing Hospital Clermont-Ferrand Teaching Hospital
Place Lucie et Raymond Aubrac 63003 Clermont-Ferrand cedex 1 France
Tel: +33 4 73 75 00 28 Fax: +33 4 73 75 06 09 alabbe@chu-clermontferrand.fr

Scientific collaborator: *Prof. Vincent Sapin (Biologist, Univ. Prof./Hosp. Practitioner, dept. head)*
Medical Biochemistry and Molecular Biology Clermont-Ferrand Teaching Hospital
58, Rue Montalembert 63003 Clermont-Ferrand cedex 1 France
Tel: +33 4 73 75 18 01 Fax: +33 4 73 75 18 55 vsapin@chu-clermontferrand.fr

1.5.2. Scientific collaborators and Co-investigators

Center	Biochemists (Scientific collaborators)	Pediatricians (Co-investigators)
**Limoges Teaching hospital**	**Prof. Franck Sturtz, Univ. Prof./Hospital Practitioner**<br><br>Biochemistry depatment<br>Hôpital Dupuytren<br>Limoges teaching Hospital<br>2, Avenue Martin Luther King<br>87042 Limoges cedex, France<br><br>Tel: +33 5 55 05 63 41 / +33 5 55 05 80 82<br>franck.sturtz@unilim.fr	**Prof. Guigonis Vincent, Hospital Practitioner**<br><br>Hôpital de la mère et de l'enfant<br>8, avenue Dominique Larrey<br>87042 Limoges cedex, France<br><br>Tel : +33 5 55 05 63 58<br>vincent.guigonis@unilim.fr
**Hospices Civils de Lyon**	**Dr. Régine Cartier, Hospital Practitioner**<br><br>Groupement Hospitalier Est<br>Biology and Pathology Center East<br>59, boulevard Pinel<br>69677 Bron Cedex, France<br><br>Tel: +33 4 72 35 71 90<br>regine.cartier@chu-lyon.fr	**Prof. Yves Gillet, Hospital Practitioner**<br><br>Groupement Hospitalier Est<br>Maternity and Paediatric Hospital<br>Pediatric Emergency Admissions<br>59, boulevard Pinel<br>69677 Bron Cedex, France<br><br>Tel: +33 4 27 85 56 34/+33 4 27 85 56 42<br>yves.gillet@chu-lyon.fr
**Assistance Publique des Hôpitaux de Marseille**	**Prof. Régis Guieu, Univ. Prof./Hospital Practitioner**<br>Department Head<br><br>Biochemistry Laboratory<br>Hôpital de la Timone<br>Boulevard Jean Moulin<br>13005 Marseille, France<br><br>Tel: +33 4 91 38 56 50<br>regis-pierre.guieu@ap-hm.fr	**Prof. Franck Launay, Univ. Prof./Hospital Practitioner**<br>Department Head<br><br>Pediatric Emergencies<br>Hôpital Timone Enfants<br>264 rue St Pierre<br>13385 Marseille cedex 5, France<br><br>Tel: +33 4 91 38 66 52<br>Franck.LAUNAY@ap-hm.fr
**Montpellier Teaching hopsital**	**Prof. Cristol Jean Paul, Univ. Prof./Hospital Practitioner**<br>Biochemistry and hormonology department<br>Montpellier Teaching hospital – Lapeyronie<br>371 Avenue du Doyen Gaston Giraud<br>34295 Montpellier Cedex 5<br><br>Tel: +33 4 67 33 83 14<br>jp-cristol@chu-montpellier.fr	**Dr Guyon Gaël, Hospital Practitioner**<br><br>Pediatric emergency department<br>Montpellier Teaching hospital– Lapeyronie<br>371 Avenue du Doyen Gaston Giraud<br>34295 Montpellier Cedex 5<br><br>Tel:+33 4 67 33 22 86<br>g-guyon@chu-montpellier.fr

Center	Biochemists (Scientific collaborators)	Pediatricians (Co-investigators)
**Nice Teaching Hospital**	**Dr. Pascale Bayer, Hospital Practitioner**<br><br>Biology Laboratory<br>Hôpital Pasteur<br>30, avenue de la voie Romaine<br>CS 51069<br>06200 Nice cedex 1, France<br><br>Tel: +33 4 92 03 81 63<br>bayer.p@chu-nice.fr	**Dr. Hervé Haas, Hospital Practitioner**<br><br>Pediatric Emergencies<br>Lenval Teaching Hospital<br>57, Avenue de la Californie<br>06200 Nice, France<br><br>Tel: +33 4 92 03 05 79<br>haas.h@pediatrie-chulenval-nice.fr
**Reims Teaching Hospital**	**Dr. Jean Baptiste Oudart, Univ. And Hospital Practitioner**<br><br>Biochemistry Laboratory<br>Hôpital Robert Debré<br>Reims Teaching Hospital<br>Avenue du Général Koenig<br>51092 Reims Cedex, France<br><br>Tel: +33 3 10 73 62 87 / +33 3 26 78 83 46<br>joudart@chu-reims.fr	**Dr. Yannick Plenier, Hospital Practitioner**<br><br>Pediatrics Emergencies<br>Reims Teaching Hospital<br>45, rue Cognacq- Jay<br>51092 Reims Cedex, France<br><br>Tel: +33 3 26 78 89 92<br>yplenier@chu-reims.fr
**Saint-Etienne Teaching hospital**	**Prof. Gonzalo Philippe, Univ. Prof./Hospital Practitioner**<br><br>Biologie-Pathologie departement<br>Hôpital Nord<br>Saint Etienne Teaching Hopsital<br>Avenue Albert Raimond<br>42055 Saint-Étienne cedex 2, France<br><br>Tel : +33 4 77 12 75 53<br>philippe.gonzalo@chu-st-etienne.fr	**Dr. Mory Olivier, Hospital Practitioner**<br><br>Pediatrics- Medical/Surgical Emergencies<br>Hôpital Nord<br>Saint Etienne Teaching Hopsital<br>Avenue Albert Raimond<br>42055 Saint-Étienne cedex 2, France<br><br>Tel:+33 4 77 82 81 34/+33 4 77 82 86 32<br>olivier.mory@chu-st-etienne.fr
**Toulouse Teaching Hospital**	**Prof. Bertrand Perret, Univ. Prof./Hospital Practitioner**<br><br>Biochemistry Laboratory<br>Biology Unit<br>Purpan Teaching Hospital<br>330, Avenue de Grande-Bretagne<br>31059 Toulouse Cedex, France<br><br>Tel: +33 5 67 69 03 17<br>Perret.b@chu-toulouse.fr	**Dr. Isabelle Claudet, Hospital Practitioner**<br><br>Pediatrics- Medical/Surgical Emergencies<br>Pediatrics Unit<br>Purpan Teaching Hospital<br>330, Avenue de Grande-Bretagne<br>31059 Toulouse Cedex, France<br><br>Tel: +33 5 34 55 84 79<br>Claudet.i@chu-toulouse.fr

1.6. Associate Partners

Methodologist: ***Bruno Pereira (PhD)***
Clinical Research and Innovation Department Clermont-Ferrand Teaching Hospital
Villa annexe IFSI 58, rue Montalembert 63003 Clermont-Ferrand cedex 1 France
Tel: +33 4 73 75 49 64 Fax: +33 4 73 75 47 30 bpereira@chu-clermontferrand.fr

Economist: *Charline Mourgues (MSc)*
Clinical Research and Innovation Department Clermont-Ferrand Teaching Hospital
Villa annexe IFSI 58, rue Montalembert 63003 Clermont-Ferrand cedex 1 France
Tel: +33 4 73 75 03 50 c_mourgues@chu-clermontferrand.fr

Project Leader: *David Balayssac (PhD, PharmD)*
Clinical Research and Innovation Department Clermont-Ferrand Teaching Hospital
Villa annexe IFSI 58, rue Montalembert 63003 Clermont-Ferrand cedex 1 France
Tel: +33 4 73 75 10 28 Fax: +33 4 73 75 47 30 dbalayssac@chu-clermontferrand.fr

1.7. Study sites

Center		Departments
**1**	**Clermont-Ferrand Teaching Hospital**	Pediatric Emergencies<br>Estaing Hospital<br>Place Lucie et Raymond Aubrac<br>63000 Clermont-Ferrand, France
**2**	**Limoges Teaching hospital**	Hôpital de la mère et de l'enfant<br>8, avenue Dominique Larrey<br>87042 Limoges cedex, France
**3**	**Hospices Civils de Lyon**	Groupement Hospitalier Est<br>Maternity and Paediatric Hospital<br>Pediatric Emergency Admissions<br>59, boulevard Pinel<br>69677 Bron Cedex, France
**4**	**Assistance Publique des Hôpitaux de Marseille**	Pediatric Emergencies<br>Hôpital Timone Enfants<br>264 rue St Pierre<br>13385 Marseille cedex 5, France
**5**	**Montpellier Teaching hopsital**	Pediatric emergency department<br>191, Avenue du Doyen Gaston Giraud<br>34090 Montpellier, France
**6**	**Nice Teaching Hospital**	Pediatric Emergencies<br>Lenval Teaching Hospital<br>57, Avenue de la Californie<br>06200 Nice, France
**7**	**Reims Teaching Hospital**	Pediatrics Emergencies<br>Reims Teaching Hospital<br>45, rue Cognacq- Jay<br>51092 Reims Cedex, France

**8**	**Saint-Etienne Teaching hospital**	Pediatrics- Medical/Surgical Emergencies<br>Hôpital Nord<br>Saint Etienne Teaching Hopsital<br>Avenue Albert Raimond<br>42055 Saint-Étienne cedex 2, France
**9**	**Toulouse Teaching Hospital**	Pediatrics- Medical/Surgical Emergencies<br>Pediatrics Unit<br>Purpan Teaching Hospital<br>330, Avenue de Grande-Bretagne<br>31059 Toulouse Cedex, France

1.8. Data processing

Clinical Research and Innovation Delegation
Clermont-Ferrand Teaching Hospital
Villa annexe IFSI
58, rue Montalembert
63003 Clermont-Ferrand cedex 1, France

1.9. Ethics Committee

Southeast Ethics Committee VI (IRB: 00008526)
Clermont-Ferrand Teaching Hospital
Administration centrale
BP 69
63003 Clermont-Ferrand Cedex 1, France

1.10. Estimated time frame

Ethics Committee submission: April 2016
ANSM authorization: June 2016
Study start: July 2016
Inclusion period: July 2016 – July 2018 (24 months)
Estimated end of study: August 2018
Final study report: November 2018

2. Study rationale / Scientific justification

2.1. Current state of scientific knowledge

Pediatric traumatic brain injury (TBI) can be classified into three categories – severe, moderate and mild – each of which requires a specific treatment algorithm, as recently restated by the French Society of Emergency Medicine (SFMU) (Jehlé *et al.*, 2012) and the French Society of Pediatry (SFP) (Lorton *et al.*, 2014). Severe and moderate TBI, with a Glasgow Coma Scale (GCS) score <8 or between 8 and 12, respectively, require hospitalization or transfer to the resuscitation bay for severe TBI. In pediatric patients, the management of mild traumatic brain injury (mTBI), defined by a GCS score between 13 and 15, is much more difficult than in adults, due to the type of patient. mTBI accounts for around 80 to 90% of all cases of pediatric TBI presenting to emergency rooms, and domestic accidents are the most common cause (75%). Common post-mTBI symptoms and deficits in children are not specific to mTBI and appear to resolve with time; however, limited evidence suggests that children with intracranial pathology on imaging may experience persisting symptoms or deficits (Hung *et al.*, 2014). Therefore, cranial computed tomography (CCT-scan) scans and short inpatient observation are very frequently ordered so as to be sure not to overlook any complications, which are known to occur at a rate of 0-7% (American Academy of Pediatrics, 1999). Yet, it has been very clearly established that 93% of these measures (CCT-scan and/or short hospitalization) are ultimately unnecessary (Homer and Kleinman, 1999). Reducing the number of unnecessary CCT-scans in pediatric patients is all the more important because several recent large-scale epidemiological studies have described a link between radiation exposure from CT-scans performed during childhood (effective dose 0.03 to 69.2 mSv per scan) and the risk of cancer (Pearce *et al.,* 2012; Mathews *et al.*, 2013; Miglioretti *et al.*, 2013). Indeed, iatrogenic radiation with a cumulative dose of 50 mGy may triple the risk of leukemia, and cumulative doses of 60 mGy could triple the risk of brain tumors (Pearce *et al.,* 2012). Moreover, an Australian study of 11 million children found a 24% increase in cancer risk for the 680,000 children who underwent CT-scan (including 59% CCT-scans), and a 35% increase for the 1-4 year-old age group. This study also noted that the risk of brain tumors was 2.44 times higher for children who had CCT-scan (3.24 times for the 1-4 year-old age group) (Mathews *et al.*, 2013).

The same issue arises in France, based on a recent analysis by the radiology department at Estaing Teaching Hospital in Clermont-Ferrand showing that the mean radiation exposure from a pediatric CCT-scan is approximately 400 mGy.cm (or an effective dose of 1.6 mSv). In this context, the S100B protein stands out as a relevant biomarker that could potentially be of great interest for reducing unnecessary CCT-scans and hospitalizations which form the cornerstone of the current recommendations for the management of mTBI (Jehlé *et al.,* 2012 ; Lorton *et al.*, 2014). Lorton et al. mention this perspective.

The S100B protein is a 21 kDa dimeric holoprotein discovered in 1965 by Moore during an electrophoresis study of protein extracts from human and animal brains. The name “S100 protein” derives from its solubility in a saturated (100%) ammonium sulfate solution (Beaudeux *et al.,* 1999). Its utility in clinical biology is related to its release into the extracellular medium either during overexpression of its gene (trisomy 21, Alzheimer’s disease, Creutzfeldt-Jakob disease, multiple sclerosis, melanoma) or in association with vascular or traumatic brain injury (Beaudeux *et al.,* 2001 and 2002). The protein can be detected in serum; it is eliminated by the kidneys with a half-life of around 2 hours (Anderson *et al.,* 2001). In the medical laboratory, determination of

serum levels of S100B protein is of real interest mainly in two types of pathologies: meningeal hemorrhage (Sanchez-Peña *et al.,* 2008) and TBI.

The utility of serum S100B determination for reducing unnecessary CCT-scans in the management of mTBI in adults has been well established in many studies and recently confirmed in a meta-analysis (Undén and Romner, 2010). With a serum cutoff threshold of 0.1 µg/L, the S100B protein identifies patients with positive CCT-scan lesions with a sensitivity of 100% and a specificity of approximately 30%. Since the assay must be carried out within 3 hours following the trauma (due to the half-life of the biomarker), it also appears to shorten the duration of patient management compared to CCT-scan, which is generally performed 6 hours post-trauma. In this same meta-analysis published in 2010, the authors noted a lack of convincing data in pediatric populations. Indeed, the interest of S100B in pediatric mTBI is more difficult to evaluate due to physiological variations in serum S100B concentrations, especially during the first 3 years of life (Bouvier *et al.,* 2011).

It is in this context that the Medical Biochemistry and Pediatrics departments of Clermont-Ferrand Hospital carried out a single-center, prospective study on the interest of serum S100B determination in the management of pediatric mTBI. The primary objective of this study was to establish reference intervals for children under 3 years old, since such data were not available in the literature at the time. To define these reference ranges, 186 healthy controls aged 0-3 years were analyzed (Bouvier *et al.,* 2011), yielding specific values for pediatric patients that differed from those in adults. Four age groups were defined to establish pediatric reference intervals for S100B protein (95th percentile), as follows: 0 to 3 months: 0.62 µg/L; 4 to 9 months: 0.35 µg/L; 10 to 24 months: 0.23 µg/L; and >25 months: 0.18 µg/L. Given the large standard deviation for the 0-3 month age group, it was decided to create a single age group of 0 to 9 months with a cutoff of 0.35 µg/L. In the group of children under 2 years old, serum S100B concentrations are inversely proportional to age ($r = -0.60$, $p<0.001$) with no gender-related differences. We also showed that serum S100B levels are inversely correlated with predicted head circumference according to the equation: serum S100B concentration (µg/L) = -1.884 x head circumference (meters) + 1.0455 ($r^2 = -0.96$, $p<0.001$) (Bouvier *et al.,* 2011). The secondary objective was to determine if there was a significant difference in serum S100B levels according to the severity of the TBI (mild, moderate or severe). This also yielded an idea of the discriminatory power of this protein with regards to the decision to order clinical observation or computed tomography during the conventional management of mTBI. To this end, 446 patients aged 0-18 years with TBI were prospectively enrolled in a 1-year study. A blood sample was taken within 3 hours post-trauma to assay S100B protein (Bouvier *et al.,* 2012). Serum S100B protein levels were found to increase significantly according to the severity of TBI (severe > moderate > mild). Of the 65 patients who underwent CCT-scan, 23 had positive findings (CCT+). In these pediatric patients, measurement of S100B identified patients correctly as CCT+ with a sensitivity of 100% and a specificity of 33%. These findings are in line with data obtained in adults (Unden and Romner, 2010) and with an Austrian study in 109 children (Castellani *et al*., 2009). Furthermore, in the Clermont-Ferrand study, serum S100B determination was shown to be a discriminatory test which correctly identified 21 patients as having an unfavorable clinical evolution following mTBI with a sensitivity of 100% and a negative predictive value of 100%. Lastly, of the 242 hospitalizations, 81 could have been avoided by including the S100B concentration in the decision-making algorithm. After establishing the

specific reference ranges for S100B in children under the age of 3 years, we showed that S100B assay could theoretically reduce the number of CCT-scans by 33%, thereby reducing radiation exposure, and could also reduce unnecessary hospitalizations by 33%, thereby saving costs related to the current practices for managing pediatric mTBI (Bouvier *et al.,* 2012). Recent studies confirm these results (Manzano et al., 2015 ; Simon-Pimmel *et al.*, 2015).

2.2. Hypotheses and objectives

Based on these initial results from a prospective study, a multicenter interventional study will be necessary to validate the routine use of this biomarker (Bouvier, 2013). The ultimate goal is to include serum S100B assay in the current recommendations for mTBI management based on the study of Kuppermann *et al*. (2009), as mTBI accounts for 5-8% of pediatric emergency admissions in France (60-100 per 100,000 children). The study of Kuppermann *et al.* strongly dictated the recommandations for mTBI management by the French Society of Emergency Medicine (SFMU) (Jehlé *et al.*, 2012). Then, from these 2 publications, the French Society of Pediatrics (SFP) redacted their recommendations after adjustment method recommendations for clinical practice, used by the French High Authority of Health (HAS) (Lorton *et al.*, 2014).

The use of serum S100B assay as part of the management of pediatric mTBI should make it possible to reduce the number of additional examinations, in particular a 30% reduction in the number of CCT-scans, with a resultant reduction in radiation exposure, known to be a risk factor for cancer (Pearce *et al*., 2012; Mathews *et al.*, 2013; Miglioretti *et al.*, 2013).

2.3. Summary of the benefits and foreseeable and known risks for subjects participating in the research

Determination of serum S100B protein in pediatric mTBI has a sensitivity of 100% (a positive result is associated with intracerebral lesions on CCT-scan and/or development of clinical complications) and a negative predictive value of 100% (a negative result is associated with absence of intracerebral lesions and/or development of clinical complications) (Table 1) (Bouvier *et al.,* 2012). Thus, the benefit to the patient consists in a simplified course of management compared with the conventional approach. The risks to the patient are minimal since the probability of a false negative result is virtually nul. Furthermore, the blood assay of S100B protein is minimally invasive because it requires just a single blood sampling using a micro-method.

	**Sensitivity**	**Specificity**	**Positive predictive value**	**Negative predictive value**
**Positive CCT-scan**	100% (82.5 - 100%)	33% (20 - 50%)	45% (31 - 60%)	100% (77 – 100%)
**Unfavorable clinical evolution**	100% (84 - 100%)	36% (31 - 41%)	8% (5 - 11%)	100% (97 – 100%)

Table 1: Characteristics of the serum S100B assay relative to a positive CCT-scan or an unfavorable clinical evolution in pediatric mTBI (Bouvier *et al.,* 2012) (95% confidence interval in parentheses).

2.4. Expected impact

Inclusion of serum S100B determination in the diagnostic algorithm of mTBI in pediatric patients has the potential to avoid unnecessary tests such as CCT-scans. This reduction in the number of CCT-scans performed is expected at the end of the study and would result in a reduction of exposure of the children to iatrogenic radiation.

This study will be the first multicenter study and will establish, promote and convey a new management strategy for pediatric mTBI.

In view of the literature regarding the serum S100B assay in children (no multicenter randomized study to date) and considering those for adults (Undén *et al.,* 2010), a randomized clinical trial ("conventional management" *versus* "S100B management") will assess the main objective and should lead to an international publication with a high impact factor.

2.5. References to the scientific literature and to pertinent data serving as a basis for the research

Biberthaler P, Linsenmeier U, Pfeifer KJ, Kroetz M, Mussack T, Kanz KG, Hoecherl EF, Jonas F, Marzi I, Leucht P, Jochum M, Mutschler W. Serum S-100B concentration provides additional information for the indication of computed tomography in patients after mild head injury: a prospective multicenter study. Shock. 2006 May; 25(5):446-53.

Bouvier D, Fournier M, Dauphin JB, Amat F, Ughetto S, Labbé A, Sapin V. Serum S100B determination in the management of pediatric mild traumatic brain injury. Clin Chem. 2012 Jul; 58(7):1116-22.

Bouvier D, Castellani C, Fournier M, Dauphin JB, Ughetto S, Breton M, Labbé A, Weinberg AM, Sapin V. Reference ranges for serum S100B protein during the first three years of life. Clin Biochem. 2011 Jul;44(10-11):927-9.

Calcagnile O, Undén L, Undén J. Clinical validation of S100B use in management of mild head injury. BMC Emerg Med. 2012 Oct 27;12(1):13.

Kuppermann N, Holmes JF, Dayan PS, Hoyle JD, Atabaki SM, Holubkov R et al. Identification of children at very low risk of clinically-important brain injuries after head trauma: a prospective cohort study. Lancet. 2009;374(9696):1160-70.

Mathews JD, Forsythe AV, Brady Z, Butler MW, Goergen SK, Byrnes GB, Giles GG, Wallace AB, Anderson PR, Guiver TA, McGale P, Cain TM, Dowty JG, Bickerstaffe AC, Darby SC. Cancer risk in 680,000 people exposed to computed tomography scans in childhood or adolescence: data linkage study of 11 million Australians. BMJ. 2013 May 21;346:f2360.

Undén J, Romner B. Can low serum levels of S100B predict normal CT findings after mild head injury in adults?: an evidence-based review and meta-analysis. J Head Trauma Rehabil. 2010 Jul-Aug; 25(4):228-40.

3. Study objectives

3.1. Primary objective

The primary objective is to evaluate the utility of serum S100B measurement in the management of pediatric mTBI by demonstrating a decrease in the proportion of CCT-scans prescribed in the “S100B management” intervention arm compared with the “conventional management” control arm, hypothesizing a 30% decrease in the number of CCT-scans between the intervention and control arms.

3.2. Secondary objectives

Secondary objectives are to demonstrate the utility of serum S100B measurement with respect to:

- Reduction in the time spent in the pediatric emergency room
- Reduction in the duration of hospitalization
- Reduction in radiation exposure
- Reduction in sedation and use of sedatives
- Detection of complications (intracranial lesions) by CCT-scan which can occur at a rate of 0-7% in patients with mTBI (American Academy of Pediatrics, 1999)
- Absence of intercurrent events at 48 hours and 3 weeks after mTBI
- Compliance of emergency physicians with the S100B assay
- Reduction of the cost of management

4. Description of the study

4.1. Type of study

The study protocol corresponds to a diagnostic prospective, controlled, multicenter study using a stepped wedge cluster design, in which pediatric patients (aged ≤ 16 years) presenting to the pediatric emergency room for mTBI with a GCS score of 15 will benefit from usual care (“conventional management” arm) in the control group, and from S100B management in the interventional group.

A recent systematic review (Mdege *et al.*, 2011) indicates stepped wedge cluster randomized design is particularly used to evaluate interventions during routine implementation, particularly for interventions that have been shown to be effective in more controlled research settings, or where there is lack of evidence of effectiveness but there is a strong belief that they will do more good than harm. Cluster randomized trials are often used to evaluate therapies or interventions in situations where individual randomization is not possible or not desirable for logistic (as in our study), financial or ethical reasons. A stepped wedge design is a type of crossover design in which different clusters cross over (switch treatments) at different time points. In addition, the clusters cross over in one direction only typically, from control to intervention. The first time point usually corresponds to a baseline measurement where none of the clusters receive the intervention of interest (figure 1). At baseline, all the patients will receive in this study the conventional management for mTBI. At subsequent time points, clusters initiate the intervention of interest, here the S100B management, and the response to the intervention is measured. More

than one cluster may start the intervention at a time point, but the time at which a cluster begins the intervention is randomized. Although the stepped wedge design extends the length of a randomized trial due to the presence of multiple time intervals, the nature of the design may be beneficial in certain settings. In a parallel or traditional crossover design, the intervention must be implemented in half of the total clusters simultaneously. The stepped wedge design allows the researcher to implement the intervention in a smaller fraction of the clusters at each time point. Another unique feature of the stepped wedge design is that the crossover is unidirectional. All clusters eventually receive the intervention and, in particular, the intervention is never removed once it has been implemented which may alleviate ethical and/or community concerns. This makes the stepped wedge design particularly useful for evaluating the population-level impact of an intervention that has been shown to be effective in an individually randomized trial. This point is essential in our study due to intuitive beliefs that the intervention is likely to do more good than harm. This should minimize the potential contamination bias.

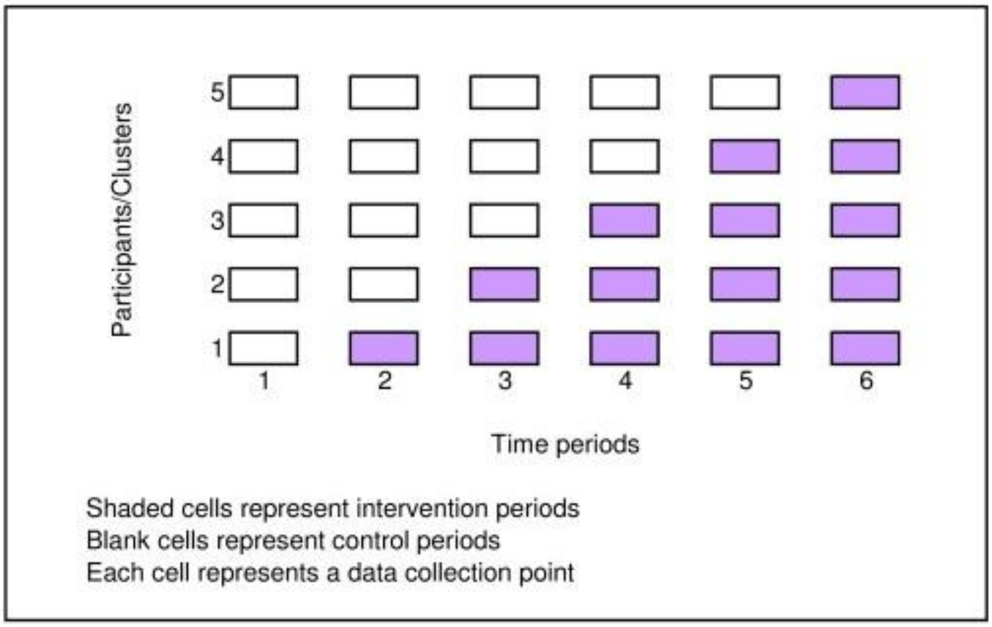

Figure 1: Stepped wedge study design of our trial. Six intervals of 4 months will be fixed over 24 months. The randomization will involve 5 steps for which 2 (to 1) centers will be included in each cluster.

4.2. Research category

The study protocol will focus on the evaluation of a diagnostic procedure.

5. Study population

The study population comprises pediatric patients (aged ≤ 16 years) presenting to the pediatric emergency room for mTBI with a GCS score between 13 and 15 (Appendix 3). mTBI accounts for 5-8% of pediatric emergency admissions, i.e. 60-100 per 100,000 children (0-16 years: 12,000 emergency room visits per year in France) (Jehlé *et al.,* 2012).

5.1. Inclusion criteria

- Age ≤ 16 years
- Therapeutic management within 3 hours after TBI

GCS score of 15 classically requiring hospitalization and/or CCT-scan as per SFP recommendations (Lorton *et al.*, 2014). These criteria are:

- For children aged under 2 years (CCT-scan or hospitalization recommended according to physician's evaluation):
    - Parietal or occipital scalp hematoma,
    - Loss of consciousness for more than 5 seconds,
    - Trauma due to serious accident (road accident with passenger ejected from vehicle or death of another person or rollover; pedestrian hit by a moving vehicle; cyclist not wearing a helmet; fall from a height greater than 0.9 meter),
    - Abnormal behavior in the opinion of parents.
- For children 2 years and older (CCT-scan or hospitalization recommended according to physician's evaluation):
    - Loss of consciousness at time of accident,
    - Vomiting,
    - Trauma due to serious accident (road accident with passenger ejected from vehicle or death of another person or rollover; pedestrian hit by a moving vehicle; cyclist not wearing a helmet; fall from a height greater than 1.5 meter),
    - Severe headache.

5.2. Non-inclusion criteria

Patient already enrolled in another therapeutic trial with drug administration

Down syndrome

Melanoma

Refusal of child

Refusal of parents or legal guardian

Trauma more than 3 hours earlier

GCS score of 13 or 14, or signs of skull fracture or lesions of the skull base (CCT-scan recommended)

Children with TBI not requiring hospitalization and/or CCT-scan as per SFP recommendations (Lorton *et al.*, 2014). This group is defined by the absence of the following criteria:

- GCS score different from 15,
- Age < 3 months,
- Seriousness of accident:
    - road accident with passenger ejected from vehicle or death of another person or rollover,
    - pedestrian hit by a moving vehicle,
    - cyclist not wearing a helmet.

- Fall:
  - of more than 0.9 m before age 2 years,
  - of more than 1.5 m after age 2 years.
- Loss of consciousness for 5 seconds or more,
- Inconsolable crying,
- Agitation, drowsiness, feeling “slowed down”, obnubilation,
- Vomiting or headache,
- Facial or cranial hematoma,
- Otorrhea, rhinorrhea,
- Child under 2 years old,
- Loss of consciousness for less than 5 seconds,
- Unusual behaviour,
- Concern of family members.

5.3. Procedure for premature treatment discontinuation

The study can be stopped for the reasons identified in advance:

- Decision of the sponsor following new knowledge about the study.

In addition, a patient can withdraw from the study for the following reasons:

- Withdrawal of consent by the patient, parents or legal guardian,
- Non compliance of the patient with the stud,
- Serious events independent to the MTBi

5.4. Exclusion period and participation in another study

No enrolled patient can participate in another clinical research study during the duration of the present study.

No exclusion period for another clinical research study is planned outside the duration of the present study.

5.5. Compensation of subjects

The subjects will receive no compensation.

5.6. Recruitment modalities

The study will run over a period of 24 months at 9 participating hospital centers (see section 1.7). Patients will be enrolled in the study, in accordance with the selection criteria defined in sections 5.1 and 5.2, by the emergency doctor who will explain to the parents the interest of the study and the study procedures.

Both parents (or holders of parental authority) of each patient enrolled in the study will have to sign a consent form (Appendix 2) after receiving verbal and written information about the study and being given time to think it over that is compatible with the study procedure and without any hospital staff being present (Appendix 1). If one of the parents is absent and unable to come to the hospital, the consent form will be signed by only one parent. If both parents are

absent, the child will not be included in the study, since the trusted person accompanying the child does not have parental authority.

The possibility that the child will refuse to participate in the study is of course taken into account. Furthermore, the information notice and consent form will only be used if the child is in a condition to sign it and not overly anxious.

After obtaining consent (from the patient, parents or legal guardian), the patient will be included in the study.

6. Study methodology

6.1. Medical evaluation criteria

6.1.1. Primary endpoint

The primary endpoint is the proportion of CCT-scans prescribed (absence/presence of CCT-scan for each patient) within 48 hours following TBI, compared between the two arms ("S100B management" intervention arm *versus* "conventional management" control arm).

6.1.2. Secondary endpoints

Secondary endpoints are:

- Duration of management defined by the time spent in the pediatric emergency department (time between emergency room admission and discharge)
- Duration of hospitalization in another hospital department for observation
- Effective radiation dose (mSv) for each CCT-scan
- Sedation and quantity of sedatives prescribed
- Presence of intracranial injury on CCT-scan
- Presence of persistent clinical signs at the telephon follow-up interview 48 hours and 3 weeks after the mTBI (Appendix 4)
- Proportion of positive/negative CCT-scans in each arm
- Cost of management

6.2. Description of study methodology

The proposed protocol is a randomized, multicenter, open, prospective, interventional study (9 centers) using a stepped wedge cluster design, with two arms:

- Control group "Conventional Care": The children in the control group will have a conventional management treatment in accordance with the SFP recommendations (Lorton *et al.*, 2014) (Figure 2).

- Intervention group "S100B management": Patients in the "S100B management" intervention arm will have blood drawn for S100B determination within 3 hours after the trauma and their subsequent management will depend on the S100B assay results, which are available 1 hour after arrival at the laboratory (Bouvier et *al.,* 2012). The S100B serum assay will be considered positive for the following values according to age (Bouvier *et al.,* 2012):

0 to 9 months: > 0.35 µg/L,
9 to 24 months: > 0.23 µg/L,
Above 24 months: > 0.18 µg/L.

For a positive test, the children will have a conventional management treatment in accordance with the SFP recommendations (Lorton *et al.*, 2014). For a negative test, the children will be discharged from the emergency department **after 6 hours of observation** (Figure 3).

The presence of persistent clinical signs 48 hours and 3 weeks after the mTBI (telephone call) will be monitored for the two groups.

This stepped wedge cluster randomization (stratified by cluster size) was chosen 1) to improve feasibility in emergency department and 2) to avoid the major risk of contamination bias in the control group. The stepped wedge design provides an innovative choice for a cluster randomized crossover trial that is subject to constraints that limits the use more conventional designs. In our study, ethical objections arising from withholding an intervention anticipated to be beneficial is a motivation for employing a stepped wedge design. Considering the number of participating centers (n= 9) and the duration of this study (24 months), it was proposed to fix 6 intervals (time periods) of 4 months each one. Then, as it was proposed in Figure 1, the randomization will involve 5 steps for which 2 (or 1) centers will be included in each cluster.

Figure 2: Decision algorithm for CCT-scan or hospitalization indication for mTBI management in control group "conventional management":

- ***For children < 2 years***

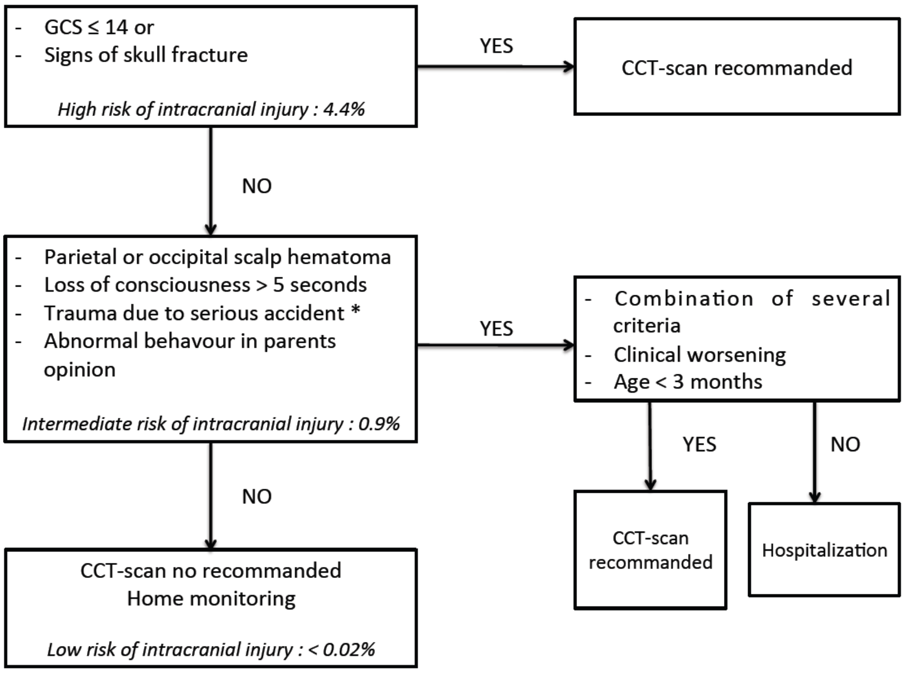

* road accident with passenger ejected from vehicle or death of another person or rollover; pedestrian hit by a moving vehicle; cyclist not wearing a helmet; fall from a height greater than 0.9 meter).

- ***For children ≥ 2 years***

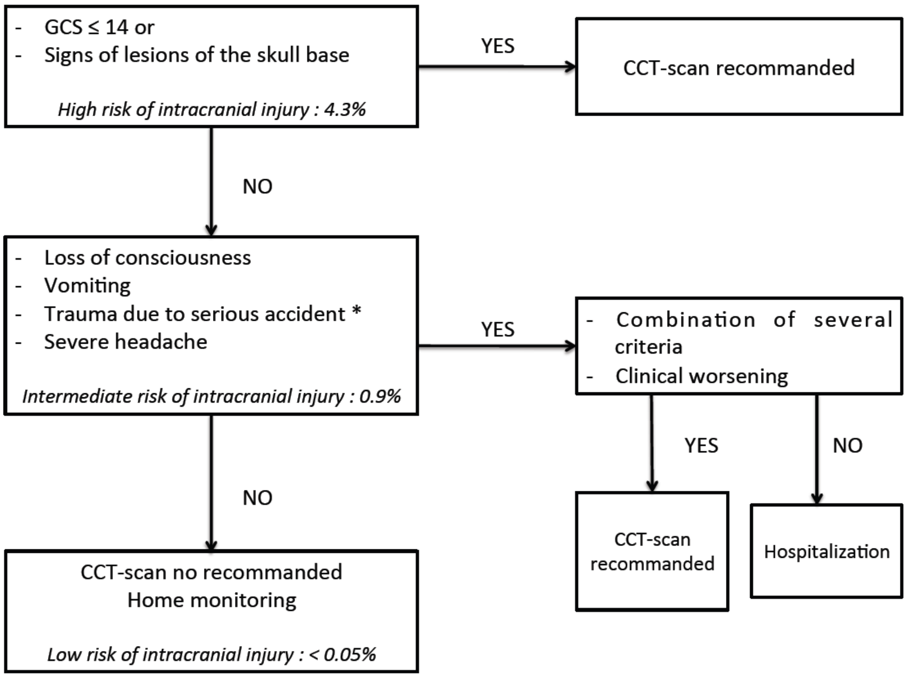

* road accident with passenger ejected from vehicle or death of another person or rollover; pedestrian hit by a moving vehicle; cyclist not wearing a helmet; fall from a height greater than 1.5 meter

Figure 3: Decision algorithm for CCT-scan or hospitalization indication for mTBI management in intervention group “S100B management”:

- ***For children < 2 years***

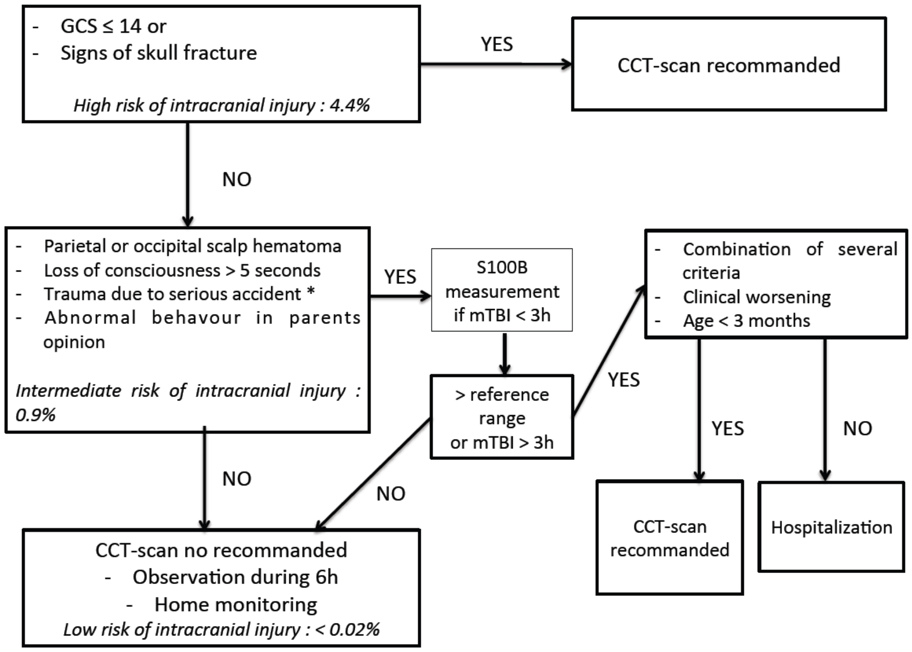

* road accident with passenger ejected from vehicle or death of another person or rollover; pedestrian hit by a moving vehicle; cyclist not wearing a helmet; fall from a height greater than 0.9 meter).

- ***For children ≥ 2 years***

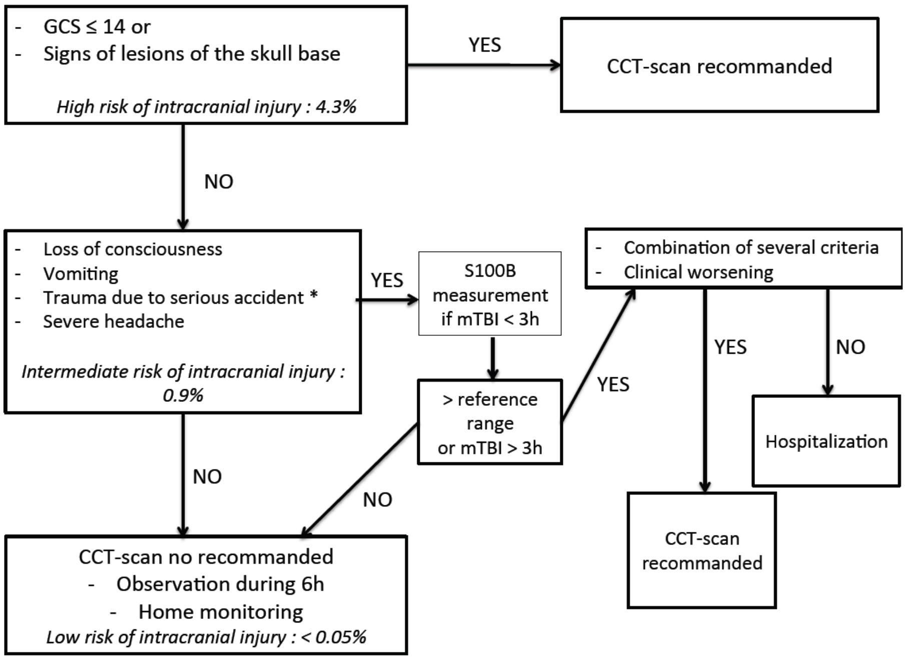

* road accident with passenger ejected from vehicle or death of another person or rollover; pedestrian hit by a moving vehicle; cyclist not wearing a helmet; fall from a height greater than 1.5 meter

6.3. Description of the measures taken to reduce and avoid bias

6.3.1. Justification of randomization arms

In view of the literature data on serum S100B protein determination in children (no randomized studies to date) and considering the data obtained in adults (Undén *et al.,* 2010), a stepped wedge cluster randomized design, with 2-arm study ("conventional management" *versus* "S100B management") will allow us to evaluate the primary endpoint in the framework of a research protocol that could lead to an international publication with a Grade A recommendation.

Nevertheless, this study is intended to be pragmatic because in the "S100B management" arm, the pediatricians will still be able to not base their management on the S100B concentration. This aspect will be taken into account in the statistical considerations.

6.3.2. Blinding bias

The context in which the study is conducted precludes any blinding with respect to the patient, the pediatrician or the biologist. However, the radiologist who analyzes the CCT-scans will be blinded as to the arm in which the patient is enrolled.

The S100B assay results, prepared by the biologist, will be reported to the pediatrician in quantitative form with comments emphasizing the following points:

- The excellent negative predictive value of the biomarker when the measured serum S100B concentration is within the age-related reference interval (Bouvier *et al.,* 2012). The aim of this precaution is to promote a change in the strategy planned by the physician before the assay (i.e., to promote observation at home without CCT-scan or hospitalization).
- The low positive predictive value of the biomarker when the measured serum S100B concentration is above the age-related reference interval (Bouvier *et al.,* 2012). The aim of

this precaution is to not change the strategy planned by the physician before the assay (CCT-scan or hospitalization according to SFP recommendations (Lorton *et al.*, 2014)).

6.3.3. Randomization

Each center will be randomized according to the stepped wedge design considered for this study. Centers will be randomly allocated by the study's statistician (block design). Stratification according to planned recruitment of each participant center ("size of center") will also be proposed. A document describing the randomization procedure will be stored confidentially at the DRCI of the Clermont-Ferrand Hospital. All patients who will have the selection criteria and sign the consent form will be included in the same arm of a center.

The stepped wedge design also imposes some practical implementation challenges, such as preventing contamination between intervention participants and those waiting for the intervention and ensuring that those assessing outcomes are blind to the participant's status as intervention or control to help guard against information bias. Concerning contamination of physician waiting for the intervention, a visit or a video conference precising the exact nature of the intervention will be only performed in each center during the 15 days preceding the shift from the control period to the intervention period, in order to limit modification of usual practice in the management of control patients. Over the duration of the study, it is almost impossible to blind physicians performing outcome assessment since they will be aware of the "step" from control to intervention status. We will control for bias by assessing and grading outcomes according to pre-defined criteria and by assessing "subjective" outcomes by a blinding physician not involved in the pediatric care.

6.3.4. Reproducibility of the assay

The analytical method used in the study (at all study centers) is based on an electrochemiluminescence assay (on Roche Diagnostics® instruments). This is currently the most suitable technology because it is an automated piecemeal technology that is the most sensitive on the market. The previous publications from the Clermont-Ferrand group (Bouvier *et al.,* 2011 and 2012) have demonstrated the excellent feasibility of this technology for use in the future study. Its analytical performance (coefficient of variation of 3.1%) requires only a single determination (no need for duplicates) without risk of analytical error. The Clermont-Ferrand group confirms thes analytical performances (Bouvier *et al.*, 2015) The test sample volume (20 µL) and dead volume (150 µL) are altogether suitable for the type of blood sampling (micro-method) chosen to be least traumatic to the children. On the other hand, because of this small volume, it will not be possible to use the other commercially available assay (DiaSorin®), though in any case the pediatric reference values for this method are not available, which would have posed a problem of interpretation were we to have used this method.

6.3.5. Control of attrition bias

Missing data about primary endpoint are not expected in this short-term study (3 weeks).

7. Practical conduct of the study

7.1. Detailed description of procedures (description of each visit)

- ***« Conventional management » control arm***
  - CCT-scans and/or hospitalization will be prescribed by the physician in accordance with SFP recommendations
  - The Clinical Research Technician (CRT) from the study center will conduct a telephone interview 48 hours and 3 weeks after the trauma to ask a number of standardized questions (Appendix 4) and determine whether there has been any deterioration. The telephone number of the parents or legal guardian will be indicated on the consent form (Appendix 2).

- ***« S100B management» intervention arm***
  - Serum S100B concentrations will be determined on a peripheral venous blood sample drawn into a dry tube with separator gel, preferably by the micro-method technique (approximately 1 mL of blood), within the first 3 hours after the trauma.
    - If serum concentrations are above the reference interval (see section 6.2), a CCT-scan and/or hospitalization will be prescribed as per SFP recommendations (Lorton *et al.*, 2014).
    - If serum concentrations are within the reference range, the patient will be discharged after 6 hours of observation and the parents will be instructed on how to observe the child at home for the next 24 hours.

  - The CRT from the study center will conduct a telephone interview 48 hours and 3 weeks after the trauma to ask a number of standardized questions (Appendix 4) and determine whether there has been any deterioration. The telephone number of the parents or legal guardian will be indicated on the consent form (Appendix 2).

Some patients may undergo double window CCT-scan without contrast injection, with one window for the central nervous system (parenchyma window) and the other for the bones of the cranium (osseous window, with analysis of the vault and base of the skull, the cervico-occipital joint and the face). CCT-scan will be performed on multiarray machines. Patients who underwent CCT-scan will be classified into two groups according to the radiology report: CCT+ (presence of intracerebral lesion(s), and CCT- (no sign of intracerebral injury), but the radiologists will not know the result of the S100B protein assay. The effective radiation dose will be recorded (mSv) from the machine display.

7.2. Description of the general logistical organization of the study

Patients will be recruited in the pediatric emergency admission departments under the responsibility of the investigator at each center.

For the "S100B management" intervention arm, the blood required for serum S100B determination will be drawn in the pediatric emergency department and the blood sample will be conveyed to the medical biology laboratory at the same center. The laboratory biologist will then communicate the assay result to the investigator.

At each center, a CRT will collect all data required for the study.

7.3. Samples and biological analyses

For the “S100B management” intervention arm, the serum S100B assay will be carried out in the medical biology laboratory at each center (with the same technique of immunoassay in the 9 centers) under the responsibility of the biologist participating in the study.

An aliquot of each serum sample will be stored in a dedicated serum bank in case it needs to be retested at a later date. The sample will then be destroyed after the results are exploited.

The immunoassay from Roche Diagnostics® holds CE marking for *in vitro* quantitative determination of S100B protein on human serum on any of the following instruments: Elecsys 1010, Elecsys 2010, Modular Analytics E 170, cobas e411 and cobas e601. The assay is based on electrochemoluminescence (ECLIA) with a total analytical cycle of 18 minutes comprising two incubation steps, followed by luminescence reading on a photomultiplier. The results are obtained with the aid of a two-point calibration curve generated for the instrument used and a reference curve memorized in the bar code of the reagent. The instrument automatically calculates the S100B protein concentration in each sample and expresses the results in µg/L. The result is not altered by icteria (bilirubin < 428 µmol/L), hemolysis (hemoglobin < 10 g/L) or lipemia (intralipid < 15 g/L). No hook effect has been observed for S100B protein concentrations of up to 100 µg/L. The range of measurement (defined as the limit of detection and the maximum of the reference curve) is 0.005 to 39 µg/L. Functional sensitivity is less than 0.02 µg/L. There is no cross-reactivity with S100A protein dimers. The coefficients of variation (VC) calculated on pooled human serum and on controls show an intra-series VC which is always less than 2.1%, implying an overall precision always less than 2.8% (Appendix 5).

The kit comprises three ready-to-use reagent bottles with the information required to perform the test memorized on the bar codes of each reagent bottle, which is enough for 100 assays. The composition of the three bottles is given in the product sheet (reference 03175243) in Appendix 5. The kits must be stored between +2 and +8°C and are stable when installed on the analytical instrument for a period of 4 weeks.

As indicated above, each kit is calibrated with CalSet calibrators (Roche Diagnostics®) according to the supplier’s recommendations. To guarantee that the assay is accurate and reproducible, two Elecsys PreciControl (low and high level) quality controls (Roche Diagnostics®) will be done daily. Serum from a patient will be centrifuged at 4000 rpm for 10 minutes and then assayed. Considering the controls performed and the proposed VCs, the determinations will be made on a single sample. The medical biology laboratory at each center is able to implement all the recommendations for use related to this assay.

7.4. Planned duration of participation and study time frame

Estimated study duration: 2 years and 3 months

Inclusion period: 24 months

Study start (first patient in): July 2016

Study end (last patient out): August 2018

Total duration of patient participation: 3 weeks

The date of the end of the study will be communicated to the competent authority and to the Ethics Committee within 90 days.

In the event of early study termination, the information will be communicated to the competent authority and to the Ethics committee within 15 days.

8. Study product

This is a diagnostic study. There are no unauthorized medicines and/or non-pharmacological treatments during the study.

8.1. Description of study product

Not applicable

8.2. Presentation of the products

Not applicable

8.3. Use of the product

Not applicable

8.4. Authorized and unauthorized medicines and treatments during the study

Not applicable

9. Data collected

The following information will be recorded on the case report form for each patient:

- Group in the study in which we included the child (control or interventional)

- Patient characteristics:
    - Identification code (center number + inclusion number + patient's initials)
    - Distance between home and hospital
    - Age
    - Gender
    - Current treatments
    - Intercurrent diseases

- Information about the mTBI:
    - Type of accident
    - Date and time of mTBI
    - GCS score at arrival at emergency department
    - Clinical signs:
        - presence (+) or absence (-) of signs of impaired consciousness
        - presence (+) or absence (-) of signs of skull fracture
        - presence (+) or absence (-) of signs of fracture of skull base
        - presence (+) or absence (-) of parietal or occipital scalp hematoma

  - presence (+) or absence (-) of loss of consciousness for more than 5 seconds in children under 2 years old
  - presence (+) or absence (-) of serious accident (road accident with passenger ejected from vehicle or death of another person or rollover; pedestrian hit by a moving vehicle; cyclist not wearing a helmet, fall from a height greater than 0.9 meter (in children under 2 years old) or 1.5 meter (in children over 2 years old))
  - Presence (+) or absence (-) of immediate loss of consciousness in children over 2 years old
  - Presence (+) or absence (-) of vomiting in children over 2 years old
  - Presence (+) or absence (-) of abnormal behavior in the opinion of parents in children under 2 years old
  - Presence (+) or absence (-) of severe headache in children over 2 years old

- Information related to serum S100B assay:
  - Date and time of blood sampling (time between mTBI and blood sampling)
  - Serum S100B concentration in µg/L
  - Concentration higher (+) or lower (-) than age-related reference value

- Information related to CCT-scan:
  - Presence (CCT+) or absence (CCT-) of intracranial trauma
  - Nature of lesion in case of CCT+
  - Effective radiation dose per patient (mSv)

- Information about the stay in the pediatric emergency department:
  - Date and time of arrival at emergency department (time between mTBI and arrival at emergency department)
  - Length of stay in emergency room
  - Length of stay in other hospital departments
  - Presence (+) or absence (-) of hospitalization
  - Indication (+) or absence of indication (-) for CCT-scan

- Information from the follow-up at 48 hours and 3 weeks post-mTBI: positive (+) or negative (-) answer to the standardized questions (Appendix 4)

- Information related to the cost of management
  - S100B monitoring
  - CCT-scan
  - Hospitalization by departments and other later hospitalization.

10.Statistical considerations

From a statistical viewpoint, the key characteristic of the stepped wedge cluster design is that the individual units within a cluster are correlated and this feature must be incorporated into

power calculations and the trial analysis. Analysis of the stepped wedge design is complex, particularly because of the need to control for temporal trends in outcome variables, such as fluctuations in disease prevalence over the course of the trial and accounting for repeated measures on the same individual. Hussey and Hughes (2007) provide a guide to the design and analysis for stepped wedge cluster randomized controlled trials, including the approaches to data analysis, sample size calculation, and power calculation. They suggested that power calculations should take into account the intra-cluster correlation, number of randomization steps, and treatment delay. As it was previously suggested, considering the number of participating centers (n= 9) and the duration of this study (24 months), it was proposed to fix 6 intervals (time periods) of 4 months each. Then, the randomization will involve 5 steps for which 4 (to 5) centers will be included in each cluster.

10.1. Sample size estimation

For an individual randomized trial, to show a 30% reduction in the rate of CCT-scans prescribed in the “S100B management” arm *versus* the “conventional management” arm, with 80% power and a two-sided risk I error of 5%, a sample of 800 subjects per arm is required. In fact, it is important to take into account that, based on the literature, the proportion of CCT-scans ordered during conventional management is roughly 20% (between 10% and 30%; 14% at Clermont-Ferrand Hospital) (Bouvier *et al.,* 2012). Therefore, to show a difference between the two arms of 6%, i.e. a 30% relative reduction (Bouvier *et al.,* 2012), a sample of 615 subjects per arm is required for an analysis using a chi-squared test for repeated clustered data. Furthermore, in light of the publication of Calcagnile *et al.* (2012), it is expected that 30% of physicians treating patients in the “S100B management” arm will not base their practice on the result of the S100B assay. Thus, in consideration of these points, it is planned to enroll 800 subjects per arm, so as to conserve a power of 80% with the previously defined hypotheses (Machin, 2004).

A fundamental assumption for randomized controlled trials is that the outcome for an individual patient is completely unrelated to that for any other patient - they are said to be 'independent'. This assumption is violated in cluster randomized clinical trials because patients within any one cluster (center in our case) are more likely to respond in a similar manner. A measure of this similarity is known as the intra-correlation coefficient (ICC). Because of this lack of independence, sample sizes require to be inflated. The sample size estimation for stepped wedged cluster randomized trials should consider ICC, variability of center population size (9 centers, coefficient of variation of cluster size (Eldridge *et al.*, 2006) defined as the ratio of the standard deviation of cluster sizes (45) to the mean cluster size (120 - 250) according to data given by participants centers), number of randomization steps (5 steps for which 2 (or 1) centers will be included) and duration of participation for each patient. Several simulations were proposed in relation to the value of ICC according to literature (Mdege *et al.*, 2011) and database of ICCs related by the University of Aberdeen (http://www.abdn.ac.uk/hsru/research/delivery/behaviour/methodological-research/).

According to these aspects (ICC=0.01 to 0.05, (Adams *et al.*, 2004)), 5% of lost to follow-up and the feasibility of this study (ability to recruit the estimated sample size), 2000 patients by group will be needed.

10.2. Data analysis: general points

The principal analysis will be performed using the software Stata (version13, StataCorp, College Station, TX). The tests will be two-sided, with a type I error set at α=0.05.

Quantitative variables will be presented as mean ± SD when normally distributed (assumption of normality studied by Shapiro-Wilk test), and for non-normal distributions as median, quartiles and range. Qualitative variables will be expressed as numbers and associated percentages. When possible, analyses will be displayed in graphs.

Inter-group comparisons will systematically be made 1) without adjustment and 2) adjusting on factors liable to be biased between groups.

10.3. Characteristics of patients at baseline

The two groups will be compared for the following patient characteristics at baseline: compliance with selection criteria, epidemiologic characteristics, clinical characteristics and treatments. Protocol violations and protocol violations per patient, as well as reasons for study dropout will also be described. The number of patients enrolled and the accrual curve will be presented for each arm.

10.4. Primary analysis

To compare the proportion of CCT-scans prescribed, a random-effect model taking into account center effect will be proposed more especially a generalized linear mixed model (logit according to statistical distribution). Randomization groups, steps of randomization, time periods and their interactions were evaluated as fixed effects. A robust Poisson mixed model should be used to complete these results in order to present results as relative risks and 95% confidence intervals.

10.5. Secondary analyses

Inter-group comparison on the other assessment criteria detailed previously will be based on the same models proposed for aim analysis to take into account between and within center variability: linear (duration of management, duration of hospitalization, effective radiation dose, quantity of sedatives prescribed – If necessary, a transformation to access the normality statistical distribution should be envisaged using log transformation for example) or generalized linear mixed model (age groups defined by age-related S100B cut-off values, sedation yes/no, presence of intracranial lesions on CCT-scan, presence of persistent clinical signs at the telephone interview) according to dependent variable. Adjusted analyses should be performed according to the univariate results and for clinically relevant parameters. Random-effects models were also used to study correlated longitudinal data (48 hours and 3 weeks) considering subject patient as random effect. These last models (with random effects) can also be used to take into account possible inter and intra physician variability in decision-making regarding the primary endpoint.

10.6. Economic analyses

The main objective of the economic analysis is to measure and to compare costs in the two arms, with early blood test of the protein S100B (intervention group) *versus* current management (control group).

We choose to perform a cost minimization analysis because for both, intervention or control group, the clinical endpoint is the same. Protein S100B blood test enables to care patient appropriately, to detect earlier TBI and to prevent from performing useless CT-scan.

The cost analysis will be performed under a University Hospital perspective. Our study will be focused on the analyses of costs of care and avoided costs in each arm. French hospital financing is based on a pricing scale for any current medical care. When an innovative medical care is developed, the financing system is temporarily based on grants. We hypothesize that the financing system for innovative medical care finances protein S100B blood test and that the price will be closed to the future price out of the regular pricing scale. Considering the French pricing system, if the number of CT-scan performed for cranial trauma decrease, due to the blood test of the protein S100B, the hospital might lose incomes. But, according to the waiting time for this exam and the public/private competition, we hypothesize that incomes remain stable because each avoided CT-scan will be replaced by another one, irrespective of the medical motive.

Costs will be analysed by microcosting during the standard follow-up of 3 weeks planned in the study: costs of S100B monitoring, costs of the CT-scans including medical costs if children should be under sedation during the CT-scan and costs of hospitalization by departments and other later hospitalizations.

So, the cost analysis focuses on avoided costs thanks to protein S100B blood test. We do not consider other consequences such as the impact of quicker appropriate care, the decrease of radiation withdrawal effects by reducing inappropriate CT-scan and the lower risk of healthcare-associated infections due to a shorter length of stay.

10.7. Method for managing missing, unused or invalid data

In view of the dispositions taken in this protocol, there is not expected to be any missing data on the primary endpoint.

Regarding other secondary endpoints, a sensitivity analysis will be proposed to define the statistical nature of possible missing data and propose the most appropriate method of imputation.

10.8. Persons in charge of analysis

Statistical analysis will be performed by Bruno Pereira (PhD in biostatistics), of the Clermont-Ferrand University Hospital Clinical Research and Innovation Delegation. Methodological developments on cluster randomized trials were BP's PhD theme (2008). BP is implied in several cluster randomized trials (see ref below) and gives his expertise about this subject for many institutes as INPES, INCa and WHO.

Vaillant-Roussel H, Laporte C, **Pereira B**, Tanguy G, Cassagnes J, Ruivard M et al. Patient education in chronic heart failure in primary care (ETIC) and its impact on patient quality of life: design of a cluster randomised trial. BMC Fam Pract. 2014 Dec 24;15:208.

Laporte C, Vaillant-Roussel H, **Pereira B**, Blanc O, Tanguy G, Frappé P et al. CANABIC: CANnabis and Adolescents: effect of a Brief Intervention on their Consumption--study protocol for a randomized controlled trial. Trials. 2014 Jan 30;15:40.

Sancho-Garnier H, **Pereira B**, Césarini P. A cluster randomized trial to evaluate a health education programme "Living with Sun at School". Int J Environ Res Public Health. 2012 Jul;9(7):2345-61.

Medico-economic objectives will be analysed by Charline Mourgues (MSc Economist), of the Clermont-Ferrand University Hospital Clinical Research and Innovation Delegation.

Mourgues C, Gerbaud L, Leger S, Auclair C, Peyrol F, Blanquet M et al. Positive and cost-effectiveness effect of spa therapy on the resumption of occupational and non-occupational activities in women in breast cancer remission: a French multicentre randomised controlled trial. Eur J Oncol Nurs. 2014 Oct;18(5):505-11.

11. Safety assessment – Management of adverse events

The investigator is responsible for reporting all adverse events on the case report form.

11.1. Definitions

Adverse event: any untoward medical occurrence in a patient or clinical investigation subject administered a pharmaceutical product and which does not necessarily have to have a causal relationship with the research or with this treatment.

Adverse effect: any untoward response related to the research.

Serious adverse effects are subgrouped as follows:

- ***Expected** serious adverse event*: any event that is described in the most recent version of the Investigator's Brochure or in the Summary of Product Characteristics for marketed medicinal products, or in the instruction notice when the research concerns a medical device which is subject to CE marking. This definition also applies to an investigational medicinal product when administered for a same population outside the labeled indications.

- ***Unexpected** serious adverse event*: any event, the nature, severity or outcome of which is not consistent with the information in the most recent version of the Investigator's Brochure or the Summary of Product Characteristics for a marketed medicinal product or the information notice for a medical device.

Serious adverse event or effect: any undesirable event or effect which results in death, is life-threatening, requires in-patient hospitalization or prolongation of existing hospitalization, results in persistent or significant disability/incapacity, or is a congenital anomaly/birth defect.

The term "life-threatening" refers to an event in which the patient was at risk of death at the time of the event, independently of the consequences of corrective or palliative treatment.

The terms "*disability*" or "*incapacity*" refer to any clinically significant, temporary or persistent disability.

Death, regardless of the cause, including when it corresponds to progression of the disease under treatment, is considered a serious adverse event.

Other events which do not correspond to the above definitions can be considered "*potentially serious*", in particular certain laboratory anomalies. The investigator or sponsor's medical judgement can result in such events being reported in the same manner as "serious" events. It is necessary for study protocols to specify the characteristics of "potentially serious" events that are subject to reporting.

New information: event concerning the conduct of the research or the development of the medicinal product or related product which is the object of the research, when said new information may jeopardize the safety of the research subjects. Examples include:

- an increase in the rate of occurrence of serious events;
- serious adverse events related to the clinical trial procedures;
- lack of efficacy with a medicinal product used to treat life-threatening disease;
- a major safety finding from animal studies that provides new information on the safety of the product;
- and generally, any new information that could lead to an unfavorable reassessment of the benefit/risk ratio of the research.

Any new information concerning the research (or the product used) which may jeopardize the safety of the research subjects will be subjected to appropriate urgent measures and prompt and timely notification by the Sponsor to the competent authority and the Ethics Committee.

The only expected adverse effect is a risk of false negative results. However, this risk is virtually absent, in view of the sensitivity and the negative predictive value of the assay and the fact that the assay can only be done within 3 hours after the mTBI (see section 2.3 Summary of the benefits and foreseeable and known risks for research subjects).

11.2. Serious adverse event reporting

It is the investigator's obligation to report within 24 hours any serious adverse event occurring in any patient enrolled in a study:

- during the active phase of the study,
- in the weeks following cessation of treatment,
- within the deadlines established for safety monitoring off treatment, before (wash-out or withdrawal phase) or after the active phase,
- after termination of the study, regardless of the time of the event, when no cause other than the research can reasonably be incriminated,

on the "Serious adverse event report form" (Appendix 7), indicating the date of onset, the severity, the causal relationship with the treatment (or product), and the follow-up/outcome.

The narrative describing the event should be completed and transmitted to the sponsor as soon as new, pertinent information is received. Depending on the nature and seriousness of the event, copies of the patient's anonymized medical record can be attached, as well as laboratory results.

When a serious adverse event persists at the end of the study, the investigator will continue to follow the patient until said event is considered resolved.

In accordance with the implementing decree 2006-477 of 26/04/2006 amending chapter 1 of title II of Book I of the first part of the Public Health Code relating to biomedical research, all suspected unexpected serious adverse effects must be reported by the sponsor to ANSM and to the Ethics Committee at first knowledge and no later than:

- 7 days after occurrence in case of death or a life-threatening event
- 15 days after occurrence for all other unexpected serious adverse events (SAE).

The sponsor will decide upon the significance of the serious adverse events that it reports and the consequences thereof, in particular with respect to the conduct of the research.

The sponsor will also assess the causality of the adverse event with the research by means of a joint analysis with the Regional Pharmacovigilance Center.

In the framework of this study, no expected serious adverse events are anticipated.

The sponsor will maintain a detailed list of all adverse events reported by the investigator(s).

Once per year, or on request, the sponsor will submit an annual safety update report to ANSM and to the Ethics Committee containing all available safety information.

The sponsor will also provide the investigators with any information that may affect the safety of the research subjects.

11.3. Monitoring Committee

An independent monitoring committee will be created, composed of:

Biochemist: ***Prof Jean-Louis Beaudeux***
Hôpital Necker-Enfants Malades
Biochemistry department
149 rue de Sèvres
75015 Paris, France

Pediatrician: ***Prof Christelle Gras-Le Guen***
Pediatric emergency department
CHU de Nantes
38, boulevard Jean-Monnet
44093 Nantes cedex 1, France

Christelle Gras-Le Guen is an author of the "New recommendations for the management of children after minor head trauma" (Lorton *et al.*, 2014).

Methodologist ***Dr Amélie Anota***
Biostatistics unit
Centre Léon Bérard
28 Prom. Léa et Napoléon Bullukian
69008 Lyon, France

This independent monitoring committee will meet a first time at study initiation and then throughout the duration of the study at its own initiative or at the sponsor's request. The committee will also issue a general opinion on the conduct of the study and can aid decision-making under the following circumstances:

- Opinion on premature study termination (because the study is no longer practicable or because the information needed to draw conclusions has already been obtained),

- Opinion on substantial protocol amendments that have become necessary for reasons of recruitment or monitoring, or to take account of new scientific data. All substantial protocol amendments will be submitted to the independent committee prior to submission to the sponsor, then to the Ethics Committee and to ANSM.

The Monitoring Committee's opinion will be submitted in writing to the sponsor.

11.4. Termination of the study

The study can be terminated for the following reasons:

- Notification of an excess rate of SAE
- Decision of the sponsor following new knowledge about the study product
- Decision of the investigator

11.5. Follow-up of patients presenting an adverse event

Patients with persistent serious adverse events at the end of the study will continue to be followed up by the investigator until the event is considered resolved. Patients who had non-serious adverse events will be followed up until the final study visit.

12. Right of access to source documents and data

12.1. Access to data

The sponsor is responsible for obtaining the agreement of all parties involved in the research in order to guarantee direct access to all study sites, source data, source documents and reports for purposes of the sponsor's quality control and audit.

The investigators will provide access to the documents and individual data that are strictly necessary for purposes of monitoring, quality control and audit of the biomedical research, to the persons authorized to consult said documents pursuant to the legislative and regulatory provisions in force (articles L.1121-3 and R.5121-13 Public Health Code).

12.2. Source data

Source documents, defined as any original document or object which proves the existence or accuracy of data or information recorded during the clinical study, will be stored for a period of 15 years by the investigator or by the hospital in the case of a hospital medical record.

12.3. Data confidentiality

Subject to the provisions relating to the confidentiality of data to which persons in charge of quality control of biomedical research have access (article L.1121-3 Public Health Code), and subject to the provisions relating to the confidentiality of information as concerns in particular the nature of the products being studied, the trials, the persons undergoing the research and the results obtained (article R.5121-13 Public Health Code), persons having direct access shall take all necessary precautions to ensure the confidentiality of the information relating to the products being studied, the trials, the persons undergoing the research and notably their identity, and the results obtained.

These persons, as well as the investigators themselves, are bound by professional secrecy (in accordance with the conditions laid down in articles 226-13 and 226-14 of the penal code).

During the biomedical research or upon its completion, the data collected on the research subjects and transmitted to the sponsor by the investigators (or any other specialized study staff) shall be rendered anonymous.

In no case shall the names or addresses of the persons undergoing the research appear.

Anonymity of the subjects will be guaranteed by the creation of a subject code corresponding to 5 numerals and 2 letters, as follows: study center number (2 numerals) + inclusion number at center (3 numerals) + patient's initials (last name, first name).

The sponsor will ensure that each research subject has given his written consent allowing access his personal data which is strictly necessary for quality control of the research.

12.4. Registration in the national file of biomedical research subjects

Not applicable

13. Quality control and assurance

13.1. Engagement of the investigators and the sponsor

The investigator undertakes to conduct the study in compliance with public health law 2004-806 of 9 August 2004 relating to biomedical research, the implementing decree 2006-477 of 26/04/2006 amending chapter 1 of title II of book 1 of the first part of the Public Health Code relating to biomedical research, and with the bylaws in force.

The study will also be conducted in compliance with Good Clinical Practices for biomedical research on medicinal products for human use, as laid down in article L.1121-3 Public Health Code and the decree of 24 November 2006.

The investigator also undertakes to comply with the Declaration of Helsinki of the World Medical Assembly (Tokyo 2004, revision).

13.2. Quality Assurance

A Clinical Research Associate (CRA) designated by the sponsor will ensure the proper conduct of the study, the collection of data generated in writing, and their documentation, recording and reporting, as per the Standard Operating Procedures in effect at the Clermont-Ferrand Hospital and in compliance with Good Clinical Practices and legislative and regulatory provisions in force.

13.3. Quality Control

The investigator guarantees the authenticity of the data collected during the study and accepts the legal provisions authorizing the study sponsor to implement quality control.

The coordinating investigator and associated investigators therefore agree to make themselves available during Quality Control visits by the Clinical Research Associate that will be scheduled at regular intervals. The following items will be examined at each visit:

- Informed consent
- Compliance with the study protocol and procedures

- Quality of data recorded in the case report forms: accuracy, missing data, coherence with source documents (medical records, appointment calendars, original copies of laboratory results, etc.)
- Management of any study products.

13.4. Case report form

All the information required by the study protocol will be recorded in an electronic case report form (eCRF). Data will be collected as it is obtained and transcribed in a clear and legible manner in the case report form.

The eCRF will be developed and accessible via the internet (SSL 128-bit SSL encryption). Access will be controlled by a personal password and all consultations and changes will be logged. Data will be entered in single input on the eCRF at each study center.

Data will be validated according to the data management plan jointly established between the coordinating investigator and the DRCI (methodologist, data manager and statistician). ACCESS® and STATA® software will be used. Data will be frozen/unfrozen according to standard procedures at Clermont-Ferrand Hospital (raw data frozen in XML format and as EXCEL® spreadsheets). All the data will be saved every night, kept for 4 weeks, then backed up to tape every month.

14. Ethical considerations

14.1. Ethics Committee

The study protocol, patient information notice and consent form and the case report form for the study will be submitted to the Southeast VI Ethics Committee for an opinion.

Notification of a favorable opinion from the Ethics Committee will be transmitted to the sponsor and to ANSM. A study authorization application will be sent by the sponsor to ANSM prior to study start.

14.2. Information for patients and written informed consent form

Patients and the parents or legal guardians will be informed in complete and faithful terms and in understandable language of the objectives and constraints of the study, the potential risks, the required observation and safety measures, and their right to refuse to participate in the study or to revoke their consent at any time. The investigator must also inform the subjects of the Ethics Committee opinion.

All this information appears in an information notice and consent form given to the patient (Appendix 1). The free, informed and written consent of the patient will be obtained by the investigator (Appendix 2). These documents (Appendix 1 and 2) are approved by the competent Ethics Committee and are to be used for the study in question, to the exclusion of any other document.

Two original copies will be co-signed by both the investigator and the patient and the parents or legal guardian. The second copy is to be kept in the patient’s medical record.

14.3. Protocol amendments

Protocol amendments must be qualified as substantial or non-substantial. According to their nature, they will be the object of a new Ethics Committee opinion and/or authorization from the competent authority.

14.4. Management relating to the research

Patients in the “conventional management” control arm will be managed for mTBI according to SFP recommendations (Lorton *et al.*, 2014).

Patients in the “S100B” intervention arm will have a single blood sample drawn by a micro-method (total of 1 mL of blood) in a first step. They will then be managed according to the result of the serum S100B concentration:

- Serum S100B level below the cutoff defined according to age group: the patient will be discharged and return home after 6 hours of observation.
- Serum S100B level above the cutoff defined according to age group: the patient will be hospitalized and managed according to SFP recommendations (Lorton *et al.*, 2014).

In the framework of the study, parents or legal guardians of all enrolled patients must answer the follow-up questions at 48 hours and 3 weeks.

15. Data processing and storage of study documents and data

15.1. Data entry and processing

Data will be entered on an electronic CRF by the investigators and/or CRT at each study center.

The statistical analysis of the data will be carried out at the DCRI of Clermont-Ferrand Hospital by Bruno Pereira (methodologist, biostatistician).

15.2. CNIL

This study enters in the scope of “Reference Methodology” (MR-001) in application of the provisions of the law of 6 August 2004 relating to the protection of natural persons with regard to the processing of personal data and amending the law of 6 January 1978 relating to computer processing, data files and civil liberties. This change was approved by decision of 5 January 2006. Clermont-Ferrand Hospital, the study sponsor, has signed a commitment to comply with this “Reference Methodology” on 15/03/2007.

15.3. Record-keeping

The following documents will be archived under the study name in the Biochemistry department (Prof. Vincent Sapin) at Clermont-Ferrand Hospital until the end of the period of practical usefulness (24 months inclusion + 3 months for data analysis).

These documents are:

- Protocol and appendices, and any amendments,
- Signed, original information notices and consent forms,
- Individual data (authenticated copies of raw data),

- Follow-up documents
- Statistical analyses
- Final study report

At the end of the period of practical usefulness, all documents to be archived, such as defined in procedure PG.06.005 “Management of documentation relating to protocols” of Clermont-Ferrand Hospital will be transferred to the central archives and placed under the sponsor’s responsibility for a period of 15 years after study completion, in accordance with institutional practices.

These documents cannot be moved or destroyed without the sponsor’s permission. After the 15 years are up, the sponsor will be consulted for destruction. All the data as well as all documents and reports may be subject to audit or inspection.

16. Funding and insurance

16.1. Study budget

The budget is obtained from a PHRC (Hospital Clinical Research Program). The budget obtained is 538 186 €.

Some centers are not equipped with an automated instrument (Cobas, Roche Diagnostics) available 24/24 and 7/7, in which case it will be necessary to rent one (Cobas E411 Rotor with inverter). Roche Diagnostics France has agreed to offer a significant discount, since the catalogue price for renting this instrument is roughly 36,000 €. Each center not equipped with an automated instrument will also benefit from a two-way connection between the rented instrument and the laboratory’s computer system, thereby enabling secure data transmission and input of the S100B assay results on the hospital server for laboratory results. Furthermore, for non-equipped centers, Roche will deliver the instrument and provide on-site training to the operators.

16.2. Insurance

In accordance with regulatory provisions, Clermont-Ferrand Hospital, in its capacity as sponsor, has taken out civil liability insurance covering any damages resulting from the research with the Société Hospitalière d’Assurances Mutuelles (SHAM). The policy number is 147161.

It should be noted that non-observance of the legal conditions of the research (absence of Ethics Committee opinion, absence of ANSM authorization, non-consent of subjects, continuation of a suspended or prohibited study) shall render this coverage void.

17.Communication – Rules for publication

The data will only be disclosed after preliminary joint agreement of the investigator and the sponsor. The results will be the subject of communications and publications.

The study will be registered and declared on the *Clinical trials.gov*. internet site.

All publications of the results will mention the sources of funding for the study.

18. Feasibility of the study

The feasibility of the study is excellent based on the following criteria:

- The Medical Biochemistry and Pediatrics departments at Clermont-Ferrand Hospital, which are the principal investigators for this study, have extensive experience on the subject of “S100B and mTBI”. In fact, our previous work on this subject has been published in national and international scientific journals (Bouvier *et al.,* 2009, 2011, 2012, 2012b, 2013 and 2015).
- Recruitment potential: The recruitment potential at each study center will amply meet the accrual target, since the pediatric emergency departments see children with mTBI every day. Among 446 children enrolled in our first study (Bouvier *et al*., 2012), 241 met the inclusion criteria for this new study in 1 year and considering that Clermont-Ferrand is a medium size hospital. In addition, blood was drawn for the S100B determination on average 2 hours 17 minutes after the trauma, indicating that the requirement to obtain a blood sample within 3 hours after trauma is not a feasibility constraint for this study.
- Regarding the practical conduct of the study, the blood samples will follow the same circuit as all biological samples from the pediatric emergency department and will therefore be processed in a time period compatible with the proper functioning of such a department.
- A CRA at the Clermont-Ferrand Hospital and a CRT at each study center will enable regular monitoring of the advancement of the study in compliance with the protocol.
- Lastly, the pediatrician investigators in the study are motivated by their desire to validate the utility of a biomarker allowing to reduce the number of CCT-scans prescribed for their patients.
- As explained before, statistical analysis will be performed by Bruno Pereira (PhD in biostatistics), of the Clermont-Ferrand University Hospital Clinical Research and Innovation Delegation. Bruno Pereira is implied in several cluster randomized trials and gives his expertise about this subject for many institutes as INPES, INCa and WHO.

19. Comments and answers to previous examinations

In order to show the dynamic improvement of our project to CPP members, we present in this chapter comments and answers to previous examninations from our PHRC (Hospital Clinical Research Program) candidature.

Previous expert comments

Expertise 2 :

« Le point le plus important à discuter est celui de la pertinence de la question de recherche posée. Il est en effet important d’évaluer en quoi la mise à disposition d’un test sanguin S100B permettant d’exclure un risque de complications permet de réduire, en pratique, l’utilisation du scanner. Les choix des urgentistes, qui restent maitres de leur démarche diagnostique, pronostique et éventuellement thérapeutique, tiennent compte de facteurs difficiles à mesurer dans leur totalité,

voire même à recenser. Il importe donc de connaitre l'impact effectif de la mise à disposition d'un nouvel outil. C'est la question posée par ce projet.
Cependant, cette question n'est pertinente que si nous avons la certitude de la fiabilité de l'outil diagnostique (S100B) lorsque l'on fait le choix de l'utiliser. Pour entrer dans les détails, il est important de connaitre le nombre de faux négatifs. Plus précisément, il importe de connaitre la valeur prédictive négative du test ainsi que, et c'est le plus important, la borne inférieur de son intervalle de confiance qui donne une idée de la probabilité d'avoir une complication même si le test S100 est négatif.
Pour connaître ces paramètres, des études doivent être conduites chez un grand nombre de patients, essentiellement parce que la fréquence des complications est faible. Comme le souligne les auteurs, cela a été fait parmi plusieurs milliers de patients adultes. Malheureusement, rien de tel chez l'enfant puisque les études disponibles portent ensembles sur moins de 500 patients. Le nombre total d'enfants avec un scan positif est extrêmement faible (pour faire simple, le résultat du scan est utilisé comme proxi de la complication pour améliorer la précision des estimations). Il importe donc :
1/ de se poser la question de la confiance que l'on a dans l'estimation de la valeur prédictive négative du test. Cette question fondamentale n'est pas abordée dans le document.
2/ En fonction de la réponse au point 1), il faut décider si la priorité doit être à l'amélioration de la précision de la mesure de cette VPN ou si nous pouvons passez à des mesures plus pragmatiques comme celle de la baisse de l'utilisation du scan. »

Corresponding answers Expertise 2

Oui, le nombre d'études sur l'intérêt de la S100B dans la prise en charge du traumatisme crânien léger de l'enfant est faible. Cela pose effectivement le problème de l'intervalle de confiance autour des valeurs de sensibilité et de VPN rapportées, qui étaient déjà précisées dans le protocole soumis l'an dernier, contrairement aux commentaires de l'expert 2. Néanmoins, ces éléments seront mieux précisés dans la nouvelle version. Nous souhaitons passer directement à une grande étude randomisée interventionnelle pour l'amélioration des pratiques en pédiatrie. Nos arguments sont les suivants:
Les innombrables études observationnelles chez l'adulte ont toutes démontré la même chose. Nous utilisons nous mêmes ce biomarqueur en routine pour notre SAU adultes et retrouvons les données de la littérature. Nous choisissons la sécurité avec le critère d'inclusion d'un prélèvement dans les 3h après le TCL comme dans les études de Castellani et al. (2009) et Bouvier et al. (2012) où la sensibilité est de 100%. Dans d'autres études (Bechtel et al., 2009 ; Babcock et al., 2012), le délai maximal entre le TCL et la prise de sang est de 6h et la sensibilité est plus faible.
Les cut off assurent une bonne sensibilité et des spécificités d'environ 30%. Nous sommes donc bien sur des seuils de dépistage privilégiant la sensibilité.
Nous allons dans la prochaine version mettre en place un comité de surveillance de l'étude constitué entre autre d'une pédiatre, le Pr Gras Le Guen, spécialiste du TCL de l'enfant et qui met en place le dosage de la S100B en routine dans son service.
Comme suggéré par d'autres experts, pour minimiser le risque, nous allons introduire dans le protocole du bras intervention une surveillance hospitalière clinique de 6 heures post TCL.

Enfin, les enfants avec traumatisme crânien léger à haut risque de lésion intracrânienne ne seront pas inclus (Cf critères d'exclusion et figures 2 et 3).

Expertise 2 :

« La pertinence du choix des mesures secondaires doit être mieux étudiée. Par exemple la mesure de la dose de radiation, ou la présence d'une lésion intracrânienne : quelle différence est attendue entre les groupes ? N'y a t il pas un biais d'indication ? Une plus grande fréquence de + dans le groupe S100 signe t elle un succès (ceux qui ont un scan malgré une S100 négative avaient d'autres bonnes raison d'être scannés) ou un échec (la valeur prédictive de la S100 n'est pas de 100). Le choix des mesures secondaires doit être mieux explicité en discutant les hypothèses sousjacentes.
Sur ce point enfin, pourquoi ne pas essayer de connaitre les raisons qui ont conduit les cliniciens à faire un scan en dépit d'une s100 negative ?
Page 22 : Nevertheless, this study is intended to be pragmatic because in the "S100B management" arm, the pediatricians will still be able to not base their management on the S100B concentration. This aspect will be taken into account in the statistical considerations.
Il convient de dire comment cela sera fait. »

Corresponding answers Expertise 2

Les conseils de l'expert ont été suivis, une attention particulière sera portée aux raisons qui ont conduit les cliniciens à faire un scanner en dépit d'une s100b négative. Concernant le manque de précision des objectifs secondaires, il était déjà indiqué qu'une diminution de la dose de radiation était escomptée dans le bras s100b. Cet objectif a donc été conservé comme tel. Par contre, des précisions ont été apportées dans la description des critères de jugement secondaires concernant la présence d'une lésion intracrânienne : nombre de lésions rapporté au nombre de scanners réalisés. Nous présenterons dans la prochaine version deux schémas (un par bras) explicitant les algorithmes décisionnels de la SFP (Figures 2 et 3). Dans le bras interventionnel S100B, le dosage de S100B sera introduit après l'examen clinique et la décision de prescrire une TDM. D'une part, l'estimation des effectifs est majorée pour tenir de compte de ce fait. Concernant l'analyse statistique, une analyse descriptive détaillée sera réalisée pour les patients pour lesquels le médecin pédiatre ne suivra pas les recommandations (résultats s100b). Par ailleurs, comme cela était précisé dans le protocole, une analyse multivariée prenant en compte cet élément sera considérée.

Expertise 3 :

« Il s'agit d'un excellent projet d'étude clinique en pédiatrie, dont la méthodologie est adaptée et dont le rationnel, très bien explicité et correspond à la pratique des services recevant ces enfants, et où les recommandations SFMU sont maintenant largement appliquées. Ma seule réserve concerne la faisabilité de l'étude, même si l'expérience de l'équipe lors de leur dernière étude semble plutôt en accord avec ce qui est ici prévu. En effet, parmi tous les enfants admis pour TC aux urgences, nombre se présentent plus de 3 heures après l'accident, ce qui sera un facteur limitant de recrutement. Dans ce contexte, l'admission, l'installation, l'examen clinique, l'information de l'enfant et de sa famille, le recueil de consentement puis sa randomisation (même en ligne) constituent un « contre la montre » avant la ponction veineuse (lorsque le binome

d'infirmières/AP sera disponible …) qui risque d'être difficile à tenir. Dans ce contexte, je proposerais volontiers d'élargir le nombre des centres participants ou de partir sur une durée d'étude plus longue. Un design en steppedwedge permettrait également une randomisation par centre et non par enfant, sans doute moins chronophage dans ce contexte ?
Ce projet constitue un tel espoir d'amélioration des pratiques qu'il serait vraiment dommage qu'il soit mis en difficulté sur un recrutement insuffisant dans ce contexte difficile que constitue la recherche clinique aux urgences pédiatriques. »

Corresponding answers
Comme conseillé par l'expert, un design en steppedwedge sera adopté dans la version de cette année pour faciliter l'inclusion des enfants dans les centres investigateurs, ce qui implique automatiquement une inflation du nombre de sujets nécessaires afin de prendre au mieux en compte la variabilité inter et intra centre, mesurée par le coefficient de corrélation intraclasse.
Comme évoqué par l'expert, le chapitre faisabilité de notre précédente version montre que nous pourrons inclure suffisamment d'enfants. Nous allons également augmenter le nombre de centres pour nous en assurer.

Expertise 4 :
« Hypothèse relevante cliniquement mais le protocole est basé sur une seule étude qui détermine une VPN de 100% pour dédouaner une lésion avec des seuils variables selon l'âge.
Il me semble nécessaire de minimiser le risque en introduisant dans le protocole du bras intervention une surveillance hospitalière clinique de 6 heures post TC. On ne fait sortir aucun enfant inclus avant la 6eme heure. »

Corresponding answers Expertise 4 :

Nous allons suivre les conseils de l'auteur.

Expertise 5 :
"This is an ambitious and well designed study which will have a massive impact on head injury management in children thoughtout the world. If the ethical board approves the study (which to me seems unlikely given the present evidence), I think this study will be very successful and will be publishable in a high-impact journal. It will have direct impact on patient care and the possibility of saving health-economic resources."

Corresponding answers Expertise 5 :
Merci à l'expert pour son encouragement. Le succès de l'étude et la publication de ses résultats sont très souhaités par les investigateurs qui ne désespèrent pas de mettre en place cette étude. Pour lever les craintes éthiques de l'expert, nous allons mettre en place un comité de surveillance de l'étude et garder tous les enfants aux urgences pour surveillance au moins 6 heures. Enfin, les enfants avec traumatisme crânien léger à haut risque de lésion intracrânienne ne seront pas inclus (Cf critères d'exclusion et figures 2 et 3).

20.Bibliography

Adams G, Gulliford MC, Ukoumunne OC, Eldridge S, Chinn S, Campbell MJ. Patterns of intra-cluster correlation from primary care research to inform study design and analysis. J Clin Epidemiol. 2004 Aug;57(8):785-94.

American Academy of Pediatrics. The management of mild closed head injury in children. Committee on Quality Improvement, Commission on Clinical Policies and Research, American Academy of Family Physicians. Pediatrics. 1999 Dec;104(6):1407-15.

Anderson RE, Hansson LO, Nilsson O, Liska J, Settergren G, Vaage J. Increase in serum S100A1-B and S100BB during cardiac surgery arises from extracerebral sources. Ann Thorac Surg. 2001 ; 71 :1512-7.

Beaudeux JL, Dequen L, Foglietti MJ. La protéine S-100beta : un nouveau marqueur biologique de pathologie cérébrale. Ann Biol Clin. 1999;57(3):261-72.

Beaudeux JL, Roche S, Puyssabet L, Foglietti MJ. Physiologie de la protéine S100B et apport de son dosage dans les pathologies neurologiques. Immunoanal Biol Spéc. 2001;16:14-148.

Beaudeux JL, Soler C, Foglietti MJ. Physiopathologie de la protéine S-100B : intérêt de son dosage en biologie clinique. Immunoanal Bio Spec. 2002;17: 280-6.

Biberthaler P, Linsenmeier U, Pfeifer KJ, Kroetz M, Mussack T, Kanz KG et al. Serum S-100B concentration provides additional information for the indication of computed tomography in patients after mild head injury: a prospective multicenter study. Shock. 2006 May;25(5):446-53.

Bouvier D, Duret T, Rouzaire P, Jabaudon M, Rouzaire M, Nourrisson C et al. Preanalytical, analytical, gestational and pediatric aspects of the S100B immuno-assays. Clin Chem Lab Med. 2015 Oct 17.

Bouvier D. [Interest of S100B protein blood level determination in severe or moderate head injury]. Ann Biol Clin (Paris). 2013 Mar-Apr;71(2):145-50.

Bouvier D, Castellani C, Fournier M, Dauphin JB, Ughetto S, Breton M et al. Reference ranges for serum S100B protein during the first three years of life. Clin Biochem. 2011 Jul;44(10-11):927-9.

Bouvier D, Eisenmann N, Gillart T, Bonneau J, Guelon D, Schoeffler P et al. [Jugular venous and arterial concentrations of serum S100B protein in patients with severe head injury]. Ann Biol Clin (Paris). 2012b May-Jun;70(3):269-75.

Bouvier D, Fournier M, Dauphin JB, Amat F, Ughetto S, Labbé A et al. Serum S100B determination in the management of pediatric mild traumatic brain injury. Clin Chem. 2012 Jul;58(7):1116-22.

Bouvier D, Oddoze C, Ben Haim D, Moustafa F, Legrand A, Alazia M et al. [Interest of S100B protein blood level determination for the management of patients with mild head trauma]. Ann Biol Clin (Paris). 2009 Jul-Aug;67(4):425-31.

Calcagnile O, Undén L, Undén J. Clinical validation of S100B use in management of mild head injury. BMC Emerg Med. 2012 Oct 27;12(1):13.

Castellani C, Bimbashi P, Ruttenstock E, Sacherer P, Stojakovic T, Weinberg AM. Neuroprotein s-100B -- a useful parameter in paediatric patients with mild traumatic brain injury? Acta Paediatr. 2009;98(10):1607-12.

Eldridge SM, Ashby D, Kerry S. Sample size for cluster randomized trials: effect of coefficient of variation of cluster size and analysis method. Int J Epidemiol. 2006 Oct;35(5):1292-300.

Homer CJ, Kleinman L. Technical report: mild head injury in children. Pediatrics. 1999 Dec;104(6):e78.

Hung R, Carroll LJ, Cancelliere C, Côté P, Rumney P, Keightley M et al. Systematic review of the clinical course, natural history, and prognosis for pediatric mild traumatic brain injury: results of the International Collaboration on Mild Traumatic Brain Injury Prognosis. Arch Phys Med Rehabil. 2014 Mar;95(3 Suppl):S174-91.

Hussey MA, Hughes JP. Design and analysis of stepped wedge cluster randomized trials. Contemp Clin Trials. 2007 Feb;28(2):182-91.

Jehlé E, Honnart D, Grasleguen C, Bouget J, Dejoux C, Lestavel P et al. Traumatisme crânien léger (score de Glasgow de 13 à 15) : triage, évaluation, examens complémentaires et prise en charge précoce chez le nouveau-né, l'enfant et l'adulte. Ann Fr Med Urgence. 2012 ; 2:199-214.

Kuppermann N, Holmes JF, Dayan PS, Hoyle JD, Atabaki SM, Holubkov R et al. Identification of children at very low risk of clinically-important brain injuries after head trauma: a prospective cohort study. Lancet. 2009;374(9696):1160-70.

Laporte C, Vaillant-Roussel H, Pereira B, Blanc O, Tanguy G, Frappé P et al. CANABIC: CANnabis and Adolescents: effect of a Brief Intervention on their Consumption--study protocol for a randomized controlled trial. Trials. 2014 Jan 30;15:40.

Lorton F, Levieux K, Vrignaud B, Hamel O, Jehlé E, Hamel A et al. Actualisation des recommandations pour la prise en charge du traumatisme crânien léger chez l'enfant. 2014 ; 21:790-796.

Machin D. On the evolution of statistical methods as applied to clinical trials. J Intern Med. 2004 May;255(5):521-8.

Mathews JD, Forsythe AV, Brady Z, Butler MW, Goergen SK, Byrnes GB et al. Cancer risk in 680,000 people exposed to computed tomography scans in childhood or adolescence: data linkage study of 11 million Australians. BMJ. 2013 May 21;346:f2360.

Manzano S, Holzinger IB, Kellenberger CJ, Lacroix L, Klima-Lange D, Hersberger M, La Scala G, Altermatt S, Staubli G. Diagnostic performance of S100B protein serum measurement in detecting intracranial injury in children with mild head trauma. Emerg Med J. 2015 (in press).

Mdege ND, Man MS, Taylor Nee Brown CA, Torgerson DJ. Systematic review of stepped wedge cluster randomized trials shows that design is particularly used to evaluate interventions during routine implementation. J Clin Epidemiol. 2011 Sep;64(9):936-48.

Miglioretti DL, Johnson E, Williams A, Greenlee RT, Weinmann S, Solberg LI et al. The use of computed tomography in pediatrics and the associated radiation exposure and estimated cancer risk. JAMA Pediatr. 2013 Aug 1;167(8):700-7.

Mourgues C, Gerbaud L, Leger S, Auclair C, Peyrol F, Blanquet M et al. Positive and cost-effectiveness effect of spa therapy on the resumption of occupational and non-occupational activities in women in breast cancer remission: a French multicentre randomised controlled trial. Eur J Oncol Nurs. 2014 Oct;18(5):505-11.

Pearce MS, Salotti JA, Little MP, McHugh K, Lee C, Kim KP et al. Radiation exposure from CCT scans in childhood and subsequent risk of leukaemia and brain tumours: a retrospective cohort study. Lancet. 2012;380(9840):499-505.

Sanchez-Peña P, Pereira AR, Sourour NA, Biondi A, Lejean L, Colonne C et al. S100B as an additional prognostic marker in subarachnoid aneurysmal hemorrhage. Crit Care Med. 2008;36(8):2267-73.

Sancho-Garnier H, Pereira B, Césarini P. A cluster randomized trial to evaluate a health education programme "Living with Sun at School". Int J Environ Res Public Health. 2012 Jul;9(7):2345-61.

Simon-Pimmel J, Lorton F, Guiziou N, Levieux K, Vrignaud B, Masson D et al. Serum S100β Neuroprotein Reduces Use of Cranial Computed Tomography in Children After Minor Head Trauma. Shock. 2015 Nov;44(5):410-6.

Undén J, Romner B. Can low serum levels of S100B predict normal CT findings after mild head injury in adults?: an evidence-based review and meta-analysis. J Head Trauma Rehabil. 2010 Jul-Aug;25(4):228-40.

Vaillant-Roussel H, Laporte C, Pereira B, Tanguy G, Cassagnes J, Ruivard M et al. Patient education in chronic heart failure in primary care (ETIC) and its impact on patient quality of life: design of a cluster randomised trial. BMC Fam Pract. 2014 Dec 24;15:208.

21. List of appendix

Appendix 1: Fiches d’information
Appendix 2: Formulaires de consentement
Appendix 3: Score de Glasgow en fonction de l’âge
Appendix 4: Fiche d’évolution clinique
Appendix 5: Notice du kit de dosage de la protéine S100B (Roche)
Appendix 6: Fiche de déclaration d’événement indésirable grave

APPENDIX 1A
FORMULAIRE D'INFORMATION
POUR LES PARENTS OU TITULAIRES DE L'AUTORITE PARENTALE

Etude interventionnelle évaluant l'apport du dosage sanguin de la protéine S100B dans la prise en charge du traumatisme crânien léger de l'enfant
PROS100B

- **Promoteur**
  CHU de Clermont-Ferrand
  58 Rue de Montalembert, 63003 Clermont-Ferrand Cedex 1

- **Investigateur coordonnateur : Damien Bouvier**
  Service de Biochimie Médicale
  CHU de Clermont-Ferrand

Madame, Monsieur,

Nous vous proposons de faire participer votre enfant à une recherche dont l'intitulé est mentionné ci-dessus, qui va se dérouler dans le Service des Urgences pédiatriques de 9 centres hospitaliers en France.

L'objectif de l'étude est d'améliorer la stratégie de prise en charge des enfants traumatisés crâniens.

Par le dosage sanguin d'une protéine (protéine S100B), nous pouvons écourter en cas de résultat normal, le temps d'hospitalisation, et diminuer le nombre d'examens complémentaires chez l'enfant.

La protéine S100B est synthétisée essentiellement dans des cellules cérébrales et est libérée dans le sang en cas de traumatisme crânien.

L'étude se déroulera en 2 étapes :

- Votre enfant va bénéficier de manière aléatoire (tirage au sort) :
  - d'une prise en charge classique selon les recommandations de la Société Française de Médecine d'Urgence et du Groupe francophone de réanimation et urgences pédiatriques

  ou

  - d'une prise en charge avec dosage sanguin de la protéine S100B. Dans ce dernier cas, votre enfant aura une prise de sang réalisée par une infirmière du service. Le dosage de la protéine S100B nécessite le prélèvement d'un seul tube de sang, soit par prise de sang classique (environ 1 ml de sang), soit par micro-méthode pour les nourrissons de moins de 6 mois (prélèvement au bout du doigt ou éventuellement au talon).
    - En cas de résultat normal, votre enfant pourra écourter son séjour aux urgences avec des consignes classiques de surveillance à domicile.
    - En cas de résultat anormal, la prise en charge classique sera réalisée. A la fin de l'étude, les échantillons seront détruits.

- Ensuite une personne du corps médical vous appellera à domicile à 48h et 3 semaines après le traumatisme de votre enfant pour vous poser quelques questions concernant son état de santé.

La participation de votre enfant à cette étude peut faire progresser les moyens thérapeutiques mis à la disposition du corps médical.

La prise de sang sera réalisée dans des règles strictes d'asepsie. Les risques encourus par votre enfant sont ceux qui peuvent être rencontrés lors d'une prise de sang (malaise, ecchymose au point de ponction, douleur locale…).

Votre participation à cette recherche biomédicale n'engendrera pour vous aucun frais supplémentaire par rapport à ceux que vous auriez dans la prise en charge habituelle de votre enfant.

Toutefois, pour pouvoir participer à cette recherche vous devez être affiliés ou bénéficier d'un régime de sécurité sociale.

Le CHU de Clermont Ferrand, qui organise cette recherche biomédicale en qualité de promoteur, a contracté une assurance conformément aux dispositions législatives, garantissant sa responsabilité civile et celle de tout intervenant auprès de la Société Hospitalière d'Assurances Mutuelles (SHAM, contrat n°147161). Dans le cas où l'état de santé de votre enfant serait altéré du fait de sa participation à l'étude, conformément à la loi de Santé Publique n°2004-806 du 9 août 2004, vous seriez en droit de recevoir des dédommagements dans le cadre de ce contrat d'assurance spécifique.

Cette recherche a reçu l'avis favorable du Comité de Protection des Personnes Sud Est VI le …./…./…. ainsi que l'autorisation préalable de l'autorité compétente de santé.

Il est possible que cette recherche soit interrompue, si les circonstances le nécessitent, par le promoteur ou à la demande de l'autorité de santé.

Les informations relatives à l'étude recueillies par l'investigateur sont traitées confidentiellement. En accord avec la Loi Informatique et Liberté, le nom des sujets est systématiquement remplacé par un N° de code dont la correspondance est connue des seuls investigateurs.

Vous acceptez :

- que ces données puissent faire l'objet d'un traitement informatisé anonyme.

- leur consultation par des collaborateurs participant à la recherche, désignés par le promoteur et éventuellement par un représentant des autorités de santé. Conformément aux dispositions de loi relative à l'informatique aux fichiers et aux libertés, vous disposez également d'un droit d'accès et de rectification. Vous disposez également d'un droit d'opposition à la transmission des données couvertes par le secret professionnel susceptible d'être utilisées dans le cadre de cette recherche et d'être traitées.

Vous pouvez également accéder directement ou par l'intermédiaire d'un médecin de votre choix à l'ensemble des données médicales de votre enfant en application des dispositions de l'article L. 1111-7 du code de la santé publique. Ces droits s'exercent auprès du Docteur …………. qui suit votre enfant dans le cadre de la recherche et qui connaît son identité.

Vous êtes libre d'accepter ou de refuser que votre enfant participe à cette recherche sans avoir à vous justifier. De plus vous pouvez exercer à tout moment votre droit de retrait de cette recherche sans avoir à donner de raison. Le fait de ne plus participer à cette recherche ne modifiera pas la qualité des soins qui lui seront prodigués. Vous pouvez demander à tout moment des explications complémentaires sur l'étude à l'équipe soignante.

Par ailleurs, vous pourrez être tenu informé des résultats globaux de cette recherche à la fin de l'étude.

Lorsque vous aurez lu cette note d'information et obtenu les réponses aux questions que vous vous posez en interrogeant le médecin investigateur, il vous sera proposé, si vous êtes en accord, de donner votre consentement écrit en signant le document préparé à cet effet après avoir eu un moment de réflexion en dehors de la présence d'un membre du CHU.

Date : …../…./…..

Signature des parents ou des titulaires de l'autorité parentale :
(Précédée de la mention « Lu et compris »)

Paraphe de l'investigateur

APPENDIX 1B
FORMULAIRE D'INFORMATION
POUR LES ENFANTS DE 8 A 13 ANS

Etude interventionnelle évaluant l'apport du dosage sanguin de la protéine S100B dans la prise en charge du traumatisme crânien léger de l'enfant
PROS100B

Tu viens de te faire mal à la tête et tu as dû venir à l'hôpital pour qu'on te soigne. Nous te proposons de participer à une étude.

Pour cette étude, on va peut-être te faire une prise de sang. Elle permet de prendre un peu de ton sang pour le faire analyser dans un laboratoire. Les résultats pourront aider les médecins à évaluer ton traumatisme à la tête et donc à bien te soigner.

- Le but est de faire une prise de sang pour mieux surveiller les enfants qui se font mal à la tête,

- Les résultats de ta prise de sang seront utilisés pour la recherche médicale sans que ton nom soit écrit.

- Tu pourras poser les questions que tu veux au docteur.

TU PEUX DIRE NON SI TU NE VEUX PAS QU'ON FASSE CETTE RECHERCHE.

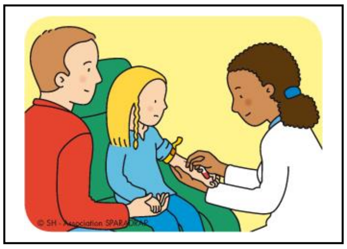
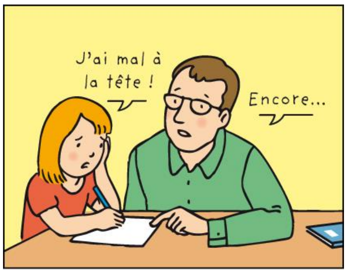

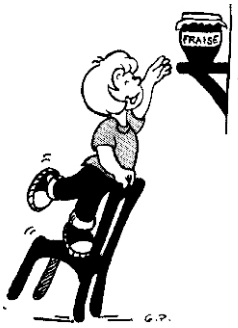

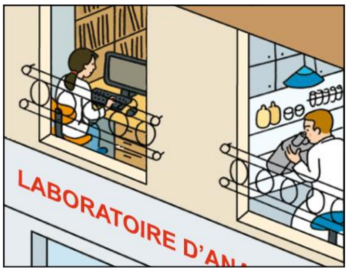

APPENDIX 1C
FORMULAIRE D'INFORMATION POUR LES ADOLESCENTS

Etude interventionnelle évaluant l'apport du dosage sanguin de la protéine S100B dans la prise en charge du traumatisme crânien léger de l'enfant
PROS100B

- **Promoteur**
  CHU de Clermont-Ferrand
  58 Rue de Montalembert, 63003 Clermont-Ferrand Cedex 1

- **Investigateur coordonnateur : Damien Bouvier**
  Service de Biochimie Médicale
  CHU de Clermont-Ferrand

Mademoiselle, Monsieur,

Nous te proposons de participer à une recherche dont l'intitulé est mentionné ci-dessus, qui est organisée par le Service des Urgences pédiatriques du CHU de Clermont-Ferrand.

L'objectif de l'étude est d'améliorer la stratégie de prise en charge des enfants traumatisés crâniens.

Par le dosage sanguin d'une protéine (protéine S100B), nous pourrons écourter en cas de résultat normal, le temps d'hospitalisation, et diminuer le nombre d'examens complémentaires.

La protéine S100B est synthétisée essentiellement dans des cellules cérébrales et est libérée dans le sang en cas de traumatisme crânien.

L'étude se déroule de la manière suivante :

- Tu vas bénéficier de manière aléatoire (tirage au sort) :
  - d'une prise en charge classique selon les recommandations de la Société Française de Médecine d'Urgence et du Groupe francophone de réanimation et urgences pédiatriques

  ou

  - d'une prise en charge avec dosage sanguin de la protéine S100B. Dans ce dernier cas, tu auras une prise de sang réalisée par une infirmière du service. Le dosage de la protéine S100B nécessite le prélèvement d'un seul tube de sang par prise de sang classique (environ 1 ml de sang).
    - En cas de résultat normal, tu pourras écourter ton séjour aux urgences avec des consignes classiques de surveillance à domicile.
    - En cas de résultat anormal, la prise en charge classique sera réalisée. A la fin de l'étude, les échantillons de sang seront détruits.

- Ensuite, une personne du corps médical te rappellera (toi ou tes parents) à domicile 48h et 3 semaines après ton traumatisme pour vous poser quelques questions concernant ton état de santé.

Ta participation à cette étude peut aider les médecins à mieux soigner les patients.

La prise de sang sera réalisée dans des règles strictes d'asepsie. Les risques que tu encoures sont ceux qui peuvent être rencontrés lors d'une prise de sang (malaise, ecchymose au point de ponction, douleur locale…).

Ta participation à cette recherche biomédicale n'engendrera pour tes parents aucun frais supplémentaire par rapport à ceux que vous auriez en cas de prise en charge habituelle.

Toutefois, pour pouvoir participer à cette recherche tes parents doivent être affiliées ou bénéficier d'un régime de sécurité sociale.

Le CHU de Clermont Ferrand, qui organise cette recherche biomédicale en qualité de promoteur, a contracté une assurance conformément aux dispositions législatives, garantissant sa responsabilité civile et celle de tout intervenant auprès de la Société Hospitalière d'Assurances Mutuelles (SHAM, contrat n°147161). Dans le cas où ton état de santé serait altéré du fait de ta participation à l'étude, conformément à la loi de Santé Publique n°2004-806 du 9 août 2004, vous seriez en droit de recevoir des dédommagements dans le cadre de ce contrat d'assurance spécifique.

Cette recherche a reçu l'avis favorable du Comité de Protection des Personnes Sud Est VI le …./…./…. ainsi que l'autorisation préalable de l'autorité compétente de santé.

Les informations relatives à l'étude recueillies par l'investigateur sont traitées confidentiellement (traitement informatisé anonyme).

Conformément aux dispositions de loi relative à l'informatique aux fichiers et aux libertés, tu disposes également d'un droit d'accès et de rectification. Tu disposes également d'un droit d'opposition à la transmission des données couvertes par le secret professionnel susceptible d'être utilisées dans le cadre de cette recherche et d'être traitées.

Tu peux également accéder directement ou par l'intermédiaire d'un médecin de ton choix à l'ensemble de tes données médicales. Ces droits s'exercent auprès du Docteur …………………… qui te suit dans le cadre de la recherche et qui connaît ton identité.

Tu es libre d'accepter ou de refuser de participer à cette recherche sans avoir à te justifier. De plus tu peux exercer à tout moment ton droit de retrait de cette recherche sans avoir à donner de raison. Le fait de ne plus participer à cette recherche ne modifiera pas la qualité des soins qui te seront prodigués. Tu peux demander à tout moment des explications complémentaires sur l'étude à l'équipe soignante.

Par ailleurs, tu pourras être tenu informé des résultats globaux de cette recherche à la fin de l'étude.

Lorsque tu auras lu cette note d'information et obtenu les réponses aux questions que tu te poses en interrogeant le médecin investigateur, il te sera proposé, si tu es d'accord, de donner ton consentement écrit en signant le document préparé à cet effet, après avoir eu un moment de réflexion en dehors de la présence d'un membre du CHU.

Date : …../…./…..

Signature du patient **Paraphe de l'investigateur**
(Précédée de la mention « Lu et compris »)

APPENDIX 2A
FORMULAIRE DE CONSENTEMENT DE PARTICIPATION A UNE RECHERCHE BIOMEDICALE
POUR LES PARENTS OU TITULAIRES DE L'AUTORITE PARENTALE

Etude interventionnelle évaluant l'apport du dosage sanguin de la protéine S100B dans la prise en charge du traumatisme crânien léger de l'enfant
PROS100B

Investigateur coordonnateur : **Damien Bouvier**
Service de Biochimie Médicale – CHU de Clermont-Ferrand

Je soussigné(e)
Mme, M (*nom, prénom*)…………………………………………
Né(e) le ………………………………...
Demeurant………………………………………………………………………………
Téléphone fixe………………………… Téléphone portable…………………………………
Déclare :
- que le Docteur (*nom, prénom,* ~~*téléphone*~~)……………………………………… m'a proposé de faire participer mon enfant à l'étude sus nommée,
- qu'il m'a expliqué en détail le protocole,
- qu'il m'a notamment fait connaître :

- l'objectif, la méthode et la durée de l'étude
- les contraintes et les risques potentiels encourus
- le rappel téléphonique à 48h et à 3 semaines
- mon droit de refuser de participer et en cas de désaccord de retirer mon consentement à tout moment
- mon obligation d'inscription à un régime de sécurité sociale
- que, si je le souhaite, à son terme, je serai informé par le médecin investigateur des résultats globaux de la recherche
- que le Comité de Protection des Personnes Sud Est VI a émis un avis favorable en date du ……….
- que dans le cadre de cette étude le promoteur, le CHU de Clermont-Ferrand, a souscrit à une assurance couvrant cette recherche.

Les informations relatives à l'étude recueillies par l'investigateur sont traitées confidentiellement.

J'accepte :
- que ces données puissent faire l'objet d'un traitement informatisé anonyme. J'ai bien noté que le droit d'accès prévu par la loi du 6 janvier 1978 relative à l'informatique, aux fichiers et aux libertés (art. 39) s'exerce à tout moment auprès du médecin qui me suit dans le cadre de la recherche et qui connaît mon identité. Je pourrai exercer mon droit de rectification et d'opposition auprès de ce même médecin, qui contactera le promoteur de la recherche.

Je pourrai à tout moment demander des informations complémentaires au Dr ……………… en appelant le ………………………

Après avoir discuté librement et obtenu réponse à toutes mes questions, j'accepte librement et volontairement de participer à cette recherche biomédicale dans les conditions précisées dans le formulaire d'information et de consentement.

**Nom et prénom du patient :**<br>**…………………………………………………**<br><br>**Date :……./……./…….**<br>**Signature des parents ou des titulaires de l'autorité parentale :**	**Nom de l'investigateur :**<br>**…………………………………………………**<br><br>**Date :……./……./…….**<br>**Signature :**

Précédée de la mention « Lu et compris » :

Ce document est à réaliser en 2 exemplaires originaux, dont le premier doit être gardé 15 ans par l'investigateur, un autre remis à la personne donnant son consentement.

APPENDIX 2B
FORMULAIRE DE CONSENTEMENT DE PARTICIPATION A UNE RECHERCHE BIOMEDICALE
POUR L'ENFANT DE 8 A 13 ANS

Etude interventionnelle évaluant l'apport du dosage sanguin de la protéine S100B dans la prise en charge du traumatisme crânien léger de l'enfant
PROS100B

Investigateur coordonnateur : **Damien Bouvier**
Service de Biochimie Médicale – CHU de Clermont-Ferrand

De M. ou Mlle ....(Nom, Prénom)

Né(e) le ....

Le Docteur .... m'a proposé de participer à l'étude organisée par le CHU de Clermont-Ferrand. Il m'a précisé que je suis libre d'accepter ou de refuser ; ceci ne changera pas nos relations. Le but, les risques et la durée de cette étude et la manière dont elle va se passer m'ont été clairement expliqués. J'ai bien compris toutes les informations qui m'ont été fournies.

Je pourrai à tout moment demander une information complémentaire au médecin, en faisant appeler mes parents au ……………………………

Si je le souhaite, je pourrai quand je veux arrêter ma participation à cette étude. J'en informerai alors immédiatement le Docteur ……………………

Il m'a été signalé que les résultats de la prise de sang seront utilisés sans que mon identité ne soit révélée.

Je sais que cette étude a reçu l'approbation du Comité de Protection des Personnes (CPP) Sud Est VI lors de sa séance du…………...

J'ACCEPTE DE PARTICIPER A CETTE RECHERCHE DANS LES CONDITIONS PRECISEES DANS LE DOCUMENT D'INFORMATION QUI M'A ÉTÉ REMIS AVEC CE FORMULAIRE ET QUE J'AI LU AVEC ATTENTION.

Nom et prénom du patient :
…………………………………………………

Date :……./……./…….
Signature
Précédée de la mention « Lu et compris » :

Nom de l'investigateur :
…………………………………………………

Date :……./……./…….
Signature :

Ce document est à réaliser en 2 exemplaires originaux, dont le premier doit être gardé 15 ans par l'investigateur, un autre remis à la personne donnant son consentement.

APPENDIX 2C
FORMULAIRE DE CONSENTEMENT DE PARTICIPATION A UNE RECHERCHE BIOMEDICALE POUR L'ADOLESCENT

Etude interventionnelle évaluant l'apport du dosage sanguin de la protéine S100B dans la prise en charge du traumatisme crânien léger de l'enfant
PROS100B

Investigateur coordonnateur : **Damien Bouvier**
Service de Biochimie Médicale – CHU de Clermont-Ferrand

Je soussigné(e)
Mme, M (*nom, prénom*)…………………………………………
Né(e) le ……………….………………………………………………….
Demeurant………………………………………………………………………………
Déclare :
- que le Docteur (*nom, prénom,* ~~*téléphone*~~) ……………….……………………………… m'a proposé de participer à l'étude sus nommée,
- qu'il m'a expliqué en détail le protocole,
- qu'il m'a notamment fait connaître :
  - l'objectif, la méthode et la durée de l'étude
  - les contraintes et les risques potentiels encourus
  - mon droit de refuser de participer et en cas de désaccord de retirer mon consentement à tout moment
  - l'obligation de mes parents d'inscription à un régime de sécurité sociale
  - que, si je le souhaite, à son terme, je serai informé par le médecin investigateur des résultats globaux de la recherche
  - que le Comité de Protection des Personnes Sud Est VI a émis un avis favorable en date du ……………..
  - que dans le cadre de cette étude le promoteur, le CHU de Clermont-Ferrand, a souscrit à une assurance couvrant cette recherche.

Les informations relatives à l'étude recueillies par l'investigateur sont traitées confidentiellement.

J'accepte :
- que ces données puissent faire l'objet d'un traitement informatisé anonyme. J'ai bien noté que le droit d'accès prévu par la loi du 6 janvier 1978 relative à l'informatique, aux fichiers et aux libertés (art. 39) s'exerce à tout moment auprès du médecin qui me suit dans le cadre de la recherche et qui connaît mon identité. Je pourrai exercer mon droit de rectification et d'opposition auprès de ce même médecin, qui contactera le promoteur de la recherche.

J'ACCEPTE DE PARTICIPER A CETTE RECHERCHE DANS LES CONDITIONS PRECISEES DANS LE DOCUMENT D'INFORMATION QUI M'A ÉTÉ REMIS AVEC CE FORMULAIRE ET QUE J'AI LU AVEC ATTENTION

Je pourrai à tout moment demander des informations complémentaires au Dr …………….. en appelant le ………………………

Nom et prénom du patient :
…………………………………………………

Date :……./……./…….
Signature
Précédée de la mention « Lu et compris » :

Nom de l'investigateur :
…………………………………………………

Date :……./……./…….
Signature :

Ce document est à réaliser en 2 exemplaires originaux, dont le premier doit être gardé 15 ans par l'investigateur, un autre remis à la personne donnant son consentement.

APPENDIX 3
Score de Glasgow en fonction de l'âge
Recommandations de la SFMU
Jehlé *et al.,* 2012

Echelle de Glasgow standard (> 5 ans)	Echelle de Glasgow de 2 à 5 ans	Echelle de Glasgow de 0 à 2 ans
Ouverture des yeux :<br>4 - spontanément<br>3 - aux stimuli verbaux<br>2 - aux stimuli douloureux<br>1 - aucune réponse	Ouverture des yeux :<br>4 - spontanément<br>3 - aux stimuli verbaux<br>2 - aux stimuli douloureux<br>1 - aucune réponse	Ouverture des yeux :<br>4 - spontanément<br>3 - aux stimuli verbaux<br>2 - aux stimuli douloureux<br>1 - aucune réponse
Réponse verbale :<br>5 - est orienté et parle<br>4 - est désorienté et parle<br>3 - paroles inappropriées<br>2 - sons incompréhensibles<br>1 - aucune réponse	Réponse verbale :<br>5 - mots appropriés, sourit, fixe, suit du regard<br>4 - mots appropriés, pleure, est consolable<br>3 - hurle, est inconsolable<br>2 - gémit aux stimuli douloureux<br>1 - aucune réponse	Réponse verbale :<br>5 - agit normalement<br>4 - pleure<br>3 - hurlements inappropriés<br>2 - gémissements (grunting)<br>1 - aucune réponse
Réponse motrice :<br>6 - répond aux demandes<br>5 - localise la douleur<br>4 - se retire à la douleur<br>3 - flexion à la douleur (décortication)<br>2 - extension à la douleur (décérébration)<br>1 - aucune réponse	Réponse motrice :<br>6 - répond aux demandes<br>5 - localise la douleur<br>4 - se retire à la douleur<br>3 - flexion à la douleur (décortication)<br>2 - extension à la douleur (décérébration)<br>1 - aucune réponse	Réponse motrice :<br>6 - mouvements spontanés intentionnels<br>5 - se retire au toucher<br>4 - se retire à la douleur<br>3 - flexion à la douleur (décortication)<br>2 - extension à la douleur (décérébration)<br>1 - aucune réponse

APPENDIX 4

Fiche d'évolution clinique

Vous avez amené votre enfant aux urgences pédiatriques, suite à un traumatisme crânien. Nous vous rappelons, pour prendre de ses nouvelles.

	oui	non
A-t-il vomi depuis son retour à domicile ? Combien de fois ?		
(Pour les enfants de plus de 4 ans.) Se plaint-il de maux de tête ?		
Avez-vous remarqué un problème pour bouger un bras, une jambe ?		
A-t-il convulsé depuis ?		
A-t-il un changement dans sa mimique ? (paralysie faciale)		
Pour vous, son état est-il revenu à l'état antérieur ?		
Si non, pouvez-vous m'indiquer ce que vous avez remarqué ?	……………………	

Pour les dossiers consultés :

	oui	non
A-t-il vomit ? Combien de fois ?		
(Pour les enfants de plus de 4 ans.) Se plaint-il de maux tête ?		
A-t-il un déficit moteur ?		
A-t-il convulsé ?		
A-t-il une paralysie faciale ?		
Le réflexe photomoteur est-il présent ?		
L'enfant a-t-il été transféré dans un service de neurochirurgie ou de réanimation ?	……………………	
L'enfant a-t-il eu un scanner?		
Et si oui quel est le résultat ?	……………………	

APPENDIX 5
Notice du kit de dosage de la protéine S100B (Roche)

12177293001V5

S100
S100

cobas®

03175243 190 **100 tests**

• Réactifs utilisables sur les analyseurs suivants :

Elecsys 1010	Elecsys 2010	MODULAR ANALYTICS E170	**cobas e 411**	**cobas e 601**
•	•	•	•	•

Français

Remarque
La concentration en protéine S100 d'un échantillon de patient peut varier selon le test pratiqué. Le compte rendu du laboratoire doit donc toujours préciser la méthode de dosage de S100 utilisée. Les taux de S100 d'un patient obtenus à partir de différentes méthodes ne peuvent être comparés, ceci pouvant conduire à des erreurs d'interprétation médicale. En cas de changement de méthode au cours du suivi thérapeutique, les taux de S100 doivent être confirmés pendant une période transitoire en effectuant des dosages en parallèle par les deux méthodes.

Domaine d'utilisation
Test immunologique pour la détermination quantitative *in vitro* de la protéine S100 (S100 A1B et S100 BB) dans le sérum humain.
Le test Elecsys S100 peut être utilisé
- comme aide au suivi de patients atteints de mélanome malin (le test Elecsys S100 n'est pas approprié pour le diagnostic de mélanome malin).
- comme aide pour l'évaluation de souffrances cérébrales potentielles, en association avec les données cliniques et des techniques d'imagerie.

Ce test par électrochimiluminescence « ECLIA » s'utilise sur les analyseurs Elecsys et **cobas e**.

Caractéristiques
La protéine S100 est une petite protéine dimérique d'un poids moléculaire d'env. 10,5 kD. Elle appartient à la famille multigénique des protéines liant le calcium.[1,2]
Les protéines S100A1 (α) et S100B (β) ont été les premières décrites et isolées, à l'origine, par Moore, comme un mélange non fractionné[3] de cerveau bovin, et nommées S100 en raison de leur solubilité dans une solution de 100% de sulfate d'ammonium saturé. Depuis, au moins 21 différentes protéines de la famille S100 ont été identifiées.[4]
Les protéines S100A1 et S100B sont principalement exprimées par des cellules du systéme nerveux central, surtout dans les cellules du cytosol glial, mais également dans les cellules de mélanomes et, dans une certaine mesure, dans d'autres tissus. La protéine fonctionnelle, composées d'hétérodimères et d'homodimères des sous-unités A1 et B, est impliquée dans différentes activités régulatrices intra et extracellulaires.
Chez les patients atteints de mélanome malin, et surtout dans les stades II, III et IV, une augmentation des taux sériques de S100 peut indiquée une progression de la maladie. Des séries de dosages peuvent être utiles pour le suivi des patients et la surveillance de la réponse au traitement.[7,8,9,10,11,12,13]
Par ailleurs, les concentrations de S100 augmentent dans le LCR (liquide céphalo-rachidien) à la suite de certaines lésions cérébrales et sont relarguées dans le sang périphérique.
La S100 peut être détectée chez les patients présentant une lésion cérébrale survenue de diverses manières, tels que les traumatismes crâniens [14,15,16,17,18,19] ou les accidents cardiovasculaires.[20,21,22]

Principe
Méthode « sandwich ». Durée totale du cycle analytique : 18 minutes
- 1ère incubation : une prise d'essai de 20 µL est mise en présence d'un anticorps monoclonal anti-S100 spécifique biotinylé et d'un anticorps monoclonal anti-S100 spécifique marqué au ruthénium[a]. Il se forme un « sandwich ».
- 2e incubation : les microparticules tapissées de streptavidine sont ajoutées dans la cuvette réactionnelle. Le complexe immunologique est fixé à la phase solide par une liaison streptavidine-biotine.
- Le mélange réactionnel est transféré dans la cellule de mesure, les microparticules sont maintenues au niveau de l'électrode par un aimant. L'élimination de la fraction libre est effectuée par le passage de ProCell. Une différence de potentiel appliquée à l'électrode déclenche la production de luminescence qui est mesurée par un photomultiplicateur.
- Les résultats sont obtenus à l'aide d'une courbe de calibration. Celle-ci est générée, pour l'analyseur utilisé, par une calibration en 2 points et une courbe de référence mémorisée dans le code-barres du réactif.

a) $Ru(bpy)_3^{2+}$: Tris(2,2'-bipyridyl)ruthénium(II)

Réactifs - composition et concentrations

M Microparticules tapissées de streptavidine, 1 flacon contenant 6,5 mL (bouchon transparent) :
microparticules tapissées de streptavidine 0,72 mg/mL ; conservateur

R1 Ac anti-S100~biotine, 1 flacon contenant 9 mL (bouchon gris) :
anticorps (monoclonal de souris) anti-S100 biotinylé 1,0 mg/L ; tampon phosphate 50 mmol/L, pH 7,2 ; conservateur

R2 Ac anti-S100~$Ru(bpy)_3^{2+}$, 1 flacon contenant 9 mL (bouchon noir) :
anticorps (monoclonal de souris) anti-S100 marqué au ruthénium 1,0 mg/L ; tampon phosphate 50 mmol/L, pH 7,2 ; conservateur

Précautions d'emploi et mises en garde
Pour diagnostic *in vitro*
Observer les précautions habituelles de manipulation en laboratoire.
L'élimination de tous les déchets doit être effectuée conformément aux dispositions légales.
Fiche de sécurité disponible sur demande pour les professionnels.
Éviter la formation de mousse dans les réactifs et les échantillons de tous types (échantillons de patients, calibrateurs et contrôles).

Préparation des réactifs
Les réactifs contenus dans le coffret sont prêts à l'emploi et ne peuvent être utilisés séparément.
Toutes les informations nécessaires au déroulement du test sont mémorisées sur le code-barres des flacons de réactifs et doivent être saisies.

Conservation et stabilité
Conservation entre 2 et 8°C.
Ranger le coffret Elecsys S100 **en position verticale**, de manière à ce que toutes les microparticules soient rassemblées lors de l'homogénéisation qui précède l'analyse.
Stabilité :

Avant ouverture, entre 2 et 8°C	jusqu'à la date de péremption indiquée
Après ouverture, entre 2 et 8°C	12 semaines
Sur MODULAR ANALYTICS E170 et **cobas e** 601	8 semaines
Sur Elecsys 2010 et **cobas e** 411	8 semaines
Sur Elecsys 1010	4 semaines (conservation alternée au réfrigérateur et dans l'appareil entre 20 et 25°C, flacons ouverts au maximum 20 heures).

Prélèvement et préparation des échantillons
Seul le type d'échantillon suivant a été testé et peut être utilisé :
Sérum recueilli sur tubes standard ou contenant un gel séparateur.
Ne pas utiliser de plasma.
Stabilité : 8 heures entre 15 et 25°C, 2 jours entre 2 et 8°C, 3 mois à -20°C.
Les différents types d'échantillons indiqués ci-dessus ont été testés à l'aide d'une sélection de tubes de prélèvement disponibles dans le commerce au moment du test : les tubes de prélèvement des différents fabricants n'ont pas tous été testés. Les systèmes de prélèvement du sang de divers fabricants peuvent contenir différents matériaux pouvant, dans certains cas, influencer le résultat du test. En cas d'utilisation de tubes primaires (systèmes de prélèvement du sang), suivre les instructions données par le fabricant.
Centrifuger les échantillons contenant un précipité avant l'analyse. Ne pas utiliser d'échantillons inactivés par la chaleur. Les échantillons ou contrôles stabilisés par de l'azide ne doivent pas être utilisés.
S'assurer avant l'analyse que la température des échantillons, des calibrateurs et des contrôles se situe entre 20 et 25°C.

S100

En raison des risques d'évaporation, il est recommandé de doser les échantillons, les contrôles et les calibrateurs dans les 2 heures qui suivent leur mise en place sur les analyseurs.

Matériel fourni

Voir paragraphe « Réactifs - composition et concentrations ».

Matériel auxiliaire nécessaire

- Réf. 03289834, S100 CalSet pour 4 x 1 mL
- Réf. 03330648, PreciControl S100 : PreciControl S100 1 pour 2 x 2 mL et PreciControl S100 2 pour 2 x 2 mL
- Equipement habituel de laboratoire
- Analyseur Elecsys 1010/2010, MODULAR ANALYTICS E170 ou **cobas e**

Matériel auxiliaire pour les analyseurs Elecsys 1010/2010 et **cobas e** 411 :

- Réf. 11662988, ProCell, 6 x 380 mL, tampon système
- Réf. 11662970, CleanCell, 6 x 380 mL, solution de lavage pour la cellule de mesure
- Réf. 11930346, Elecsys SysWash, 1 x 500 mL, additif à la solution de lavage
- Réf. 11933159, Adaptateur pour SysClean
- Réf. 11706829, Elecsys 1010 AssayCup, 12 x 32 cuvettes réactionnelles ou Réf. 11706802, Elecsys 2010 AssayCup, 60 x 60 cuvettes réactionnelles
- Réf. 11706799, Elecsys 2010 AssayTip, 30 x 120 embouts de pipette

Matériel auxiliaire pour les analyseurs MODULAR ANALYTICS E170 et **cobas e** 601 :

- Réf. 04880340, ProCell M, 2 x 2 L, solution tampon
- Réf. 04880293, CleanCell M, 2 x 2 L, solution de lavage pour la cellule de mesure
- Réf. 03023141, PC/CC-Cups, 12 godets pour la thermorégulation de ProCell M et CleanCell M
- Réf. 03005712, ProbeWash M, 12 x 70 mL, solution de lavage de l'aiguille en fin de série et entre les changements de réactifs
- Réf. 12102137, AssayTip/AssayCup Combimagazine M, 48 blocs de 84 tubes à essai/embouts de pipettes, sacs pour déchets
- Réf. 03023150, WasteLiner (sacs pour déchets)
- Réf. 03027651, SysClean Adapter M, adaptateur pour SysClean

Pour tous les analyseurs :

- Réf. 11298500, Elecsys SysClean, 5 x 100 mL, solution de lavage du système

Réalisation du test

Pour garantir le bon fonctionnement du test, se conformer aux instructions relatives à l'analyseur utilisé indiquées dans la présente notice. Pour les instructions spécifiques de l'analyseur, se référer au manuel d'utilisation approprié.

L'analyseur effectue automatiquement l'homogénéisation des microparticules.

Les informations spécifiques du test mémorisées dans le code-barres doivent être saisies. Si, exceptionnellement, le code-barres ne peut être lu par l'appareil, saisir manuellement la série des 15 chiffres inscrits sur l'étiquette.

Analyseurs MODULAR ANALYTICS E170, Elecsys 2010 et **cobas e** : amener les réactifs réfrigérés à env. 20°C avant le chargement et les placer dans le plateau réactifs de l'appareil thermostaté à 20°C. Eviter la formation de mousse. L'analyseur **gère** le contrôle de la température, l'ouverture et la fermeture des flacons.

Analyseur Elecsys 1010 : amener les réactifs réfrigérés à env. 20-25°C et les placer dans le plateau réactifs/échantillons de l'analyseur (thermostaté entre 20 et 25°C). Eviter la formation de mousse. **Ouvrir** les flacons avant la mise en route de l'analyseur, puis les **refermer**. Les replacer au réfrigérateur après la série de dosages.

Calibration

Traçabilité : la méthode a été standardisé par pesée par rapport à la protéine S100 β/β.

Le code-barres des réactifs Elecsys S100 contient toutes les informations nécessaires à la calibration du lot. La courbe de référence est adaptée à l'analyseur à l'aide des calibrateurs Elecsys S100 CalSet.

Fréquence des calibrations : effectuer une calibration par lot en utilisant du réactif frais (ayant été enregistré depuis au maximum 24 heures sur l'analyseur). Une nouvelle calibration est recommandée pour :

Analyseurs MODULAR ANALYTICS E170, Elecsys 2010 et **cobas e** :

- après 1 mois (28 jours) pour un même lot de réactif
- après 7 jours pour un même flacon de réactif resté sur l'analyseur

Analyseur Elecsys 1010 :

- à chaque nouveau coffret
- après 7 jours entre 20 et 25°C
- après 3 jours entre 25 et 32°C

Pour tous les analyseurs :

- quand elle s'avère nécessaire : par ex. si les résultats du contrôle de qualité se situent en dehors des limites de confiance.

Contrôle de qualité

Utiliser Elecsys PreciControl S100 1 et 2.

D'autres contrôles appropriés peuvent également être utilisés.

Il est recommandé de doser les sérums de contrôle en simple au moins une fois toutes les 24 heures pendant une routine, pour chaque nouveau coffret et lors d'une calibration. La fréquence des contrôles et les limites de confiance doivent être adaptées aux exigences du laboratoire. Les résultats doivent se situer dans les limites de confiance définies.

Chaque laboratoire devra établir la procédure à suivre si les résultats se situent en dehors de ces limites.

Calcul des résultats

L'analyseur calcule automatiquement la concentration en analyte de chaque échantillon. Les résultats sont exprimés au choix en µg/L, en ng/mL ou en pg/mL.

Limites d'utilisation - interférences

Le test n'est pas influencé par l'ictère (bilirubine < 428 µmol/L ou < 25 mg/dL), l'hémolyse (Hb < 0,621 mmol/L ou < 1,0 g/dL), la lipémie (Intralipid < 1500 mg/dL) et la biotine (< 205 nmol/L ou < 50 ng/mL).

Critère d'acceptabilité : recouvrement ± 10% par rapport à la valeur initiale.

Chez les patients traités par de fortes doses de biotine (> 5 mg/jour), il est recommandé d'effectuer le prélèvement de l'échantillon au moins 8 heures après la dernière administration.

Le résultat n'est pas influencé par le facteur rhumatoïde jusqu'à 1000 UI/mL.

On n'a pas observé d'effet crochet jusqu'à 10 µg de S100/mL.

L'influence de 18 médicaments fréquemment administrés a été recherchée *in vitro* : aucune interférence n'a été observée.

Comme dans tous les tests contenant des anticorps monoclonaux de souris, les échantillons de patients ayant reçu des préparations d'anticorps monoclonaux de souris à des fins thérapeutiques ou diagnostiques peuvent donner des résultats erronés.

Dans de rares cas, des titres très élevés d'anticorps anti-streptavidine ou anti-ruthénium peuvent conduire à des interférences.

Le test contient des additifs permettant de minimiser ces effets.

Pour le diagnostic, les résultats doivent toujours être confrontés aux données de l'anamnèse du patient, au tableau clinique et aux résultats d'autres examens.

Domaine de mesure

0,005-39 µg/L (défini par la limite de détection et le maximum de la courbe de référence). Les taux situés en dessous de la limite de détection sont exprimés de la manière suivante : < 0,005 µg/L et les taux situés au-dessus du domaine de mesure de la manière suivante : > 39 µg/L.

Dilution des échantillons

Les échantillons dont le concentrations en S100 se situent au-dessus du domaine de mesure peuvent être dilués à l'aide de Elecsys S100 Cal1 ou de sérum humain exempt de S100. L'utilisation de Elecsys Diluent Universal n'est pas recommandée. Rapport de dilution recommandé : 1/5 (dilution manuelle). La concentration obtenue avec l'échantillon dilué doit être > 1 µg/L. Après dilution manuelle, le résultat obtenu doit être multiplié par le facteur de dilution.

12177293001V5

S100

S100

Valeurs de référence

- *Adultes apparemment sains et patients atteints de mélanome malin*

Les valeurs suivantes ont été obtenues à partir de dosages effectués avec le test Elecsys S100 sur des échantillons de sujets apparemment sains et de patients atteints de mélanome malin à différents stades de la tumeur et sous suivi thérapeutique :

Population	Sous-groupe	Nbre d'échant. (patients)	Médiane µg/L	95e percentile	Nbre d'échant. au-dessus du seuil (> 0,105 µg/L)[b]
Adultes apparemment sains		206 (206)	0,046	0,105	10 sur 206 (4,9%)
Patients avec mélanome malin (à tous stades et sous suivi thérapeutique)	ASM[c]	821 (408)	0,044	0,109	45 sur 821 (5,5%)
	Métastases ganglionnaires régionales	32 (24)	0,047	0,120	4 sur 32 (12,5%)
	Métastases ganglionnaires lointaines/ cutanées	21 (15)	0,093	0,511	10 sur 21 (47,6%)
	Métastases viscérales/ distantes	70 (48)	0,077	0,759	30 sur 70 (42,9%)

b) Nombre d'échantillons d'adultes apparemment sains > 95e percentile
c) Aucun signe de maladie, absence de tumeur

- *Adultes atteints de souffrance cérébrale potentielle*

Les taux de Elecsys S100 ont été mesurés chez des patients présentant une léger traumatisme crânien (Glasgow Coma Score, GCS 13-15) et au moins un symptome au cours des 3 heures suivant l'accident à l'origine du traumatisme. Une tomographie de la boîte crânienne (TBC) a été effectuée au cours des 6 heures suivant l'accident. En utilisant le 95e percentile de sujets apparemment sains (0,105 µg/L) comme valeur seuil, les résultats obtenus avec le test Elecsys S100 comparés à la scannographie de référence étaient les suivants : VPN (valeur prédictive négative) 99,7% ; VPP (valeur prédictive positive) 11% ; sensibilité 98,8% ; spécificité 32,9% (intervalle de confiance de 95% : VPN 99,1-100% ; VPP 8,8-13,3% ; sensibilité 96,5-100% ; spécificité 30-35,9%)

	TBC positive	TBC négative	Total
Elecsys S100 positifs	83	670	753
Elecsys S100 négatifs	1[d]	329	330
Total	84	999	1083

d) 0,098 µg/L

Chaque laboratoire devra vérifier la validité de ces valeurs et établir au besoin ses propres domaines de référence selon la population examinée.

Performances analytiques

Les performances analytiques indiquées ci-dessous sont représentatives. Les résultats obtenus au laboratoire peuvent différer de ceux-ci.

Précision

La reproductibilité a été déterminée à l'aide de réactifs Elecsys, de pools de sérum humain et de contrôles, selon un protocole modifié (EP5-A) du N.C.C.L.S. (National Committee for Clinical Laboratory Standards). Chaque échantillon a été analysé 6 fois par jour pendant 10 jours (n = 60) ; CV intra-série sur l'analyseur MODULAR ANALYTICS E170, n = 21. Les résultats suivants ont été obtenus :

Analyseurs Elecsys 1010/2010 et **cobas e** 411					
		**Précision intra-série**		**Précision totale**	
Echantillon	Moyenne µg/L	DS µg/L	CV %	DS µg/L	CV %
Sérum humain 1	0,08	0,002	2,1	0,002	2,8
Sérum humain 2	0,24	0,003	1,3	0,005	2,0
Sérum humain 3	2,13	0,042	2,0	0,052	2,4
PreciControl S100_1	0,26	0,005	1,8	0,006	2,3
PreciControl S100_2	3,33	0,046	1,4	0,056	1,7

Analyseurs MODULAR ANALYTICS E170 et **cobas e** 601						
	**Précision intra-série**			**Précision totale**		
Echantillon	Moyenne µg/L	DS µg/L	CV %	Moyenne µg/L	DS µg/L	CV %
Sérum humain 1	0,09	0,001	1,0	0,09	0,003	3,1
Sérum humain 2	0,26	0,005	1,8	0,26	0,006	2,5
Sérum humain 3	2,25	0,015	0,7	2,24	0,064	2,9
PreciControl S100_1	0,27	0,004	1,3	0,28	0,007	2,7
PreciControl S100_2	3,39	0,031	0,9	3,38	0,092	2,7

Sensibilité analytique (limite inférieure de détection)

< 0,005 µg/L

La limite de détection correspond au plus faible taux d'analyte mesurable pouvant être distingué de zéro. Elle est obtenue par le calcul et représente la concentration du standard le plus faible de la courbe de référence + 2 écarts-type (calibrateur de référence, standard 1 + 2DS, précision intra-série, n = 21).

Comparaison de méthodes

Une comparaison du test Elecsys S100 (y) avec les tests Liamat Sangtec100 (x_1) et Liaison Sangtec100 (x_2), effectuée à partir d'échantillons cliniques de patients présentant un mélanome malin, a conduit à l'obtention des corrélations suivantes :

Passing/Bablok[23]

Elecsys/Liamat (x_1)
$y = 0,550x_1 + 0,025$
r = 0,729
Nombre d'échantillons analysés : 934
Les concentrations des échantillons étaient situées entre env. 0,00 et 9,87 µg/L.

Elecsys/Liaison (x_2)
$y = 0,783x_2 + 0,003$
r = 0,857
Nombre d'échantillons analysés : 379
Les concentrations des échantillons étaient situées entre env. 0,01 et 2,08 µg/L.

Spécificité analytique

Les réactions croisées par les dimères de la protéine S100A1 (αα) sont < 1%.

Sensibilité fonctionnelle

< 0,02 µg/L

La sensibilité fonctionnelle est définie comme étant la concentration en analyte la plus basse donnant un coefficient de variation inter-série de 20%.

Bibliographie

1. Donato R. S100: a multigenic family of calcium-modulated proteins of the EF-hand type with intracellular and extracellular functional roles. Int J Biochem Cell Biol 2001;33:637-668.
2. Zimmer DB, Cornwall EH, Landar A, Song W. The S100 protein family: history, function, and expression. Brain Res Bull 1995;4:417-429.
3. Moore BW. A soluble protein characteristic of the nervous system. Biochem Biophys Res Comm 1965;19:739-744.
4. Donato R. Intracellular and Extracellular Roles of S100 Proteins. Microscopy Research and Technique 2003;60:540-551.
5. Heizmann CW, Fritz G, Schäfer BW. S100 Proteins: Structure, Function and Pathology. Frontiers in Bioscience 2002;7:1356-1368.
6. Zimmer DB, Sadosky PW, Weber DJ. Molecular Mechanisms of S100-Target Protein Interactions. Microscopy Research and Technique 2003;60:552-559.
7. Guo HB, Stofffel-Wagner B, Bierwirth T, Mezger J, Klingmüller D. Clinical significance of serum S100 in metastatic malignant melanoma. Eur J Cancer 1995;31A:924-928.
8. Mohammed MQ, Abraha HD, Sherwood RA, MacRae K, Retsas S. Serum S100β protein as a marker of disease activity in patients with malignant melanoma. Med Oncol 2001;18:109-120.
9. Krähn G, Kaskel P, Sander S, Waizenhöfer PJ, Wortmann S, Leiter U, Peter RU. S100β is a more reliable tumor marker in peripheral blood for

patients with newly occurred melanoma metastases compared with MIA, albumin and lactate-dehydrogenase. Cancer Research 2001;21:1311-1316.
10. von Schoultz E, Hansson LO, Djureen E, Hansson J, Kärnell R, Nilsson B, Stigbrand T, Ringborg U. Prognostic value of serum analysis S-100β protein in malignant melanoma. Melanoma Research 1996;6:133-137.
11. Abraha HD, Fuller LC, Vivier AW, Higgins EM, Sherwood RA. Serum S-100 protein: a potentially useful prognostic marker in cutaneous melanoma. Br J Dermatol 1997;137:381-385.
12. Garbe C, Leiter U, Ellwanger U, Blaheta HJ, Meier F, Rassner G, Schittek B. Diagnostic Value and Prognostic Significance of Protein S-100 β, Melanoma-Inhibitory Activity, and Tyrosinase/MART-1 Reverse Transcription-Polymerase Chain Reaction in the Follow-Up of High-Risk Melanoma Patients. Cancer 2003;97(7);1737-1745.
13. Hauschild A, Engel G, Brenner W, Gläser R, Mönig R, Henze E, Christophers E. Predictive value of serum S100B for monitoring patients with metastatic melanoma during chemotherapy and/or immunotherapy. British Journal of Dermatology 1999;140:1065-1071.
14. de Kruijk JR, Leffers P, Menheere PPCA, Meerhoff S, Twijnstra A. S-100B and neuron-specific enolase in serum of mild traumatic brain injury patients – a comparison with healthy controls. Acta Neurol Scand 2001;103:175-179.
15. Herrmann M, Curio N, Jost S, Grubich C, Ebert AD, Fork ML, Synowitz H. Release of biochemical markers of damage to neuronal and glial brain tissue is associated with short and long term neuropsychological outcome after traumatic brain injury. J Neurol Neurosurg Psychiatry 2001;70:95-100.
16. Ingebrigtsen T, Romner B, Marup-Jensen S, Dons M, Lundqvist C, Bellner J, Alling C, Börgesen SE. The clinical value of serum S-100 protein measurements in minor head injury: a Scandinavian multicentre study. Brain Injury 2000;14:1047-1055.
17. Biberthaler P, Mussack T, Wiedemann E, Kanz KG, Koelsch M, Gippner-Steppert C, Jochum M. Evaluation of S-100b as a specific marker for neuronal damage due to minor head trauma. World J Surg 2001;25:93-97.
18. Herrmann M, Jost S, Kutz S, Ebert AD, Kratz T, Wunderlich MT, Synowitz H. Temporal profile of release of neurobiochemical markers of brain damage after traumatic brain injury is associated with intracranial pathology as demonstrated in cranial computerized tomography. J Neurotrauma 2000;17:113-121.
19. Townend WJ, Guy MJ, Martin B, Yates DW. Head injury outcome prediction in the emergency department: a role for S-100B? J Neurol Neurosurg Psychiatry 2002;73:542-546.
20. Abraha HD, Butterworth RJ, Bath PMW, Wassif WS, Garthwaite J, Sherwood RA. Serum S-100 protein, relationship to clinical outcome in acute stroke. Ann Clin Biochem 1997;34:366-370.
21. Fassbender K, Schmidt R, Schreiner A, Fatar M, Mühlhauser F, Daffertshofer M, Hennerici M. Leakage of brain-originated proteins in peripheral blood: temporal profile and diagnostic value in early ischemic stroke. J Neurol Sci 1997;148:101-105.
22. Thornhill S, Teasdale GM, Murray GD, McEwen J, Roy CW, Penny KL. Disability in young people and adults one year after head injury: prospective cohort study. BMJ 2000;320:1631-1635.
23. Bablok W, et al. A General Regression Procedure for Method Transformation. J Clin Chem Clin Biochem 1988;26:783-790.

Pour de plus amples informations, se référer au manuel de l'opérateur de l'analyseur utilisé, aux fiches techniques respectives, au dossier « Product Information » et aux notices d'utilisation de tous les réactifs nécessaires.

Les modifications importantes par rapport à la version précédente sont signalées par une barre verticale dans la marge. Les modifications concernant les données contenues dans le code-barres doivent être entrées manuellement.

©2007 Roche Diagnostics.

CE

Roche Diagnostics GmbH, D-68298 Mannheim

Roche

APPENDIX 6
FORMULAIRE DE RECUEIL D'EVENEMENT INDESIRABLE GRAVE

A FAXER INPERATIVEMENT DANS LES 24 H AU PROMOTEUR AU 04.73.75.47.30

Date de déclaration : I__I__I I__I__I I__I__I__I__I

1. IDENTIFICATION DE LA RECHERCHE BIOMEDICALE

N° IDRCB : **2016-A00195-46**

Code Protocole attribué par le promoteur : **PHRC N 2015 BOUVIER (PROS100B)**

Titre de l'essai clinique : **Etude interventionnelle évaluant l'apport du dosage sanguin de la protéine S100B dans la prise en charge du traumatisme crânien léger de l'enfant**

2. INFORMATIONS SUR LE PATIENT

Identification du patient :

Nom (initiales) : I__I__I__I

Prénom : I__I__I

N° Patient (CRF) : I__I__I__I__I

Date de naissance : I__I__I I__I__I I__I__I__I__I

Age : I__I__I__I

Poids (kg) : I__I__I__I

Taille (cm) : I__I__I__I

Sexe : F ☐ M ☐

Antécédents médicaux et familiaux pertinents :

3. INFORMATIONS SUR LE(S) PRODUIT(S)

Nom commercial	Dosage	N° de lot	Voie d'adm.	Posologie (Dose / rythme)	Indication thérapeutique	Début de traitement (date et heure)	Fin de traitement (date et heure (✱)	Code levé ?
1								Oui ☐ Non ☐
2								Oui ☐ Non ☐
3								Oui ☐ Non ☐

✱ Noter « continue » si le traitement n'est pas arrêté.

Si levée de l'insu, résultat : ____________________________

Disparition de l'événement après arrêt du traitement ? Oui ☐ Non ☐

4. EVALUATION DU LIEN DE CAUSALITE

Selon le promoteur, l'événement semble plutôt lié :

Au(x) traitement(s) à l'essai ☐
Au(x) traitement(s) associés ☐
A une maladie intercurrente ☐

Au(x) procédure(s) de l'essai ☐
Autre, à préciser : ☐
____

Commentaires pertinents :

____

____

Selon l'investigateur, l'événement semble plutôt lié :

Au(x) traitement(s) à l'essai ☐
Au(x) traitement(s) associés ☐
A une maladie intercurrente ☐

Au(x) procédure(s) de l'essai ☐
Autre, à préciser : ☐
____

Commentaires pertinents :

____

____

5. INFORMATIONS SUR LES TRAITEMENTS ASSOCIES MEDICAMENTEUX OU NON (à l'exclusion de ceux utilisés pour traiter l'événement)

Nom commercial ou DCI	Dosage	N° de lot	Voie d'adm.	Posologie (Dose / rythme)	Indication thérapeutique	Début de traitement (date, heure)	Fin de traitement (date, heure)
4							
5							
6							
7							

6. INFORMATIONS SUR L'EVENEMENT INDESIRABLE GRAVE

☐ Décès
☐ Mise en jeu du pronostic vital
☐ Invalidité ou incapacité
☐ Hospitalisation ou prolongation d'hospitalisation
Date de début : I__I__I I__I__I I__I__I
Date de fin : I__I__I I__I__I I__I__I
☐ Anomalie congénitale

Lieu de survenue : ______________________
Date de survenue : I__I__I I__I__I I__I__I
Heure de survenue : I__I__I I__I__I

☐ Autre (préciser) :___________________________

Description de l'événement indésirable - Préciser les symptômes prédominants, la chronologie, éventuellement le diagnostic et les traitements de l'événement (joindre les comptes-rendus anonymisés d'hospitalisation d'examens et/ou résultats de laboratoire) :

____

____

____

____

____

____

Evolution : ☐ Amélioration ☐ Stabilité ☐ Aggravation ☐Survie avec séquelles
☐ Décès (cause : lié à l'événement ☐Oui ☐Non) ☐Evolution inconnue

Description (joindre les comptes-rendus anonymisés d'hospitalisation d'examens et/ou résultats de laboratoire) :

____

____

____

Un ou des produits ont-ils été réintroduits ?

Oui		N° N° N°	Non	

Réapparition de l'événement après réintroduction ?

Oui		N° N° N°	Non	

Si oui, date : I__I__I I__I__I I__I__I heure : I__I__I I__I__I

DIAGNOSTIC DIFFERENTIEL

Autres étiologies envisagées:

____

____

Examens complémentaires réalisés et résultats :

____

____

7. INFORMATIONS SUR LE DECLARANT

Nom et adresse du centre investigateur :

____

Centre n° : _____________ Investigateur : ____
Tél. : __________________ Email : _____________________@______________________
Service : _______________

Nom et qualité du déclarant : ________________________________ Signature :

INFORMATIONS SUR LE PROMOTEUR (*cadre réservé au promoteur, ne pas remplir*)

Nom et adresse du promoteur :

____

____

Date de réception par le promoteur : I__I__I I__I__I I__I__I Type de rapport : ☐ initial
☐ suivi n°_________

Date de déclaration aux autorités : I__I__I I__I__I I__I__I

N° d'identification de l'événement par le promoteur : ______________________

Identification de l'autorisation de recherche : ______________________

Nom et qualité du représentant du promoteur: ____

Tél. : __________________ Email : ___________________@________________________

Fax : __________________ Signature :

Comité de
Protection des
Personnes
Sud-Est 6

Clermont Ferrand, le 08 juin 2016

CHU de CLERMONT-FERRAND
REÇU LE
10 JUIN 2016
RECHERCHE CLINIQUE

Monsieur A. SALAGNAC
Direction de la Recherche Clinique
Direction Générale Adjointe
HOPITAL G. MONTPIED

Titre de l'essai :	**Etude interventionnelle évaluant l'apport du dosage sanguin de la protéine S100B dans la prise en charge du traumatisme crânien léger de l'enfant**
Promoteur	CHU Clermont Ferrand
Investigateur	Dr. Damien BOUVIER
Réf. CPP	AU 1257
Réf. ID-RCB	2016-A00195-46
Réf. Promoteur	PHRC N 2015 BOUVIER
Acronyme	PROS100B

Documents examinés :	Numéro et date de version
Courriel de réponse du	13 mai 2016
Courrier de réponse de l'investigateur du	03 mai 2016
Tableau comparatif	--
Document additionnel du	13 mai 2016
Résumé	Version N°2 du 02 mai 2016
Information/Consentement	Version N°2 du 02 mai 2016
CVs des investigateurs	- -
Protocole	Version N°2 du 02 mai 2016

Monsieur,

Nous accusons réception en date du 13 mai 2016 des compléments d'information apportés à l'étude ci-dessus référencée et ce dossier a donc été réexaminé en date du 03 juin 2016.

Les renseignements fournis répondant de façon satisfaisante aux questions posées, le Comité donne un avis favorable à la réalisation de la recherche, avis rendu sur l'appréciation du respect des dispositions de l'article L.1121-1 et sur la validité de la recherche selon les dispositions de l'article L. 1123-7.

Vous trouverez donc ci-joint un avis favorable qui avait été émis lors de la réunion du 03 juin 2016.

Veuillez agréer, Monsieur, l'expression de nos salutations distinguées.

Le Vice-Président,
Pr. J.E. BAZIN

C.H.U. – Administration Centrale - 58 rue Montalembert - BP. 69 – 63003 CLERMONT FERRAND Cedex 1
Tel. 04.73.75.10.73 - Fax. 04.73.75.10.69. - Courriel cpp-sudest6@chu-clermontferrand.fr
Numéro IRB : IRB00008526

Comité de Protection des Personnes
Sud-Est 6

CHU de CLERMONT-FD REÇU LE 10 JUIN [illegible] RECHERCHE [illegible]

Clermont Fd, le 08 juin 2016

Monsieur A. SALAGNAC
Direction de la Recherche Clinique
Direction Générale Adjointe
HOPITAL G. MONTPIED

Titre de l'essai :	**Etude interventionnelle évaluant l'apport du dosage sanguin de la protéine S100B dans la prise en charge du traumatisme crânien léger de l'enfant**
Promoteur	CHU Clermont Ferrand
Investigateur	Dr. Damien BOUVIER
Réf. CPP	AU 1257
Réf. ID-RCB	2016-A00195-46
Réf. Promoteur	PHRC N 2015 BOUVIER
Acronyme	PROS100B

Documents examinés :	Numéro et date de version
Courriel de réponse du	13 mai 2016
Courrier de réponse de l'investigateur du	03 mai 2016
Tableau comparatif	--
Document additionnel du	13 mai 2016
Résumé	Version N°2 du 02 mai 2016
Information/Consentement	Version N°2 du 02 mai 2016
Protocole	Version N°2 du 02 mai 2016
CVs des investigateurs	- -

Le Comité a été saisi le **: 13 mai 2016**

par : **Monsieur A. SALAGNAC** représentant le promoteur : **CHU CLERMONT-FERRAND**

d'une demande d'avis pour un projet de recherche ci-dessus référencé.

Le Comité a examiné les informations relatives à cet essai lors de sa séance du **:**
03 juin 2016, et le projet revu à sa demande comportant certaines modifications.

Ont participé à la délibération :

**Premier Collège**		**Deuxième Collège**	
Pr. Jean-Etienne BAZIN (T)	Anesthésiste Réanimateur	M. Pascal DESSENNE (S)	Psychologue clinicien
Mme Aurélie CABRESPINE (T)	Ingénieur en recherche	M J. Mary ROUSSEAU (T)	Magistrat honoraire
Dr. Sylvaine BŒUF-GIBOT (T)	Médecin Généraliste	Mme Marion LIBERT (S)	Avocate
Dr. Daniel TERRAL (S)	Pédiatre	Mme A. Marie REGNOUX (T)	Avocate
Dr. Sylvain LEVALLOIS (S)	Pédopsychiatre	Pr. Christiane FORESTIER (T)	Représentant l'ADAPEI 63
M. Fabrice KWIATKOWSKI (S)	Biostatisticien	M. Daniel VIGIER (S)	Représentant l'ASDA
Mme M.A CIVIALE (T)	Pharmacien hospitalier	Mme Jeany GALLIOT (S)	Représentant l'ADMD
Mme Anne KEBOUR (T)	Cadre Sup. de Santé		
M. Franck HENTZ (S)	Cadre Sup. de Santé		

Le Comité a adopté la délibération suivante : **AVIS FAVORABLE**

Le Vice-Président,
Pr. J.- Etienne BAZIN

ansm

Agence nationale de sécurité du médicament et des produits de santé

AUTORISATION D'ESSAI CLINIQUE NE PORTANT PAS SUR UN PRODUIT DE SANTE (ESSAI-HPS)

Nombre de pages : 1
(Incluant la page de garde)

Envoi par Télécopie

Date : 28/04/16

Identifiants de l'essai clinique					
Titre	Evaluation de l'intérêt du dosage sérique de la protéine S100B dans la prise en charge des traumatismes crâniens légers pédiatriques				
Promoteur	CHU Clermont-Ferrand			Réf. CPP	Non disponible
Réf. Promoteur	PROS100B	N° ID RCB	2016-A00195-46	Réf. ANSM	**160358B-31**

**Expéditeur**	**Destinataire** (demandeur : nom / société / tél.)
ANSM / Direction Produit NEURHO / Equipe SYNAPS	Patrick Lacarin<br>CHU Clermont-Ferrand<br>04 73 75 11 95
Dossier suivi par : Nicolas Glasser<br>Tél : 33 (0) 1 55 87 30 51 / Fax : 33 (0) 1 55 87 33 32<br>Mel : nicolas.glasser@ansm.sante.fr	Fax **04 73 75 47 30**

**CPP destinataire en copie**	Sud-Est VI (Clermont-Ferrand)	**Fax**	**04.73.75.10.69**

Vu le code de la santé publique et notamment ses articles L. 1123-8, R. 1123-32 et vu le dossier de demande d'autorisation d'essai clinique adressé à l'Agence nationale de sécurité du médicament et des produits de santé (ANSM) ;

L'autorisation mentionnée à l'article L. 1123-8 du code de la santé publique est accordée pour l'essai clinique cité en objet. Cette autorisation est valable pour toute la durée de l'essai à compter de la date de la présente décision.

Toutefois, conformément à l'article R. 1123-33 du code de la santé publique, la présente autorisation devient caduque si la recherche n'a pas débuté dans un délai d'un an.

Cette autorisation est délivrée, considérant que les examens complémentaires mis en œuvre pour les besoins de la recherche doivent être effectués avec les mêmes mesures de sécurité que celles habituellement recommandées en pratique clinique. Il revient donc aux investigateurs et intervenants de se conformer aux usages en vigueur.

En outre, je vous rappelle notamment que pendant le déroulement de la recherche et pour ce qui concerne l'ANSM :

- toute modification substantielle du dossier initialement soumis doit faire l'objet d'une demande d'autorisation en vertu des articles L. 1123-9 et R. 1123-35 du code de la santé publique ;
- les effets indésirables graves inattendus ainsi que les faits nouveaux susceptibles de porter atteinte à la sécurité des personnes doivent être déclarés en vertu des articles L. 1123-10 et R. 1123-46 du code de la santé publique.

La directrice Adjointe
Direction des médicaments en neurologie
psychiatrie, antalgie, rhumatologie, pneumologie
ORL, ophtalmologie, stupéfiants

Nathalie RICHARD

Je vous demande de transmettre toute demande d'informations complémentaires concernant ce dossier par courriel adressé à la boite : **ams-essaiscliniques**@ansm.sante.fr. Je vous précise qu'il vous est possible d'utiliser à cet effet le système de messagerie électronique sécurisée Eudralink. Lors de l'envoi de ces dossiers, je vous demande de veiller à reporter dans l'objet du message les mentions suivantes :
- pour les MS transmises à l'Ansm pour information : **MSI/ Réf ANSM du dossier**
- pour les MS soumises pour autorisation ou pour les dossiers mixtes (comportant des modifications soumises pour autorisation et d'autres pour information) : **MSA/ Réf ANSM du dossier**

Si vous ne recevez pas toutes les pages de cette télécopie, veuillez contacter le secrétariat de la Direction Produit NEURHO **/ Equipe** SYNAPS **au : 33 (0) 1 55 8733 41.**

Confidentialité
Cette transmission est à l'attention exclusive du(des) destinataires ci-dessus mentionné(s) et peut contenir des informations privilégiées et/ou confidentielles. Si vous n'êtes pas le destinataire voulu ou une personne mandatée pour lui remettre cette transmission, vous avez reçu ce document par erreur et toute utilisation, révélation, copie ou communication de son contenu est interdite. Si vous avez reçu cette transmission par erreur, veuillez nous en informer par téléphone immédiatement et nous retourner le message original par courrier. Merci.

Confidentiality
This transmission is intended to the addressee(s) listed above only and may contain preferential or/and confidential information. If you are not the intended recipient, you are hereby notified that you have received the document by mistake and any use, disclosure, copying or communication of the content of this transmission is prohibited. If you have received this transmission by mistake, please call us immediately and return the original message by mail. Thank you.

Interventional study assessing evaluation of the interest of serum S100B protein determination in the management of pediatric mild traumatic brain injury

Running title: PROS100B

Version: 8 dated: 08/10/2019

Sponsor Code	ANSM registration number
PHRC N 2015 BOUVIER	2016-A00195-46

Sponsor: **Clermont-Ferrand Teaching Hospital**
58 Rue de Montalembert
63003 Clermont-Ferrand Cedex 1
France

Coordinating investigator: ***Dr. Damien Bouvier (Physician-Biologist, Hosp.Practitioner assistant)***
Medical Biochemistry and Molecular Biology Clermont-Ferrand Teaching Hospital
58, Rue Montalembert 63003 Clermont-Ferrand cedex 1 France

Co-investigator: ***Prof. André Labbé (Pediatrician, Univ. Prof./Hosp.Practitioner, department head)***
Pediatrics Unit Hôpital Estaing Clermont-Ferrand Teaching Hospital
Place Lucie et Raymond Aubrac 63003 Clermont-Ferrand cedex 1 France

Scientific collaborator: ***Prof. Vincent Sapin (Biologist, Univ. Prof./Hosp.Practitioner, department head)***
Medical Biochemistry and Molecular Biology Clermont-Ferrand Teaching Hospital
58, Rue Montalembert 63003 Clermont-Ferrand cedex 1 France

Methodologist: ***Bruno Pereira (PhD)***
Clinical Research and Innovation Delegation Clermont-Ferrand Teaching Hospital
Villa annexe IFSI 58, rue Montalembert 63003 Clermont-Ferrand cedex 1 France

Economist: ***Charline Mourgues (MSc)***
Clinical Research and Innovation Delegation Clermont-Ferrand Teaching Hospital
Villa annexe IFSI 58, rue Montalembert 63003 Clermont-Ferrand cedex 1 France

ABSTRACT

Background: Mild traumatic brain injury (mTBI) (Glasgow Coma Scale score between 13 and 15) is a very common reason for presentation to pediatric emergency departments. So as not to overlook the risk of complications, which occur at a rate of 0-7%, measures such as cranial computed tomography (CCT-scan) and/or short inpatient observation are prescribed. Ultimately, the majority of these measures could be avoided (Homer and Kleinman, 1999) and a large Australian cohort shows that the risk of brain tumors is 2.44 times higher for children who had a CCT-scan (3.24 for age 1-4 years) (Mathews *et al.,* 2013). Assay of a sensitive biomarker in blood, such as the S100B protein, has the potential to reduce the number of these unnecessary measures (Bouvier *et al.,* 2012). Indeed, in this context, we have shown that this assay has a sensitivity of 100% (positive test associated with brain lesions in CCT-scan and/or the appearance of clinical complications) and a negative predictive value of 100% (negative test associated with the lack of brain damage to the CCT-scan and/or the occurrence of clinical complications). Thus, the benefit to the patient would be a support simplified compared to conventional care.

Primary objective*:* Evaluate the utility of serum S100B measurement in the management of pediatric mTBI by demonstrating a decrease in the proportion of CCT-scan prescribed in the "S100B management" intervention arm compared with the "conventional management" control arm, hypothesizing a 30% decrease in the number of CCT-scan between the intervention *versus* control arms.

Secondary objectives*:* Demonstrate the utility of serum S100B measurement with respect to:

- reduction in the time spent in the pediatric emergency room
- reduction in the duration of hospitalization
- reduction in radiation exposure (mSv)
- reduction in sedation and use of sedatives
- detection of complications (intracranial lesions) by CCT-scan which can occur at a rate of 0-7% in patients with mTBI (American Academy of Pediatrics, 1999)
- absence of intercurrent events at 48 hours and 3 weeks after mTBI
- compliance of emergency physicians with the S100B assay
- reduction of the cost of management

Type of study: The proposed protocol is a randomized, multicenter, open, prospective, interventional study (11 centers) using a stepped wedge cluster design, with two arms:

- Intervention group "S100B management"
- Control group "Conventional management"

This stepped wedge cluster randomization (stratified by cluster size) was chosen 1) to improve feasibility in emergency department and 2) to avoid the major risk of contamination bias in the control group.

Number of centers: 11
Clermont-Ferrand, Limoges, Lyon, Marseille, Montpellier, Nice, Reims, Saint-Etienne, Nîmes, Vichy, Nantes (Teaching Hospital - France)

Description of the study: Children in the "conventional management" control arm will have CCT-scan or be hospitalized according to the current recommendations of the French Society of Pediatry (SFP). In the "S100B management" intervention arm, blood sampling to determine serum S100B protein levels will take place within 3 hours after the mTBI and subsequent management will depend on the assay result (which is available 1 hour after arrival in the laboratory, dosing with the same technique of immunoassay in the 11 centers). If S100B is in normal range, the children will be discharged from the emergency department after 6 hours of observation. If the result is abnormal, CCT-scan or hospitalization will be prescribed in accordance with current SFP recommendations.

Primary outcome measure: The primary outcome measure will be the proportion of CCT-scans performed (absence/presence of CCT-scan for each patient) in the 48 hours following the mTBI, compared between the two arms ("S100B management" intervention *versus* "conventional management" control group).

Sample size: 4000 (2000 in each arm)

Inclusion criteria:
- Age ≤ 16 years
- Therapeutic management within 3 hours after the mTBI
- Glasgow Coma Scale score of 15 classically managed by hospitalization and/or CCT-scan as per SFP recommendations.

Non-inclusion criteria:
- Patient already enrolled in another therapeutic trial with drug administration
- Down syndrome
- Melanoma
- Refusal of child
- Refusal of parents or legal guardian
- Child with Glasgow Coma Scale ≤ 14
- Child with head injury and Glasgow Coma Scale score of 15 not requiring hospitalization and/or CCT-scan as per SFP recommendations.

Brief description of the products: This research protocol corresponds to a diagnostic study and no products will be administered to patients. Determination of serum S100B concentrations will be carried out by the medical biology laboratory at each center using the same automated immunoassay method (CE mark).

Conduct of the study: The study will run over a period of 60 months at 11 participating centers.

Summary of the benefits and foreseeable and known risks for subjects participating in the research: The risks to the patient are minimal since the probability of a false negative result is virtually nul. Furthermore, the serum assay of S100B protein is minimally invasive because it requires only a single blood sampling using a micro-method.

CONTENTS

ABBREVIATIONS **8**

1. GENERAL INFORMATION **9**

1.1. TITLE OF THE RESEARCH 9
1.2. SPONSOR CODE 9
1.3. SPONSOR 9
1.4. COORDINATION AND MONITORING 9
1.5. INVESTIGATORS 9
1.5.1. Coordinating investigator and collaborator *9*
1.5.2. Scientific collaborators and Co-investigators *10*
1.6. ASSOCIATE PARTNERS 12
1.7. STUDY SITES 13
1.8. DATA PROCESSING 14
1.9. ETHICS COMMITTEE 14
1.10. ESTIMATED TIME FRAME 14

2. STUDY RATIONALE / SCIENTIFIC JUSTIFICATION **14**

2.1. CURRENT STATE OF SCIENTIFIC KNOWLEDGE 14
2.2. HYPOTHESES AND OBJECTIVES 16
2.3. SUMMARY OF THE BENEFITS AND FORESEEABLE AND KNOWN RISKS FOR SUBJECTS PARTICIPATING IN THE RESEARCH 17
2.4. EXPECTED IMPACT 18
2.5. REFERENCES TO THE SCIENTIFIC LITERATURE AND TO PERTINENT DATA SERVING AS A BASIS FOR THE RESEARCH 18

3. STUDY OBJECTIVES **19**

3.1. PRIMARY OBJECTIVE 19
3.2. SECONDARY OBJECTIVES 19

4. DESCRIPTION OF THE STUDY **19**

4.1. TYPE OF STUDY 19
4.2. RESEARCH CATEGORY 20

5. STUDY POPULATION **20**

5.1. INCLUSION CRITERIA 21
5.2. NON-INCLUSION CRITERIA 21
5.3. PROCEDURE FOR PREMATURE TREATMENT DISCONTINUATION 22
5.4. EXCLUSION PERIOD AND PARTICIPATION IN ANOTHER STUDY 22
5.5. COMPENSATION OF SUBJECTS 22
5.6. RECRUITMENT MODALITIES 22

6. STUDY METHODOLOGY **23**

6.1. MEDICAL EVALUATION CRITERIA 23
6.1.1. Primary endpoint *23*
6.1.2. Secondary endpoints *23*
6.2. DESCRIPTION OF STUDY METHODOLOGY 23
6.3. DESCRIPTION OF THE MEASURES TAKEN TO REDUCE AND AVOID BIAS 26
6.3.1. Justification of randomization arms *26*
6.3.2. Blinding bias *26*

6.3.3. Randomization ... 27
6.3.4. Reproducibility of the assay ... 27
6.3.5. Control of attrition bias ... 27

7. PRACTICAL CONDUCT OF THE STUDY ... 28

7.1. DETAILED DESCRIPTION OF PROCEDURES (DESCRIPTION OF EACH VISIT) ... 28
7.2. DESCRIPTION OF THE GENERAL LOGISTICAL ORGANIZATION OF THE STUDY ... 28
7.3. SAMPLES AND BIOLOGICAL ANALYSES ... 29
7.4. PLANNED DURATION OF PARTICIPATION AND STUDY TIME FRAME ... 29

8. STUDY PRODUCT ... 30

8.1. DESCRIPTION OF STUDY PRODUCT ... 30
8.2. PRESENTATION OF THE PRODUCTS ... 30
8.3. USE OF THE PRODUCT ... 30
8.4. AUTHORIZED AND UNAUTHORIZED MEDICINES AND TREATMENTS DURING THE STUDY ... 30

9. DATA COLLECTED ... 30

10. STATISTICAL CONSIDERATIONS ... 31

10.1. SAMPLE SIZE ESTIMATION ... 32
10.2. DATA ANALYSIS: GENERAL POINTS ... 33
10.3. CHARACTERISTICS OF PATIENTS AT BASELINE ... 33
10.4. PRIMARY ANALYSIS ... 33
10.5. SECONDARY ANALYSES ... 33
10.6. ECONOMIC ANALYSES ... 34
10.7. METHOD FOR MANAGING MISSING, UNUSED OR INVALID DATA ... 34
10.8. PERSONS IN CHARGE OF ANALYSIS ... 34

11. SAFETY ASSESSMENT – MANAGEMENT OF ADVERSE EVENTS ... 35

11.1. DEFINITIONS ... 35
11.2. SERIOUS ADVERSE EVENT REPORTING ... 36
11.3. MONITORING COMMITTEE ... 37
11.4. TERMINATION OF THE STUDY ... 38
11.5. FOLLOW-UP OF PATIENTS PRESENTING AN ADVERSE EVENT ... 38

12. RIGHT OF ACCESS TO SOURCE DOCUMENTS AND DATA ... 38

12.1. ACCESS TO DATA ... 38
12.2. SOURCE DATA ... 38
12.3. DATA CONFIDENTIALITY ... 38
12.4. REGISTRATION IN THE NATIONAL FILE OF BIOMEDICAL RESEARCH SUBJECTS ... 39

13. QUALITY CONTROL AND ASSURANCE ... 39

13.1. ENGAGEMENT OF THE INVESTIGATORS AND THE SPONSOR ... 39
13.2. QUALITY ASSURANCE ... 39
13.3. QUALITY CONTROL ... 39
13.4. CASE REPORT FORM ... 40

14. ETHICAL CONSIDERATIONS ... 40

14.1. ETHICS COMMITTEE ... 40
14.2. INFORMATION FOR PATIENTS AND WRITTEN INFORMED CONSENT FORM ... 40
14.3. PROTOCOL AMENDMENTS ... 41
14.4. MANAGEMENT RELATING TO THE RESEARCH ... 41

15. DATA PROCESSING AND STORAGE OF STUDY DOCUMENTS AND DATA ... 41

15.1. DATA ENTRY AND PROCESSING 41
15.2. CNIL 41
15.3. RECORD-KEEPING 41
16. FUNDING AND INSURANCE 42
16.1. STUDY BUDGET 42
16.2. INSURANCE 42
17. COMMUNICATION – RULES FOR PUBLICATION 42
18. FEASIBILITY OF THE STUDY 43
19. COMMENTS AND ANSWERS TO PREVIOUS EXAMINATIONS 43
20. BIBLIOGRAPHY 47
21. LIST OF APPENDIX 49

Abbreviations

ANSM: Agence Nationale de Sécurité du Médicament et des Produits de santé *(French Agency for the Safety of Medicines and Health Products)*

CCT-scan: cranial computed tomography

CNIL: Commission Nationale de l'Informatique et des Libertés *(French data protection authority)*

CT-scan: computed tomography

CRA: Clinical Research Associate

CRT: Clinical Research Technician

CSP: Code de la Santé Publique *(French Public Health Code)*

CST: Clinical Study Technician

CV: Coefficient of variation

DRCI: Délégation à la Recherche Clinique et à l'Innovation *(Clinical Research and Innovation Department)*

eCRF: electronic Case Report Form

GCS: Glasgow Coma Scale

kDa: kiloDalton

mGy.cm: milliGray.centimeter

mTBI: mild Traumatic Brain Injury

mSv: milliSievert

SAE: serious adverse events

SD: Standard deviation

SFMU: Société Française de Médecine d'Urgence *(French Society of Emergency Medicine)*

SFP: French Society of Pediatry

TBI: Traumatic Brain Injury

1. General information

1.1. Title of the research

Interventional study assessing evaluation of the interest of serum S100B protein determination in the management of pediatric mild traumatic brain injury

Running title: PROS100B

1.2. Sponsor code

Sponsor Code : PHRC N 2015 BOUVIER
N°IDRCB : 2016-A00195-46

1.3. Sponsor

Clermont-Ferrand Teaching Hospital
58 rue Montalembert
63003 Clermont-Ferrand cedex 1
France

Associate Directorate General – Clinical Research and Innovation Department
Tel: +33 4 73 75 11 95 Fax: +33 4 73 75 47 30

1.4. Coordination and monitoring

Clermont-Ferrand Teaching Hospital
58 rue Montalembert
63003 Clermont-Ferrand cedex 1
France

Associate Directorate General – Clinical Research and Innovation Department
Tel: +33 4 73 75 11 95 Fax: +33 4 73 75 47 30

1.5. Investigators

1.5.1. Coordinating investigator and collaborator

Coordinating investigator: ***Damien Bouvier (Physician-Biologist, Hosp.Practitioner assistant)***
Medical Biochemistry and Molecular Biology Clermont-Ferrand Teaching Hospital
58, Rue Montalembert 63003 Clermont-Ferrand cedex 1 France
Tel: +33 4 73 75 18 01 Fax: +33 4 73 75 18 55 dbouvier@chu-clermontferrand.fr

Co-investigator: *Prof. André Labbé (Pediatrician, Univ. Prof./Hosp. Practitioner, dept. head)*
Pediatrics Unit Estaing Hospital Clermont-Ferrand Teaching Hospital
Place Lucie et Raymond Aubrac 63003 Clermont-Ferrand cedex 1 France
Tel: +33 4 73 75 00 28 Fax: +33 4 73 75 06 09 alabbe@chu-clermontferrand.fr

Scientific collaborator: *Prof. Vincent Sapin (Biologist, Univ. Prof./Hosp. Practitioner, dept. head)*
Medical Biochemistry and Molecular Biology Clermont-Ferrand Teaching Hospital
58, Rue Montalembert 63003 Clermont-Ferrand cedex 1 France
Tel: +33 4 73 75 18 01 Fax: +33 4 73 75 18 55 vsapin@chu-clermontferrand.fr

1.5.2. Scientific collaborators and Co-investigators

Center	Biochemists (Scientific collaborators)	Pediatricians (Co-investigators)
**Limoges Teaching hospital**	**Prof. Franck Sturtz, Univ. Prof./Hospital Practitioner**<br><br>Biochemistry deparment<br>Hôpital Dupuytren<br>Limoges teaching Hospital<br>2, Avenue Martin Luther King<br>87042 Limoges cedex, France<br><br>Tel: +33 5 55 05 63 41 / +33 5 55 05 80 82<br>franck.sturtz@unilim.fr	**Dr. Dallocchio Aymeric, Hospital Practitioner**<br><br>Hôpital de la mère et de l'enfant<br>8, avenue Dominique Larrey<br>87042 Limoges cedex, France<br><br>Tel : +33 5 55 05 64 61<br><br>Aymeric.Dallocchio@chu-limoges.fr
**Hospices Civils de Lyon**	**Dr. Régine Cartier, Hospital Practitioner**<br><br>Groupement Hospitalier Est<br>Biology and Pathology Center East<br>59, boulevard Pinel<br>69677 Bron Cedex, France<br><br>Tel: +33 4 72 35 71 90<br>regine.cartier@chu-lyon.fr	**Prof. Yves Gillet, Hospital Practitioner**<br><br>Groupement Hospitalier Est<br>Maternity and Paediatric Hospital<br>Pediatric Emergency Admissions<br>59, boulevard Pinel<br>69677 Bron Cedex, France<br><br>Tel: +33 4 27 85 56 34/+33 4 27 85 56 42<br>yves.gillet@chu-lyon.fr
**Assistance Publique des Hôpitaux de Marseille**	**Prof. Régis Guieu, Univ. Prof./Hospital Practitioner**<br>Department Head<br><br>Biochemistry Laboratory<br>Hôpital de la Timone<br>Boulevard Jean Moulin<br>13005 Marseille, France<br><br>Tel: +33 4 91 38 56 50<br>regis-pierre.guieu@ap-hm.fr	**Prof. Franck Launay, Univ. Prof./Hospital Practitioner**<br>Department Head<br><br>Pediatric Emergencies<br>Hôpital Timone Enfants<br>264 rue St Pierre<br>13385 Marseille cedex 5, France<br><br>Tel: +33 4 91 38 66 52<br>Franck.LAUNAY@ap-hm.fr
**Montpellier Teaching hopsital**	**Prof. Cristol Jean Paul, Univ. Prof./Hospital Practitioner**<br>Biochemistry and hormonology department<br>Montpellier Teaching hospital – Lapeyronie<br>371 Avenue du Doyen Gaston Giraud<br>34295 Montpellier Cedex 5<br><br>Tel: +33 4 67 33 83 14<br>jp-cristol@chu-montpellier.fr	**Dr Jullian Chloé, Hospital Assistante**<br>Pediatric emergency department<br>Montpellier Teaching hospital– Lapeyronie<br>371 Avenue du Doyen Gaston Giraud<br>34295 Montpellier Cedex 5<br><br>Tel:+33 4 67 33 22 86<br><br>chloe-jullian@chu-montpellier.fr

Center	Biochemists (Scientific collaborators)	Pediatricians (Co-investigators)
**Nice**<br>**Teaching Hospital**	**Dr. Pascale Bayer, Hospital Practitioner**<br><br>Biology Laboratory<br>Hôpital Pasteur<br>30, avenue de la voie Romaine<br>CS 51069<br>06200 Nice cedex 1, France<br><br>Tel: +33 4 92 03 81 63<br>bayer.p@chu-nice.fr	**Dr. Hervé Haas, Hospital Practitioner**<br><br>Pediatric Emergencies<br>Lenval Teaching Hospital<br>57, Avenue de la Californie<br>06200 Nice, France<br><br>Tel: +33 4 92 03 05 79<br>haas.h@pediatrie-chulenval-nice.fr
**Reims**<br>**Teaching Hospital**	**Dr. Jean Baptiste Oudart, Univ. And Hospital Practitioner**<br><br>Biochemistry Laboratory<br>Hôpital Robert Debré<br>Reims Teaching Hospital<br>Avenue du Général Koenig<br>51092 Reims Cedex, France<br><br>Tel: +33 3 10 73 62 87 / +33 3 26 78 83 46<br>joudart@chu-reims.fr	**Dr. Yannick Plenier, Hospital Practitioner**<br><br>Pediatrics Emergencies<br>Reims Teaching Hospital<br>45, rue Cognacq- Jay<br>51092 Reims Cedex, France<br><br>Tel: +33 3 26 78 89 92<br>yplenier@chu-reims.fr
**Saint-Etienne**<br>**Teaching hospital**	**Prof. Gonzalo Philippe, Univ. Prof./Hospital Practitioner**<br><br>Biologie-Pathologie departement<br>Hôpital Nord<br>Saint Etienne Teaching Hopsital<br>Avenue Albert Raimond<br>42055 Saint-Étienne cedex 2, France<br><br>Tel : +33 4 77 12 75 53<br>philippe.gonzalo@chu-st-etienne.fr	**Dr. Mory Olivier, Hospital Practitioner**<br><br>Pediatrics- Medical/Surgical Emergencies<br>Hôpital Nord<br>Saint Etienne Teaching Hopsital<br>Avenue Albert Raimond<br>42055 Saint-Étienne cedex 2, France<br><br>Tel:+33 4 77 82 81 34/+33 4 77 82 86 32<br>olivier.mory@chu-st-etienne.fr
**Nîmes**<br>**Teaching Hospital**	**Dr. De Brauwere David-Paul, Hospital Practitioner**<br><br>Biochemistry and Molecular Biology Laboratory<br>Hôpital Caremeau<br>Nimes Teaching Hospital<br>Place Pr. Robert Debre<br>30029 Nimes cedex, France<br><br>Tel: +33 4 66 68 32 07<br>david.paul.de.brauwere@chu-nimes.fr	**Dr. Fournier Philippe, Hospital Practitioner**<br><br>Pediatric Emergencies<br>Nîmes Teaching Hospital<br>Place du professeur Debré<br>30029 Nîmes cedex 09, France<br><br>Tel: +33 4 66 68 43 21<br>philippe.fournier@chu-nimes.fr

**Vichy Teaching Hospital**	**Dr Aubailly Lucie, Hospital Practioner**<br><br>Biochemestry Laboratory<br>Centre Hospitalier Jacques Lacarin<br>Boulevard Denière<br>BP 2757<br>03207 Vichy cedex, France<br><br>Tel : +33 4 70 97 13 80<br>Lucie.Aubailly@ch-vichy.fr	**Dr David Dall'Acqua, Hospital Practioner**<br><br>Pediatrics Emergencies<br>Centre Hospitalier Jacques Lacarin<br>Boulevard Denière<br>BP 2757<br>03207 Vichy cedex, France<br><br>Tel : +33 4 70 97 33 07<br>David.DallAcqua@ch-vichy.fr
**Nantes Teaching Hospital**	**Pr Damien MASSON, Hospital Practioner**<br><br>Biochemestry Laboratory<br>Nantes Teaching Hospital<br>Hôtel-Dieu<br>1 place Alexis-Ricordeau<br>44093 NANTES Cedex 01<br><br>Tel : +33 2 40 08 33 33<br>damien.masson@chu-nantes.fr	**Pr Christele Gras-Le Guen, Hospital Practioner**<br><br>Pediatrics Emergencies<br>Nantes Teaching Hospital<br>Hôpital Mère Enfant<br>38 boulevard Jean Monnet<br>44093 NANTES Cedex 01<br><br>Tel : +33 2 40 08 34 83<br>christele.grasleguen@chu-nantes.fr

1.6. Associate Partners

Methodologist: ***Bruno Pereira (PhD)***
Clinical Research and Innovation Department Clermont-Ferrand Teaching Hospital
Villa annexe IFSI 58, rue Montalembert 63003 Clermont-Ferrand cedex 1 France
Tel: +33 4 73 75 49 64 Fax: +33 4 73 75 47 30 bpereira@chu-clermontferrand.fr

Economist: *Charline Mourgues (MSc)*
Clinical Research and Innovation Department Clermont-Ferrand Teaching Hospital
Villa annexe IFSI 58, rue Montalembert 63003 Clermont-Ferrand cedex 1 France
Tel: +33 4 73 75 03 50 c_mourgues@chu-clermontferrand.fr

Project Leader: *David Balayssac (PhD, PharmD)*
Clinical Research and Innovation Department Clermont-Ferrand Teaching Hospital
Villa annexe IFSI 58, rue Montalembert 63003 Clermont-Ferrand cedex 1 France
Tel: +33 4 73 75 10 28 Fax: +33 4 73 75 47 30 dbalayssac@chu-clermontferrand.fr

1.7. Study sites

Center		Departments
**1**	**Clermont-Ferrand Teaching Hospital**	Pediatric Emergencies<br>Estaing Hospital<br>Place Lucie et Raymond Aubrac<br>63000 Clermont-Ferrand, France
**2**	**Limoges Teaching hospital**	Hôpital de la mère et de l'enfant<br>8, avenue Dominique Larrey<br>87042 Limoges cedex, France
**3**	**Hospices Civils de Lyon**	Groupement Hospitalier Est<br>Maternity and Paediatric Hospital<br>Pediatric Emergency Admissions<br>59, boulevard Pinel<br>69677 Bron Cedex, France
**4**	**Assistance Publique des Hôpitaux de Marseille**	Pediatric Emergencies<br>Hôpital Timone Enfants<br>264 rue St Pierre<br>13385 Marseille cedex 5, France
**5**	**Montpellier Teaching hopsital**	Pediatric emergency department<br>191, Avenue du Doyen Gaston Giraud<br>34090 Montpellier, France
**6**	**Nice Teaching Hospital**	Pediatric Emergencies<br>Lenval Teaching Hospital<br>57, Avenue de la Californie<br>06200 Nice, France
**7**	**Reims Teaching Hospital**	Pediatrics Emergencies<br>Reims Teaching Hospital<br>45, rue Cognacq- Jay<br>51092 Reims Cedex, France
**8**	**Saint-Etienne Teaching hospital**	Pediatrics- Medical/Surgical Emergencies<br>Hôpital Nord<br>Saint Etienne Teaching Hopsital<br>Avenue Albert Raimond<br>42055 Saint-Étienne cedex 2, France
**9**	**Nîmes Teaching Hospital**	Pediatrics Emergencies<br>Nîmes Teaching Hospital<br>Place du Professeur Debré<br>30029 Nîmes Cedex 09, France
**10**	**Vichy Teaching Hospital**	Pediatrics Emergencies<br>Centre Hospitalier Jacques Lacarin

		Boulevard Denière<br>BP 2757<br>03207 Vichy cedex, France
**11**	**Nantes Teaching Hospital**	Pediatrics Emergencies<br>Nantes Teaching Hospital<br>Hôpital Mère Enfant<br>38 boulevard Jean Monnet<br>44093 NANTES Cedex 01

1.8. Data processing

Clinical Research and Innovation Delegation
Clermont-Ferrand Teaching Hospital
Villa annexe IFSI
58, rue Montalembert
63003 Clermont-Ferrand cedex 1, France

1.9. Ethics Committee

Southeast Ethics Committee VI (IRB: 00008526)
Clermont-Ferrand Teaching Hospital
Administration centrale
BP 69
63003 Clermont-Ferrand Cedex 1, France

1.10. Estimated time frame

Ethics Committee submission: April 2016
ANSM authorization: June 2016
Study start: July 2016
Inclusion period: July 2016 – October 2021 (60 months)
Estimated end of study: November 2021
Final study report: October 2022

2. Study rationale / Scientific justification

2.1. Current state of scientific knowledge

Pediatric traumatic brain injury (TBI) can be classified into three categories – severe, moderate and mild – each of which requires a specific treatment algorithm, as recently restated by the French Society of Emergency Medicine (SFMU) (Jehlé *et al.*, 2012) and the French Society of Pediatry (SFP) (Lorton *et al.*, 2014). Severe and moderate TBI, with a Glasgow Coma Scale (GCS) score <8 or between 8 and 12, respectively, require hospitalization or transfer to the resuscitation bay for severe TBI. In pediatric patients, the management of mild traumatic brain injury (mTBI), defined by a GCS score between 13 and 15, is much more difficult than in adults, due to the type of patient. mTBI accounts for around 80 to 90% of all cases of pediatric TBI presenting to emergency rooms, and domestic accidents are the most common cause (75%). Common post-mTBI symptoms and deficits in children are not specific to mTBI and appear to resolve with time; however, limited evidence suggests that children with intracranial pathology on imaging may

experience persisting symptoms or deficits (Hung *et al.*, 2014). Therefore, cranial computed tomography (CCT-scan) scans and short inpatient observation are very frequently ordered so as to be sure not to overlook any complications, which are known to occur at a rate of 0-7% (American Academy of Pediatrics, 1999). Yet, it has been very clearly established that 93% of these measures (CCT-scan and/or short hospitalization) are ultimately unnecessary (Homer and Kleinman, 1999). Reducing the number of unnecessary CCT-scans in pediatric patients is all the more important because several recent large-scale epidemiological studies have described a link between radiation exposure from CT-scans performed during childhood (effective dose 0.03 to 69.2 mSv per scan) and the risk of cancer (Pearce *et al.,* 2012; Mathews *et al.*, 2013; Miglioretti *et al.*, 2013). Indeed, iatrogenic radiation with a cumulative dose of 50 mGy may triple the risk of leukemia, and cumulative doses of 60 mGy could triple the risk of brain tumors (Pearce *et al.,* 2012). Moreover, an Australian study of 11 million children found a 24% increase in cancer risk for the 680,000 children who underwent CT-scan (including 59% CCT-scans), and a 35% increase for the 1-4 year-old age group. This study also noted that the risk of brain tumors was 2.44 times higher for children who had CCT-scan (3.24 times for the 1-4 year-old age group) (Mathews *et al.*, 2013).

The same issue arises in France, based on a recent analysis by the radiology department at Estaing Teaching Hospital in Clermont-Ferrand showing that the mean radiation exposure from a pediatric CCT-scan is approximately 400 mGy.cm (or an effective dose of 1.6 mSv). In this context, the S100B protein stands out as a relevant biomarker that could potentially be of great interest for reducing unnecessary CCT-scans and hospitalizations which form the cornerstone of the current recommendations for the management of mTBI (Jehlé *et al.,* 2012; Lorton *et al.*, 2014). Lorton et al. mention this perspective.

The S100B protein is a 21 kDa dimeric holoprotein discovered in 1965 by Moore during an electrophoresis study of protein extracts from human and animal brains. The name “S100 protein” derives from its solubility in a saturated (100%) ammonium sulfate solution (Beaudeux *et al.,* 1999). Its utility in clinical biology is related to its release into the extracellular medium either during overexpression of its gene (trisomy 21, Alzheimer’s disease, Creutzfeldt-Jakob disease, multiple sclerosis, melanoma) or in association with vascular or traumatic brain injury (Beaudeux *et al.,* 2001 and 2002). The protein can be detected in serum; it is eliminated by the kidneys with a half-life of around 2 hours (Anderson *et al.,* 2001). In the medical laboratory, determination of serum levels of S100B protein is of real interest mainly in two types of pathologies: meningeal hemorrhage (Sanchez-Peña *et al.,* 2008) and TBI.

The utility of serum S100B determination for reducing unnecessary CCT-scans in the management of mTBI in adults has been well established in many studies and recently confirmed in a meta-analysis (Undén and Romner, 2010). With a serum cutoff threshold of 0.1 µg/L, the S100B protein identifies patients with positive CCT-scan lesions with a sensitivity of 100% and a specificity of approximately 30%. Since the assay must be carried out within 3 hours following the trauma (due to the half-life of the biomarker), it also appears to shorten the duration of patient management compared to CCT-scan, which is generally performed 6 hours post-trauma. In this same meta-analysis published in 2010, the authors noted a lack of convincing data in pediatric populations. Indeed, the interest of S100B in pediatric mTBI is more difficult to evaluate due to physiological variations in serum S100B concentrations, especially during the first 3 years of life (Bouvier *et al.,* 2011).

It is in this context that the Medical Biochemistry and Pediatrics departments of Clermont-Ferrand Hospital carried out a single-center, prospective study on the interest of serum S100B determination in the management of pediatric mTBI. The primary objective of this study was to establish reference intervals for children under 3 years old, since such data were not available in the literature at the time. To define these reference ranges, 186 healthy controls aged 0-3 years were analyzed (Bouvier *et al.,* 2011), yielding specific values for pediatric patients that differed from those in adults. Four age groups were defined to establish pediatric reference intervals for S100B protein (95th percentile), as follows: 0 to 3 months: 0.62 µg/L; 4 to 9 months: 0.35 µg/L; 10 to 24 months: 0.23 µg/L; and >25 months: 0.18 µg/L. Given the large standard deviation for the 0-3 month age group, it was decided to create a single age group of 0 to 9 months with a cutoff of 0.35 µg/L. In the group of children under 2 years old, serum S100B concentrations are inversely proportional to age ($r = -0.60$, $p<0.001$) with no gender-related differences. We also showed that serum S100B levels are inversely correlated with predicted head circumference according to the equation: serum S100B concentration (µg/L) = -1.884 x head circumference (meters) + 1.0455 ($r^2 = -0.96$, $p<0.001$) (Bouvier *et al.,* 2011). The secondary objective was to determine if there was a significant difference in serum S100B levels according to the severity of the TBI (mild, moderate or severe). This also yielded an idea of the discriminatory power of this protein with regards to the decision to order clinical observation or computed tomography during the conventional management of mTBI. To this end, 446 patients aged 0-18 years with TBI were prospectively enrolled in a 1-year study. A blood sample was taken within 3 hours post-trauma to assay S100B protein (Bouvier *et al.,* 2012). Serum S100B protein levels were found to increase significantly according to the severity of TBI (severe > moderate > mild). Of the 65 patients who underwent CCT-scan, 23 had positive findings (CCT+). In these pediatric patients, measurement of S100B identified patients correctly as CCT+ with a sensitivity of 100% and a specificity of 33%. These findings are in line with data obtained in adults (Unden and Romner, 2010) and with an Austrian study in 109 children (Castellani *et al*., 2009). Furthermore, in the Clermont-Ferrand study, serum S100B determination was shown to be a discriminatory test which correctly identified 21 patients as having an unfavorable clinical evolution following mTBI with a sensitivity of 100% and a negative predictive value of 100%. Lastly, of the 242 hospitalizations, 81 could have been avoided by including the S100B concentration in the decision-making algorithm. After establishing the specific reference ranges for S100B in children under the age of 3 years, we showed that S100B assay could theoretically reduce the number of CCT-scans by 33%, thereby reducing radiation exposure, and could also reduce unnecessary hospitalizations by 33%, thereby saving costs related to the current practices for managing pediatric mTBI (Bouvier *et al.,* 2012). Recent studies confirm these results (Manzano et al., 2015; Simon-Pimmel *et al.*, 2015).

2.2. Hypotheses and objectives

Based on these initial results from a prospective study, a multicenter interventional study will be necessary to validate the routine use of this biomarker (Bouvier, 2013). The ultimate goal is to include serum S100B assay in the current recommendations for mTBI management based on the study of Kuppermann *et al*. (2009), as mTBI accounts for 5-8% of pediatric emergency admissions in France (60-100 per 100,000 children). The study of Kuppermann *et al.* strongly dictated the recommandations for mTBI management by the French Society of Emergency

Medicine (SFMU) (Jehlé *et al.*, 2012). Then, from these 2 publications, the French Society of Pediatrics (SFP) redacted their recommendations after adjustment method recommendations for clinical practice, used by the French High Authority of Health (HAS) (Lorton *et al.*, 2014).

The use of serum S100B assay as part of the management of pediatric mTBI should make it possible to reduce the number of additional examinations, in particular a 30% reduction in the number of CCT-scans, with a resultant reduction in radiation exposure, known to be a risk factor for cancer (Pearce *et al*., 2012; Mathews *et al.*, 2013; Miglioretti *et al.*, 2013).

2.3. Summary of the benefits and foreseeable and known risks for subjects participating in the research

Determination of serum S100B protein in pediatric mTBI has a sensitivity of 100% (a positive result is associated with intracerebral lesions on CCT-scan and/or development of clinical complications) and a negative predictive value of 100% (a negative result is associated with absence of intracerebral lesions and/or development of clinical complications) (Table 1) (Bouvier *et al.,* 2012). Thus, the benefit to the patient consists in a simplified course of management compared with the conventional approach. The risks to the patient are minimal since the probability of a false negative result is virtually nul. Furthermore, the blood assay of S100B protein is minimally invasive because it requires just a single blood sampling using a micro-method.

	**Sensitivity**	**Specificity**	**Positive predictive value**	**Negative predictive value**
**Positive CCT-scan**	100% (82.5 - 100%)	33% (20 - 50%)	45% (31 - 60%)	100% (77 – 100%)
**Unfavorable clinical evolution**	100% (84 - 100%)	36% (31 - 41%)	8% (5 - 11%)	100% (97 – 100%)

Table 1: Characteristics of the serum S100B assay relative to a positive CCT-scan or an unfavorable clinical evolution in pediatric mTBI (Bouvier *et al.,* 2012) (95% confidence interval in parentheses).

2.4. Expected impact

Inclusion of serum S100B determination in the diagnostic algorithm of mTBI in pediatric patients has the potential to avoid unnecessary tests such as CCT-scans. This reduction in the number of CCT-scans performed is expected at the end of the study and would result in a reduction of exposure of the children to iatrogenic radiation.

This study will be the first multicenter study and will establish, promote and convey a new management strategy for pediatric mTBI.

In view of the literature regarding the serum S100B assay in children (no multicenter randomized study to date) and considering those for adults (Undén *et al.,* 2010), a randomized clinical trial ("conventional management" *versus* "S100B management") will assess the main objective and should lead to an international publication with a high impact factor.

2.5. References to the scientific literature and to pertinent data serving as a basis for the research

Biberthaler P, Linsenmeier U, Pfeifer KJ, Kroetz M, Mussack T, Kanz KG, Hoecherl EF, Jonas F, Marzi I, Leucht P, Jochum M, Mutschler W. Serum S-100B concentration provides additional information for the indication of computed tomography in patients after mild head injury: a prospective multicenter study. Shock. 2006 May; 25(5):446-53.

Bouvier D, Fournier M, Dauphin JB, Amat F, Ughetto S, Labbé A, Sapin V. Serum S100B determination in the management of pediatric mild traumatic brain injury. Clin Chem. 2012 Jul; 58(7):1116-22.

Bouvier D, Castellani C, Fournier M, Dauphin JB, Ughetto S, Breton M, Labbé A, Weinberg AM, Sapin V. Reference ranges for serum S100B protein during the first three years of life. Clin Biochem. 2011 Jul;44(10-11):927-9.

Calcagnile O, Undén L, Undén J. Clinical validation of S100B use in management of mild head injury. BMC Emerg Med. 2012 Oct 27;12(1):13.

Kuppermann N, Holmes JF, Dayan PS, Hoyle JD, Atabaki SM, Holubkov R et al. Identification of children at very low risk of clinically-important brain injuries after head trauma: a prospective cohort study. Lancet. 2009;374(9696):1160-70.

Mathews JD, Forsythe AV, Brady Z, Butler MW, Goergen SK, Byrnes GB, Giles GG, Wallace AB, Anderson PR, Guiver TA, McGale P, Cain TM, Dowty JG, Bickerstaffe AC, Darby SC. Cancer risk in 680,000 people exposed to computed tomography scans in childhood or adolescence: data linkage study of 11 million Australians. BMJ. 2013 May 21;346:f2360.

Undén J, Romner B. Can low serum levels of S100B predict normal CT findings after mild head injury in adults?: an evidence-based review and meta-analysis. J Head Trauma Rehabil. 2010 Jul-Aug; 25(4):228-40.

3. Study objectives

3.1. Primary objective

The primary objective is to evaluate the utility of serum S100B measurement in the management of pediatric mTBI by demonstrating a decrease in the proportion of CCT-scans prescribed in the "S100B management" intervention arm compared with the "conventional management" control arm, hypothesizing a 30% decrease in the number of CCT-scans between the intervention and control arms.

3.2. Secondary objectives

Secondary objectives are to demonstrate the utility of serum S100B measurement with respect to:

- Reduction in the time spent in the pediatric emergency room
- Reduction in the duration of hospitalization
- Reduction in radiation exposure
- Reduction in sedation and use of sedatives
- Detection of complications (intracranial lesions) by CCT-scan which can occur at a rate of 0-7% in patients with mTBI (American Academy of Pediatrics, 1999)
- Absence of intercurrent events at 48 hours and 3 weeks after mTBI
- Compliance of emergency physicians with the S100B assay
- Reduction of the cost of management

4. Description of the study

4.1. Type of study

The study protocol corresponds to a diagnostic prospective, controlled, multicenter study using a stepped wedge cluster design, in which pediatric patients (aged ≤ 16 years) presenting to the pediatric emergency room for mTBI with a GCS score of 15 will benefit from usual care ("conventional management" arm) in the control group, and from S100B management in the interventional group.

A recent systematic review (Mdege *et al.*, 2011) indicates stepped wedge cluster randomized design is particularly used to evaluate interventions during routine implementation, particularly for interventions that have been shown to be effective in more controlled research settings, or where there is lack of evidence of effectiveness but there is a strong belief that they will do more good than harm. Cluster randomized trials are often used to evaluate therapies or interventions in situations where individual randomization is not possible or not desirable for logistic (as in our study), financial or ethical reasons. A stepped wedge design is a type of crossover design in which different clusters cross over (switch treatments) at different time points. In addition, the clusters cross over in one direction only typically, from control to intervention. The first time point usually corresponds to a baseline measurement where none of the clusters receive the intervention of interest (figure 1). At baseline, all the patients will receive in this study the conventional management for mTBI. At subsequent time points, clusters initiate the intervention of interest, here the S100B management, and the response to the intervention is measured. More

than one cluster may start the intervention at a time point, but the time at which a cluster begins the intervention is randomized. Although the stepped wedge design extends the length of a randomized trial due to the presence of multiple time intervals, the nature of the design may be beneficial in certain settings. In a parallel or traditional crossover design, the intervention must be implemented in half of the total clusters simultaneously. The stepped wedge design allows the researcher to implement the intervention in a smaller fraction of the clusters at each time point. Another unique feature of the stepped wedge design is that the crossover is unidirectional. All clusters eventually receive the intervention and, in particular, the intervention is never removed once it has been implemented which may alleviate ethical and/or community concerns. This makes the stepped wedge design particularly useful for evaluating the population-level impact of an intervention that has been shown to be effective in an individually randomized trial. This point is essential in our study due to intuitive beliefs that the intervention is likely to do more good than harm. This should minimize the potential contamination bias.

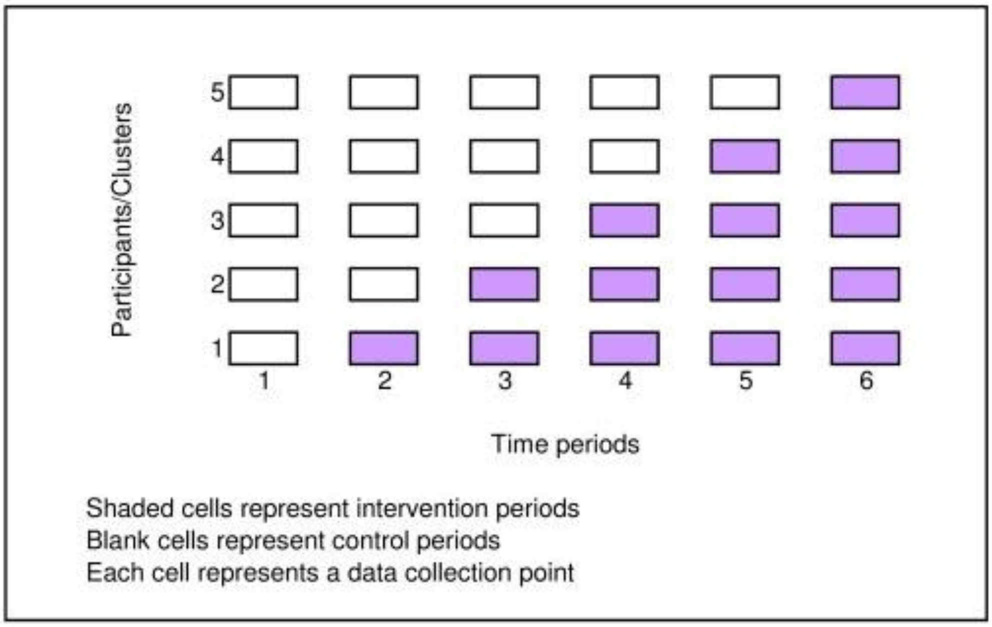

Figure 1: Stepped wedge study design of our trial. Fifteen intervals of 4 months will be fixed over 60 months. The randomization will involve 5 steps for which 2 (to 1) centers will be included in each cluster.

4.2. Research category

The study protocol will focus on the evaluation of a diagnostic procedure.

5. Study population

The study population comprises pediatric patients (aged ≤ 16 years) presenting to the pediatric emergency room for mTBI with a GCS score between 13 and 15 (Appendix 3). mTBI accounts for 5-8% of pediatric emergency admissions, i.e. 60-100 per 100,000 children (0-16 years: 12,000 emergency room visits per year in France) (Jehlé *et al.,* 2012).

5.1. Inclusion criteria

- Age ≤ 16 years
- Therapeutic management within 3 hours after TBI

GCS score of 15 classically requiring hospitalization and/or CCT-scan as per SFP recommendations (Lorton *et al.*, 2014). These criteria are:

- For children aged under 2 years (CCT-scan or hospitalization recommended according to physician's evaluation):
    - Parietal or occipital scalp hematoma,
    - Loss of consciousness for more than 5 seconds,
    - Trauma due to serious accident (road accident with passenger ejected from vehicle or death of another person or rollover; pedestrian hit by a moving vehicle; cyclist not wearing a helmet; fall from a height greater than 0.9 meter),
    - Abnormal behavior in the opinion of parents.
- For children 2 years and older (CCT-scan or hospitalization recommended according to physician's evaluation):
    - Loss of consciousness at time of accident,
    - Vomiting,
    - Trauma due to serious accident (road accident with passenger ejected from vehicle or death of another person or rollover; pedestrian hit by a moving vehicle; cyclist not wearing a helmet; fall from a height greater than 1.5 meter),
    - Severe headache.

5.2. Non-inclusion criteria

Patient already enrolled in another therapeutic trial with drug administration

Down syndrome

Melanoma

Refusal of child

Refusal of parents or legal guardian

Trauma more than 3 hours earlier

GCS score of 13 or 14, or signs of skull fracture or lesions of the skull base (CCT-scan recommended)

Children with TBI not requiring hospitalization and/or CCT-scan as per SFP recommendations (Lorton *et al.*, 2014). This group is defined by the absence of the following criteria:

- GCS score different from 15,
- Age < 3 months,
- Seriousness of accident:
    - road accident with passenger ejected from vehicle or death of another person or rollover,
    - pedestrian hit by a moving vehicle,
    - cyclist not wearing a helmet.

- Fall:
  - of more than 0.9 m before age 2 years,
  - of more than 1.5 m after age 2 years.
- Loss of consciousness for 5 seconds or more,
- Inconsolable crying,
- Agitation, drowsiness, feeling “slowed down”, obnubilation,
- Vomiting or headache,
- Facial or cranial hematoma,
- Otorrhea, rhinorrhea,
- Child under 2 years old,
- Loss of consciousness for less than 5 seconds,
- Unusual behaviour,
- Concern of family members.

5.3. Procedure for premature treatment discontinuation

The study can be stopped for the reasons identified in advance:

- Decision of the sponsor following new knowledge about the study.

In addition, a patient can withdraw from the study for the following reasons:

- Withdrawal of consent by the patient, parents or legal guardian,
- Non compliance of the patient with the stud,
- Serious events independent to the MTBi

5.4. Exclusion period and participation in another study

No enrolled patient can participate in another clinical research study during the duration of the present study.

No exclusion period for another clinical research study is planned outside the duration of the present study.

5.5. Compensation of subjects

The subjects will receive no compensation.

5.6. Recruitment modalities

The study will run over a period of 60 months at 11 participating hospital centers (see section 1.7). Patients will be enrolled in the study, in accordance with the selection criteria defined in sections 5.1 and 5.2, by the emergency doctor who will explain to the parents the interest of the study and the study procedures.

Both parents (or holders of parental authority) of each patient enrolled in the study will have to sign a consent form (Appendix 2) after receiving verbal and written information about the study and being given time to think it over that is compatible with the study procedure and without any hospital staff being present (Appendix 1). If one of the parents is absent and unable to come to the hospital, the consent form will be signed by only one parent. If both parents are

absent, the child will not be included in the study, since the trusted person accompanying the child does not have parental authority.

The possibility that the child will refuse to participate in the study is of course taken into account. Furthermore, the information notice and consent form will only be used if the child is in a condition to sign it and not overly anxious.

After obtaining consent (from the patient, parents or legal guardian), the patient will be included in the study.

6. Study methodology

6.1. Medical evaluation criteria

6.1.1. Primary endpoint

The primary endpoint is the proportion of CCT-scans prescribed (absence/presence of CCT-scan for each patient) within 48 hours following TBI, compared between the two arms ("S100B management" intervention arm *versus* "conventional management" control arm).

6.1.2. Secondary endpoints

Secondary endpoints are:

- Duration of management defined by the time spent in the pediatric emergency department (time between emergency room admission and discharge)
- Duration of hospitalization in another hospital department for observation
- Effective radiation dose (mSv) for each CCT-scan
- Sedation and quantity of sedatives prescribed
- Presence of intracranial injury on CCT-scan
- Presence of persistent clinical signs at the telephon follow-up interview 48 hours and 3 weeks after the mTBI (Appendix 4)
- Proportion of positive/negative CCT-scans in each arm
- Cost of management

6.2. Description of study methodology

The proposed protocol is a randomized, multicenter, open, prospective, interventional study (11 centers) using a stepped wedge cluster design, with two arms:

- Control group "Conventional Care": The children in the control group will have a conventional management treatment in accordance with the SFP recommendations (Lorton *et al.*, 2014) (Figure 2).

- Intervention group "S100B management": Patients in the "S100B management" intervention arm will have blood drawn for S100B determination within 3 hours after the trauma and their subsequent management will depend on the S100B assay results, which are available 1 hour after arrival at the laboratory (Bouvier et *al.,* 2012). The S100B serum assay will be considered positive for the following values according to age (Bouvier *et al.,* 2012):

0 to 9 months: > 0.35 µg/L,
9 to 24 months: > 0.23 µg/L,
Above 24 months: > 0.18 µg/L.

For a positive test, the children will have a conventional management treatment in accordance with the SFP recommendations (Lorton *et al.*, 2014). For a negative test, the children will be discharged from the emergency department **after 6 hours of observation** (Figure 3).

The presence of persistent clinical signs 48 hours and 3 weeks after the mTBI (telephone call) will be monitored for the two groups.

This stepped wedge cluster randomization (stratified by cluster size) was chosen 1) to improve feasibility in emergency department and 2) to avoid the major risk of contamination bias in the control group. The stepped wedge design provides an innovative choice for a cluster randomized crossover trial that is subject to constraints that limits the use more conventional designs. In our study, ethical objections arising from withholding an intervention anticipated to be beneficial is a motivation for employing a stepped wedge design. Considering the number of participating centers (n= 11) and the duration of this study (60 months), it was proposed to fix 12 intervals (time periods) of 4 months each one. Then, as it was proposed in Figure 1, the randomization will involve 5 steps for which 2 (or 1) centers will be included in each cluster.

Figure 2: Decision algorithm for CCT-scan or hospitalization indication for mTBI management in control group “conventional management”:

- ***For children < 2 years***

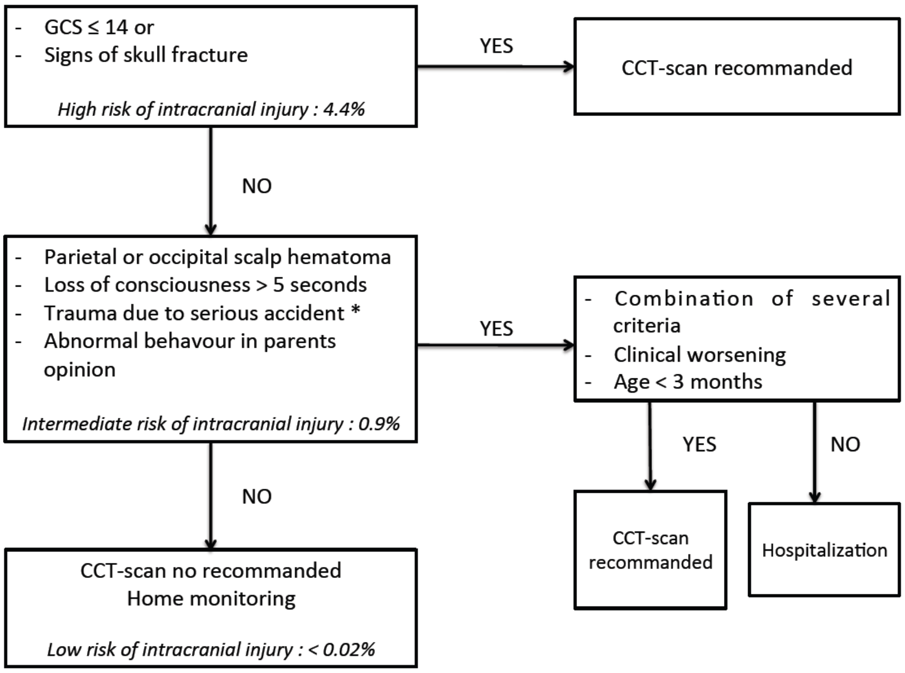

* road accident with passenger ejected from vehicle or death of another person or rollover; pedestrian hit by a moving vehicle; cyclist not wearing a helmet; fall from a height greater than 0.9 meter).

- ***For children ≥ 2 years***

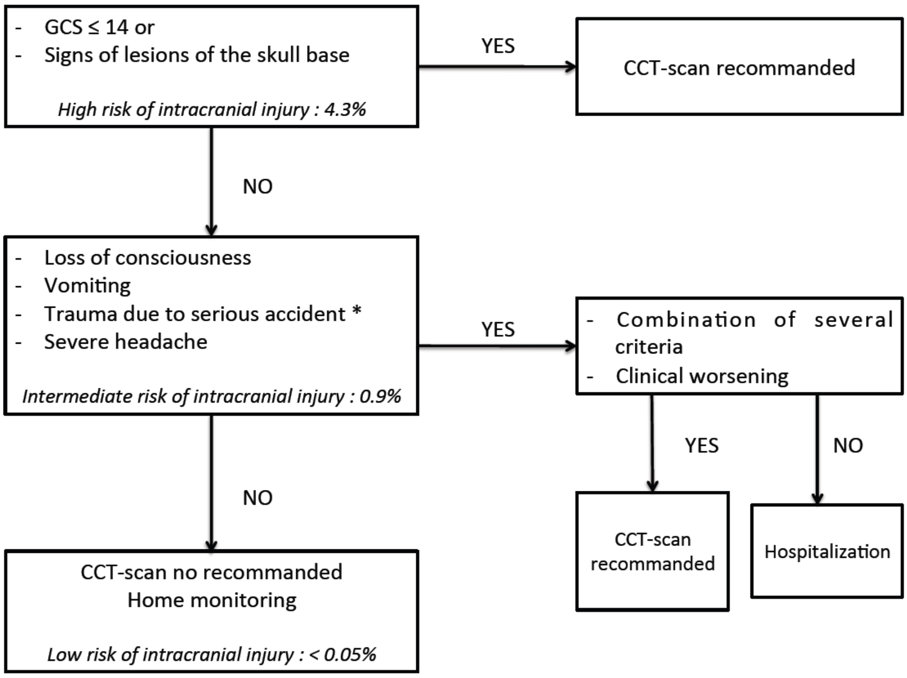

* road accident with passenger ejected from vehicle or death of another person or rollover; pedestrian hit by a moving vehicle; cyclist not wearing a helmet; fall from a height greater than 1.5 meter

Figure 3: Decision algorithm for CCT-scan or hospitalization indication for mTBI management in intervention group "S100B management":

- ***For children < 2 years***

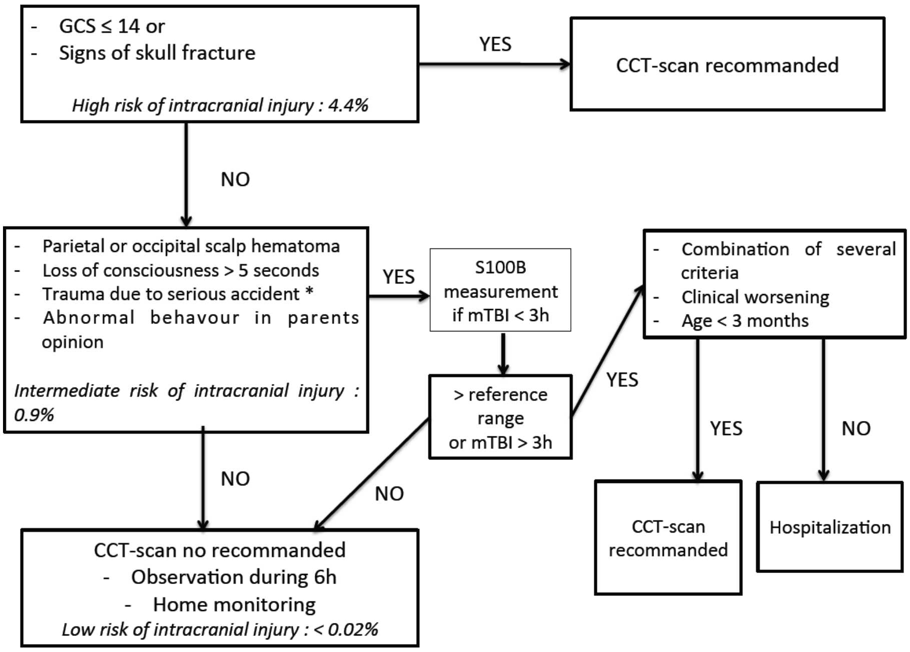

* road accident with passenger ejected from vehicle or death of another person or rollover; pedestrian hit by a moving vehicle; cyclist not wearing a helmet; fall from a height greater than 0.9 meter).

- ***For children ≥ 2 years***

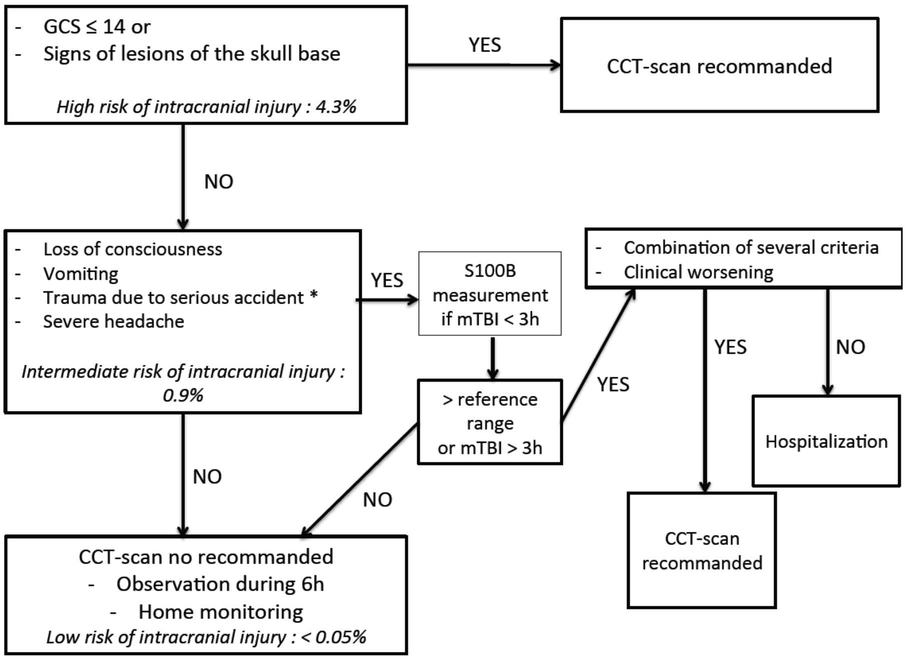

* road accident with passenger ejected from vehicle or death of another person or rollover; pedestrian hit by a moving vehicle; cyclist not wearing a helmet; fall from a height greater than 1.5 meter

6.3. Description of the measures taken to reduce and avoid bias

6.3.1. Justification of randomization arms

In view of the literature data on serum S100B protein determination in children (no randomized studies to date) and considering the data obtained in adults (Undén *et al.,* 2010), a stepped wedge cluster randomized design, with 2-arm study (“conventional management” *versus* “S100B management”) will allow us to evaluate the primary endpoint in the framework of a research protocol that could lead to an international publication with a Grade A recommendation.

Nevertheless, this study is intended to be pragmatic because in the “S100B management” arm, the pediatricians will still be able to not base their management on the S100B concentration. This aspect will be taken into account in the statistical considerations.

6.3.2. Blinding bias

The context in which the study is conducted precludes any blinding with respect to the patient, the pediatrician or the biologist. However, the radiologist who analyzes the CCT-scans will be blinded as to the arm in which the patient is enrolled.

The S100B assay results, prepared by the biologist, will be reported to the pediatrician in quantitative form with comments emphasizing the following points:

- The excellent negative predictive value of the biomarker when the measured serum S100B concentration is within the age-related reference interval (Bouvier *et al.,* 2012). The aim of this precaution is to promote a change in the strategy planned by the physician before the assay (i.e., to promote observation at home without CCT-scan or hospitalization).
- The low positive predictive value of the biomarker when the measured serum S100B concentration is above the age-related reference interval (Bouvier *et al.,* 2012). The aim of

this precaution is to not change the strategy planned by the physician before the assay (CCT-scan or hospitalization according to SFP recommendations (Lorton *et al.*, 2014)).

6.3.3. Randomization

Each center will be randomized according to the stepped wedge design considered for this study. Centers will be randomly allocated by the study's statistician (block design). Stratification according to planned recruitment of each participant center ("size of center") will also be proposed. A document describing the randomization procedure will be stored confidentially at the DRCI of the Clermont-Ferrand Hospital. All patients who will have the selection criteria and sign the consent form will be included in the same arm of a center.

The stepped wedge design also imposes some practical implementation challenges, such as preventing contamination between intervention participants and those waiting for the intervention and ensuring that those assessing outcomes are blind to the participant's status as intervention or control to help guard against information bias. Concerning contamination of physician waiting for the intervention, a visit or a video conference precising the exact nature of the intervention will be only performed in each center during the 15 days preceding the shift from the control period to the intervention period, in order to limit modification of usual practice in the management of control patients. Over the duration of the study, it is almost impossible to blind physicians performing outcome assessment since they will be aware of the "step" from control to intervention status. We will control for bias by assessing and grading outcomes according to pre-defined criteria and by assessing "subjective" outcomes by a blinding physician not involved in the pediatric care.

6.3.4. Reproducibility of the assay

The analytical method used in the study (at all study centers) is based on an electrochemiluminescence assay (on Roche Diagnostics® instruments). This is currently the most suitable technology because it is an automated piecemeal technology that is the most sensitive on the market. The previous publications from the Clermont-Ferrand group (Bouvier *et al.,* 2011 and 2012) have demonstrated the excellent feasibility of this technology for use in the future study. Its analytical performance (coefficient of variation of 3.1%) requires only a single determination (no need for duplicates) without risk of analytical error. The Clermont-Ferrand group confirms thes analytical performances (Bouvier *et al.*, 2015). The test sample volume (20 µL) and dead volume (150 µL) are altogether suitable for the type of blood sampling (micro-method) chosen to be least traumatic to the children. On the other hand, because of this small volume, it will not be possible to use the other commercially available assay (DiaSorin®), though in any case the pediatric reference values for this method are not available, which would have posed a problem of interpretation were we to have used this method.

6.3.5. Control of attrition bias

Missing data about primary endpoint are not expected in this short-term study (3 weeks).

7. Practical conduct of the study

7.1. Detailed description of procedures (description of each visit)

- ***« Conventional management » control arm***
  - CCT-scans and/or hospitalization will be prescribed by the physician in accordance with SFP recommendations
  - The Clinical Research Technician (CRT) from the study center will conduct a telephone interview 48 hours and 3 weeks after the trauma to ask a number of standardized questions (Appendix 4) and determine whether there has been any deterioration. The telephone number of the parents or legal guardian will be indicated on the consent form (Appendix 2).

- ***« S100B management» intervention arm***
  - Serum S100B concentrations will be determined on a peripheral venous blood sample drawn into a dry tube with separator gel, preferably by the micro-method technique (approximately 1 mL of blood), within the first 3 hours after the trauma.
    - If serum concentrations are above the reference interval (see section 6.2), a CCT-scan and/or hospitalization will be prescribed as per SFP recommendations (Lorton *et al.*, 2014).
    - If serum concentrations are within the reference range, the patient will be discharged after 6 hours of observation and the parents will be instructed on how to observe the child at home for the next 24 hours.

  - The CRT from the study center will conduct a telephone interview 48 hours and 3 weeks after the trauma to ask a number of standardized questions (Appendix 4) and determine whether there has been any deterioration. The telephone number of the parents or legal guardian will be indicated on the consent form (Appendix 2).

Some patients may undergo double window CCT-scan without contrast injection, with one window for the central nervous system (parenchyma window) and the other for the bones of the cranium (osseous window, with analysis of the vault and base of the skull, the cervico-occipital joint and the face). CCT-scan will be performed on multiarray machines. Patients who underwent CCT-scan will be classified into two groups according to the radiology report: CCT+ (presence of intracerebral lesion(s), and CCT- (no sign of intracerebral injury), but the radiologists will not know the result of the S100B protein assay. The effective radiation dose will be recorded (mSv) from the machine display.

7.2. Description of the general logistical organization of the study

Patients will be recruited in the pediatric emergency admission departments under the responsibility of the investigator at each center.

For the "S100B management" intervention arm, the blood required for serum S100B determination will be drawn in the pediatric emergency department and the blood sample will be conveyed to the medical biology laboratory at the same center. The laboratory biologist will then communicate the assay result to the investigator.

At each center, a CRT will collect all data required for the study.

7.3. Samples and biological analyses

For the “S100B management” intervention arm, the serum S100B assay will be carried out in the medical biology laboratory at each center (with the same technique of immunoassay in the 11 centers) under the responsibility of the biologist participating in the study.

An aliquot of each serum sample will be stored in a dedicated serum bank in case it needs to be retested at a later date. Also a panel of brain markers could be measured at the end of the study on the serum bank. The sample will then be destroyed after the results are exploited.

The immunoassay from Roche Diagnostics® holds CE marking for *in vitro* quantitative determination of S100B protein on human serum on any of the following instruments: Elecsys 1010, Elecsys 2010, Modular Analytics E 170, cobas e411 and cobas e601. The assay is based on electrochemoluminescence (ECLIA) with a total analytical cycle of 18 minutes comprising two incubation steps, followed by luminescence reading on a photomultiplier. The results are obtained with the aid of a two-point calibration curve generated for the instrument used and a reference curve memorized in the bar code of the reagent. The instrument automatically calculates the S100B protein concentration in each sample and expresses the results in µg/L. The result is not altered by icteria (bilirubin < 428 µmol/L), hemolysis (hemoglobin < 10 g/L) or lipemia (intralipid < 15 g/L). No hook effect has been observed for S100B protein concentrations of up to 100 µg/L. The range of measurement (defined as the limit of detection and the maximum of the reference curve) is 0.005 to 39 µg/L. Functional sensitivity is less than 0.02 µg/L. There is no cross-reactivity with S100A protein dimers. The coefficients of variation (VC) calculated on pooled human serum and on controls show an intra-series VC which is always less than 2.1%, implying an overall precision always less than 2.8% (Appendix 5).

The kit comprises three ready-to-use reagent bottles with the information required to perform the test memorized on the bar codes of each reagent bottle, which is enough for 100 assays. The composition of the three bottles is given in the product sheet (reference 03175243) in Appendix 5. The kits must be stored between +2 and +8°C and are stable when installed on the analytical instrument for a period of 4 weeks.

As indicated above, each kit is calibrated with CalSet calibrators (Roche Diagnostics®) according to the supplier’s recommendations. To guarantee that the assay is accurate and reproducible, two Elecsys PreciControl (low and high level) quality controls (Roche Diagnostics®) will be done daily. Serum from a patient will be centrifuged at 4000 rpm for 10 minutes and then assayed. Considering the controls performed and the proposed VCs, the determinations will be made on a single sample. The medical biology laboratory at each center is able to implement all the recommendations for use related to this assay.

7.4. Planned duration of participation and study time frame

Estimated study duration: 5 years and 1 month
Inclusion period: 60 months
Study start (first patient in): November 2016
Study end (last patient out): November 2021
Total duration of patient participation: 3 weeks

The date of the end of the study will be communicated to the competent authority and to the Ethics Committee within 90 days.

In the event of early study termination, the information will be communicated to the competent authority and to the Ethics committee within 15 days.

8. Study product

This is a diagnostic study. There are no unauthorized medicines and/or non-pharmacological treatments during the study.

8.1. Description of study product

Not applicable

8.2. Presentation of the products

Not applicable

8.3. Use of the product

Not applicable

8.4. Authorized and unauthorized medicines and treatments during the study

Not applicable

9. Data collected

The following information will be recorded on the case report form for each patient:

- Group in the study in which we included the child (control or interventional)

- Patient characteristics:
    - Identification code (center number + inclusion number + patient's initials)
    - Distance between home and hospital
    - Age
    - Gender
    - Current treatments
    - Intercurrent diseases

- Information about the mTBI:
    - Type of accident
    - Date and time of mTBI
    - GCS score at arrival at emergency department
    - Clinical signs:
        - presence (+) or absence (-) of signs of impaired consciousness
        - presence (+) or absence (-) of signs of skull fracture
        - presence (+) or absence (-) of signs of fracture of skull base
        - presence (+) or absence (-) of parietal or occipital scalp hematoma

  - presence (+) or absence (-) of loss of consciousness for more than 5 seconds in children under 2 years old
  - presence (+) or absence (-) of serious accident (road accident with passenger ejected from vehicle or death of another person or rollover; pedestrian hit by a moving vehicle; cyclist not wearing a helmet, fall from a height greater than 0.9 meter (in children under 2 years old) or 1.5 meter (in children over 2 years old))
  - Presence (+) or absence (-) of immediate loss of consciousness in children over 2 years old
  - Presence (+) or absence (-) of vomiting in children over 2 years old
  - Presence (+) or absence (-) of abnormal behavior in the opinion of parents in children under 2 years old
  - Presence (+) or absence (-) of severe headache in children over 2 years old

- Information related to serum S100B assay:
  - Date and time of blood sampling (time between mTBI and blood sampling)
  - Serum S100B concentration in µg/L
  - Concentration higher (+) or lower (-) than age-related reference value

- Information related to CCT-scan:
  - Presence (CCT+) or absence (CCT-) of intracranial trauma
  - Nature of lesion in case of CCT+
  - Effective radiation dose per patient (mSv)

- Information about the stay in the pediatric emergency department:
  - Date and time of arrival at emergency department (time between mTBI and arrival at emergency department)
  - Length of stay in emergency room
  - Length of stay in other hospital departments
  - Presence (+) or absence (-) of hospitalization
  - Indication (+) or absence of indication (-) for CCT-scan

- Information from the follow-up at 48 hours and 3 weeks post-mTBI: positive (+) or negative (-) answer to the standardized questions (Appendix 4)

- Information related to the cost of management
  - S100B monitoring
  - CCT-scan
  - Hospitalization by departments and other later hospitalization.

10.Statistical considerations

From a statistical viewpoint, the key characteristic of the stepped wedge cluster design is that the individual units within a cluster are correlated and this feature must be incorporated into

power calculations and the trial analysis. Analysis of the stepped wedge design is complex, particularly because of the need to control for temporal trends in outcome variables, such as fluctuations in disease prevalence over the course of the trial and accounting for repeated measures on the same individual. Hussey and Hughes (2007) provide a guide to the design and analysis for stepped wedge cluster randomized controlled trials, including the approaches to data analysis, sample size calculation, and power calculation. They suggested that power calculations should take into account the intra-cluster correlation, number of randomization steps, and treatment delay. As it was previously suggested, considering the number of participating centers (n= 11) and the duration of this study (60 months), it was proposed to fix 15 intervals (time periods) of 4 months each. Then, the randomization will involve 5 steps for which 4 (to 5) centers will be included in each cluster.

10.1. Sample size estimation

For an individual randomized trial, to show a 30% reduction in the rate of CCT-scans prescribed in the “S100B management” arm *versus* the “conventional management” arm, with 80% power and a two-sided risk I error of 5%, a sample of 800 subjects per arm is required. In fact, it is important to take into account that, based on the literature, the proportion of CCT-scans ordered during conventional management is roughly 20% (between 10% and 30%; 14% at Clermont-Ferrand Hospital) (Bouvier *et al.,* 2012). Therefore, to show a difference between the two arms of 6%, i.e. a 30% relative reduction (Bouvier *et al.,* 2012), a sample of 615 subjects per arm is required for an analysis using a chi-squared test for repeated clustered data. Furthermore, in light of the publication of Calcagnile *et al.* (2012), it is expected that 30% of physicians treating patients in the “S100B management” arm will not base their practice on the result of the S100B assay. Thus, in consideration of these points, it is planned to enroll 800 subjects per arm, so as to conserve a power of 80% with the previously defined hypotheses (Machin, 2004).

A fundamental assumption for randomized controlled trials is that the outcome for an individual patient is completely unrelated to that for any other patient - they are said to be 'independent'. This assumption is violated in cluster randomized clinical trials because patients within any one cluster (center in our case) are more likely to respond in a similar manner. A measure of this similarity is known as the intra-correlation coefficient (ICC). Because of this lack of independence, sample sizes require to be inflated. The sample size estimation for stepped wedged cluster randomized trials should consider ICC, variability of center population size (9 centers, coefficient of variation of cluster size (Eldridge *et al.*, 2006) defined as the ratio of the standard deviation of cluster sizes (45) to the mean cluster size (120 - 250) according to data given by participants centers), number of randomization steps (5 steps for which 2 (or 1) centers will be included) and duration of participation for each patient. Several simulations were proposed in relation to the value of ICC according to literature (Mdege *et al.*, 2011) and database of ICCs related by the University of Aberdeen (http://www.abdn.ac.uk/hsru/research/delivery/behaviour/methodological-research/).

According to these aspects (ICC=0.01 to 0.05, (Adams *et al.*, 2004)), 5% of lost to follow-up and the feasibility of this study (ability to recruit the estimated sample size), 2000 patients by group will be needed. An interim analysis is planned after enrolment of 2000 patients to estimate

statistical power according to observed ICC and absolute difference. The eventual decision to stop the study will be planned considering type I correction.

10.2. Data analysis: general points

The principal analysis will be performed using the software Stata (version13, StataCorp, College Station, TX). The tests will be two-sided, with a type I error set at α=0.05.

Quantitative variables will be presented as mean ± SD when normally distributed (assumption of normality studied by Shapiro-Wilk test), and for non-normal distributions as median, quartiles and range. Qualitative variables will be expressed as numbers and associated percentages. When possible, analyses will be displayed in graphs.

Inter-group comparisons will systematically be made 1) without adjustment and 2) adjusting on factors liable to be biased between groups.

10.3. Characteristics of patients at baseline

The two groups will be compared for the following patient characteristics at baseline: compliance with selection criteria, epidemiologic characteristics, clinical characteristics and treatments. Protocol violations and protocol violations per patient, as well as reasons for study dropout will also be described. The number of patients enrolled and the accrual curve will be presented for each arm.

10.4. Primary analysis

To compare the proportion of CCT-scans prescribed, a random-effect model taking into account center effect will be proposed more especially a generalized linear mixed model (logit according to statistical distribution). Randomization groups, steps of randomization, time periods and their interactions were evaluated as fixed effects. A robust Poisson mixed model should be used to complete these results in order to present results as relative risks and 95% confidence intervals.

10.5. Secondary analyses

Inter-group comparison on the other assessment criteria detailed previously will be based on the same models proposed for aim analysis to take into account between and within center variability: linear (duration of management, duration of hospitalization, effective radiation dose, quantity of sedatives prescribed – If necessary, a transformation to access the normality statistical distribution should be envisaged using log transformation for example) or generalized linear mixed model (age groups defined by age-related S100B cut-off values, sedation yes/no, presence of intracranial lesions on CCT-scan, presence of persistent clinical signs at the telephone interview) according to dependent variable. Adjusted analyses should be performed according to the univariate results and for clinically relevant parameters. Random-effects models were also used to study correlated longitudinal data (48 hours and 3 weeks) considering subject patient as random effect. These last models (with random effects) can also be used to take into account possible inter and intra physician variability in decision-making regarding the primary endpoint.

10.6. Economic analyses

The main objective of the economic analysis is to measure and to compare costs in the two arms, with early blood test of the protein S100B (intervention group) *versus* current management (control group).

We choose to perform a cost minimization analysis because for both, intervention or control group, the clinical endpoint is the same. Protein S100B blood test enables to care patient appropriately, to detect earlier TBI and to prevent from performing useless CT-scan.

The cost analysis will be performed under a University Hospital perspective. Our study will be focused on the analyses of costs of care and avoided costs in each arm. French hospital financing is based on a pricing scale for any current medical care. When an innovative medical care is developed, the financing system is temporarily based on grants. We hypothesize that the financing system for innovative medical care finances protein S100B blood test and that the price will be closed to the future price out of the regular pricing scale. Considering the French pricing system, if the number of CT-scan performed for cranial trauma decrease, due to the blood test of the protein S100B, the hospital might lose incomes. But, according to the waiting time for this exam and the public/private competition, we hypothesize that incomes remain stable because each avoided CT-scan will be replaced by another one, irrespective of the medical motive.

Costs will be analysed by microcosting during the standard follow-up of 3 weeks planned in the study: costs of S100B monitoring, costs of the CT-scans including medical costs if children should be under sedation during the CT-scan and costs of hospitalization by departments and other later hospitalizations.

So, the cost analysis focuses on avoided costs thanks to protein S100B blood test. We do not consider other consequences such as the impact of quicker appropriate care, the decrease of radiation withdrawal effects by reducing inappropriate CT-scan and the lower risk of healthcare-associated infections due to a shorter length of stay.

10.7. Method for managing missing, unused or invalid data

In view of the dispositions taken in this protocol, there is not expected to be any missing data on the primary endpoint.

Regarding other secondary endpoints, a sensitivity analysis will be proposed to define the statistical nature of possible missing data and propose the most appropriate method of imputation.

10.8. Persons in charge of analysis

Statistical analysis will be performed by Bruno Pereira (PhD in biostatistics), of the Clermont-Ferrand University Hospital Clinical Research and Innovation Delegation. Methodological developments on cluster randomized trials were BP's PhD theme (2008). BP is implied in several cluster randomized trials (see ref below) and gives his expertise about this subject for many institutes as INPES, INCa and WHO.

Vaillant-Roussel H, Laporte C, **Pereira B**, Tanguy G, Cassagnes J, Ruivard M et al. Patient education in chronic heart failure in primary care (ETIC) and its impact on patient quality of life: design of a cluster randomised trial. BMC Fam Pract. 2014 Dec 24;15:208.

Laporte C, Vaillant-Roussel H, **Pereira B**, Blanc O, Tanguy G, Frappé P et al. CANABIC: CANnabis and Adolescents: effect of a Brief Intervention on their Consumption--study protocol for a randomized controlled trial. Trials. 2014 Jan 30;15:40.

Sancho-Garnier H, **Pereira B**, Césarini P. A cluster randomized trial to evaluate a health education programme "Living with Sun at School". Int J Environ Res Public Health. 2012 Jul;9(7):2345-61.

Medico-economic objectives will be analysed by Charline Mourgues (MSc Economist), of the Clermont-Ferrand University Hospital Clinical Research and Innovation Delegation.

Mourgues C, Gerbaud L, Leger S, Auclair C, Peyrol F, Blanquet M et al. Positive and cost-effectiveness effect of spa therapy on the resumption of occupational and non-occupational activities in women in breast cancer remission: a French multicentre randomised controlled trial. Eur J Oncol Nurs. 2014 Oct;18(5):505-11.

11.Safety assessment – Management of adverse events

The investigator is responsible for reporting all adverse events on the case report form.

11.1. Definitions

Adverse event: any untoward medical occurrence in a patient or clinical investigation subject administered a pharmaceutical product and which does not necessarily have to have a causal relationship with the research or with this treatment.

Adverse effect: any untoward response related to the research.

Serious adverse effects are subgrouped as follows:

- ***Expected*** *serious adverse event*: any event that is described in the most recent version of the Investigator’s Brochure or in the Summary of Product Characteristics for marketed medicinal products, or in the instruction notice when the research concerns a medical device which is subject to CE marking. This definition also applies to an investigational medicinal product when administered for a same population outside the labeled indications.

- ***Unexpected*** *serious adverse event*: any event, the nature, severity or outcome of which is not consistent with the information in the most recent version of the Investigator’s Brochure or the Summary of Product Characteristics for a marketed medicinal product or the information notice for a medical device.

Serious adverse event or effect: any undesirable event or effect which results in death, is life-threatening, requires in-patient hospitalization or prolongation of existing hospitalization, results in persistent or significant disability/incapacity, or is a congenital anomaly/birth defect.

The term “life-threatening” refers to an event in which the patient was at risk of death at the time of the event, independently of the consequences of corrective or palliative treatment.

The terms “*disability*” or “*incapacity*” refer to any clinically significant, temporary or persistent disability.

Death, regardless of the cause, including when it corresponds to progression of the disease under treatment, is considered a serious adverse event.

Other events which do not correspond to the above definitions can be considered “*potentially serious*”, in particular certain laboratory anomalies. The investigator or sponsor’s medical judgement can result in such events being reported in the same manner as “serious” events. It is

necessary for study protocols to specify the characteristics of “potentially serious” events that are subject to reporting.

New information: event concerning the conduct of the research or the development of the medicinal product or related product which is the object of the research, when said new information may jeopardize the safety of the research subjects. Examples include:

- an increase in the rate of occurrence of serious events;
- serious adverse events related to the clinical trial procedures;
- lack of efficacy with a medicinal product used to treat life-threatening disease;
- a major safety finding from animal studies that provides new information on the safety of the product;
- and generally, any new information that could lead to an unfavorable reassessment of the benefit/risk ratio of the research.

Any new information concerning the research (or the product used) which may jeopardize the safety of the research subjects will be subjected to appropriate urgent measures and prompt and timely notification by the Sponsor to the competent authority and the Ethics Committee.

The only expected adverse effect is a risk of false negative results. However, this risk is virtually absent, in view of the sensitivity and the negative predictive value of the assay and the fact that the assay can only be done within 3 hours after the mTBI (see section 2.3 Summary of the benefits and foreseeable and known risks for research subjects).

11.2. Serious adverse event reporting

It is the investigator’s obligation to report within 24 hours any serious adverse event occurring in any patient enrolled in a study:

- during the active phase of the study,
- in the weeks following cessation of treatment,
- within the deadlines established for safety monitoring off treatment, before (wash-out or withdrawal phase) or after the active phase,
- after termination of the study, regardless of the time of the event, when no cause other than the research can reasonably be incriminated,

on the “Serious adverse event report form” (Appendix 7), indicating the date of onset, the severity, the causal relationship with the treatment (or product), and the follow-up/outcome.

The narrative describing the event should be completed and transmitted to the sponsor as soon as new, pertinent information is received. Depending on the nature and seriousness of the event, copies of the patient’s anonymized medical record can be attached, as well as laboratory results.

When a serious adverse event persists at the end of the study, the investigator will continue to follow the patient until said event is considered resolved.

In accordance with the implementing decree 2006-477 of 26/04/2006 amending chapter 1 of title II of Book I of the first part of the Public Health Code relating to biomedical research, all

suspected unexpected serious adverse effects must be reported by the sponsor to ANSM and to the Ethics Committee at first knowledge and no later than:

- 7 days after occurrence in case of death or a life-threatening event
- 15 days after occurrence for all other unexpected serious adverse events (SAE).

The sponsor will decide upon the significance of the serious adverse events that it reports and the consequences thereof, in particular with respect to the conduct of the research.

The sponsor will also assess the causality of the adverse event with the research by means of a joint analysis with the Regional Pharmacovigilance Center.

In the framework of this study, no expected serious adverse events are anticipated.

The sponsor will maintain a detailed list of all adverse events reported by the investigator(s).

Once per year, or on request, the sponsor will submit an annual safety update report to ANSM and to the Ethics Committee containing all available safety information.

The sponsor will also provide the investigators with any information that may affect the safety of the research subjects.

11.3. Monitoring Committee

An independent monitoring committee will be created, composed of:

Biochemist: ***Prof Jean-Louis Beaudeux***
Hôpital Necker-Enfants Malades
Biochemistry department
149 rue de Sèvres
75015 Paris, France

Pediatrician: ***Dr. Véronique Chasle***
Pediatrics- Medical/Surgical Emergencies Pediatrics Unit
Rennes Teaching Hospital
16, Boulevard de Bulgarie
35203 Rennes Cedex 2, France

Methodologist ***Dr Amélie Anota***
Biostatistics unit
Centre Léon Bérard
28 Prom. Léa et Napoléon Bullukian
69008 Lyon, France

This independent monitoring committee will meet a first time at study initiation and then throughout the duration of the study at its own initiative or at the sponsor's request. The committee will also issue a general opinion on the conduct of the study and can aid decision-making under the following circumstances:

- Opinion on premature study termination (because the study is no longer practicable or because the information needed to draw conclusions has already been obtained),
- Opinion on substantial protocol amendments that have become necessary for reasons of recruitment or monitoring, or to take account of new scientific data. All substantial protocol amendments will be submitted to the independent committee prior to submission to the sponsor, then to the Ethics Committee and to ANSM.

The Monitoring Committee's opinion will be submitted in writing to the sponsor.

11.4. Termination of the study

The study can be terminated for the following reasons:

- Notification of an excess rate of SAE
- Decision of the sponsor following new knowledge about the study product
- Decision of the investigator

11.5. Follow-up of patients presenting an adverse event

Patients with persistent serious adverse events at the end of the study will continue to be followed up by the investigator until the event is considered resolved. Patients who had non-serious adverse events will be followed up until the final study visit.

12. Right of access to source documents and data

12.1. Access to data

The sponsor is responsible for obtaining the agreement of all parties involved in the research in order to guarantee direct access to all study sites, source data, source documents and reports for purposes of the sponsor's quality control and audit.

The investigators will provide access to the documents and individual data that are strictly necessary for purposes of monitoring, quality control and audit of the biomedical research, to the persons authorized to consult said documents pursuant to the legislative and regulatory provisions in force (articles L.1121-3 and R.5121-13 Public Health Code).

12.2. Source data

Source documents, defined as any original document or object which proves the existence or accuracy of data or information recorded during the clinical study, will be stored for a period of 15 years by the investigator or by the hospital in the case of a hospital medical record.

12.3. Data confidentiality

Subject to the provisions relating to the confidentiality of data to which persons in charge of quality control of biomedical research have access (article L.1121-3 Public Health Code), and subject to the provisions relating to the confidentiality of information as concerns in particular the nature of the products being studied, the trials, the persons undergoing the research and the results obtained (article R.5121-13 Public Health Code), persons having direct access shall take all

necessary precautions to ensure the confidentiality of the information relating to the products being studied, the trials, the persons undergoing the research and notably their identity, and the results obtained.

These persons, as well as the investigators themselves, are bound by professional secrecy (in accordance with the conditions laid down in articles 226-13 and 226-14 of the penal code).

During the biomedical research or upon its completion, the data collected on the research subjects and transmitted to the sponsor by the investigators (or any other specialized study staff) shall be rendered anonymous.

In no case shall the names or addresses of the persons undergoing the research appear.

Anonymity of the subjects will be guaranteed by the creation of a subject code corresponding to 5 numerals and 2 letters, as follows: study center number (2 numerals) + inclusion number at center (3 numerals) + patient’s initials (last name, first name).

The sponsor will ensure that each research subject has given his written consent allowing access his personal data which is strictly necessary for quality control of the research.

12.4. Registration in the national file of biomedical research subjects

Not applicable

13.Quality control and assurance

13.1. Engagement of the investigators and the sponsor

The investigator undertakes to conduct the study in compliance with public health law 2004-806 of 9 August 2004 relating to biomedical research, the implementing decree 2006-477 of 26/04/2006 amending chapter 1 of title II of book 1 of the first part of the Public Health Code relating to biomedical research, and with the bylaws in force.

The study will also be conducted in compliance with Good Clinical Practices for biomedical research on medicinal products for human use, as laid down in article L.1121-3 Public Health Code and the decree of 24 November 2006.

The investigator also undertakes to comply with the Declaration of Helsinki of the World Medical Assembly (Tokyo 2004, revision).

13.2. Quality Assurance

A Clinical Research Associate (CRA) designated by the sponsor will ensure the proper conduct of the study, the collection of data generated in writing, and their documentation, recording and reporting, as per the Standard Operating Procedures in effect at the Clermont-Ferrand Hospital and in compliance with Good Clinical Practices and legislative and regulatory provisions in force.

13.3. Quality Control

The investigator guarantees the authenticity of the data collected during the study and accepts the legal provisions authorizing the study sponsor to implement quality control.

The coordinating investigator and associated investigators therefore agree to make themselves available during Quality Control visits by the Clinical Research Associate that will be scheduled at regular intervals. The following items will be examined at each visit:

- Informed consent
- Compliance with the study protocol and procedures
- Quality of data recorded in the case report forms: accuracy, missing data, coherence with source documents (medical records, appointment calendars, original copies of laboratory results, etc.)
- Management of any study products.

13.4. Case report form

All the information required by the study protocol will be recorded in an electronic case report form (eCRF). Data will be collected as it is obtained and transcribed in a clear and legible manner in the case report form.

The eCRF will be developed and accessible via the internet (SSL 128-bit SSL encryption). Access will be controlled by a personal password and all consultations and changes will be logged. Data will be entered in single input on the eCRF at each study center.

Data will be validated according to the data management plan jointly established between the coordinating investigator and the DRCI (methodologist, data manager and statistician). ACCESS® and STATA® software will be used. Data will be frozen/unfrozen according to standard procedures at Clermont-Ferrand Hospital (raw data frozen in XML format and as EXCEL® spreadsheets). All the data will be saved every night, kept for 4 weeks, then backed up to tape every month.

14. Ethical considerations

14.1. Ethics Committee

The study protocol, patient information notice and consent form and the case report form for the study will be submitted to the Southeast VI Ethics Committee for an opinion.

Notification of a favorable opinion from the Ethics Committee will be transmitted to the sponsor and to ANSM. A study authorization application will be sent by the sponsor to ANSM prior to study start.

14.2. Information for patients and written informed consent form

Patients and the parents or legal guardians will be informed in complete and faithful terms and in understandable language of the objectives and constraints of the study, the potential risks, the required observation and safety measures, and their right to refuse to participate in the study or to revoke their consent at any time. The investigator must also inform the subjects of the Ethics Committee opinion.

All this information appears in an information notice and consent form given to the patient (Appendix 1). The free, informed and written consent of the patient will be obtained by the investigator (Appendix 2). These documents (Appendix 1 and 2) are approved by the competent Ethics Committee and are to be used for the study in question, to the exclusion of any other document.

Two original copies will be co-signed by both the investigator and the patient and the parents or legal guardian. The second copy is to be kept in the patient's medical record.

14.3. Protocol amendments

Protocol amendments must be qualified as substantial or non-substantial. According to their nature, they will be the object of a new Ethics Committee opinion and/or authorization from the competent authority.

14.4. Management relating to the research

Patients in the "conventional management" control arm will be managed for mTBI according to SFP recommendations (Lorton *et al.*, 2014).

Patients in the "S100B" intervention arm will have a single blood sample drawn by a micro-method (total of 1 mL of blood) in a first step. They will then be managed according to the result of the serum S100B concentration:

- Serum S100B level below the cutoff defined according to age group: the patient will be discharged and return home after 6 hours of observation.
- Serum S100B level above the cutoff defined according to age group: the patient will be hospitalized and managed according to SFP recommendations (Lorton *et al.*, 2014).

In the framework of the study, parents or legal guardians of all enrolled patients must answer the follow-up questions at 48 hours and 3 weeks.

15. Data processing and storage of study documents and data

15.1. Data entry and processing

Data will be entered on an electronic CRF by the investigators and/or CRT at each study center.

The statistical analysis of the data will be carried out at the DCRI of Clermont-Ferrand Hospital by Bruno Pereira (methodologist, biostatistician).

15.2. CNIL

This study enters in the scope of "Reference Methodology" (MR-001) in application of the provisions of the law of 6 August 2004 relating to the protection of natural persons with regard to the processing of personal data and amending the law of 6 January 1978 relating to computer processing, data files and civil liberties. This change was approved by decision of 5 January 2006. Clermont-Ferrand Hospital, the study sponsor, has signed a commitment to comply with this "Reference Methodology" on 15/03/2007.

15.3. Record-keeping

The following documents will be archived under the study name in the Biochemistry department (Prof. Vincent Sapin) at Clermont-Ferrand Hospital until the end of the period of practical usefulness (60 months inclusion + 3 months for data analysis).

These documents are:

- Protocol and appendices, and any amendments,
- Signed, original information notices and consent forms,
- Individual data (authenticated copies of raw data),
- Follow-up documents
- Statistical analyses
- Final study report

At the end of the period of practical usefulness, all documents to be archived, such as defined in procedure PG.06.005 “Management of documentation relating to protocols” of Clermont-Ferrand Hospital will be transferred to the central archives and placed under the sponsor’s responsibility for a period of 15 years after study completion, in accordance with institutional practices.

These documents cannot be moved or destroyed without the sponsor’s permission. After the 15 years are up, the sponsor will be consulted for destruction. All the data as well as all documents and reports may be subject to audit or inspection.

16. Funding and insurance

16.1. Study budget

The budget is obtained from a PHRC (Hospital Clinical Research Program). The budget obtained is 538 186 €.

Some centers are not equipped with an automated instrument (Cobas, Roche Diagnostics) available 24/24 and 7/7, in which case it will be necessary to rent one (Cobas E411 Rotor with inverter). Roche Diagnostics France has agreed to offer a significant discount, since the catalogue price for renting this instrument is roughly 36,000 €. Each center not equipped with an automated instrument will also benefit from a two-way connection between the rented instrument and the laboratory’s computer system, thereby enabling secure data transmission and input of the S100B assay results on the hospital server for laboratory results. Furthermore, for non-equipped centers, Roche will deliver the instrument and provide on-site training to the operators.

16.2. Insurance

In accordance with regulatory provisions, Clermont-Ferrand Hospital, in its capacity as sponsor, has taken out civil liability insurance covering any damages resulting from the research with the Société Hospitalière d’Assurances Mutuelles (SHAM). The policy number is 147161.

It should be noted that non-observance of the legal conditions of the research (absence of Ethics Committee opinion, absence of ANSM authorization, non-consent of subjects, continuation of a suspended or prohibited study) shall render this coverage void.

17.Communication – Rules for publication

The data will only be disclosed after preliminary joint agreement of the investigator and the sponsor. The results will be the subject of communications and publications.

The study will be registered and declared on the *Clinical trials.gov*. internet site.

All publications of the results will mention the sources of funding for the study.

18.Feasibility of the study

The feasibility of the study is excellent based on the following criteria:

- The Medical Biochemistry and Pediatrics departments at Clermont-Ferrand Hospital, which are the principal investigators for this study, have extensive experience on the subject of "S100B and mTBI". In fact, our previous work on this subject has been published in national and international scientific journals (Bouvier *et al.,* 2009, 2011, 2012, 2012b, 2013 and 2015).
- Recruitment potential: The recruitment potential at each study center will amply meet the accrual target, since the pediatric emergency departments see children with mTBI every day. Among 446 children enrolled in our first study (Bouvier *et al*., 2012), 241 met the inclusion criteria for this new study in 1 year and considering that Clermont-Ferrand is a medium size hospital. In addition, blood was drawn for the S100B determination on average 2 hours 17 minutes after the trauma, indicating that the requirement to obtain a blood sample within 3 hours after trauma is not a feasibility constraint for this study.
- Regarding the practical conduct of the study, the blood samples will follow the same circuit as all biological samples from the pediatric emergency department and will therefore be processed in a time period compatible with the proper functioning of such a department.
- A CRA at the Clermont-Ferrand Hospital and a CRT at each study center will enable regular monitoring of the advancement of the study in compliance with the protocol.
- Lastly, the pediatrician investigators in the study are motivated by their desire to validate the utility of a biomarker allowing to reduce the number of CCT-scans prescribed for their patients.
- As explained before, statistical analysis will be performed by Bruno Pereira (PhD in biostatistics), of the Clermont-Ferrand University Hospital Clinical Research and Innovation Delegation. Bruno Pereira is implied in several cluster randomized trials and gives his expertise about this subject for many institutes as INPES, INCa and WHO.

19.Comments and answers to previous examinations

In order to show the dynamic improvement of our project to CPP members, we present in this chapter comments and answers to previous examninations from our PHRC (Hospital Clinical Research Program) candidature.

Previous expert comments

Expertise 2 :

« Le point le plus important à discuter est celui de la pertinence de la question de recherche posée. Il est en effet important d'évaluer en quoi la mise à disposition d'un test sanguin S100B permettant d'exclure un risque de complications permet de réduire, en pratique, l'utilisation du scanner. Les choix des urgentistes, qui restent maitres de leur démarche diagnostique, pronostique et

éventuellement thérapeutique, tiennent compte de facteurs difficiles à mesurer dans leur totalité, voire même à recenser. Il importe donc de connaitre l'impact effectif de la mise à disposition d'un nouvel outil. C'est la question posée par ce projet.
Cependant, cette question n'est pertinente que si nous avons la certitude de la fiabilité de l'outil diagnostique (S100B) lorsque l'on fait le choix de l'utiliser. Pour entrer dans les détails, il est important de connaitre le nombre de faux négatifs. Plus précisément, il importe de connaitre la valeur prédictive négative du test ainsi que, et c'est le plus important, la borne inférieur de son intervalle de confiance qui donne une idée de la probabilité d'avoir une complication même si le test S100 est négatif.
Pour connaître ces paramètres, des études doivent être conduites chez un grand nombre de patients, essentiellement parce que la fréquence des complications est faible. Comme le souligne les auteurs, cela a été fait parmi plusieurs milliers de patients adultes. Malheureusement, rien de tel chez l'enfant puisque les études disponibles portent ensembles sur moins de 500 patients. Le nombre total d'enfants avec un scan positif est extrêmement faible (pour faire simple, le résultat du scan est utilisé comme proxi de la complication pour améliorer la précision des estimations). Il importe donc :
1/ de se poser la question de la confiance que l'on a dans l'estimation de la valeur prédictive négative du test. Cette question fondamentale n'est pas abordée dans le document.
2/ En fonction de la réponse au point 1), il faut décider si la priorité doit être à l'amélioration de la précision de la mesure de cette VPN ou si nous pouvons passez à des mesures plus pragmatiques comme celle de la baisse de l'utilisation du scan. »

Corresponding answers Expertise 2

Oui, le nombre d'études sur l'intérêt de la S100B dans la prise en charge du traumatisme crânien léger de l'enfant est faible. Cela pose effectivement le problème de l'intervalle de confiance autour des valeurs de sensibilité et de VPN rapportées, qui étaient déjà précisées dans le protocole soumis l'an dernier, contrairement aux commentaires de l'expert 2. Néanmoins, ces éléments seront mieux précisés dans la nouvelle version. Nous souhaitons passer directement à une grande étude randomisée interventionnelle pour l'amélioration des pratiques en pédiatrie. Nos arguments sont les suivants:
Les innombrables études observationnelles chez l'adulte ont toutes démontré la même chose. Nous utilisons nous mêmes ce biomarqueur en routine pour notre SAU adultes et retrouvons les données de la littérature. Nous choisissons la sécurité avec le critère d'inclusion d'un prélèvement dans les 3h après le TCL comme dans les études de Castellani et al. (2009) et Bouvier et al. (2012) où la sensibilité est de 100%. Dans d'autres études (Bechtel et al., 2009 ; Babcock et al., 2012), le délai maximal entre le TCL et la prise de sang est de 6h et la sensibilité est plus faible.
Les cut off assurent une bonne sensibilité et des spécificités d'environ 30%. Nous sommes donc bien sur des seuils de dépistage privilégiant la sensibilité.
Nous allons dans la prochaine version mettre en place un comité de surveillance de l'étude constitué entre autre d'une pédiatre, le Pr Gras Le Guen, spécialiste du TCL de l'enfant et qui met en place le dosage de la S100B en routine dans son service.
Comme suggéré par d'autres experts, pour minimiser le risque, nous allons introduire dans le protocole du bras intervention une surveillance hospitalière clinique de 6 heures post TCL.

Enfin, les enfants avec traumatisme crânien léger à haut risque de lésion intracrânienne ne seront pas inclus (Cf critères d'exclusion et figures 2 et 3).

Expertise 2 :

« La pertinence du choix des mesures secondaires doit être mieux étudiée. Par exemple la mesure de la dose de radiation, ou la présence d'une lésion intracrânienne : quelle différence est attendue entre les groupes ? N'y a t il pas un biais d'indication ? Une plus grande fréquence de + dans le groupe S100 signe t elle un succès (ceux qui ont un scan malgré une S100 négative avaient d'autres bonnes raison d'être scannés) ou un échec (la valeur prédictive de la S100 n'est pas de 100). Le choix des mesures secondaires doit être mieux explicité en discutant les hypothèses sousjacentes.

Sur ce point enfin, pourquoi ne pas essayer de connaitre les raisons qui ont conduit les cliniciens à faire un scan en dépit d'une s100 negative ?

Page 22: Nevertheless, this study is intended to be pragmatic because in the "S100B management" arm, the pediatricians will still be able to not base their management on the S100B concentration. This aspect will be taken into account in the statistical considerations.

Il convient de dire comment cela sera fait. »

Corresponding answers Expertise 2

Les conseils de l'expert ont été suivis, une attention particulière sera portée aux raisons qui ont conduit les cliniciens à faire un scanner en dépit d'une s100b négative. Concernant le manque de précision des objectifs secondaires, il était déjà indiqué qu'une diminution de la dose de radiation était escomptée dans le bras s100b. Cet objectif a donc été conservé comme tel. Par contre, des précisions ont été apportées dans la description des critères de jugement secondaires concernant la présence d'une lésion intracrânienne : nombre de lésions rapporté au nombre de scanners réalisés. Nous présenterons dans la prochaine version deux schémas (un par bras) explicitant les algorithmes décisionnels de la SFP (Figures 2 et 3). Dans le bras interventionnel S100B, le dosage de S100B sera introduit après l'examen clinique et la décision de prescrire une TDM. D'une part, l'estimation des effectifs est majorée pour tenir de compte de ce fait. Concernant l'analyse statistique, une analyse descriptive détaillée sera réalisée pour les patients pour lesquels le médecin pédiatre ne suivra pas les recommandations (résultats s100b). Par ailleurs, comme cela était précisé dans le protocole, une analyse multivariée prenant en compte cet élément sera considérée.

Expertise 3 :

« Il s'agit d'un excellent projet d'étude clinique en pédiatrie, dont la méthodologie est adaptée et dont le rationnel, très bien explicité et correspond à la pratique des services recevant ces enfants, et où les recommandations SFMU sont maintenant largement appliquées. Ma seule réserve concerne la faisabilité de l'étude, même si l'expérience de l'équipe lors de leur dernière étude semble plutôt en accord avec ce qui est ici prévu. En effet, parmi tous les enfants admis pour TC aux urgences, nombre se présentent plus de 3 heures après l'accident, ce qui sera un facteur limitant de recrutement. Dans ce contexte, l'admission, l'installation, l'examen clinique, l'information de l'enfant et de sa famille, le recueil de consentement puis sa randomisation (même en ligne) constituent un « contre la montre » avant la ponction veineuse (lorsque le binome

d'infirmières/AP sera disponible …) qui risque d'être difficile à tenir. Dans ce contexte, je proposerais volontiers d'élargir le nombre des centres participants ou de partir sur une durée d'étude plus longue. Un design en steppedwedge permettrait également une randomisation par centre et non par enfant, sans doute moins chronophage dans ce contexte ?
Ce projet constitue un tel espoir d'amélioration des pratiques qu'il serait vraiment dommage qu'il soit mis en difficulté sur un recrutement insuffisant dans ce contexte difficile que constitue la recherche clinique aux urgences pédiatriques. »

Corresponding answers
Comme conseillé par l'expert, un design en steppedwedge sera adopté dans la version de cette année pour faciliter l'inclusion des enfants dans les centres investigateurs, ce qui implique automatiquement une inflation du nombre de sujets nécessaires afin de prendre au mieux en compte la variabilité inter et intra centre, mesurée par le coefficient de corrélation intraclasse.
Comme évoqué par l'expert, le chapitre faisabilité de notre précédente version montre que nous pourrons inclure suffisamment d'enfants. Nous allons également augmenter le nombre de centres pour nous en assurer.

Expertise 4 :
« Hypothèse relevante cliniquement mais le protocole est basé sur une seule étude qui détermine une VPN de 100% pour dédouaner une lésion avec des seuils variables selon l'âge.
Il me semble nécessaire de minimiser le risque en introduisant dans le protocole du bras intervention une surveillance hospitalière clinique de 6 heures post TC. On ne fait sortir aucun enfant inclus avant la 6eme heure. »

Corresponding answers Expertise 4 :

Nous allons suivre les conseils de l'auteur.

Expertise 5:
"This is an ambitious and well designed study which will have a massive impact on head injury management in children thoughtout the world. If the ethical board approves the study (which to me seems unlikely given the present evidence), I think this study will be very successful and will be publishable in a high-impact journal. It will have direct impact on patient care and the possibility of saving health-economic resources."

Corresponding answers Expertise 5 :
Merci à l'expert pour son encouragement. Le succès de l'étude et la publication de ses résultats sont très souhaités par les investigateurs qui ne désespèrent pas de mettre en place cette étude. Pour lever les craintes éthiques de l'expert, nous allons mettre en place un comité de surveillance de l'étude et garder tous les enfants aux urgences pour surveillance au moins 6 heures. Enfin, les enfants avec traumatisme crânien léger à haut risque de lésion intracrânienne ne seront pas inclus (Cf critères d'exclusion et figures 2 et 3).

20. Bibliography

Adams G, Gulliford MC, Ukoumunne OC, Eldridge S, Chinn S, Campbell MJ. Patterns of intra-cluster correlation from primary care research to inform study design and analysis. J Clin Epidemiol. 2004 Aug;57(8):785-94.

American Academy of Pediatrics. The management of mild closed head injury in children. Committee on Quality Improvement, Commission on Clinical Policies and Research, American Academy of Family Physicians. Pediatrics. 1999 Dec;104(6):1407-15.

Anderson RE, Hansson LO, Nilsson O, Liska J, Settergren G, Vaage J. Increase in serum S100A1-B and S100BB during cardiac surgery arises from extracerebral sources. Ann Thorac Surg. 2001 ; 71 :1512-7.

Beaudeux JL, Dequen L, Foglietti MJ. La protéine S-100beta : un nouveau marqueur biologique de pathologie cérébrale. Ann Biol Clin. 1999;57(3):261-72.

Beaudeux JL, Roche S, Puyssabet L, Foglietti MJ. Physiologie de la protéine S100B et apport de son dosage dans les pathologies neurologiques. Immunoanal Biol Spéc. 2001;16:14-148.

Beaudeux JL, Soler C, Foglietti MJ. Physiopathologie de la protéine S-100B : intérêt de son dosage en biologie clinique. Immunoanal Bio Spec. 2002;17: 280-6.

Biberthaler P, Linsenmeier U, Pfeifer KJ, Kroetz M, Mussack T, Kanz KG et al. Serum S-100B concentration provides additional information for the indication of computed tomography in patients after mild head injury: a prospective multicenter study. Shock. 2006 May;25(5):446-53.

Bouvier D, Duret T, Rouzaire P, Jabaudon M, Rouzaire M, Nourrisson C et al. Preanalytical, analytical, gestational and pediatric aspects of the S100B immuno-assays. Clin Chem Lab Med. 2015 Oct 17.

Bouvier D. [Interest of S100B protein blood level determination in severe or moderate head injury]. Ann Biol Clin (Paris). 2013 Mar-Apr;71(2):145-50.

Bouvier D, Castellani C, Fournier M, Dauphin JB, Ughetto S, Breton M et al. Reference ranges for serum S100B protein during the first three years of life. Clin Biochem. 2011 Jul;44(10-11):927-9.

Bouvier D, Eisenmann N, Gillart T, Bonneau J, Guelon D, Schoeffler P et al. [Jugular venous and arterial concentrations of serum S100B protein in patients with severe head injury]. Ann Biol Clin (Paris). 2012b May-Jun;70(3):269-75.

Bouvier D, Fournier M, Dauphin JB, Amat F, Ughetto S, Labbé A et al. Serum S100B determination in the management of pediatric mild traumatic brain injury. Clin Chem. 2012 Jul;58(7):1116-22.

Bouvier D, Oddoze C, Ben Haim D, Moustafa F, Legrand A, Alazia M et al. [Interest of S100B protein blood level determination for the management of patients with mild head trauma]. Ann Biol Clin (Paris). 2009 Jul-Aug;67(4):425-31.

Calcagnile O, Undén L, Undén J. Clinical validation of S100B use in management of mild head injury. BMC Emerg Med. 2012 Oct 27;12(1):13.

Castellani C, Bimbashi P, Ruttenstock E, Sacherer P, Stojakovic T, Weinberg AM. Neuroprotein s-100B -- a useful parameter in paediatric patients with mild traumatic brain injury? Acta Paediatr. 2009;98(10):1607-12.

Eldridge SM, Ashby D, Kerry S. Sample size for cluster randomized trials: effect of coefficient of variation of cluster size and analysis method. Int J Epidemiol. 2006 Oct;35(5):1292-300.

Homer CJ, Kleinman L. Technical report: mild head injury in children. Pediatrics. 1999 Dec;104(6):e78.

Hung R, Carroll LJ, Cancelliere C, Côté P, Rumney P, Keightley M et al. Systematic review of the clinical course, natural history, and prognosis for pediatric mild traumatic brain injury: results of the International Collaboration on Mild Traumatic Brain Injury Prognosis. Arch Phys Med Rehabil. 2014 Mar;95(3 Suppl):S174-91.

Hussey MA, Hughes JP. Design and analysis of stepped wedge cluster randomized trials. Contemp Clin Trials. 2007 Feb;28(2):182-91.

Jehlé E, Honnart D, Grasleguen C, Bouget J, Dejoux C, Lestavel P et al. Traumatisme crânien léger (score de Glasgow de 13 à 15) : triage, évaluation, examens complémentaires et prise en charge précoce chez le nouveau-né, l'enfant et l'adulte. Ann Fr Med Urgence. 2012 ; 2:199-214.

Kuppermann N, Holmes JF, Dayan PS, Hoyle JD, Atabaki SM, Holubkov R et al. Identification of children at very low risk of clinically-important brain injuries after head trauma: a prospective cohort study. Lancet. 2009;374(9696):1160-70.

Laporte C, Vaillant-Roussel H, Pereira B, Blanc O, Tanguy G, Frappé P et al. CANABIC: CANnabis and Adolescents: effect of a Brief Intervention on their Consumption--study protocol for a randomized controlled trial. Trials. 2014 Jan 30;15:40.

Lorton F, Levieux K, Vrignaud B, Hamel O, Jehlé E, Hamel A et al. Actualisation des recommandations pour la prise en charge du traumatisme crânien léger chez l'enfant. 2014 ; 21:790-796.

Machin D. On the evolution of statistical methods as applied to clinical trials. J Intern Med. 2004 May;255(5):521-8.

Mathews JD, Forsythe AV, Brady Z, Butler MW, Goergen SK, Byrnes GB et al. Cancer risk in 680,000 people exposed to computed tomography scans in childhood or adolescence: data linkage study of 11 million Australians. BMJ. 2013 May 21;346:f2360.

Manzano S, Holzinger IB, Kellenberger CJ, Lacroix L, Klima-Lange D, Hersberger M, La Scala G, Altermatt S, Staubli G. Diagnostic performance of S100B protein serum measurement in detecting intracranial injury in children with mild head trauma. Emerg Med J. 2015 (in press).

Mdege ND, Man MS, Taylor Nee Brown CA, Torgerson DJ. Systematic review of stepped wedge cluster randomized trials shows that design is particularly used to evaluate interventions during routine implementation. J Clin Epidemiol. 2011 Sep;64(9):936-48.

Miglioretti DL, Johnson E, Williams A, Greenlee RT, Weinmann S, Solberg LI et al. The use of computed tomography in pediatrics and the associated radiation exposure and estimated cancer risk. JAMA Pediatr. 2013 Aug 1;167(8):700-7.

Mourgues C, Gerbaud L, Leger S, Auclair C, Peyrol F, Blanquet M et al. Positive and cost-effectiveness effect of spa therapy on the resumption of occupational and non-occupational activities in women in breast cancer remission: a French multicentre randomised controlled trial. Eur J Oncol Nurs. 2014 Oct;18(5):505-11.

Pearce MS, Salotti JA, Little MP, McHugh K, Lee C, Kim KP et al. Radiation exposure from CCT scans in childhood and subsequent risk of leukaemia and brain tumours: a retrospective cohort study. Lancet. 2012;380(9840):499-505.

Sanchez-Peña P, Pereira AR, Sourour NA, Biondi A, Lejean L, Colonne C et al. S100B as an additional prognostic marker in subarachnoid aneurysmal hemorrhage. Crit Care Med. 2008;36(8):2267-73.

Sancho-Garnier H, Pereira B, Césarini P. A cluster randomized trial to evaluate a health education programme "Living with Sun at School". Int J Environ Res Public Health. 2012 Jul;9(7):2345-61.

Simon-Pimmel J, Lorton F, Guiziou N, Levieux K, Vrignaud B, Masson D et al. Serum S100β Neuroprotein Reduces Use of Cranial Computed Tomography in Children After Minor Head Trauma. Shock. 2015 Nov;44(5):410-6.

Undén J, Romner B. Can low serum levels of S100B predict normal CT findings after mild head injury in adults?: an evidence-based review and meta-analysis. J Head Trauma Rehabil. 2010 Jul-Aug;25(4):228-40.

Vaillant-Roussel H, Laporte C, Pereira B, Tanguy G, Cassagnes J, Ruivard M et al. Patient education in chronic heart failure in primary care (ETIC) and its impact on patient quality of life: design of a cluster randomised trial. BMC Fam Pract. 2014 Dec 24;15:208.

21. List of appendix

Appendix 1: Fiches d'information

Appendix 2: Formulaires de consentement

Appendix 3: Score de Glasgow en fonction de l'âge

Appendix 4: Fiche d'évolution clinique

Appendix 5: Notice du kit de dosage de la protéine S100B (Roche)

Appendix 6: Fiche de déclaration d'événement indésirable grave

APPENDIX 1A
FORMULAIRE D'INFORMATION
POUR LES PARENTS OU TITULAIRES DE L'AUTORITE PARENTALE

Etude interventionnelle évaluant l'apport du dosage sanguin de la protéine S100B dans la prise en charge du traumatisme crânien léger de l'enfant
PROS100B

- **Promoteur**
  CHU de Clermont-Ferrand
  58 Rue de Montalembert, 63003 Clermont-Ferrand Cedex 1

- **Investigateur coordonnateur : Damien Bouvier**
  Service de Biochimie Médicale
  CHU de Clermont-Ferrand

Madame, Monsieur,

Nous vous proposons de faire participer votre enfant à une recherche dont l'intitulé est mentionné ci-dessus, qui va se dérouler dans le Service des Urgences pédiatriques de plusieurs centres hospitaliers en France.

L'objectif de l'étude est d'améliorer la stratégie de prise en charge des enfants traumatisés crâniens.

Par le dosage sanguin d'une protéine (protéine S100B), nous pouvons écourter en cas de résultat normal, le temps d'hospitalisation, et diminuer le nombre d'examens complémentaires chez l'enfant.

La protéine S100B est synthétisée essentiellement dans des cellules cérébrales et est libérée dans le sang en cas de traumatisme crânien.

L'étude se déroulera en 2 étapes :

- Votre enfant va bénéficier de manière aléatoire (tirage au sort) :
  - d'une prise en charge classique selon les recommandations de la Société Française de Médecine d'Urgence et du Groupe francophone de réanimation et urgences pédiatriques

  ou
  - d'une prise en charge avec dosage sanguin de la protéine S100B. Dans ce dernier cas, votre enfant aura une prise de sang réalisée par une infirmière du service. Le dosage de la protéine S100B nécessite le prélèvement d'un seul tube de sang, soit par prise de sang classique (Tube de 2 à 5 ml de sang), soit par micro-méthode pour les nourrissons de moins de 6 mois (prélèvement au bout du doigt ou éventuellement au talon).
    - En cas de résultat normal, votre enfant pourra écourter son séjour aux urgences avec des consignes classiques de surveillance à domicile.
    - En cas de résultat anormal, la prise en charge classique sera réalisée.
- Ensuite une personne du corps médical vous appellera à domicile à 48h et 3 semaines après le traumatisme de votre enfant pour vous poser quelques questions concernant son état de santé.

La participation de votre enfant à cette étude peut faire progresser les moyens thérapeutiques mis à la disposition du corps médical.

La prise de sang sera réalisée dans des règles strictes d'asepsie. Les risques encourus par votre enfant sont ceux qui peuvent être rencontrés lors d'une prise de sang (malaise, ecchymose au point de ponction, douleur locale…).

Un échantillon anonymisé de cette prise de sang sera conservé au sein du laboratoire de l'hôpital et sera envoyé au CRB Auvergne (Centre de Ressource Biologique) du Centre Hospitalier de Clermont-Ferrand en fin d'étude, afin d'être utilisé pour des programmes de recherche, dans le respect de la confidentialité. Ces programmes de recherche porteront sur les pathologies infantiles et viseront à améliorer les connaissances et la prise en charge de ces pathologies.

Aucun test génétique ne pourra être réalisé sans votre accord.

Conformément à la loi (article 16-1 et 16-6 du code civil), ce prélèvement ne pourra être cédé à titre commercial, ni donner lieu à une rémunération à votre bénéfice. Il pourra être utilisé pour des recherches effectuées soit par les équipes de recherche du CHU de Clermont-Ferrand, soit en partenariat avec un ou plusieurs organismes publics ou privés soit après cession par un ou plusieurs organismes publics ou privés.

Votre participation à cette recherche biomédicale n'engendrera pour vous aucun frais supplémentaire par rapport à ceux que vous auriez dans la prise en charge habituelle de votre enfant.

Toutefois, pour pouvoir participer à cette recherche vous devez être affiliés ou bénéficier d'un régime de sécurité sociale.

Le CHU de Clermont Ferrand, qui organise cette recherche biomédicale en qualité de promoteur, a contracté une assurance conformément aux dispositions législatives, garantissant sa responsabilité civile et celle de tout intervenant auprès de la Société Hospitalière d'Assurances Mutuelles (SHAM, contrat n°147161). Dans le cas où l'état de santé de votre enfant serait altéré du fait de sa participation à l'étude, conformément à la loi de Santé Publique n°2004-806 du 9 août 2004, vous seriez en droit de recevoir des dédommagements dans le cadre de ce contrat d'assurance spécifique.

Cette recherche a reçu l'avis favorable du Comité de Protection des Personnes Sud Est VI le 08/06/2016 ainsi que l'autorisation préalable de l'autorité compétente de santé.

Il est possible que cette recherche soit interrompue, si les circonstances le nécessitent, par le promoteur ou à la demande de l'autorité de santé.

Les informations relatives à l'étude recueillies par l'investigateur sont traitées confidentiellement.

<u>Protection de vos données personnelles</u> :

Dans le cadre de cette recherche, le CHU de Clermont-Ferrand est responsable de la mise en œuvre du traitement de données à caractère personnel. Ce traitement informatique a pour but d'analyser les résultats de la recherche au regard de l'objectif de cette dernière qui vous a été présenté.

Le fondement juridique, au regard de l'article 6 du RGPD (Règlement Général sur la Protection des Données) est l'intérêt légitime du promoteur à mettre en œuvre le traitement de données médicales à des fins de recherche scientifique (article 9.2 du RGPD).

A cette fin, les données médicales concernant votre enfant et les données relatives à vos habitudes de vie nécessaires pour la recherche seront transmises au Promoteur, ou aux personnes ou sociétés agissant pour son compte, en France ou à l'étranger.

Ces données seront identifiées par un numéro de code et les initiales de votre enfant. Ces données pourront également, dans des conditions assurant leur confidentialité, être transmises aux autorités de santé françaises, à d'autres entités du CHU de Clermont Ferrand.

Les données seront conservées au minimum 15 ans après la fin de la recherche, selon les dispositions légales en vigueur.

Conformément aux dispositions de la loi informatique et libertés du 6 janvier 1978 modifiée, vous disposez d'un droit d'accès, de rectification et de limitation du traitement des données de votre enfant. Vous disposez également d'un droit d'opposition à la transmission des données couvertes par le secret professionnel susceptibles d'être utilisées dans le cadre de cette recherche et d'être traitées.

Conformément à l'article 17.3 du RGPD, les données recueillies préalablement au retrait du consentement, le cas échéant, ne pourront pas être effacées et pourront continuer à être traitées dans les conditions prévues par la recherche.

Pour exercer ces droits ou pour toute question sur le traitement des données de votre enfant, vous pouvez contacter notre délégué à la protection des données : CHU de Clermont-Ferrand – Direction de la Qualité – Gestion des Risques et Droits des Usagers – 58 rue Montalembert – 63003 Clermont-Ferrand cedex 1 (ou dpd@chu-clermontferrand.fr)

Vous pouvez également accéder directement ou par l'intermédiaire d'un médecin de votre choix à l'ensemble des données médicales de votre enfant en application des dispositions de l'article L. 1111-7 du code de la santé publique. Ces droits s'exercent auprès du médecin qui suit votre enfant dans le cadre de la recherche et qui connaît son identité.

Si vous estimez, après nous avoir contactés, que vos droits Informatique et Libertés ne sont pas respectés ou que le dispositif de contrôle d'accès n'est pas conforme aux règles de protection des données, vous pouvez adresser une réclamation auprès de la CNIL par courrier.

Vous êtes libre d'accepter ou de refuser que votre enfant participe à cette recherche sans avoir à vous justifier. De plus vous pouvez exercer à tout moment votre droit de retrait de cette recherche sans avoir à donner de raison. Le fait de ne plus participer à cette recherche ne modifiera pas la qualité des soins qui lui seront prodigués. Vous pouvez demander à tout moment des explications complémentaires sur l'étude à l'équipe soignante.

Par ailleurs, vous pourrez être tenu informé des résultats globaux de cette recherche à la fin de l'étude.

Lorsque vous aurez lu cette note d'information et obtenu les réponses aux questions que vous vous posez en interrogeant le médecin investigateur, il vous sera proposé, si vous êtes en accord, de donner votre consentement écrit en signant le document préparé à cet effet après avoir eu un moment de réflexion en dehors de la présence d'un membre du CHU.

Date : …../…./…..

Signature des parents ou des titulaires de l'autorité parentale :
(Précédée de la mention « Lu et compris »)

Paraphe de l'investigateur

Version N°3 – 12/11/2018

APPENDIX 1B
FORMULAIRE D'INFORMATION
POUR LES ENFANTS DE 8 A 13 ANS

Etude interventionnelle évaluant l'apport du dosage sanguin de la protéine S100B dans la prise en charge du traumatisme crânien léger de l'enfant
PROS100B

Tu viens de te faire mal à la tête et tu as dû venir à l'hôpital pour qu'on te soigne. Nous te proposons de participer à une étude.

Pour cette étude, on va peut-être te faire une prise de sang. Elle permet de prendre un peu de ton sang pour le faire analyser dans un laboratoire. Les résultats pourront aider les médecins à évaluer ton traumatisme à la tête et donc à bien te soigner.
Ton sang sera conservé au laboratoire de l'hôpital pour essayer de mieux soigner les maladies chez les enfants.

- Le but est de faire une prise de sang pour mieux surveiller les enfants qui se font mal à la tête,

- Les résultats de ta prise de sang seront utilisés pour la recherche médicale sans que ton nom soit écrit.

- Tu pourras poser les questions que tu veux au docteur.

TU PEUX DIRE NON SI TU NE VEUX PAS QU'ON FASSE CETTE RECHERCHE.

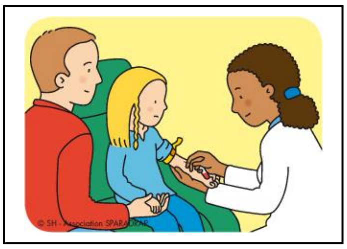

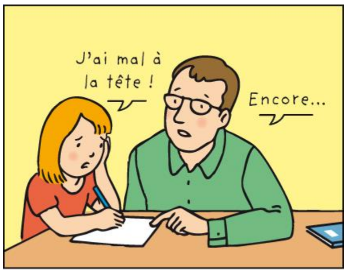

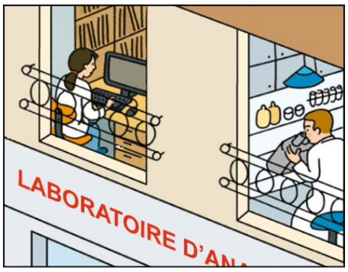

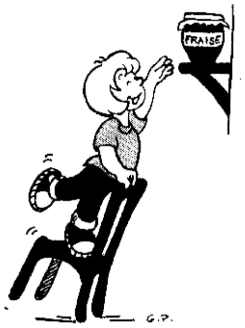

Version N° 4 – 12/11/2018

APPENDIX 1C
FORMULAIRE D'INFORMATION POUR LES ADOLESCENTS

Etude interventionnelle évaluant l'apport du dosage sanguin de la protéine S100B dans la prise en charge du traumatisme crânien léger de l'enfant
PROS100B

- **Promoteur**
  CHU de Clermont-Ferrand
  58 Rue de Montalembert, 63003 Clermont-Ferrand Cedex 1

- **Investigateur coordonnateur : Damien Bouvier**
  Service de Biochimie Médicale
  CHU de Clermont-Ferrand

Mademoiselle, Monsieur,

Nous te proposons de participer à une recherche dont l'intitulé est mentionné ci-dessus, qui est organisée par le Service des Urgences pédiatriques du CHU de Clermont-Ferrand.

L'objectif de l'étude est d'améliorer la stratégie de prise en charge des enfants traumatisés crâniens.

Par le dosage sanguin d'une protéine (protéine S100B), nous pourrons écourter en cas de résultat normal, le temps d'hospitalisation, et diminuer le nombre d'examens complémentaires.

La protéine S100B est synthétisée essentiellement dans des cellules cérébrales et est libérée dans le sang en cas de traumatisme crânien.

L'étude se déroule de la manière suivante :

- Tu vas bénéficier de manière aléatoire (tirage au sort) :
  - d'une prise en charge classique selon les recommandations de la Société Française de Médecine d'Urgence et du Groupe francophone de réanimation et urgences pédiatriques

  ou

  - d'une prise en charge avec dosage sanguin de la protéine S100B. Dans ce dernier cas, tu auras une prise de sang réalisée par une infirmière du service. Le dosage de la protéine S100B nécessite le prélèvement d'un seul tube de sang par prise de sang classique (Tube de 2 à 5 ml de sang).
    - En cas de résultat normal, tu pourras écourter ton séjour aux urgences avec des consignes classiques de surveillance à domicile.
    - En cas de résultat anormal, la prise en charge classique sera réalisée.

- Ensuite, une personne du corps médical te rappellera (toi ou tes parents) à domicile 48h et 3 semaines après ton traumatisme pour vous poser quelques questions concernant ton état de santé.

Ta participation à cette étude peut aider les médecins à mieux soigner les patients.

La prise de sang sera réalisée dans des règles strictes d'asepsie. Les risques que tu encoures sont ceux qui peuvent être rencontrés lors d'une prise de sang (malaise, ecchymose au point de ponction, douleur locale…).

Un échantillon anonymisé de cette prise de sang sera conservé au sein du laboratoire de l'hôpital et sera envoyé au CRB Auvergne (Centre de Ressource Biologique) du Centre Hospitalier de Clermont-Ferrand en fin d'étude, afin d'être utilisé pour des programmes de recherche, dans le respect de la confidentialité. Ces programmes de recherche porteront sur les pathologies infantiles et viseront à améliorer les connaissances et la prise en charge de ces pathologies.

Aucun test génétique ne pourra être réalisé sans ton accord.

Conformément à la loi (article 16-1 et 16-6 du code civil), ce prélèvement ne pourra être cédé à titre commercial, ni donner lieu à une rémunération à votre bénéfice. Il pourra être utilisé pour des recherches effectuées soit par les équipes de recherche du CHU de Clermont-Ferrand, soit en partenariat avec un ou plusieurs organismes publics ou privés soit après cession par un ou plusieurs organismes publics ou privés.

Ta participation à cette recherche biomédicale n'engendrera pour tes parents aucun frais supplémentaire par rapport à ceux que vous auriez en cas de prise en charge habituelle.

Toutefois, pour pouvoir participer à cette recherche tes parents doivent être affiliées ou bénéficier d'un régime de sécurité sociale.

Le CHU de Clermont Ferrand, qui organise cette recherche biomédicale en qualité de promoteur, a contracté une assurance conformément aux dispositions législatives, garantissant sa responsabilité civile et celle de tout intervenant auprès de la Société Hospitalière d'Assurances Mutuelles (SHAM, contrat n°147161). Dans le cas où ton état de santé serait altéré du fait de ta participation à l'étude, conformément à la loi de Santé Publique n°2004-806 du 9 août 2004, vous seriez en droit de recevoir des dédommagements dans le cadre de ce contrat d'assurance spécifique.

Cette recherche a reçu l'avis favorable du Comité de Protection des Personnes Sud Est VI le 08/06/2016 ainsi que l'autorisation préalable de l'autorité compétente de santé.

Les informations relatives à l'étude recueillies par l'investigateur sont traitées confidentiellement (traitement informatisé anonyme).

<u>Protection de tes données personnelles</u> :

Dans le cadre de cette recherche, le CHU de Clermont-Ferrand est responsable de la mise en œuvre du traitement informatique des données à caractère personnel qui a pour but d'analyser les résultats de la recherche.

Selon l'article 6 du RGPD (Règlement Général sur la Protection des Données), les données médicales te concernant et les données relatives à tes habitudes de vie nécessaires pour la recherche seront transmises au Promoteur, ou aux personnes ou sociétés agissant pour son compte, en France ou à l'étranger.

Ces données seront identifiées par un numéro de code et tes initiales. Ces données pourront également, dans des conditions assurant leur confidentialité, être transmises aux autorités de santé françaises, à d'autres entités du CHU de Clermont Ferrand.

Les données seront conservées au minimum 15 ans après la fin de la recherche, selon les dispositions légales en vigueur.

Conformément aux dispositions de la loi informatique et libertés du 6 janvier 1978 modifiée, tu disposes d'un droit d'accès, de rectification et de limitation du traitement de tes données. Tu disposes également d'un droit d'opposition à la transmission des données couvertes par le secret professionnel susceptibles d'être utilisées dans le cadre de cette recherche et d'être traitées.

Tu peux également accéder directement ou par l'intermédiaire d'un médecin de ton choix à l'ensemble de tes données médicales selon l'article L. 1111-7 du code de la santé publique. Ces droits s'exercent auprès du médecin qui te suit dans le cadre de la recherche et qui connaît ton identité.

Tu es libre d'accepter ou de refuser de participer à cette recherche sans avoir à te justifier. De plus tu peux exercer à tout moment ton droit de retrait de cette recherche sans avoir à donner de raison. Le fait de ne plus participer à cette recherche ne modifiera pas la qualité des soins qui te seront prodigués. Tu peux demander à tout moment des explications complémentaires sur l'étude à l'équipe soignante.

Par ailleurs, tu pourras être tenu informé des résultats globaux de cette recherche à la fin de l'étude.

Lorsque tu auras lu cette note d'information et obtenu les réponses aux questions que tu te poses en interrogeant le médecin investigateur, il te sera proposé, si tu es d'accord, de donner ton consentement écrit en signant le document préparé à cet effet, après avoir eu un moment de réflexion en dehors de la présence d'un membre du CHU.

Date : …../…./…..

Signature du patient **Paraphe de l'investigateur**
(Précédée de la mention « Lu et compris »)

APPENDIX 2A
FORMULAIRE DE CONSENTEMENT DE PARTICIPATION A UNE RECHERCHE BIOMEDICALE
POUR LES PARENTS OU TITULAIRES DE L'AUTORITE PARENTALE

Etude interventionnelle évaluant l'apport du dosage sanguin de la protéine S100B dans la prise en charge du traumatisme crânien léger de l'enfant
PROS100B

Investigateur coordonnateur : **Damien Bouvier**
Service de Biochimie Médicale – CHU de Clermont-Ferrand

Je soussigné(e)
Mme, M (*nom, prénom*)…………………………………………
Né(e) le …………………………………………………………………
Demeurant……………………………………………………………………………………
Téléphone fixe………………………….. Téléphone portable…………………………………
Déclare :
- que le Docteur (*nom, prénom*)……………………………………… m'a proposé de faire participer mon enfant à l'étude sus nommée,
- qu'il m'a expliqué en détail le protocole,
- qu'il m'a notamment fait connaître :
  - l'objectif, la méthode et la durée de l'étude
  - les contraintes et les risques potentiels encourus
  - le rappel téléphonique à 48h et à 3 semaines
  - mon droit de refuser de participer et en cas de désaccord de retirer mon consentement à tout moment
  - mon obligation d'inscription à un régime de sécurité sociale
  - que, si je le souhaite, à son terme, je serai informé par le médecin investigateur des résultats globaux de la recherche
  - que le Comité de Protection des Personnes Sud Est VI a émis un avis favorable en date du 08/06/2016
  - que dans le cadre de cette étude le promoteur, le CHU de Clermont-Ferrand, a souscrit à une assurance couvrant cette recherche.

Les informations relatives à l'étude recueillies par l'investigateur sont traitées confidentiellement.

J'accepte que les données enregistrées à l'occasion de cette recherche puissent faire l'objet d'un traitement informatisé anonyme. J'ai bien noté que les droits d'accès, de rectification, d'opposition et de limitation du traitement des données prévus par la loi informatique et libertés du 6 janvier 1978 modifiée s'exercent à tout moment auprès du médecin qui suit mon enfant dans le cadre de la recherche et qui connaît l'identité de mon enfant ou du délégué de protection des données du promoteur dont les coordonnées sont mentionnées dans la note d'information qui m'a été remise.

Je pourrai à tout moment demander des informations complémentaires au Dr …………….. en appelant le ………………………

Après avoir discuté librement et obtenu réponse à toutes mes questions, j'accepte librement et volontairement de participer à cette recherche biomédicale dans les conditions précisées dans le formulaire d'information et de consentement.

Nom et prénom du patient :	Nom de l'investigateur :
…………………………………………………	…………………………………………………
Date :……./……./…….	Date :……./……./…….
Signature des parents ou des titulaires de l'autorité parentale :	Signature :

Précédée de la mention « Lu et compris » :

Ce document est à réaliser en 2 exemplaires originaux, dont le premier doit être gardé 15 ans par l'investigateur, un autre remis à la personne donnant son consentement.

Version N°3 – 12/11/2018

APPENDIX 2B
FORMULAIRE DE CONSENTEMENT DE PARTICIPATION A UNE RECHERCHE BIOMEDICALE
POUR L'ENFANT DE 8 A 13 ANS

Etude interventionnelle évaluant l'apport du dosage sanguin de la protéine S100B dans la prise en charge du traumatisme crânien léger de l'enfant
PROS100B

Investigateur coordonnateur : **Damien Bouvier**
Service de Biochimie Médicale – CHU de Clermont-Ferrand

De M. ou Mlle ....(Nom, Prénom)

Né(e) le ....

Le Docteur .... m'a proposé de participer à l'étude organisée par le CHU de Clermont-Ferrand. Il m'a précisé que je suis libre d'accepter ou de refuser ; ceci ne changera pas nos relations. Le but, les risques et la durée de cette étude et la manière dont elle va se passer m'ont été clairement expliqués. J'ai bien compris toutes les informations qui m'ont été fournies.

Je pourrai à tout moment demander une information complémentaire au médecin, en faisant appeler mes parents au ………………………….

Si je le souhaite, je pourrai quand je veux arrêter ma participation à cette étude. J'en informerai alors immédiatement le Docteur ……………………

Il m'a été signalé que les résultats de la prise de sang seront utilisés sans que mon identité ne soit révélée.

Je sais que cette étude a reçu l'approbation du Comité de Protection des Personnes (CPP) Sud Est VI lors de sa séance du 08/06/2016

J'ACCEPTE DE PARTICIPER A CETTE RECHERCHE DANS LES CONDITIONS PRECISEES DANS LE DOCUMENT D'INFORMATION QUI M'A ÉTÉ REMIS AVEC CE FORMULAIRE ET QUE J'AI LU AVEC ATTENTION.

Nom et prénom du patient :
…………………………………………………

Date :……./……./…….
Signature
Précédée de la mention « Lu et compris » :

Nom de l'investigateur :
…………………………………………………

Date :……./……./…….
Signature :

Ce document est à réaliser en 2 exemplaires originaux, dont le premier doit être gardé 15 ans par l'investigateur, un autre remis à la personne donnant son consentement.

APPENDIX 2C
FORMULAIRE DE CONSENTEMENT DE PARTICIPATION A UNE RECHERCHE BIOMEDICALE POUR L'ADOLESCENT

Etude interventionnelle évaluant l'apport du dosage sanguin de la protéine S100B dans la prise en charge du traumatisme crânien léger de l'enfant
PROS100B

Investigateur coordonnateur : **Damien Bouvier**
Service de Biochimie Médicale – CHU de Clermont-Ferrand

Je soussigné(e)
Mme, M (*nom, prénom*)…………………………………………
Né(e) le ………………..……………………………………………….
Demeurant………………………………………………………………………………………
Déclare :
- que le Docteur (*nom, prénom*) ……………….………………………… m'a proposé de participer à l'étude sus nommée,
- qu'il m'a expliqué en détail le protocole,
- qu'il m'a notamment fait connaître :
  - l'objectif, la méthode et la durée de l'étude
  - les contraintes et les risques potentiels encourus
  - mon droit de refuser de participer et en cas de désaccord de retirer mon consentement à tout moment
  - l'obligation de mes parents d'inscription à un régime de sécurité sociale
  - que, si je le souhaite, à son terme, je serai informé par le médecin investigateur des résultats globaux de la recherche
  - que le Comité de Protection des Personnes Sud Est VI a émis un avis favorable en date du 08/06/2016
  - que dans le cadre de cette étude le promoteur, le CHU de Clermont-Ferrand, a souscrit à une assurance couvrant cette recherche.

Les informations relatives à l'étude recueillies par l'investigateur sont traitées confidentiellement.

J'accepte que les données enregistrées à l'occasion de cette recherche puissent faire l'objet d'un traitement informatisé anonyme. J'ai bien noté que les droits d'accès, de rectification, d'opposition et de limitation du traitement des données prévus par la loi informatique et libertés du 6 janvier 1978 modifiée s'exercent à tout moment auprès du médecin qui me suit dans le cadre de la recherche et qui connaît mon identité ou du délégué de protection des données du promoteur dont les coordonnées sont mentionnées dans la note d'information qui m'a été remise.

J'ACCEPTE DE PARTICIPER A CETTE RECHERCHE DANS LES CONDITIONS PRECISEES DANS LE DOCUMENT D'INFORMATION QUI M'A ÉTÉ REMIS AVEC CE FORMULAIRE ET QUE J'AI LU AVEC ATTENTION

Je pourrai à tout moment demander des informations complémentaires au Dr .... en appelant le ....

Nom et prénom du patient :
…………………………………………………

Date :……./……./…….
Signature
Précédée de la mention « Lu et compris » :

Nom de l'investigateur :
…………………………………………………

Date :……./……./…….
Signature :

Ce document est à réaliser en 2 exemplaires originaux, dont le premier doit être gardé 15 ans par l'investigateur, un autre remis à la personne donnant son consentement.

APPENDIX 3

Score de Glasgow en fonction de l'âge
Recommandations de la SFMU
Jehlé *et al.,* 2012

Echelle de Glasgow standard (> 5 ans)	Echelle de Glasgow de 2 à 5 ans	Echelle de Glasgow de 0 à 2 ans
Ouverture des yeux :<br>4 - spontanément<br>3 - aux stimuli verbaux<br>2 - aux stimuli douloureux<br>1 - aucune réponse	Ouverture des yeux :<br>4 - spontanément<br>3 - aux stimuli verbaux<br>2 - aux stimuli douloureux<br>1 - aucune réponse	Ouverture des yeux :<br>4 - spontanément<br>3 - aux stimuli verbaux<br>2 - aux stimuli douloureux<br>1 - aucune réponse
Réponse verbale :<br>5 - est orienté et parle<br>4 - est désorienté et parle<br>3 - paroles inappropriées<br>2 - sons incompréhensibles<br>1 - aucune réponse	Réponse verbale :<br>5 - mots appropriés, sourit, fixe, suit du regard<br>4 - mots appropriés, pleure, est consolable<br>3 - hurle, est inconsolable<br>2 - gémit aux stimuli douloureux<br>1 - aucune réponse	Réponse verbale :<br>5 - agit normalement<br>4 - pleure<br>3 - hurlements inappropriés<br>2 - gémissements (grunting)<br>1 - aucune réponse
Réponse motrice :<br>6 - répond aux demandes<br>5 - localise la douleur<br>4 - se retire à la douleur<br>3 - flexion à la douleur (décortication)<br>2 - extension à la douleur (décérébration)<br>1 - aucune réponse	Réponse motrice :<br>6 - répond aux demandes<br>5 - localise la douleur<br>4 - se retire à la douleur<br>3 - flexion à la douleur (décortication)<br>2 - extension à la douleur (décérébration)<br>1 - aucune réponse	Réponse motrice :<br>6 - mouvements spontanés intentionnels<br>5 - se retire au toucher<br>4 - se retire à la douleur<br>3 - flexion à la douleur (décortication)<br>2 - extension à la douleur (décérébration)<br>1 - aucune réponse

APPENDIX 4

Fiche d'évolution clinique

Vous avez amené votre enfant aux urgences pédiatriques, suite à un traumatisme crânien. Nous vous rappelons, pour prendre de ses nouvelles.

	oui	non
A-t-il vomi depuis son retour à domicile ? Combien de fois ?		
(Pour les enfants de plus de 4 ans.) Se plaint-il de maux de tête ?		
Avez-vous remarqué un problème pour bouger un bras, une jambe ?		
A-t-il convulsé depuis ?		
A-t-il un changement dans sa mimique ? (paralysie faciale)		
Pour vous, son état est-il revenu à l'état antérieur ?		
Si non, pouvez-vous m'indiquer ce que vous avez remarqué ?	……………………	

Pour les dossiers consultés :

	oui	non
A-t-il vomit ? Combien de fois ?		
(Pour les enfants de plus de 4 ans.) Se plaint-il de maux tête ?		
A-t-il un déficit moteur ?		
A-t-il convulsé ?		
A-t-il une paralysie faciale ?		
Le réflexe photomoteur est-il présent ?		
L'enfant a-t-il été transféré dans un service de neurochirurgie ou de réanimation ?	……………………	
L'enfant a-t-il eu un scanner?		
Et si oui quel est le résultat ?	……………………	

APPENDIX 5
Notice du kit de dosage de la protéine S100B (Roche)

12177293001V5

S100

S100

cobas®

03175243 190 — 100 tests

• Réactifs utilisables sur les analyseurs suivants :

Elecsys 1010	Elecsys 2010	MODULAR ANALYTICS E170	**cobas e** 411	**cobas e** 601
•	•	•	•	•

Français

Remarque
La concentration en protéine S100 d'un échantillon de patient peut varier selon le test pratiqué. Le compte rendu du laboratoire doit donc toujours préciser la méthode de dosage de S100 utilisée. Les taux de S100 d'un patient obtenus à partir de différentes méthodes ne peuvent être comparés, ceci pouvant conduire à des erreurs d'interprétation médicale. En cas de changement de méthode au cours du suivi thérapeutique, les taux de S100 doivent être confirmés pendant une période transitoire en effectuant des dosages en parallèle par les deux méthodes.

Domaine d'utilisation
Test immunologique pour la détermination quantitative *in vitro* de la protéine S100 (S100 A1B et S100 BB) dans le sérum humain.
Le test Elecsys S100 peut être utilisé
- comme aide au suivi de patients atteints de mélanome malin (le test Elecsys S100 n'est pas approprié pour le diagnostic de mélanome malin).
- comme aide pour l'évaluation de souffrances cérébrales potentielles, en association avec les données cliniques et des techniques d'imagerie.

Ce test par électrochimiluminescence « ECLIA » s'utilise sur les analyseurs Elecsys et **cobas e**.

Caractéristiques
La protéine S100 est une petite protéine dimérique d'un poids moléculaire d'env. 10,5 kD. Elle appartient à la famille multigénique des protéines liant le calcium.[1,2]
Les protéines S100A1 (α) et S100B (β) ont été les premières décrites et isolées, à l'origine, par Moore, comme un mélange non fractionné[3] de cerveau bovin, et nommées S100 en raison de leur solubilité dans une solution de 100% de sulfate d'ammonium saturé. Depuis, au moins 21 différentes protéines de la famille S100 ont été identifiées.[4]
Les protéines S100A1 et S100B sont principalement exprimées par des cellules du systéme nerveux central, surtout dans les cellules du cytosol glial, mais également dans les cellules de mélanomes et, dans une certaine mesure, dans d'autres tissus. La protéine fonctionnelle, composées d'hétérodimères et d'homodimères des sous-unités A1 et B, est impliquée dans différentes activités régulatrices intra et extracellulaires.
Chez les patients atteints de mélanome malin, et surtout dans les stades II, III et IV, une augmentation des taux sériques de S100 peut indiquée une progression de la maladie. Des séries de dosages peuvent être utiles pour le suivi des patients et la surveillance de la réponse au traitement.[7,8,9,10,11,12,13]
Par ailleurs, les concentrations de S100 augmentent dans le LCR (liquide céphalo-rachidien) à la suite de certaines lésions cérébrales et sont relarguées dans le sang périphérique.
La S100 peut être détectée chez les patients présentant une lésion cérébrale survenue de diverses manières, tels que les traumatismes crâniens [14,15,16,17,18,19] ou les accidents cardiovasculaires.[20,21,22]

Principe
Méthode « sandwich ». Durée totale du cycle analytique : 18 minutes
- 1ère incubation : une prise d'essai de 20 µL est mise en présence d'un anticorps monoclonal anti-S100 spécifique biotinylé et d'un anticorps monoclonal anti-S100 spécifique marqué au ruthénium[a]. Il se forme un « sandwich ».
- 2e incubation : les microparticules tapissées de streptavidine sont ajoutées dans la cuvette réactionnelle. Le complexe immunologique est fixé à la phase solide par une liaison streptavidine-biotine.
- Le mélange réactionnel est transféré dans la cellule de mesure, les microparticules sont maintenues au niveau de l'électrode par un aimant. L'élimination de la fraction libre est effectuée par le passage de ProCell. Une différence de potentiel appliquée à l'électrode déclenche la production de luminescence qui est mesurée par un photomultiplicateur.
- Les résultats sont obtenus à l'aide d'une courbe de calibration. Celle-ci est générée, pour l'analyseur utilisé, par une calibration en 2 points et une courbe de référence mémorisée dans le code-barres du réactif.

a) $Ru(bpy)_3^{2+}$: Tris(2,2'-bipyridyl)ruthénium(II)

Réactifs - composition et concentrations

M — Microparticules tapissées de streptavidine, 1 flacon contenant 6,5 mL (bouchon transparent) : microparticules tapissées de streptavidine 0,72 mg/mL ; conservateur

R1 — Ac anti-S100~biotine, 1 flacon contenant 9 mL (bouchon gris) : anticorps (monoclonal de souris) anti-S100 biotinylé 1,0 mg/L ; tampon phosphate 50 mmol/L, pH 7,2 ; conservateur

R2 — Ac anti-S100~$Ru(bpy)_3^{2+}$, 1 flacon contenant 9 mL (bouchon noir) : anticorps (monoclonal de souris) anti-S100 marqué au ruthénium 1,0 mg/L ; tampon phosphate 50 mmol/L, pH 7,2 ; conservateur

Précautions d'emploi et mises en garde
Pour diagnostic *in vitro*
Observer les précautions habituelles de manipulation en laboratoire.
L'élimination de tous les déchets doit être effectuée conformément aux dispositions légales.
Fiche de sécurité disponible sur demande pour les professionnels.
Éviter la formation de mousse dans les réactifs et les échantillons de tous types (échantillons de patients, calibrateurs et contrôles).

Préparation des réactifs
Les réactifs contenus dans le coffret sont prêts à l'emploi et ne peuvent être utilisés séparément.
Toutes les informations nécessaires au déroulement du test sont mémorisées sur le code-barres des flacons de réactifs et doivent être saisies.

Conservation et stabilité
Conservation entre 2 et 8°C.
Ranger le coffret Elecsys S100 **en position verticale**, de manière à ce que toutes les microparticules soient rassemblées lors de l'homogénéisation qui précède l'analyse.
Stabilité :

Avant ouverture, entre 2 et 8°C	jusqu'à la date de péremption indiquée
Après ouverture, entre 2 et 8°C	12 semaines
Sur MODULAR ANALYTICS E170 et **cobas e** 601	8 semaines
Sur Elecsys 2010 et **cobas e** 411	8 semaines
Sur Elecsys 1010	4 semaines (conservation alternée au réfrigérateur et dans l'appareil entre 20 et 25°C, flacons ouverts au maximum 20 heures).

Prélèvement et préparation des échantillons
Seul le type d'échantillon suivant a été testé et peut être utilisé :
Sérum recueilli sur tubes standard ou contenant un gel séparateur.
Ne pas utiliser de plasma.
Stabilité : 8 heures entre 15 et 25°C, 2 jours entre 2 et 8°C, 3 mois à -20°C.
Les différents types d'échantillons indiqués ci-dessus ont été testés à l'aide d'une sélection de tubes de prélèvement disponibles dans le commerce au moment du test : les tubes de prélèvement des différents fabricants n'ont pas tous été testés. Les systèmes de prélèvement du sang de divers fabricants peuvent contenir différents matériaux pouvant, dans certains cas, influencer le résultat du test. En cas d'utilisation de tubes primaires (systèmes de prélèvement du sang), suivre les instructions données par le fabricant.
Centrifuger les échantillons contenant un précipité avant l'analyse. Ne pas utiliser d'échantillons inactivés par la chaleur. Les échantillons ou contrôles stabilisés par de l'azide ne doivent pas être utilisés.
S'assurer avant l'analyse que la température des échantillons, des calibrateurs et des contrôles se situe entre 20 et 25°C.

S100

S100

cobas®

En raison des risques d'évaporation, il est recommandé de doser les échantillons, les contrôles et les calibrateurs dans les 2 heures qui suivent leur mise en place sur les analyseurs.

Matériel fourni

Voir paragraphe « Réactifs - composition et concentrations ».

Matériel auxiliaire nécessaire

- Réf. 03289834, S100 CalSet pour 4 x 1 mL
- Réf. 03330648, PreciControl S100 : PreciControl S100 1 pour 2 x 2 mL et PreciControl S100 2 pour 2 x 2 mL
- Equipement habituel de laboratoire
- Analyseur Elecsys 1010/2010, MODULAR ANALYTICS E170 ou **cobas e**

Matériel auxiliaire pour les analyseurs Elecsys 1010/2010 et **cobas e** 411 :

- Réf. 11662988, ProCell, 6 x 380 mL, tampon système
- Réf. 11662970, CleanCell, 6 x 380 mL, solution de lavage pour la cellule de mesure
- Réf. 11930346, Elecsys SysWash, 1 x 500 mL, additif à la solution de lavage
- Réf. 11933159, Adaptateur pour SysClean
- Réf. 11706829, Elecsys 1010 AssayCup, 12 x 32 cuvettes réactionnelles ou Réf. 11706802, Elecsys 2010 AssayCup, 60 x 60 cuvettes réactionnelles
- Réf. 11706799, Elecsys 2010 AssayTip, 30 x 120 embouts de pipette

Matériel auxiliaire pour les analyseurs MODULAR ANALYTICS E170 et **cobas e** 601 :

- Réf. 04880340, ProCell M, 2 x 2 L, solution tampon
- Réf. 04880293, CleanCell M, 2 x 2 L, solution de lavage pour la cellule de mesure
- Réf. 03023141, PC/CC-Cups, 12 godets pour la thermorégulation de ProCell M et CleanCell M
- Réf. 03005712, ProbeWash M, 12 x 70 mL, solution de lavage de l'aiguille en fin de série et entre les changements de réactifs
- Réf. 12102137, AssayTip/AssayCup Combimagazine M, 48 blocs de 84 tubes à essai/embouts de pipettes, sacs pour déchets
- Réf. 03023150, WasteLiner (sacs pour déchets)
- Réf. 03027651, SysClean Adapter M, adaptateur pour SysClean

Pour tous les analyseurs :

- Réf. 11298500, Elecsys SysClean, 5 x 100 mL, solution de lavage du système

Réalisation du test

Pour garantir le bon fonctionnement du test, se conformer aux instructions relatives à l'analyseur utilisé indiquées dans la présente notice. Pour les instructions spécifiques de l'analyseur, se référer au manuel d'utilisation approprié.
L'analyseur effectue automatiquement l'homogénéisation des microparticules.
Les informations spécifiques du test mémorisées dans le code-barres doivent être saisies. Si, exceptionnellement, le code-barres ne peut être lu par l'appareil, saisir manuellement la série des 15 chiffres inscrits sur l'étiquette.
Analyseurs MODULAR ANALYTICS E170, Elecsys 2010 et **cobas e** : amener les réactifs réfrigérés à env. 20°C avant le chargement et les placer dans le plateau réactifs de l'appareil thermostaté à 20°C. Eviter la formation de mousse. L'analyseur **gère** le contrôle de la température, l'ouverture et la fermeture des flacons.
Analyseur Elecsys 1010 : amener les réactifs réfrigérés à env. 20-25°C et les placer dans le plateau réactifs/échantillons de l'analyseur (thermostaté entre 20 et 25°C). Eviter la formation de mousse. **Ouvrir** les flacons avant la mise en route de l'analyseur, puis les **refermer**. Les replacer au réfrigérateur après la série de dosages.

Calibration

Traçabilité : la méthode a été standardisé par pesée par rapport à la protéine S100 β/β.
Le code-barres des réactifs Elecsys S100 contient toutes les informations nécessaires à la calibration du lot. La courbe de référence est adaptée à l'analyseur à l'aide des calibrateurs Elecsys S100 CalSet.
Fréquence des calibrations : effectuer une calibration par lot en utilisant du réactif frais (ayant été enregistré depuis au maximum 24 heures sur l'analyseur). Une nouvelle calibration est recommandée pour :

Analyseurs MODULAR ANALYTICS E170, Elecsys 2010 et **cobas e** :

- après 1 mois (28 jours) pour un même lot de réactif
- après 7 jours pour un même flacon de réactif resté sur l'analyseur

Analyseur Elecsys 1010 :

- à chaque nouveau coffret
- après 7 jours entre 20 et 25°C
- après 3 jours entre 25 et 32°C

Pour tous les analyseurs :

- quand elle s'avère nécessaire : par ex. si les résultats du contrôle de qualité se situent en dehors des limites de confiance.

Contrôle de qualité

Utiliser Elecsys PreciControl S100 1 et 2.
D'autres contrôles appropriés peuvent également être utilisés.
Il est recommandé de doser les sérums de contrôle en simple au moins une fois toutes les 24 heures pendant une routine, pour chaque nouveau coffret et lors d'une calibration. La fréquence des contrôles et les limites de confiance doivent être adaptées aux exigences du laboratoire. Les résultats doivent se situer dans les limites de confiance définies.
Chaque laboratoire devra établir la procédure à suivre si les résultats se situent en dehors de ces limites.

Calcul des résultats

L'analyseur calcule automatiquement la concentration en analyte de chaque échantillon. Les résultats sont exprimés au choix en µg/L, en ng/mL ou en pg/mL.

Limites d'utilisation - interférences

Le test n'est pas influencé par l'ictère (bilirubine < 428 µmol/L ou < 25 mg/dL), l'hémolyse (Hb < 0,621 mmol/L ou < 1,0 g/dL), la lipémie (Intralipid < 1500 mg/dL) et la biotine (< 205 nmol/L ou < 50 ng/mL).
Critère d'acceptabilité : recouvrement ± 10% par rapport à la valeur initiale.
Chez les patients traités par de fortes doses de biotine (> 5 mg/jour), il est recommandé d'effectuer le prélèvement de l'échantillon au moins 8 heures après la dernière administration.
Le résultat n'est pas influencé par le facteur rhumatoïde jusqu'à 1000 UI/mL.
On n'a pas observé d'effet crochet jusqu'à 10 µg de S100/mL.
L'influence de 18 médicaments fréquemment administrés a été recherchée *in vitro* : aucune interférence n'a été observée.
Comme dans tous les tests contenant des anticorps monoclonaux de souris, les échantillons de patients ayant reçu des préparations d'anticorps monoclonaux de souris à des fins thérapeutiques ou diagnostiques peuvent donner des résultats erronés.
Dans de rares cas, des titres très élevés d'anticorps anti-streptavidine ou anti-ruthénium peuvent conduire à des interférences.
Le test contient des additifs permettant de minimiser ces effets.
Pour le diagnostic, les résultats doivent toujours être confrontés aux données de l'anamnèse du patient, au tableau clinique et aux résultats d'autres examens.

Domaine de mesure

0,005-39 µg/L (défini par la limite de détection et le maximum de la courbe de référence). Les taux situés en dessous de la limite de détection sont exprimés de la manière suivante : < 0,005 µg/L et les taux situés au-dessus du domaine de mesure de la manière suivante : > 39 µg/L.

Dilution des échantillons

Les échantillons dont le concentrations en S100 se situent au-dessus du domaine de mesure peuvent être dilués à l'aide de Elecsys S100 Cal1 ou de sérum humain exempt de S100. L'utilisation de Elecsys Diluent Universal n'est pas recommandée. Rapport de dilution recommandé : 1/5 (dilution manuelle). La concentration obtenue avec l'échantillon dilué doit être > 1 µg/L. Après dilution manuelle, le résultat obtenu doit être multiplié par le facteur de dilution.

12177293001V5

S100

S100

Valeurs de référence

- *Adultes apparemment sains et patients atteints de mélanome malin*

Les valeurs suivantes ont été obtenues à partir de dosages effectués avec le test Elecsys S100 sur des échantillons de sujets apparemment sains et de patients atteints de mélanome malin à différents stades de la tumeur et sous suivi thérapeutique :

Population	Sous-groupe	Nbre d'échant. (patients)	Médiane µg/L	95e percentile	Nbre d'échant. au-dessus du seuil (> 0,105 µg/L)[b]
Adultes apparemment sains		206 (206)	0,046	0,105	10 sur 206 (4,9%)
Patients avec mélanome malin (à tous stades et sous suivi thérapeutique)	ASM[c]	821 (408)	0,044	0,109	45 sur 821 (5,5%)
	Métastases ganglionnaires régionales	32 (24)	0,047	0,120	4 sur 32 (12,5%)
	Métastases ganglionnaires lointaines/ cutanées	21 (15)	0,093	0,511	10 sur 21 (47,6%)
	Métastases viscérales/ distantes	70 (48)	0,077	0,759	30 sur 70 (42,9%)

b) Nombre d'échantillons d'adultes apparemment sains > 95e percentile
c) Aucun signe de maladie, absence de tumeur

- *Adultes atteints de souffrance cérébrale potentielle*

Les taux de Elecsys S100 ont été mesurés chez des patients présentant une léger traumatisme crânien (Glasgow Coma Score, GCS 13-15) et au moins un symptome au cours des 3 heures suivant l'accident à l'origine du traumatisme. Une tomographie de la boîte crânienne (TBC) a été effectuée au cours des 6 heures suivant l'accident. En utilisant le 95e percentile de sujets apparemment sains (0,105 µg/L) comme valeur seuil, les résultats obtenus avec le test Elecsys S100 comparés à la scannographie de référence étaient les suivants : VPN (valeur prédictive négative) 99,7% ; VPP (valeur prédictive positive) 11% ; sensibilité 98,8% ; spécificité 32,9% (intervalle de confiance de 95% : VPN 99,1-100% ; VPP 8,8-13,3% ; sensibilité 96,5-100% ; spécificité 30-35,9%)

	TBC positive	TBC négative	Total
Elecsys S100 positifs	83	670	753
Elecsys S100 négatifs	1[d]	329	330
Total	84	999	1083

d) 0,098 µg/L

Chaque laboratoire devra vérifier la validité de ces valeurs et établir au besoin ses propres domaines de référence selon la population examinée.

Performances analytiques

Les performances analytiques indiquées ci-dessous sont représentatives. Les résultats obtenus au laboratoire peuvent différer de ceux-ci.

Précision

La reproductibilité a été déterminée à l'aide de réactifs Elecsys, de pools de sérum humain et de contrôles, selon un protocole modifié (EP5-A) du N.C.C.L.S. (National Committee for Clinical Laboratory Standards). Chaque échantillon a été analysé 6 fois par jour pendant 10 jours (n = 60) ; CV intra-série sur l'analyseur MODULAR ANALYTICS E170, n = 21. Les résultats suivants ont été obtenus :

Analyseurs Elecsys 1010/2010 et **cobas e** 411					
		**Précision intra-série**		**Précision totale**	
Echantillon	Moyenne µg/L	DS µg/L	CV %	DS µg/L	CV %
Sérum humain 1	0,08	0,002	2,1	0,002	2,8
Sérum humain 2	0,24	0,003	1,3	0,005	2,0
Sérum humain 3	2,13	0,042	2,0	0,052	2,4
PreciControl S100_1	0,26	0,005	1,8	0,006	2,3
PreciControl S100_2	3,33	0,046	1,4	0,056	1,7

Analyseurs MODULAR ANALYTICS E170 et **cobas e** 601						
	**Précision intra-série**			**Précision totale**		
Echantillon	Moyenne µg/L	DS µg/L	CV %	Moyenne µg/L	DS µg/L	CV %
Sérum humain 1	0,09	0,001	1,0	0,09	0,003	3,1
Sérum humain 2	0,26	0,005	1,8	0,26	0,006	2,5
Sérum humain 3	2,25	0,015	0,7	2,24	0,064	2,9
PreciControl S100_1	0,27	0,004	1,3	0,28	0,007	2,7
PreciControl S100_2	3,39	0,031	0,9	3,38	0,092	2,7

Sensibilité analytique (limite inférieure de détection)

< 0,005 µg/L

La limite de détection correspond au plus faible taux d'analyte mesurable pouvant être distingué de zéro. Elle est obtenue par le calcul et représente la concentration du standard le plus faible de la courbe de référence + 2 écarts-type (calibrateur de référence, standard 1 + 2DS, précision intra-série, n = 21).

Comparaison de méthodes

Une comparaison du test Elecsys S100 (y) avec les tests Liamat Sangtec100 (x_1) et Liaison Sangtec100 (x_2), effectuée à partir d'échantillons cliniques de patients présentant un mélanome malin, a conduit à l'obtention des corrélations suivantes :

Passing/Bablok[23]

Elecsys/Liamat (x_1)
$y = 0{,}550x_1 + 0{,}025$
$r = 0{,}729$
Nombre d'échantillons analysés : 934
Les concentrations des échantillons étaient situées entre env. 0,00 et 9,87 µg/L.

Elecsys/Liaison (x_2)
$y = 0{,}783x_2 + 0{,}003$
$r = 0{,}857$
Nombre d'échantillons analysés : 379
Les concentrations des échantillons étaient situées entre env. 0,01 et 2,08 µg/L.

Spécificité analytique

Les réactions croisées par les dimères de la protéine S100A1 (αα) sont < 1%.

Sensibilité fonctionnelle

< 0,02 µg/L

La sensibilité fonctionnelle est définie comme étant la concentration en analyte la plus basse donnant un coefficient de variation inter-série de 20%.

Bibliographie

1. Donato R. S100: a multigenic family of calcium-modulated proteins of the EF-hand type with intracellular and extracellular functional roles. Int J Biochem Cell Biol 2001;33:637-668.
2. Zimmer DB, Cornwall EH, Landar A, Song W. The S100 protein family: history, function, and expression. Brain Res Bull 1995;4:417-429.
3. Moore BW. A soluble protein characteristic of the nervous system. Biochem Biophys Res Comm 1965;19:739-744.
4. Donato R. Intracellular and Extracellular Roles of S100 Proteins. Microscopy Research and Technique 2003;60:540-551.
5. Heizmann CW, Fritz G, Schäfer BW. S100 Proteins: Structure, Function and Pathology. Frontiers in Bioscience 2002;7:1356-1368.
6. Zimmer DB, Sadosky PW, Weber DJ. Molecular Mechanisms of S100-Target Protein Interactions. Microscopy Research and Technique 2003;60:552-559.
7. Guo HB, Stofffel-Wagner B, Bierwirth T, Mezger J, Klingmüller D. Clinical significance of serum S100 in metastatic malignant melanoma. Eur J Cancer 1995;31A:924-928.
8. Mohammed MQ, Abraha HD, Sherwood RA, MacRae K, Retsas S. Serum S100β protein as a marker of disease activity in patients with malignant melanoma. Med Oncol 2001;18:109-120.
9. Krähn G, Kaskel P, Sander S, Waizenhöfer PJ, Wortmann S, Leiter U, Peter RU. S100β is a more reliable tumor marker in peripheral blood for

S100

S100

cobas®

patients with newly occurred melanoma metastases compared with MIA, albumin and lactate-dehydrogenase. Cancer Research 2001;21:1311-1316.
10. von Schoultz E, Hansson LO, Djureen E, Hansson J, Kärnell R, Nilsson B, Stigbrand T, Ringborg U. Prognostic value of serum analysis S-100β protein in malignant melanoma. Melanoma Research 1996;6:133-137.
11. Abraha HD, Fuller LC, Vivier AW, Higgins EM, Sherwood RA. Serum S-100 protein: a potentially useful prognostic marker in cutaneous melanoma. Br J Dermatol 1997;137:381-385.
12. Garbe C, Leiter U, Ellwanger U, Blaheta HJ, Meier F, Rassner G, Schittek B. Diagnostic Value and Prognostic Significance of Protein S-100 β, Melanoma-Inhibitory Activity, and Tyrosinase/MART-1 Reverse Transcription-Polymerase Chain Reaction in the Follow-Up of High-Risk Melanoma Patients. Cancer 2003;97(7);1737-1745.
13. Hauschild A, Engel G, Brenner W, Gläser R, Mönig R, Henze E, Christophers E. Predictive value of serum S100B for monitoring patients with metastatic melanoma during chemotherapy and/or immunotherapy. British Journal of Dermatology 1999;140:1065-1071.
14. de Kruijk JR, Leffers P, Menheere PPCA, Meerhoff S, Twijnstra A. S-100B and neuron-specific enolase in serum of mild traumatic brain injury patients – a comparison with healthy controls. Acta Neurol Scand 2001;103:175-179.
15. Herrmann M, Curio N, Jost S, Grubich C, Ebert AD, Fork ML, Synowitz H. Release of biochemical markers of damage to neuronal and glial brain tissue is associated with short and long term neuropsychological outcome after traumatic brain injury. J Neurol Neurosurg Psychiatry 2001;70:95-100.
16. Ingebrigtsen T, Romner B, Marup-Jensen S, Dons M, Lundqvist C, Bellner J, Alling C, Börgesen SE. The clinical value of serum S-100 protein measurements in minor head injury: a Scandinavian multicentre study. Brain Injury 2000;14:1047-1055.
17. Biberthaler P, Mussack T, Wiedemann E, Kanz KG, Koelsch M, Gippner-Steppert C, Jochum M. Evaluation of S-100b as a specific marker for neuronal damage due to minor head trauma. World J Surg 2001;25:93-97.
18. Herrmann M, Jost S, Kutz S, Ebert AD, Kratz T, Wunderlich MT, Synowitz H. Temporal profile of release of neurobiochemical markers of brain damage after traumatic brain injury is associated with intracranial pathology as demonstrated in cranial computerized tomography. J Neurotrauma 2000;17:113-121.
19. Townend WJ, Guy MJ, Martin B, Yates DW. Head injury outcome prediction in the emergency department: a role for S-100B? J Neurol Neurosurg Psychiatry 2002;73:542-546.
20. Abraha HD, Butterworth RJ, Bath PMW, Wassif WS, Garthwaite J, Sherwood RA. Serum S-100 protein, relationship to clinical outcome in acute stroke. Ann Clin Biochem 1997;34:366-370.
21. Fassbender K, Schmidt R, Schreiner A, Fatar M, Mühlhauser F, Daffertshofer M, Hennerici M. Leakage of brain-originated proteins in peripheral blood: temporal profile and diagnostic value in early ischemic stroke. J Neurol Sci 1997;148:101-105.
22. Thornhill S, Teasdale GM, Murray GD, McEwen J, Roy CW, Penny KL. Disability in young people and adults one year after head injury: prospective cohort study. BMJ 2000;320:1631-1635.
23. Bablok W, et al. A General Regression Procedure for Method Transformation. J Clin Chem Clin Biochem 1988;26:783-790.

Pour de plus amples informations, se référer au manuel de l'opérateur de l'analyseur utilisé, aux fiches techniques respectives, au dossier « Product Information » et aux notices d'utilisation de tous les réactifs nécessaires.

Les modifications importantes par rapport à la version précédente sont signalées par une barre verticale dans la marge. Les modifications concernant les données contenues dans le code-barres doivent être entrées manuellement.

©2007 Roche Diagnostics.

CE

Roche Diagnostics GmbH, D-68298 Mannheim

Roche

APPENDIX 6
FORMULAIRE DE RECUEIL D'EVENEMENT INDESIRABLE GRAVE

A FAXER INPERATIVEMENT DANS LES 24 H AU PROMOTEUR AU 04.73.75.47.30

Date de déclaration : I__I__I I__I__I I__I__I__I__I

1. IDENTIFICATION DE LA RECHERCHE BIOMEDICALE

N° IDRCB : **2016-A00195-46**

Code Protocole attribué par le promoteur : **PHRC N 2015 BOUVIER (PROS100B)**

Titre de l'essai clinique : **Etude interventionnelle évaluant l'apport du dosage sanguin de la protéine S100B dans la prise en charge du traumatisme crânien léger de l'enfant**

2. INFORMATIONS SUR LE PATIENT

Identification du patient :

Nom (initiales) : I__I__I__I

Prénom : I__I__I

N° Patient (CRF) : I__I__I__I__I

Date de naissance : I__I__I I__I__I I__I__I__I__I

Age : I__I__I__I

Poids (kg) : I__I__I__I

Taille (cm) : I__I__I__I

Sexe : F ☐ M ☐

Antécédents médicaux et familiaux pertinents :

3. INFORMATIONS SUR LE(S) PRODUIT(S)

Nom commercial	Dosage	N° de lot	Voie d'adm.	Posologie (Dose / rythme)	Indication thérapeutique	Début de traitement (date et heure)	Fin de traitement (date et heure (✱)	Code levé ?
1								Oui ☐<br>Non ☐
2								Oui ☐<br>Non ☐
3								Oui ☐<br>Non ☐

✱ Noter « continue » si le traitement n'est pas arrêté.

Si levée de l'insu, résultat : ____________________________

Disparition de l'événement après arrêt du traitement ? Oui ☐ Non ☐

4. EVALUATION DU LIEN DE CAUSALITE

Selon le promoteur, l'événement semble plutôt lié :

Au(x) traitement(s) à l'essai ☐
Au(x) traitement(s) associés ☐
A une maladie intercurrente ☐
Au(x) procédure(s) de l'essai ☐
Autre, à préciser : ☐
____

Commentaires pertinents :

____

____

Selon l'investigateur, l'événement semble plutôt lié :

Au(x) traitement(s) à l'essai ☐
Au(x) traitement(s) associés ☐
A une maladie intercurrente ☐
Au(x) procédure(s) de l'essai ☐
Autre, à préciser : ☐
____

Commentaires pertinents :

____

____

5. INFORMATIONS SUR LES TRAITEMENTS ASSOCIES MEDICAMENTEUX OU NON (à l'exclusion de ceux utilisés pour traiter l'événement)

Nom commercial ou DCI	Dosage	N° de lot	Voie d'adm.	Posologie (Dose / rythme)	Indication thérapeutique	Début de traitement (date, heure)	Fin de traitement (date, heure)
4							
5							
6							
7							

6. INFORMATIONS SUR L'EVENEMENT INDESIRABLE GRAVE

☐ Décès
☐ Mise en jeu du pronostic vital
☐ Invalidité ou incapacité
☐ Hospitalisation ou prolongation d'hospitalisation
Date de début : I__I__I I__I__I I__I__I
Date de fin : I__I__I I__I__I I__I__I
☐ Anomalie congénitale

Lieu de survenue : ______________________
Date de survenue : I__I__I I__I__I I__I__I
Heure de survenue : I__I__I I__I__I

☐ Autre (préciser) :__________________________

Description de l'événement indésirable - Préciser les symptômes prédominants, la chronologie, éventuellement le diagnostic et les traitements de l'événement (joindre les comptes-rendus anonymisés d'hospitalisation d'examens et/ou résultats de laboratoire) :

____

____

____

____

____

____

Evolution : ☐ Amélioration ☐ Stabilité ☐ Aggravation ☐Survie avec séquelles
☐ Décès (cause : lié à l'événement ☐Oui ☐Non) ☐Evolution inconnue

Description (joindre les comptes-rendus anonymisés d'hospitalisation d'examens et/ou résultats de laboratoire) :

____

____

____

Un ou des produits ont-ils été réintroduits ?

Oui		N° N° N°	Non	

Réapparition de l'événement après réintroduction ?

Oui		N° N° N°	Non	

Si oui, date : I__I__I I__I__I I__I__I heure : I__I__I I__I__I

DIAGNOSTIC DIFFERENTIEL

Autres étiologies envisagées:

____

____

Examens complémentaires réalisés et résultats :

____

____

7. INFORMATIONS SUR LE DECLARANT

Nom et adresse du centre investigateur :

____

Centre n° : _____________ Investigateur : ____

Tél. : __________________ Email : ______________________@______________________

Service : _______________

Nom et qualité du déclarant : ________________________________ Signature :

INFORMATIONS SUR LE PROMOTEUR (*cadre réservé au promoteur, ne pas remplir*)

Nom et adresse du promoteur :

____

____

Date de réception par le promoteur : I__I__I I__I__I I__I__I Type de rapport : ☐ initial

☐ suivi n°_________

Date de déclaration aux autorités : I__I__I I__I__I I__I__I

N° d'identification de l'événement par le promoteur : ______________________

Identification de l'autorisation de recherche : ______________________

Nom et qualité du représentant du promoteur: ____

Tél. : __________________ Email : ____________________@_________________________

Fax : __________________ Signature :

CPP SUD-EST VI
Clermont-Ferrand

CHU de CLERMONT-FD
REÇU LE
20 [illegible] 2019
RECHERCHE CLINIQUE

Clermont Ferrand, le 18 novembre 2019

M. Gérald GOUBY
Direction de la Recherche Clinique
Direction Générale Adjointe
HOPITAL G. MONTPIED

Titre de l'essai :	**Etude interventionnelle évaluant l'apport du dosage sanguin de la protéine S100B dans la prise en charge du traumatisme crânien léger de l'enfant**		
Promoteur	CHU Clermont Ferrand		
Investigateur	Dr. Damien BOUVIER		
Réf. CPP	AU 1257		
Réf. ID-RCB	2016-A00195-46		
Réf. Promoteur	PHRC N 2015 BOUVIER		
Acronyme	PROS100B	Acronyme	PROS100B

Documents examinés :	Numéro et date de version
Courriel de soumission	*11 octobre 2019*
Courrier de l'investigateur justifiant les modifications substantielles souhaitées	*08 octobre 2019*
Courrier ANSM-HPS de demande de modification substantielle	*10 octobre 2019*
Protocole d'étude modifié	*Version n°8 du 08 octobre 2019*
Résumé de l'étude modifié	*Version n°6 du 08 octobre 2019*
Liste des investigateurs actualisée	*Version n°7 du 04 octobre 2019*
Tableau comparatif des versions de documents	- -
CVs des investigateurs	- -
**Modification substantielle**	**n° 6**

Monsieur,

Nous accusons réception en date du 11 octobre 2019, d'une demande d'avis pour modification substantielle (n° 6) concernant une étude ci-dessus référencée.

Le Comité a émis un avis favorable à cet amendement, avis rendu sur l'appréciation du respect des dispositions de l'article L.1121-1 et sur la validité de la recherche selon les dispositions des articles L.1123-7 à L.1123-9 et R.1123-42.

Vous trouverez ci-joint un avis favorable qui a été émis lors de la réunion du 08 novembre 2019.

Veuillez agréer, Monsieur, l'expression de nos salutations distinguées.

Le Président,
Pr. Jean-Etienne BAZIN

CPP SUD-EST VI
Clermont-Ferrand

Clermont Ferrand, le 18 novembre 2019

Titre de l'essai :	**Etude interventionnelle évaluant l'apport du dosage sanguin de la protéine S100B dans la prise en charge du traumatisme crânien léger de l'enfant**		
Promoteur	CHU Clermont Ferrand		
Investigateur	Dr. Damien BOUVIER		
Réf. CPP	AU 1257		
Réf. ID-RCB	2016-A00195-46		
Réf. Promoteur	PHRC N 2015 BOUVIER		
Acronyme	PROS100B	Acronyme	PROS100B

Documents examinés :	Numéro et date de version
Courriel de soumission	*11 octobre 2019*
Courrier de l'investigateur justifiant les modifications substantielles souhaitées	*08 octobre 2019*
Courrier ANSM-HPS de demande de modification substantielle	*10 octobre 2019*
Protocole d'étude modifié	*Version n°8 du 08 octobre 2019*
Résumé de l'étude modifié	*Version n°6 du 08 octobre 2019*
Liste des investigateurs actualisée	*Version n°7 du 04 octobre 2019*
Tableau comparatif des versions de documents	- -
CVs des investigateurs	- -
**Modification substantielle**	**n° 6**

Le Comité a été saisi le **: 11 octobre 2019**

par : **Monsieur Gérald GOUBY**

représentant le promoteur : **CHU de Clermont Ferrand**

d'une demande d'avis pour un projet de recherche ci-dessus référencé.

Le Comité a examiné les informations relatives à cet amendement lors de sa séance du **: 08 novembre 2019**.

Ont participé à la délibération :

**Premier Collège**		**Deuxième Collège**	
Pr. Jean-Etienne BAZIN (T)	Anesthésiste Réanimateur	M. Bertrand NOUAILLES (T)	Philosophe
Dr. Maureen BERNADACH (T)	Médecin Oncologue	Mme Julie SOUSTRE (S)	Philosophe
Pr. Claude DUBRAY (S)	Pharmacologue	M. Pascal DESSENNE (S)	Psychologue clinicien
Dr. Daniel TERRAL (S)	Pédiatre	M. David LUGEZ (T)	Educateur spécialisé
Dr. Sylvain LEVALLOIS (S)	Pédopsychiatre	Mme Céline VERLET (S)	Assistante sociale
M. Fabrice KWIATKOWSKI (S)	Biostatisticien	Mme Rose-Marie BORGES (T)	Maitre de Conf. en droit privé
Dr. Maylis CAULE (T)	Médecin Généraliste	Me Lucie-Hélène PAGNAT(S)	Avocate
Mme Catherine COUDERT (S)	Pharmacien Hospitalier	Pr. Christiane FORESTIER (T)	Représentant l'ADAPEI 63
Mr. Franck HENTZ (T)	Cadre Sup. de Santé	M. Daniel VIGIER (S)	Représentant l'ASDA
Mme Anne KEBOUR (S)	Cadre Sup. de Santé		

Le Comité a adopté la délibération suivante : **AVIS FAVORABLE**

Le Président,
Pr. Jean-Etienne BAZIN

BMJ Open

Assessment of the advantage of the serum S100B protein biomonitoring in the management of paediatric mild traumatic brain injury—PROS100B: protocol of a multicentre unblinded stepped wedge cluster randomised trial

Damien Bouvier,[1] David Balayssac,[2] Julie Durif,[3] Charline Mourgues,[4] Catherine Sarret,[5] Bruno Pereira,[4] Vincent Sapin[1]

AUTHOR PROOF

To cite: Bouvier D, Balayssac D, Durif J, *et al*. Assessment of the advantage of the serum S100B protein biomonitoring in the management of paediatric mild traumatic brain injury—PROS100B: protocol of a multicentre unblinded stepped wedge cluster randomised trial. *BMJ Open* 2019;**0**:e027365. doi:10.1136/bmjopen-2018-027365

► Prepublication history and additional material for this paper are available online. To view these files, please visit the journal online (http://dx.doi.org/10.1136/bmjopen-2018-027365).

Received 19 October 2018
Revised 14 February 2019
Accepted 29 March 2019

Check for updates

© Author(s) (or their employer(s)) 2019. Re-use permitted under CC BY-NC. No commercial re-use. See rights and permissions. Published by BMJ.

For numbered affiliations see end of article.

Correspondence to
Dr Damien Bouvier;
dbouvier@chu-clermontferrand.fr

ABSTRACT

Introduction S100B serum analysis in clinical routine could reduce the number of cranial CT (CCT) scans performed on children with mild traumatic brain injury (mTBI). Sampling should take place within 3 hours of trauma and cut-off levels should be based on paediatric reference ranges. The aim of this study is to evaluate the utility of measuring serum S100B in the management of paediatric mTBI by demonstrating a decrease in the number of CCT scans prescribed in an S100B biomonitoring group compared with a 'conventional management' control group, with the assumption of a 30% relative decrease of the number of CCT scans between the two groups.

Methods and analysis The protocol is a randomised, multicentre, unblinded, prospective, interventional study (nine centres) using a stepped wedge cluster design, comparing two groups (S100B biomonitoring and control). Children in the control group will have CCT scans or be hospitalised according to the current recommendations of the French Society of Paediatrics (SFP). In the S100B biomonitoring group, blood sampling to determine serum S100B protein levels will take place within 3 hours after mTBI and subsequent management will depend on the assay. If S100B is in the normal range according to age, the children will be discharged from the emergency department after 6 hours' observation. If the result is abnormal, CCT scans or hospitalisation will be prescribed in accordance with current SFP recommendations. The primary outcome measure will be the proportion of CCT scans performed (absence/presence of CCT scan for each patient) in the 48 hours following mTBI.

Ethics and dissemination The protocol presented (Version 5, 03 November 2017) has been approved by the ethics committee Comité de Protection des Personnes sud-est 6 (first approval 08 June 2016, IRB: 00008526). Participation in the study is voluntary and anonymous. The study findings will be disseminated in international peer-reviewed journals and presented at relevant conferences.

Trial registration number NCT02819778.

Strengths and limitations of this study

- ► The PROS100B trial is the first large-scale interventional study on the interest of serum S100B protein determination in the management of paediatric mild traumatic brain injury.
- ► A stepped wedge cluster design will be used involving sequential serum assay of S100B use in clusters of participant centres. As more clusters are exposed to the intervention in a stepped wedge cluster design towards the end of the study than in its early stages, there is a risk that the effect of the intervention might be confounded with underlying temporal trends. However, the statistical analysis is intended to encompass the assessment of this temporal effect.
- ► The unblinded design of the study may lead to a contamination bias during the control period during which the investigators may seek to improve their performance, and to an information bias related to physicians assessing outcomes by knowing the time period of the study. Nevertheless, a learning curve analysis will be performed to evaluate if an improvement in terms of primary outcome is observed over time. As proposed by Cook JA *et al* (Clin Trials 2004), this effect will be analysed using Bayesian hierarchical models to adjust the trial results in the case of a learning curve effect.

BACKGROUND

The incidence of traumatic brain injury (TBI) in children is high (691 per 100 000 in emergency departments), and TBI constitutes a leading public health problem.[1 2] Mild TBI (mTBI), defined according to the Glasgow Coma Scale (GCS) from 13 to 15,[3] is one of the most common causes of paediatric hospital admission.[4 5] Children with mTBI account for 5%–8% of visits to French

paediatric emergency departments (60–100 per 100 000 children).[6]

Cranial CT (CCT) is a standard diagnostic tool for adults with TBI. In children, however, several recent large-scale epidemiological studies have described a link between radiation exposure from CCT scans and the risk of future cancer.[7–9] An Australian study of 11 million children found a 24% (relative risk 2.44) increase in cancer risk for the 680 000 children who underwent CT scanning (including 59% CCT scans) and a 35% (relative risk 3.24) increase in the age group 1–4 years.[8]

Alternatively, children can be admitted for inpatient observation with CCT scans performed only on those with clinical deterioration. This approach reduces X-ray exposure but is more costly than using CCT scans for initial diagnosis.[10 11] However, most CCT scans and inpatient observations could be avoided, since 93%– 100% according to the studies of children suffering from mTBI have no intracerebral lesions.[12] In this context, clinical decision rules were developed to help clinicians identify children showing a very low risk of developing intracerebral lesions.[13] Clinical prediction rules were first validated by the Pediatric Emergency Care Applied Research Network (PECARN) algorithm in a prospective cohort.[14] The application of the PECARN algorithm for children with mTBI leads to beneficial management and more cost-effective care.[15] More recently, Scandinavian guidelines for the initial management of mTBI in children were published but must be validated before extensive clinical use.[16] The PECARN strategy suggests an algorithm in which children with mTBI can be divided into three risk categories (very low, intermediate and high risk) according to their risk of developing clinically severe brain injuries, which directly impacts decision-making regarding CCT scanning. The American study[14] strongly advocated the recommendations for mTBI management issued by the French Society of Emergency Medicine[6] and the French Society of Paediatrics (SFP).[17 18] This algorithm decreased by 10% the use of CCT scans for all three risk categories.[13]

The use of biomarkers is a supplementary tool for identifying patients at risk of intracerebral lesions who might need imaging. Serum S100B protein is well established as a sensitive biomarker in TBI. S100B is one of the calcium-binding proteins found in glial cells. It is a small dimeric cytosolic protein (21 kDa) involved in a variety of intracellular and extracellular regulatory activities.[19 20] After cerebral lesions, S100B is immediately released from damaged glial cells into the blood (detection 30 min after trauma) and eliminated by the kidney. It has a short half-life of about 30–100 min.[21–23] The potential of serum S100B in reducing unnecessary CCT scans in adults presenting mTBI has been well established in many observational studies[24–27] and confirmed by two interventional studies.[28 29] The addition of serum S100B to the Scandinavian guidelines for mTBI management decreased the need for CCT scans in adults by one-third, with a significant cost reduction.[29 30] Undén *et al* described these findings in a meta-analysis of adults with mTBI and voiced the need for more studies in children.[31] Further efforts should focus on standardising serum S100B interpretation in this paediatric population, using specific references ranges.[16] Although some studies have reported data from children with mTBI.[32–36] A recent meta-analysis demonstrated the usefulness of serum S100B as a biomarker in the management of paediatric mTBI while emphasising that a large multicentre study is missing for this population.[37]

In this context, the primary objective of our study is to evaluate the performance of serum S100B measurement in the management of paediatric mTBI by demonstrating a decrease in the proportion of CCT scans prescribed in an S100B biomonitoring group compared with a control group ('conventional management'), with the assumption of a 30% relative decrease in the number of CCT scans between the S100B biomonitoring and control groups. The secondary objectives are to demonstrate the utility of serum S100B measurement with regards to the time spent in the paediatric emergency room, hospitalisations, radiation exposure, sedation and sedative use, the detection of complications (intracranial lesions) by CCT scan, which can occur at a rate of 0%–7% in patients with mTBI, the absence of late side effects at 48 hours and 3 weeks after mTBI, and the compliance of emergency physicians with the S100B assay, and lower management costs.

METHODS AND ANALYSIS

Study settings

This multicentre study will be performed in nine French University Hospital Centres (Clermont-Ferrand, Limoges, Lyon, Marseille, Montpellier, Nice, Reims, Saint-Etienne and Nîmes). The sponsor of the study will be the University Hospital Centre of Clermont-Ferrand. The inclusions period runs from November 2016 to the end of March 2020.

Study design

This is a diagnostic prospective, randomised, controlled, unblinded multicentre study using a stepped wedge cluster design, in which paediatric patients (aged ≤16 years) presenting with mTBI in the paediatric emergency room with a GCS score of 15 will benefit from usual care ('conventional management') in the control group, and from S100B result-related clinical management in the S100B biomonitoring group.

A recent systematic review indicated that stepped wedge cluster randomised design is used in particular 'to evaluate interventions during routine implementation, particularly for interventions that have been shown to be effective in more controlled research settings, or where there is lack of evidence of effectiveness but there is a strong belief that they will do more good than harm'.[38] A stepped wedge cluster-randomised controlled design allows delivering the interventions sequentially to all trial clusters over a number of time periods. The order in which

AUTHOR PROOF

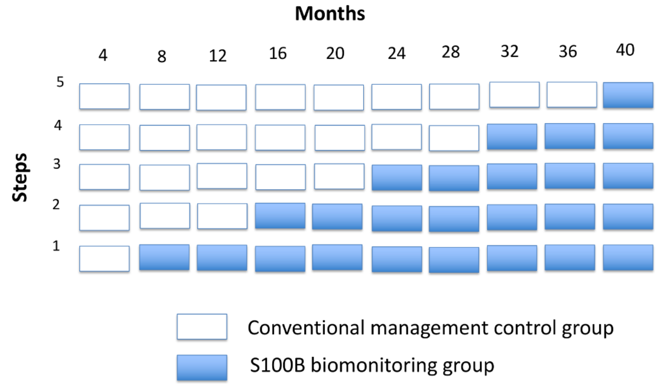

Figure 1 Stepped wedge study design.

the clusters receive interventions is randomised, and by the end of the study all the clusters will have adopted the interventions. Centres will be randomly allocated to a step by the study's statistician using a block randomisation sequence generated in Stata V.13. The step constitution is stratified according to the planned recruitment of each participating centre. Ten intervals of 4 months will be fixed over 40 months. The randomisation will involve five steps for which two centres will be included in the following steps 1, 2, 4 and 5, and one centre in step 3 (figure 1). The children in the control group will receive conventional management treatment in accordance with the SFP recommendations (figure 2). Patients in the S100B biomonitoring group will have a single

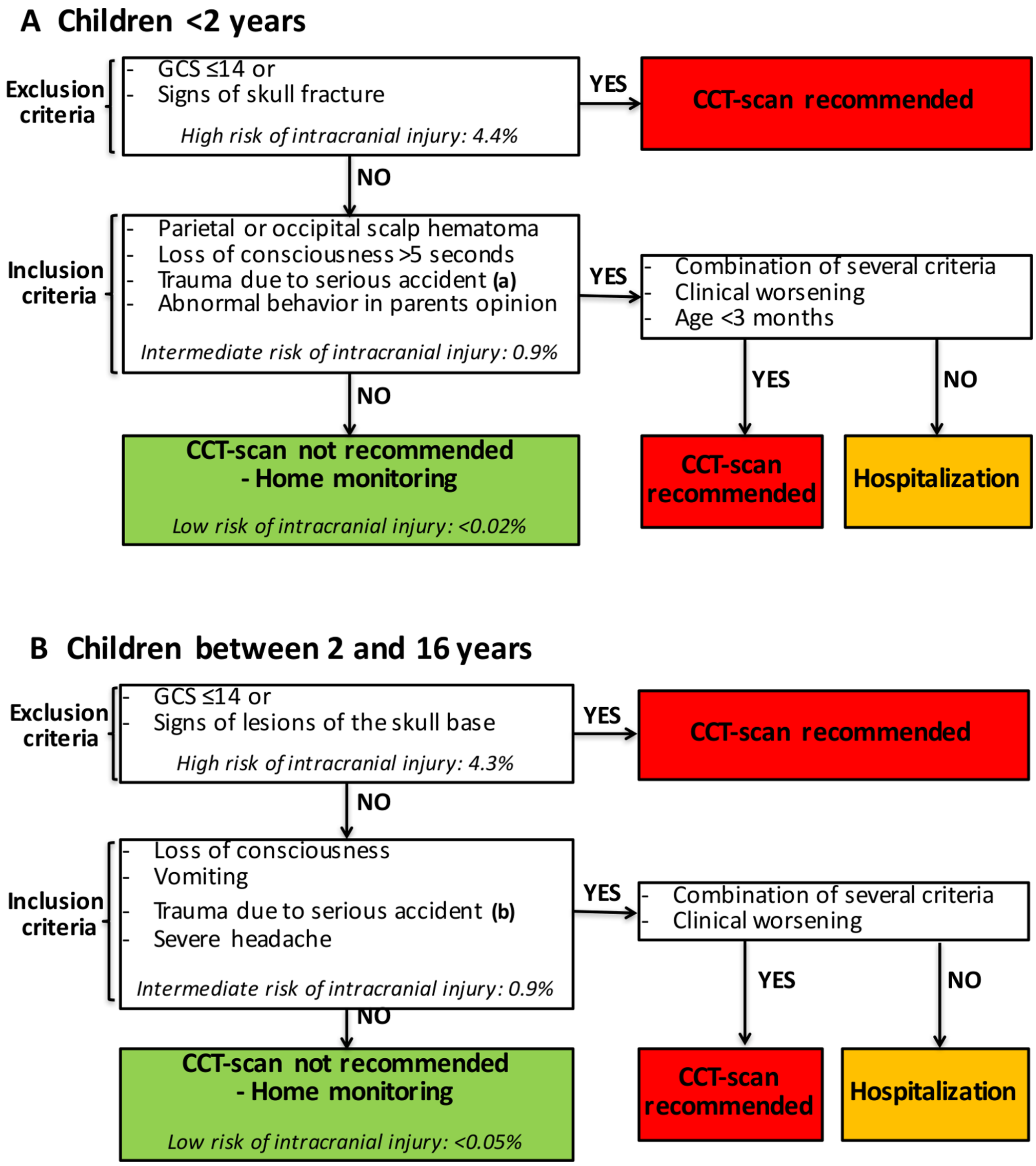

Figure 2 Decision algorithm for CCT scan or hospitalisation indication for children with mild traumatic brain injury management in the 'conventional management' control group. (a) Criteria of serious accident for children under 2 years old: road accident with passenger ejected from vehicle or death of another person or rollover; pedestrian hit by a moving vehicle; cyclist not wearing a helmet; fall from a height greater than 0.9 m. (b) Criteria of serious accident for children over 2 years old: road accident with passenger ejected from vehicle or death of another person or rollover; pedestrian hit by a moving vehicle; cyclist not wearing a helmet; fall from a height over 1.5 m. CCT, Cranial CT; GCS, Glasgow Coma Scale.

AUTHOR PROOF

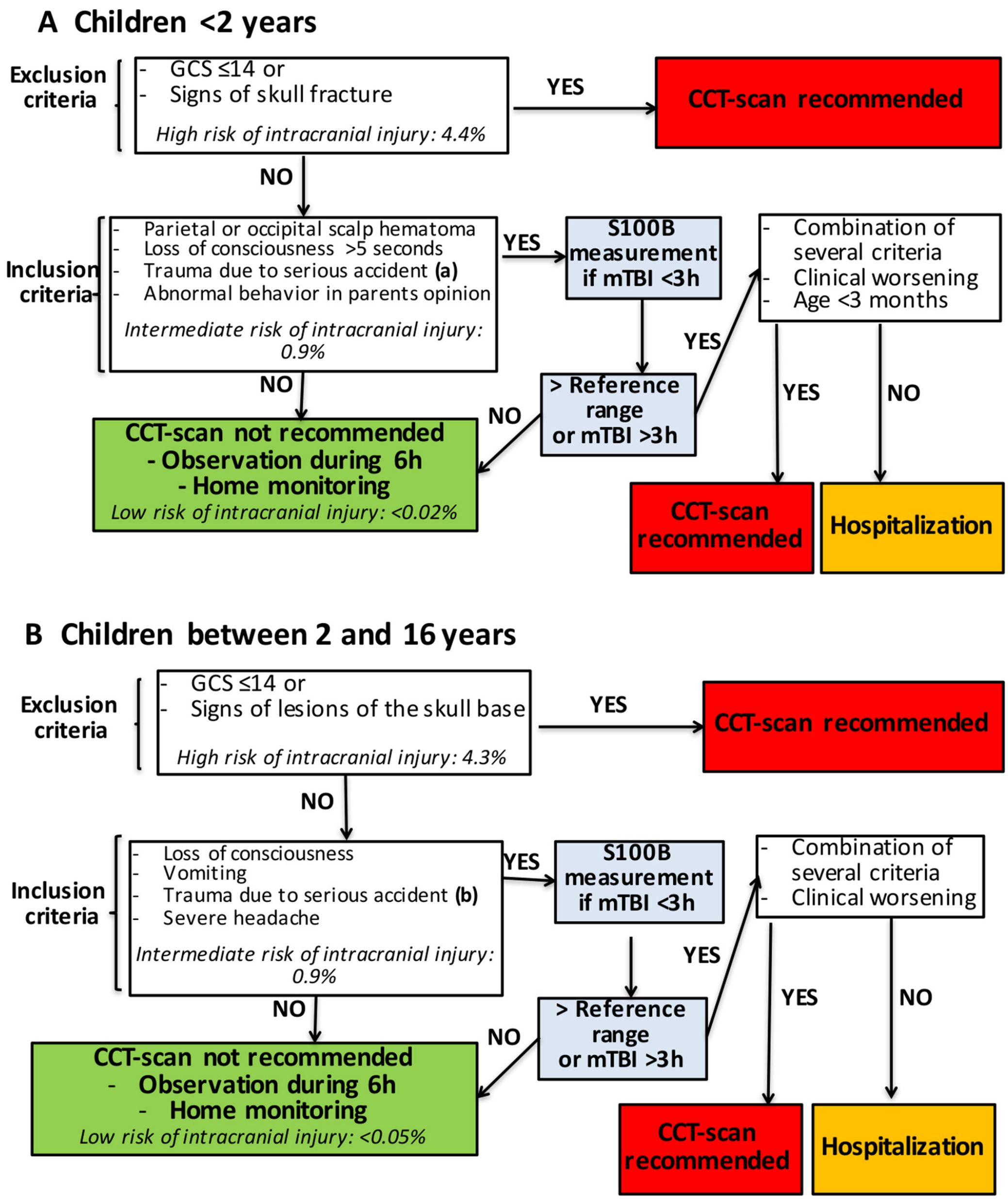

Figure 3 Decision algorithm for CCT scan or hospitalisation indication for children with mTBI management in the S100B biomonitoring group. (a) Criteria of serious accident for children under 2 years old: road accident with passenger ejected from vehicle or death of another person or rollover; pedestrian hit by a moving vehicle; cyclist not wearing a helmet; fall from a height >0.9 m). (b) Criteria of serious accident for children over 2 years old: road accident with passenger ejected from vehicle or death of another person or rollover; pedestrian hit by a moving vehicle; cyclist not wearing a helmet; fall from a height over 1.5 m. CCT, Cranial CT; GCS, Glasgow Coma Scale; mTBI, mild traumatic brain injury.

AUTHOR PROOF

blood sample drawn by a micromethod (1 mL of blood) for S100B determination within 3 hours after trauma, and their subsequent management will depend on the S100B assay results. Children with positive tests will receive conventional management treatment in accordance with the SFP recommendations.[17] In the case of a negative test, the children will be discharged from the emergency department after 6 hours' observation (figure 3).

Physicians will include patients after information and obtain a signed consent form from parents. Research associates will enter the data into the electronic case report form (eCRF). The presence of persistent clinical signs 48 hours and 3 weeks after mTBI will be monitored by the clinical research associates for the two groups by telephone calls. Other hospitalisations will be also sought in the patient' file. Overall, this will be a 41-months study (40 months of inclusion + 1 month for the follow-up of the last patients included).

Eligibility

The study population comprises paediatric patients (aged ≤16 years) admitted to paediatric emergency departments for mTBI with a GCS of 15 requiring hospitalisation and/or CCT scan as per SFP recommendations. The

GCS will be used to evaluate children older than 2 years while the paediatric GCS was assessed in non-verbal children younger than 2 years.[39]

Inclusion criteria

- Age ≤16 years.
- Management within 3 hours after TBI (for blood sampling).
- GCS score of 15 classically requiring hospitalisation and/or CCT scan as per SFP recommendations (figures 2 and 3).[17] These criteria are for children aged under 2 years: parietal or occipital scalp haematoma, loss of consciousness for more than 5 s, trauma due to serious accident (road accident with passenger ejected from vehicle or death of another person or rollover, pedestrian hit by a moving vehicle, cyclist not wearing a helmet, fall from a height superior to 0.9 m), abnormal behaviour in the opinion of parents. For children aged over 2 years, the criteria are: loss of consciousness at time of accident, vomiting, trauma due to serious accident (road accident with passenger ejected from vehicle or death of another person or rollover, pedestrian hit by a moving vehicle, cyclist not wearing a helmet, fall from a height of more than 1.5 m) and severe headache.

Exclusion criteria

- Patient already enrolled in another therapeutic trial with drug administration.
- Down's syndrome, melanoma.
- Refusal of child, parents or legal guardian.
- Trauma occurring more than 3 hours previously.
- GCS score of 13 or 14, or signs of skull fracture or lesions of the skull base (CCT scan recommended) (figures 2 and 3).
- Children with TBI not requiring hospitalisation and/or CCT scan as per SFP recommendations (figures 2 and 3).[17]

Consent

The patients and the parents or legal guardians will be fully and sincerely informed in understandable language of the objectives and constraints of the study, the potential risks, the observation required and safety measures, and of their right to refuse to participate in the study and the possibility to revoke their consent at any time. The investigator must also inform the subjects of the ethics committee's opinion.

All this information appears in an information notice and consent form (online supplementary file 1) given to the patient. The free, informed and written consent of the patient, when given, will be collected by the investigator. These documents are approved by the competent ethics committee and are to be used for the study in question, to the exclusion of any other document.

Two original copies will be co-signed by both the investigator, the patient and the parents or legal guardian. The second copy will be filed in the patient's medical record.

Outcome measures

The primary endpoint is the proportion of CCT scans prescribed (absence/presence of CCT scan for each patient) within 48 hours following TBI, compared between the two groups (S100B biomonitoring group vs 'conventional management' control group).

The secondary endpoints are duration of management defined by the time spent in the paediatric emergency department (time between emergency room admission and discharge), duration of hospitalisation in another hospital department for observation, effective radiation dose (mSv) for each CCT scan, sedation and quantity of sedatives prescribed, presence of intracranial injury on CCT scan, presence of persistent clinical signs at the telephone follow-up interview 48 hours and 3 weeks after the mTBI, and the proportion of positive/negative CCT scans in each group, and cost of management. Data collected are summarised in table 1. Presence of persistent clinical signs is appraised over 48 hours and 3 weeks after mTBI. They were defined by the following clinical symptoms: vomiting, facial paralysis, movement disorders, vertigo, photomotor reflex disorder, seizure, progressive headache or behaviour change. The presence of these signs is sought in the file of the child in case of hospitalisation. Specific hospitalisation in neurosurgery or intensive care unit is also sought. The patients who are not hospitalised will be followed for 48 hours and 3 weeks after consultation, with a standardised telephone interview led by a clinical research associate. The following items will be collected in all participating children: frequency of vomiting since returning home; problems or difficulties related to arms or legs movements observed by parents, convulsions, ocular discomfort, any changes in facial expression, parental opinion of child's return to the previous state of health before the consultation or changes that had been observed. For the children aged over 4 years, additional information will be collected regarding complaints of headaches.

S100B assay

The analytical method used in the study is based on an electrochemiluminescence assay (Roche Diagnostics instruments). Its analytical performance (coefficient of variation of 3.1%) requires only a single determination without risk of analytical error. The test sample volume (20 µL) and dead volume (150 µL) are suitable for the type of blood sampling (micromethod) chosen to be least traumatic to the children. The S100B serum assay will be considered positive according to age: 0–9 months: >0.35 µg/L, 9–24 months: >0.23 µg/L and >24 months: >0.18 µg/L.[40]

Sample size estimation

The proportion of CCT scans ordered during conventional management of mTBI is roughly 20% (between 10% and 30%).[40] Thus, to show a 6% difference between the two groups, ie, a 30% relative reduction[40] in the rate of CCT scans prescribed in the S100B biomonitoring

AUTHOR PROOF

Table 1: Data collected. The information in the table will be recorded on the case report form for each patient

Information	Data collected
Group in the study in which the child is included	► Control or S100B biomonitoring.
Patient characteristics	► Identification code (centre number+inclusion number+patient's initials).<br>► Distance between home and hospital.<br>► Age.<br>► Gender.<br>► Current treatments.<br>► Intercurrent diseases.
Information about the mTBI	► Type of accident.<br>► Date and time of mTBI.<br>► GCS score at arrival at emergency department.<br>► Clinical signs:<br>– presence (+) or absence (–) of signs of impaired consciousness.<br>– presence (+) or absence (–) of signs of skull fracture.<br>– presence (+) or absence (–) of signs of fracture of skull base.<br>– presence (+) or absence (–) of parietal or occipital scalp hematoma.<br>– presence (+) or absence (–) of loss of consciousness for more than 5 s in children under 2 years old.<br>– presence (+) or absence (–) of serious accident.<br>– Presence (+) or absence (–) of immediate loss of consciousness in children over 2 years old.<br>– Presence (+) or absence (–) of vomiting in children over 2 years old.<br>– Presence (+) or absence (–) of abnormal behaviour in the opinion of parents in children under 2 years old.<br>– Presence (+) or absence (–) of severe headache in children over 2 years old.
Information related to serum S100B assay	► Date and time of blood sampling (time between mTBI and blood sampling).<br>► Serum S100B concentration in µg/L.<br>► Concentration higher (+) or lower (–) than age-related reference value.
Information related to CCT scan	► Presence (CCT+) or absence (CCT–) of intracranial trauma.<br>► Nature of lesion in case of CCT+.<br>► Effective radiation dose per patient (mSv).
Information on the stay in the paediatric emergency department	► Date and time of arrival at emergency department (time between mTBI and arrival at emergency department).<br>► Length of stay in emergency room.<br>► Length of stay in other hospital departments.<br>► Presence (+) or absence (–) of hospitalisation (neurosurgery, ICU of other).<br>► Indication (+) or absence of indication (–) for CCT scan.
Information from the follow-up at 48 hours and 3 weeks post-mTBI: positive (+) or negative (–) answer to the standardised questions	► Number of vomiting.<br>► Presence (+) or absence (–) of headache in children over 2 years old.<br>► Presence (+) or absence (–) of convulsion.<br>► Presence (+) or absence (–) of motor deficit.<br>► Presence (+) or absence (–) of facial paralysis.<br>► Presence (+) or absence (–) of abnormal behaviour.<br>► Presence (+) or absence (–) of abnormal photomotor reflex.
Information related to the cost of management	► S100B monitoring.<br>► CCT scan.<br>► Hospitalisation by departments and other later hospitalisation.

CCT, cranial computer tomography; GCS, Glasgow Coma Scale; ICU, intensive care unit; mTBI, mild traumatic brain injury.

group compared with the control group, a sample of 615 subjects per group is required using an individual randomised design, a two-sided type I error at 5% at a statistical power of 80%. Furthermore, in light of the publication of Calcagnile *et al*, it is expected that 30% of physicians treating patients in the S100B biomonitoring group will not be based on their practice on the result of the serum S100B assay.[28] Thus, 800 subjects will be enrolled per group, to conserve a power of 80% with the hypotheses defined previously.[41] The assumption in randomised controlled trials that the outcome of an individual patient is completely unrelated to that of any other patient is violated in cluster randomised trials as patients within any one cluster (centre in our case) are more likely to respond

AUTHOR PROOF

in a similar manner. This similarity is known as the intraclass correlation coefficient (ICC). Because of this lack of independence, larger sample sizes are required. The ICC values usually described in the literature[42] and reported by the University of Aberdeen on a database dedicated to ICC (https://www.abdn.ac.uk/hsru/what-we-do/tools/index.php), range between 0.005 and 0.05. According to these considerations, and in view of the randomisation sequence, time periods, average number of patients per centre, coefficient of variation of cluster size defined as the ratio of the SD of cluster sizes,[43] and lost to follow-up (around 5%), 4000 patients (n=2000 patients by group) will be needed. Every trimester, a newsletter will be sent to each participating centre to present the inclusion rate and encourage inclusions of patients. To estimate sample size, Stata routine stepped wedge developed by Hemming and Girling[44] is used.

An interim analysis is planned after the enrolment of 2000 patients to estimate the statistical power according to the ICC and absolute difference observed. The possible decision to stop the study will be planned considering a type I correction.

Statistical analyses

Analyses will be performed using Stata V.13. All the data will be analysed by intention to treat. The tests will be two-sided, with a type I error set at α=0.05. Baseline characteristics (centres and patients) will be presented as the mean and standard deviation or the median and interquartile range for each randomisation group for continuous data, and as the number of patients and associated percentages for categorical parameters. The characteristics of the patients and clusters will be summarised by randomisation group to allow considering selection biases and lack of balance. Patients will be described and compared between randomised groups at baseline for eligibility, and epidemiological, clinical and treatment characteristics. Protocol deviations and reasons for withdrawal will be described. Other parameters such as the numbers analysed, average cluster size, cluster characteristics and important patient characteristics will be compared in each cluster by period. To compare the proportion of CCT scans prescribed, a generalised linear mixed model (robust Poisson) will be proposed. Randomisation groups, randomisation steps, time and their interactions will be evaluated as fixed effects and centre and time as random effect. Results will be expressed as relative risks and 95% confidence intervals. The estimated intracluster correlation and time effect from the fitted model will be reported.

Multivariable analysis will use the same statistical model with covariates determined according to the univariate results and clinical relevance (age groups defined by age-related S100B cut-off values). Furthermore, particular attention will be paid to between and within physician variability in decision-making regarding the primary endpoint. As it is difficult to know if physician and centre effects will be not nested and confounding, physician within centre will be treated as a random effect in a sensitivity analysis. The comparisons between the two groups for other endpoints will be performed using the same random-effects models, taking into account between and within centre variability: linear (duration of management, duration of hospitalisation, effective radiation dose, quantity of sedatives prescribed; if necessary, logarithmic transformation to obtain the normal distribution will be envisaged) or generalised linear (sedation yes/no, presence of intracranial lesions on CCT scans, presence of persistent clinical signs at the telephone interview). The random-effects models will also be used to study longitudinal repeated data (48 hours and 3 weeks) considering the patient as a random effect in addition to the centre. The statistical nature of missing data will be studied and a sensitivity analysis will be proposed to analyse the impact of missing data on the results and to propose the most appropriate method of imputation.

Finally, according to the design of the study, a sensitivity analysis will be conducted with hospital (cluster) as the main unit of analysis. Usual statistical tests (Student, Mann-Whitney, X^2) will be applied.

Economic analyses

The main objective of the economic analysis will be to measure and compare costs in the two groups.

We choose to perform a cost minimisation analysis because the clinical endpoint is the same for both the intervention and the control group. The protein S100B blood test enables caring for the patient appropriately, detecting TBI earlier and avoiding performing unnecessary CCT scans. The cost analysis will be performed from the standpoint of a University Hospital. Our study will focus on the analyses of care costs and avoided costs in each group. French hospital financing is based on a pricing scale for all current forms of medical care (fee for service). When an innovative medical treatment is developed, the financing system is temporarily based on grants. We hypothesise that the financing system for innovative medical care will finance the protein S100B blood test and that the price will be close to the future price outside the contractual pricing scale of the French health system. Considering the French pricing system, if the number of CCT scans performed for cranial trauma decreases due to introduction of protein S100B blood test in the investigation of mTBI, the hospital might lose income. However, according to the waiting time for this exam and public/private competition, we hypothesise that income will remain stable as each avoided CCT scan will be replaced by another one, irrespective of the medical indication. Costs will be analysed by microcosting during the standard follow-up of 3 weeks planned in the study: the costs of S100B monitoring, costs of the CCT scans including medical costs if children are under sedation during the CCT scan, and costs of hospitalisation by departments and other later hospitalisations. Therefore, the cost analysis focuses on avoided costs thanks to the introduction of protein S100B blood test. We will not consider other

AUTHOR PROOF

consequences such as the impact of decreased delay in care, the decrease of radiation withdrawal effects by reducing inappropriate CCT scans or the lower risk of healthcare associated infections due to shorter lengths of stay.

Study monitoring

The study coordinators will ensure that the study is conducted in accordance with Good Clinical Practice through site monitoring visits. A monitoring protocol has been written and agreed prior to randomisation. A Clinical Research Associate designated by the sponsor will ensure the proper conduct of the study, the collection of data generated in writing, and their documentation, recording and reporting, as per the Standard Operating Procedures in effect at the University Hospital of Clermont-Ferrand and in compliance with Good Clinical Practices and the legislative and regulatory provisions in force.

For each centre, there is an implementation visit protocol, a monitoring visit per year and a closing visit at the end of the inclusions. The implementation visit protocol was made by the principal investigator (Damien Bouvier) and the main Clinical Research Associate (Julie Durif). They presented to the co-investigators the study using a PowerPoint support, brought the investigator file (with all the documents relating to the study) and provided the centre with forms (information notice, consent form, explanatory leaflets for physicians who will recruit participants). The monitoring visits will be conducted by the main Clinical Research Associate who will study patient's research files randomly and provide the centre with forms. All files will be monitored on the eligibility criteria, consent forms and primary endpoint. The closing visits will be conducted by the main Clinical Research Associate to ensure that participant inclusion is closed and archiving for 15 years of all documents related to the study.

Any new information concerning the study which may jeopardise the safety of the research subjects will be subjected to appropriate urgent measures (prompt and timely notification by the Sponsor to the competent authority and the ethics committee). The principal investigator is responsible for reporting all adverse events on the CRF. The only expected adverse effect is a risk of false negative results (expected to be low in view of the sensitivity and negative predictive value of the assay).

An independent monitoring committee will be set up, composed of a biochemist, a paediatrician and a methodologist. This committee will meet for the first time at study initiation and then throughout the duration of the study at its own initiative or at the sponsor's request. The committee will also issue a general opinion on the conduct of the study and can provide an opinion on decision-making related to premature study termination and substantial protocol amendments.

Data management

All the information required by the study protocol (table 1) will be recorded in an eCRF. Data will be collected and transcribed in a clear and legible manner in the eCRF. The eCRF will be developed and made accessible via the internet (SSL 128-bit SSL encryption). Access will be controlled by a personal password and all consultations and changes in the eCRF will be logged. The data will be entered in single input on the eCRF at each study centre. They will be stored on a secured web platform (Clinsight, Ennov Clinical, France) and be validated according to the data management plan established jointly between the principal investigator and the sponsor. Lastly, the data will be frozen/unfrozen according to standard procedures at Clermont-Ferrand University Hospital. All the data will be saved every night, kept for 4 weeks, then backed up on tape every month. Completion of the study is scheduled for December 2020.

Patient and public involvement

Patients and/or public were not involved in the research question and design of the study.

ETHICS AND DISSEMINATION

Important modifications to the protocol, such as modification of eligibility criteria, outcomes, analyses, investigator or centre must be validated by the competent authority and the ethics committee. After these approvals, the new protocol will be transferred to each participating investigator and centre. The data will only be disclosed after the preliminary joint agreement of the investigator and the sponsor. According to the sponsor agreement, the study findings will be disseminated in international peer-reviewed journals and presented at relevant conferences.

Author affiliations
[1]Biochemistry and Molecular Genetic Department, CHU Clermont-Ferrand, Université Clermont Auvergne, CNRS, INSERM, GReD, Clermont-Ferrand, France
[2]DRCI, CHU Clermont-Ferrand, Université Clermont-Auvergne, INSERM U1107, NEURO-DOL, Clermont-Ferrand, France
[3]Biochemistry and Molecular Genetic Department, CHU Clermont-Ferrand, Clermont-Ferrand, France
[4]DRCI, CHU Clermont-Ferrand, Clermont-Ferrand, France
[5]Pediatric Department, CHU Clermont-Ferrand, Clermont-Ferrand, France

Contributors VS and DBo designed the study, designed the trial and obtained ethical approval. JD and CS supervised the trial and data collection. BP provided statistical advice for the study design and analysed the data. DBa drafted the manuscript and all the authors substantially contributed to its revision. CM provided medico-economic advice for the study design.

Funding This study is funded by a grant from the French Ministry of Health (PHRC-15-188).

Competing interests None declared.

Ethics approval The protocol presented(version 5, 03 November 2017) has been approved by the ethics committee CPP (Comité de protection des personnes) sud-est 6 (first approval 08 June 2016, IRB:00008526). The study has been approved by the competent French authority (Agence Nationale de Sécurité du Médicament et des produits de santé, 28 April 2016, ID-RCB no 2016-A00195-46).

Provenance and peer review Not commissioned; externally peer reviewed.

AUTHOR PROOF

Data sharing statement It is anticipated that anonymous data will ultimately be deposited in an appropriate data repository. The final decision regarding data access will be made in conjunction with the publishing journal.

Open access This is an open access article distributed in accordance with the Creative Commons Attribution Non Commercial (CC BY-NC 4.0) license, which permits others to distribute, remix, adapt, build upon this work non-commercially, and license their derivative works on different terms, provided the original work is properly cited, appropriate credit is given, any changes made indicated, and the use is non-commercial. See: http://creativecommons.org/licenses/by-nc/4.0/.

REFERENCES

1. Thurman DJ. The epidemiology of traumatic brain injury in children and youths: A review of research since 1990. *J Child Neurol* 2016;31:20–7.
2. Trefan L, Houston R, Pearson G, *et al*. Epidemiology of children with head injury: a national overview. *Arch Dis Child* 2016;101:527–32.
3. Kristman VL, Borg J, Godbolt AK, *et al*. Methodological issues and research recommendations for prognosis after mild traumatic brain injury: results of the International Collaboration on Mild Traumatic Brain Injury Prognosis. *Arch Phys Med Rehabil* 2014;95:S265–S277.
4. Cassidy JD, Carroll LJ, Peloso PM, *et al*. Incidence, risk factors and prevention of mild traumatic brain injury: results of the WHO Collaborating Centre Task Force on Mild Traumatic Brain Injury. *J Rehabil Med* 2004:28–60.
5. Schutzman SA, Greenes DS. Pediatric minor head trauma. *Ann Emerg Med* 2001;37:65–74.
6. Jehlé E, Honnart D, Grasleguen C, *et al*. Traumatisme crânien léger (score de Glasgow de 13 à 15) : triage, évaluation, examens complémentaires et prise en charge précoce chez le nouveau-né, l'enfant et l'adulte. *Annales françaises de médecine d'urgence* 2012;2:199–214.
7. Pearce MS, Salotti JA, Little MP, *et al*. Radiation exposure from CT scans in childhood and subsequent risk of leukaemia and brain tumours: a retrospective cohort study. *Lancet* 2012;380:499–505.
8. Mathews JD, Forsythe AV, Brady Z, *et al*. Cancer risk in 680,000 people exposed to computed tomography scans in childhood or adolescence: data linkage study of 11 million Australians. *BMJ* 2013;346:f2360.
9. Miglioretti DL, Johnson E, Williams A, *et al*. The use of computed tomography in pediatrics and the associated radiation exposure and estimated cancer risk. *JAMA Pediatr* 2013;167:700–7.
10. Norlund A, Marké LA, af Geijerstam JL, *et al*. Immediate computed tomography or admission for observation after mild head injury: cost comparison in randomised controlled trial. *BMJ* 2006;333:469.
11. Af Geijerstam JL, Britton M, Marké LA. Mild head injury: observation or computed tomography? Economic aspects by literature review and decision analysis. *Emerg Med J* 2004;21:54–8.
12. Homer CJ, Kleinman L. Technical report: minor head injury in children. *Pediatrics* 1999;104:e78.
13. Babl FE, Borland ML, Phillips N, *et al*. Accuracy of PECARN, CATCH, and CHALICE head injury decision rules in children: a prospective cohort study. *Lancet* 2017;389:2393–402.
14. Kuppermann N, Holmes JF, Dayan PS, *et al*. Identification of children at very low risk of clinically-important brain injuries after head trauma: a prospective cohort study. *Lancet* 2009;374:1160–70.
15. Nishijima DK, Yang Z, Urbich M, *et al*. Cost-effectiveness of the PECARN rules in children with minor head trauma. *Ann Emerg Med* 2015;65:72–80.
16. Astrand R, Rosenlund C, Undén J, *et al*. Scandinavian guidelines for initial management of minor and moderate head trauma in children. *BMC Med* 2016;14:33.
17. Lorton F, Levieux K, Vrignaud B, *et al*. Actualisation des recommandations pour la prise en charge du traumatisme crânien léger chez l'enfant. *Archives de Pédiatrie* 2014;21:790–6.
18. Lorton F, Poullaouec C, Legallais E, *et al*. Validation of the PECARN clinical decision rule for children with minor head trauma: a French multicenter prospective study. *Scand J Trauma Resusc Emerg Med* 2016;24:98.
19. Donato R. S100: a multigenic family of calcium-modulated proteins of the EF-hand type with intracellular and extracellular functional roles. *Int J Biochem Cell Biol* 2001;33:637–68.
20. Zimmer DB, Cornwall EH, Landar A, *et al*. The S100 protein family: history, function, and expression. *Brain Res Bull* 1995;37:417–29.
21. Petzold A, Keir G, Lim D, *et al*. Cerebrospinal fluid (CSF) and serum S100B: release and wash-out pattern. *Brain Res Bull* 2003;61:281–5.
22. Jönsson H, Johnsson P, Höglund P, *et al*. Elimination of S100B and renal function after cardiac surgery. *J Cardiothorac Vasc Anesth* 2000;14:698–701.
23. Townend W, Dibble C, Abid K, *et al*. Rapid elimination of protein S-100B from serum after minor head trauma. *J Neurotrauma* 2006;23:149–55.
24. Biberthaler P, Linsenmeier U, Pfeifer KJ, *et al*. Serum S-100B concentration provides additional information fot the indication of computed tomography in patients after minor head injury: a prospective multicenter study. *Shock* 2006;25:446–53.
25. Ingebrigtsen T, Romner B, Marup-Jensen S, *et al*. The clinical value of serum S-100 protein measurements in minor head injury: a Scandinavian multicentre study. *Brain Inj* 2000;14:1047–55.
26. Müller K, Townend W, Biasca N, *et al*. S100B serum level predicts computed tomography findings after minor head injury. *J Trauma* 2007;62:1452–6.
27. Bouvier D, Oddoze C, Ben Haim D, *et al*. [Interest of S100B protein blood level determination for the management of patients with minor head trauma]. *Ann Biol Clin* 2009;67:425–31.
28. Calcagnile O, Undén L, Undén J. Clinical validation of S100B use in management of mild head injury. *BMC Emerg Med* 2012;12:13.
29. Undén L, Calcagnile O, Undén J, *et al*. Validation of the Scandinavian guidelines for initial management of minimal, mild and moderate traumatic brain injury in adults. *BMC Med* 2015;13:292.
30. Calcagnile O, Anell A, Undén J. The addition of S100B to guidelines for management of mild head injury is potentially cost saving. *BMC Neurol* 2016;16:200.
31. Undén J, Romner B. Can low serum levels of S100B predict normal CT findings after minor head injury in adults?: an evidence-based review and meta-analysis. *J Head Trauma Rehabil* 2010;25:228–40.
32. Filippidis AS, Papadopoulos DC, Kapsalaki EZ, *et al*. Role of the S100B serum biomarker in the treatment of children suffering from mild traumatic brain injury. *Neurosurg Focus* 2010;29:E2.
33. Schiavi P, Laccarino C, Servadei F. The value of the calcium binding protein S100 in the management of patients with traumatic brain injury. *Acta Bio-Medica Atenei Parm* 2012;83:5–20.
34. Mondello S, Schmid K, Berger RP, *et al*. The challenge of mild traumatic brain injury: role of biochemical markers in diagnosis of brain damage. *Med Res Rev* 2014;34:503–31.
35. Papa L, Ramia MM, Kelly JM, *et al*. Systematic review of clinical research on biomarkers for pediatric traumatic brain injury. *J Neurotrauma* 2013;30:324–38.
36. Heidari K, Vafaee A, Rastekenari AM, *et al*. S100B protein as a screening tool for computed tomography findings after mild traumatic brain injury: Systematic review and meta-analysis. *Brain Inj* 2015;29:1146–57.
37. Oris C, Pereira B, Durif J, *et al*. The biomarker s100b and mild traumatic brain injury: A meta-analysis. *Pediatrics* 2018;141:141.
38. Mdege ND, Man MS, Taylor Nee Brown CA, *et al*. Systematic review of stepped wedge cluster randomized trials shows that design is particularly used to evaluate interventions during routine implementation. *J Clin Epidemiol* 2011;64:936–48.
39. Borgialli DA, Mahajan P, Hoyle JD, *et al*. Performance of the pediatric glasgow coma scale score in the evaluation of children with blunt head trauma. *Acad Emerg Med* 2016;23:878–84.
40. Bouvier D, Fournier M, Dauphin JB, *et al*. Serum S100B determination in the management of pediatric mild traumatic brain injury. *Clin Chem* 2012;58:1116–22.
41. Machin D. On the evolution of statistical methods as applied to clinical trials. *J Intern Med* 2004;255:521–8.
42. Adams G, Gulliford MC, Ukoumunne OC, *et al*. Patterns of intra-cluster correlation from primary care research to inform study design and analysis. *J Clin Epidemiol* 2004;57:785–94.
43. Eldridge SM, Ashby D, Kerry S. Sample size for cluster randomized trials: effect of coefficient of variation of cluster size and analysis method. *Int J Epidemiol* 2006;35:1292–300.
44. Hemming K, Girling A. A menu-driven facility for power and detectable-difference calculations in stepped-wedge cluster-randomized trials. *Stata J* 2014;14:363–80.

AUTHOR PROOF

Statistical Analysis Plan

Effectiveness of the serum S100B in the management of paediatric minor head injury

Study sponsor
University Hospital of Clermont-Ferrand
58 Rue de Montalembert
63003 Clermont-Ferrand Cedex 1

Principal coordinator
Damien BOUVIER, MD, PhD
Biochemistry and Molecular Genetic Department
University Hospital of Clermont-Ferrand
58 Rue Montalembert, 63000 Clermont-Ferrand, France
Tel: + 33 4 73 75 48 82
Email: dbouvier@chu-clermontferrand.fr

Study methodology
Bruno PEREIRA, PhD
Biostatistics unit, Delegation Recherche Clinique & Innovation (DRCI)
University Hospital of Clermont-Ferrand
58 Rue Montalembert, 63000 Clermont-Ferrand, France
Tel: +33 4 73 75 49 64
Email: bpereira@chu-clermontferrand.fr

Clinical Trials: NCT02819778

Ethics committee CPP: Comité de protection des personnes sud-est 6 (first approval 08/06/2016, IRB: 00008526).

Competent French authority: Agence Nationale de Sécurité du Médicament et des produits de santé, 28/04/2016, ID-RCB n°2016-A00195-46.

CONTENTS

1. TRIAL SUMMARY 3
1.1 PRIMARY OBJECTIVE 3
1.2 SECONDARY OBJECTIVES 3
1.3 TRIAL DESIGN 3
1.4 RANDOMIZATION 3
1.5 ELIGIBILITY CRITERIA 4
1.5.1 Inclusion criteria 4
1.5.2 Exclusion criteria 4
1.6 SAMPLE SIZE 5
1.7 PRIMARY OUTCOME MEASURE 6
1.8 SECONDARY OUTCOME MEASURES 6

2. STATISTICAL ANALYSIS PLAN
2.1 GENERALITY 7
2.2 STUDY POPULATION 7
2.3 PRIMARY ANALYSIS 7
2.4 SECONDARY ANALYSES 7
2.5 ECONOMIC ANALYSES 8

3. MODIFIED STATISTICAL ANALYSIS PLAN
3.1 GENERALITY 10
3.2 STUDY POPULATION 10
3.3 PRIMARY ANALYSIS 11
3.4 SECONDARY ANALYSES 11
3.5 ECONOMIC ANALYSES 11

1. Trial summary

1.1 Primary objective

The primary objective of our study is to evaluate the performance of serum S100B measurement in the management of pediatric mHT by demonstrating a decrease in the proportion of CCT-scans prescribed in a S100B biomonitoring group compared with a control group ("conventional management"), with the assumption of a 30% relative decrease in the number of CCT-scans between the S100B biomonitoring and control groups.

1.2 Secondary objectives

To demonstrate the utility of serum S100B measurement with regards to the time spent in the pediatric emergency room, hospitalisations, radiation exposure, sedation and sedative use, the detection of complications (intracranial lesions) by CCT-scan, which can occur at a rate of 0-7% in patients with mHT, the absence of late side effects at 48 hours and 3 weeks after mHT, and the compliance of emergency physicians with the S100B assay, and lower management costs.

1.3 Trial design

Diagnostic prospective, randomized, controlled, unblinded multicenter study using a stepped-wedge cluster design, in which pediatric patients (aged ≤16 years) presenting with mHT in the pediatric emergency room with a GCS score of 15 will benefit from usual care ("conventional management") in the control group, and from S100B result-related clinical management in the S100B biomonitoring group.

1.4 Randomization

As per the stepped-wedge cluster-randomized trial design, all centres (clusters) delivered conventional management (control group) at the start of the study and crossed over to care according to the S100B biomonitoring (intervention group). At the end of the trial, all centres had crossed over to the intervention group. Randomization of the timing of crossover for each centre was done by an independent statistician using a using a block randomization sequence generated in Stata software (version 13, StataCorp, 160 College Station, US). Centers were stratified according to their planned recruitment. Fifteen 4-month intervals were defined over 60 months. The

randomization consisted of five steps, with two centres enrolled in each of steps 1 to 4 and three centres enrolled in step 5 (Figure 1). Patients and investigators were not masked to treatment.

1.5 Eligibility criteria

Pediatric patients (aged ≤16 years) admitted to pediatric emergency departments for mHT with a GCS of 15 requiring hospitalisation and/or CCT-scan as per SFP recommendations.

The Glasgow coma scale (GCS) will be used to evaluate children older than 2 year-old while the pediatric GCS will be assessed in non-verbal children younger than 2 year-old.

1.5.1 Inclusion criteria

- Age ≤16 years,
- Management within 3 hours after HT (for blood sampling),
- GCS score of 15 classically requiring hospitalisation and/or CCT-scan as per SFP recommendations (Supp Figures 1 and 2). These criteria are for children under 2 years old: parietal or occipital scalp hematoma, loss of consciousness for more than 5 seconds, trauma due to serious accident (road accident with passenger ejected from vehicle or death of another person or rollover, pedestrian hit by a moving vehicle; cyclist not wearing a helmet, fall from a height superior to 0.9 meter), abnormal behavior in the opinion of parents. For children over 2 years old, the criteria are: loss of consciousness at time of accident, vomiting, trauma due to serious accident (road accident with passenger ejected from vehicle or death of another person or rollover; pedestrian hit by a moving vehicle; cyclist not wearing a helmet; fall from a height of more than 1.5 meters) and severe headache.

1.5.2 Exclusion criteria

- Patient already enrolled in another therapeutic trial with drug administration;
- Down’s syndrome, melanoma;
- Refusal of child, parents or legal guardian;
- Trauma occurring more than 3 hours previously;

- GCS score of 13 or 14, or signs of skull fracture or lesions of the skull base (CCT-scan recommended) (Supp Figures 1 and 2);
- Children with HT not requiring hospitalisation and/or CCT-scan as per SFP recommendations (Supp Figures 1 and 2).

1.6 Sample size

The proportion of CCT-scans ordered during conventional management of mHT is roughly 20% (between 10-30%). Thus, to show a 6% difference between the two groups, i.e. a 30% relative reduction in the rate of CCT-scans prescribed in the S100B biomonitoring group compared to the control group, a sample of 615 subjects per group is required using an individual randomized design, a two-sided type I error at 5% at a statistical power of 80%.

Furthermore, in light of the publication of Calcagnile *et al.,* it is expected that 30% of physicians treating patients in the S100B biomonitoring group will not base their practice on the result of the serum S100B assay. Thus, 800 subjects will be enrolled per group, to conserve a power of 80% with the hypotheses defined previously.

The assumption in randomized controlled trials that the outcome of an individual patient is completely unrelated to that of any other patient is violated in cluster randomized trials as patients within any one cluster (center in our case) are more likely to respond in a similar manner. This similarity is known as the intraclass correlation coefficient (ICC). Because of this lack of independence, larger sample sizes are required. The ICC values usually described in the literature and reported by the University of Aberdeen on a database dedicated to ICC, range between 0.005 to 0.05.

According to these considerations, and in view of the randomization sequence, time periods, average number of patients per center, coefficient of variation of cluster size defined as the ratio of the standard deviation of cluster sizes, and lost to follow-up (around 5%), 4000 patients (n=2000 patients by group) will be needed. To estimate sample size, Stata routine *steppedwedge* is used.

An interim analysis is planned after the enrolment of 2,000 patients to estimate the statistical power according to the ICC and absolute difference observed. The possible decision to stop the study will be planned considering a type I correction. More precisely, if the analyses will be conducted for the half of inclusions, statistical adjustment for the interim analysis need to be applied. Applying O'Brien-Fleming

estimates, the inflate type I error will be 0.005. For the primary endpoint, a two-sided P value of less than 0.005 will be considered to indicate statistical significance

1.7 Primary outcome measure

Proportion of CCT-scans prescribed (absence/presence of CCT-scan for each patient) within 48 hours following HT, compared between the two groups (S100B biomonitoring group versus “conventional management” control group).

1.8 Secondary outcome measures

- Duration of management defined by the time spent in the pediatric emergency department (time between emergency room admission and discharge)
- Duration of hospitalisation in another hospital department for observation
- Effective radiation dose (mSv) for each CCT-scan
- Presence of intracranial injury on CCT-scan
- Presence of persistent clinical signs at the telephone follow-up interview 48 hours and 3 weeks after the mHT and cost of management
- Presence of persistent clinical signs is appraised over 48 hours and 3 weeks after mHT. They will be defined by the following clinical symptoms: vomiting, facial paralysis, movement disorders, vertigo, photomotor reflex disorder, seizure, progressive headache, or behavior change. The presence of these signs is sought in the file of the child in case of hospitalisation. Specific hospitalisation in neurosurgery or intensive care unit is also sought. The no hospitalised patients will be followed for 48 hours and 3 weeks after consultation, with a standardized telephone interview led by a clinical research associate.
- The following items will be collected in all participating children: frequency of vomiting since returning home; problems or difficulties related to arms or legs movements observed by parents; convulsions; ocular discomfort; any changes in facial expression; parental opinion of child’s return to the previous state of health before the consultation or changes that had been observed.
- For the children over 4 years old, additional information will be collected regarding complaints of headaches.

2. Statistical Analysis Plan

2.1 Generality

All analyses will be performed with the use of Stata software (version 15, StataCorp, College Station, USA) before the breaking of the randomization code, according to International Conference on Harmonization-Good Clinical Practice guidelines.

The primary analysis will be conducted in the modified intention-to-treat (ITT) population. The criteria for including patients in the modified ITT are provided below. No subgroup analysis will be conducted.

Baseline variables will be reported as numbers and percentages for categorical variables and medians with interquartile ranges (IQRs) for continuous variables.

2.2 Study population

Intention-to treat (ITT) population: All randomised patients
This population will not be analysed.

Modified intention-to-treat population: All randomised patients except patients with one or more major protocol violations.

2.3 Primary analysis

To compare the proportion of CCT-scans prescribed, a random-effect model taking into account center effect will be proposed more especially a generalized linear mixed model (logit according to statistical distribution). Randomization groups, steps of randomization, time periods and their interactions will be evaluated as fixed effects. A robust Poisson mixed model should be used to complete these results in order to present results as relative risks and 95% confidence intervals.

2.4 Secondary analyses

Adjusted analyses will be performed with model defined for the primary analysis with prespecified covariates determined according to clinical relevant: age group distribution and causes of mHT. Significance of the intervention will be assessed based

on p-values and risk ratios with 95% confidence intervals calculated from this regression.

Continuous variables will be presented as mean and standard deviations (as median and quartiles, otherwise). Secondary endpoints will be compared using random-effects models, taking into account between and within center variability: linear for continuous endpoints and generalized linear for categorical endpoints. Randomization groups, steps of randomization, time periods and their interactions will be evaluated as fixed effects. Results will be presented as absolute differences and risk ratios with CIs for categorical data and between-group difference with CIs for continuous data.

A two-sided P value of less than 0.05 will be considered for statistical significance of all analyzes. Because of the potential for type 1 error due to multiple comparisons, findings from analyses of secondary endpoints will be interpreted as exploratory.

2.5 Economic analyses

An economic analysis has been drafted from the Hospital point of view, comparing the costs of hospitalisation stays in the two groups. These costs of stays are directly derived from the cost accounting of institutions and provided by the management financial control departments. It was possible to obtain the actual costs in euros of a day of hospitalisation in general paediatrics unit (conventional hospitalisation unit, 735 euros), short stay hospitalisation unit (498 euros), ICU (2,486.74 euros), surgery unit (1,461.25 euros) and the cost of a transfer to the Emergency Department (181 euros) for the year 2019. The data of charges are available, with a minimum a year of delay, 2021 data are not yet available, and 2020 data have not been considered due to the change in the organization of care and therefore the change in loads induced by the COVID crisis. It was therefore decided to privilege the costs calculated from the expenses of the year 2019 which are more representative of the usual organization of care structures. The cost calculation method used in the study is that of "full costs". It consists in accounting the direct costs (personnel, medicines, medical devices, maintenance, hospitalisation, and equipment depreciation), the induced costs (costs of operative rooms, anesthesia, laboratory, and imaging) and indirect expenses (logistics expenses, general management, and depreciation of financial expenses).

Hospital costs are calculated by multiplying the number of hospital days per patient by the cost per department (conventional hospitalisation unit, short stay hospitalisation unit, ICU, Surgery unit, visit to emergency department) previously mentioned. The cost of an emergency visit alone including "6-hours observation" was recorded at 181 euros. The cost of S100B measurement (32€) is included in the laboratory expenses allowing the average cost to be calculated.To approach the opportunity costs for parents, they were asked if they had to take days or time off to stay with their child for their care (leave taken by a parent).

3. Modified Statistical Analysis Plan

3.1 Generality

All analyses will be performed with the use of Stata software (version 15, StataCorp, College Station, USA) before the breaking of the randomization code, according to International Conference on Harmonization-Good Clinical Practice guidelines.

The primary analysis will be conducted in the modified intention-to-treat (ITT) population. As recommended by the independent committee, we will also perform a planned exploratory per-protocol analysis on the primary outcome. The criteria for including patients in the modified ITT and in the per-protocol population, respectively, are provided below.
No subgroup analysis will be conducted.

Baseline variables will be reported as numbers and percentages for categorical variables and medians with interquartile ranges (IQRs) for continuous variables.

3.2 Study population

Intention-to treat (ITT) population: All randomised patients
This population will not be analysed.

Modified intention-to-treat population: All randomised patients except patients with one or more major protocol violations.

Post hoc analysis
Patients included from only well-functioning centres (i.e. when the S100B was correctly implemented) defined as: centres that did not comply with the decision algorithm for CCT scans and hospitalisations for over 20% of patients, and/or centres that included at least 60% patients less in the S100B biomonitoring group than in the control group. Only primary outcome will be concerned for this post-hoc analysis.

3.3 Primary analysis

To compare the proportion of CCT-scans prescribed, a random-effect model taking into account center effect will be proposed more especially a generalized linear mixed model (logit according to statistical distribution). Randomization groups, steps of randomization, time periods and their interactions will be evaluated as fixed effects. A robust Poisson mixed model should be used to complete these results in order to present results as relative risks and 95% confidence intervals.

3.4 Secondary analyses

Adjusted analyses will be performed with model defined for the primary analysis with prespecified covariates determined according to clinical relevant: age group distribution and causes of mHT. Significance of the intervention will be assessed based on p-values and risk ratios with 95% confidence intervals calculated from this regression.

Continuous variables will be presented as mean and standard deviations (as median and quartiles, otherwise). Secondary endpoints will be compared using random-effects models, taking into account between and within center variability: linear for continuous endpoints and generalized linear for categorical endpoints. Randomization groups, steps of randomization, time periods and their interactions will be evaluated as fixed effects. Results will be presented as absolute differences and risk ratios with CIs for categorical data and between-group difference with CIs for continuous data.

A two-sided P value of less than 0.05 will be considered for statistical significance of all analyzes. Because of the potential for type 1 error due to multiple comparisons, findings from analyses of secondary endpoints will be interpreted as exploratory.

3.5 Economic analyses

An economic analysis has been drafted from the Hospital point of view, comparing the costs of hospitalisation stays in the two groups. These costs of stays are directly derived from the cost accounting of institutions and provided by the management financial control departments. It was possible to obtain the actual costs in euros of a

day of hospitalisation in general paediatrics unit (conventional hospitalisation unit, 735 euros), short stay hospitalisation unit (498 euros), ICU (2,486.74 euros), surgery unit (1,461.25 euros) and the cost of a transfer to the Emergency Department (181 euros) for the year 2019. The data of charges are available, with a minimum a year of delay, 2021 data are not yet available, and 2020 data have not been considered due to the change in the organization of care and therefore the change in loads induced by the COVID crisis. It was therefore decided to privilege the costs calculated from the expenses of the year 2019 which are more representative of the usual organization of care structures. The cost calculation method used in the study is that of "full costs". It consists in accounting the direct costs (personnel, medicines, medical devices, maintenance, hospitalisation, and equipment depreciation), the induced costs (costs of operative rooms, anesthesia, laboratory, and imaging) and indirect expenses (logistics expenses, general management, and depreciation of financial expenses). Hospital costs are calculated by multiplying the number of hospital days per patient by the cost per department (conventional hospitalisation unit, short stay hospitalisation unit, ICU, Surgery unit, visit to emergency department) previously mentioned. The cost of an emergency visit alone including "6-hours observation" was recorded at 181 euros. The cost of S100B measurement (32€) is included in the laboratory expenses allowing the average cost to be calculated.To approach the opportunity costs for parents, they were asked if they had to take days or time off to stay with their child for their care (leave taken by a parent).
