# Supplementary material for: Serum S100B Level in the Management of Pediatric Minor Head Trauma: A Randomized Clinical Trial
Source: JAMA Netw Open. 2024 Mar 19;7(3):e242366. doi: 10.1001/jamanetworkopen.2024.2366 (PMC10951739; doi:10.1001/jamanetworkopen.2024.2366)
Supplement: Supplement 2. — eFigure 1. Stepped-Wedge Study Design eFigure 2. Decision Algorithm for CCT Scan or Hospitalization Indication for Children With Minor Head Trauma Management in the Conventional Treatment Group eFigure 3. Decision Algorithm for CCT Scan or Hospitalization Indication for Children With Minor Head Trauma Management in the S100B Biomonitoring Group eTable 1. Rationale for Exploratory Post Hoc Analysis eTable 2. Characteristics of the Conventional Treatment Group and S100B Biomonitoring Group: Exploratory Post Hoc Analysis on 4 Centers [file jamanetwopen-e242366-s002.pdf]

# Supplementary Online Content

Bouvier D, Cantais A, Laspougeas A, et al. Effectiveness of serum S100B level in the management of pediatric minor head trauma: a randomized clinical trial. *JAMA Netw Open*. 2024;7(3):e242366. doi:10.1001/jamanetworkopen.2024.2366

**eFigure 1.** Stepped-Wedge Study Design

**eFigure 2.** Decision Algorithm for CCT Scan or Hospitalization Indication for Children With Minor Head Trauma Management in the Conventional Management Control Group

**eFigure 3.** Decision Algorithm for CCT Scan or Hospitalization Indication for Children With Minor Head Trauma Management in the S100B Biomonitoring Group

**eTable 1.** Rationale for Exploratory Post Hoc Analysis

**eTable 2.** Characteristics of the Conventional Management Control and S100B Biomonitoring Groups: Exploratory Post Hoc Analysis on 4 Centers

This supplementary material has been provided by the authors to give readers additional information about their work.

© 2024 Bouvier D et al. *JAMA Network Open*.

**eFigure 1.** Stepped-Wedge Study Design

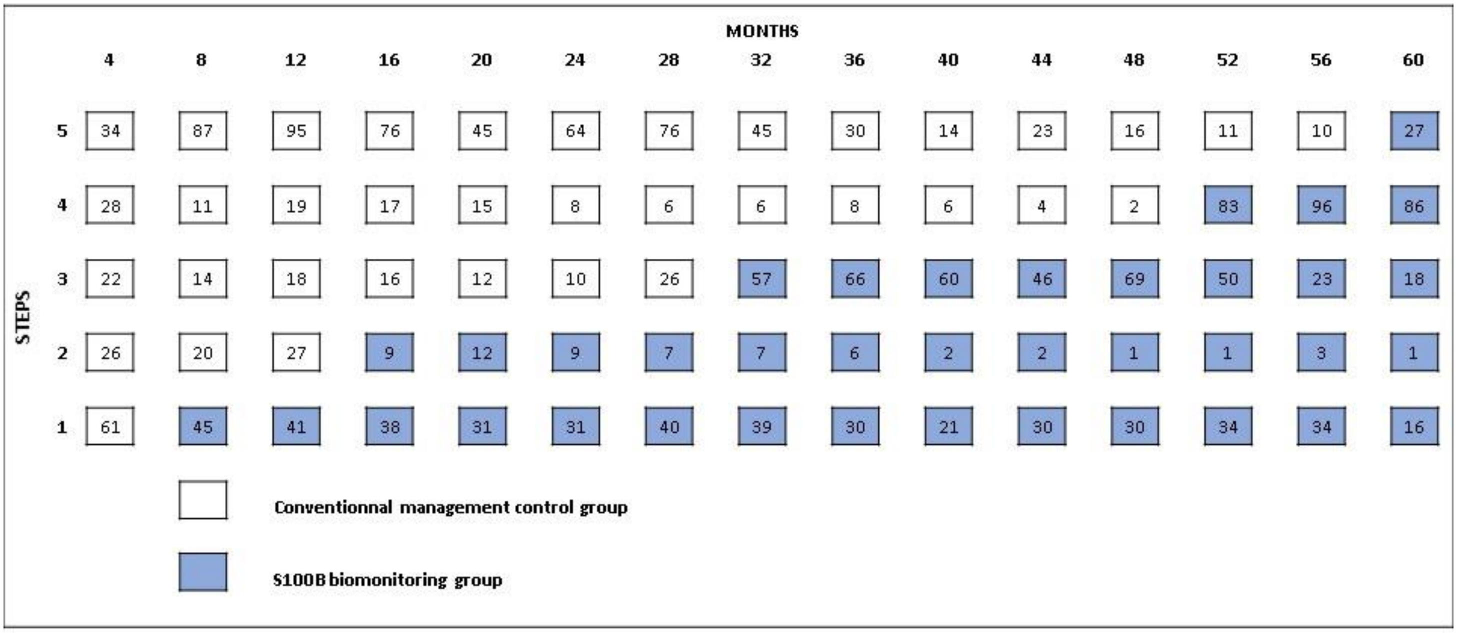


The randomization consisted of five steps, with two centres enrolled in each of steps 1 to 4 and three centres enrolled in step 5. Patients and investigators were not masked to treatment. Number in case: number of patients included in each period of four months

© 2024 Bouvier D et al. *JAMA Network Open*.

**eFigure 2.** Decision Algorithm for CCT Scan or Hospitalization Indication for Children With Minor Head Trauma Management in the Conventional Management Control Group

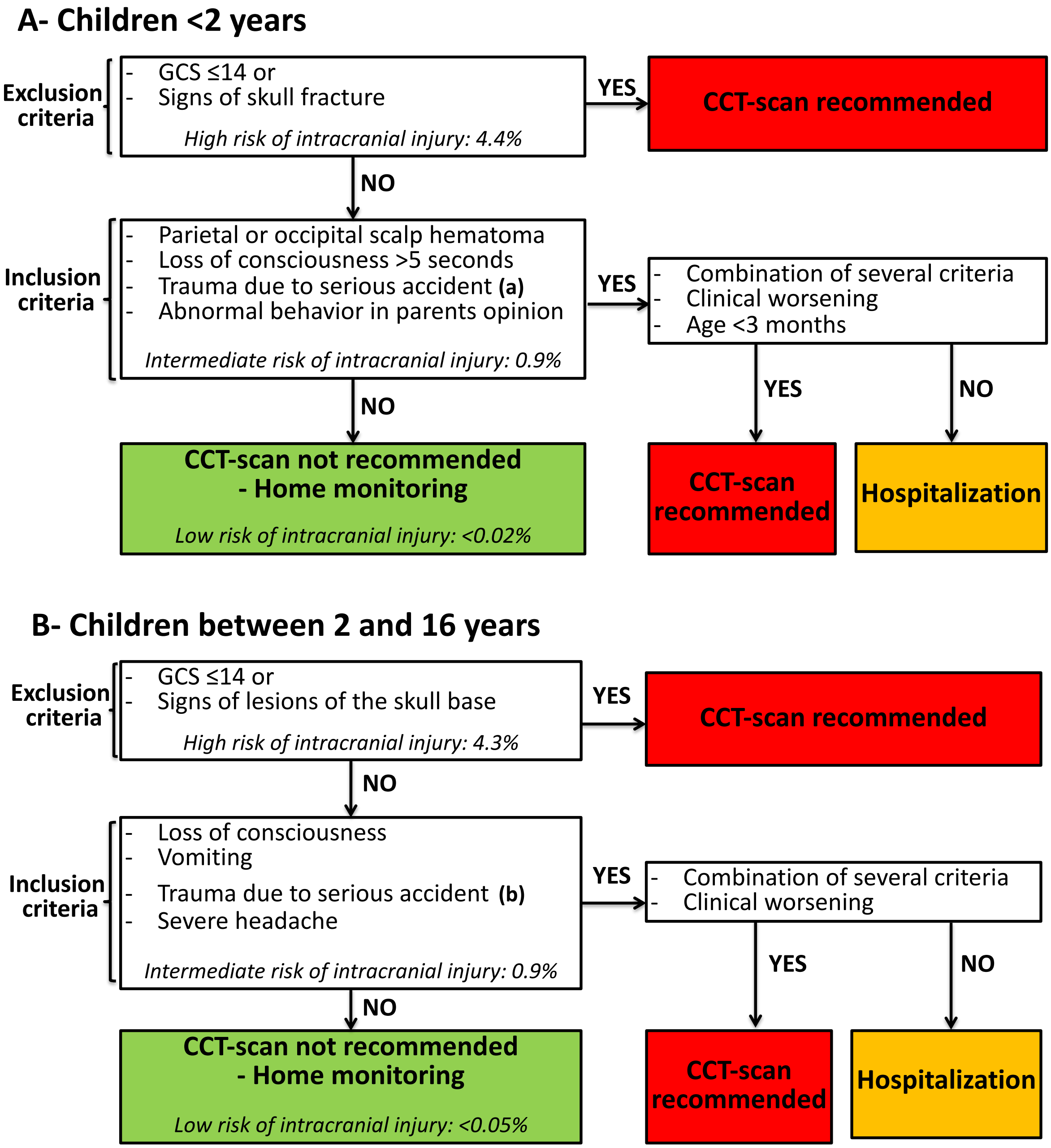


a = criteria of serious accident for children under 2 years old: road accident with passenger ejected from vehicle or death of another person or rollover; pedestrian hit by a moving vehicle; cyclist not wearing a helmet; fall from a height greater than 0.9 meter). b = criteria of serious accident for children over 2 years old: road accident with passenger ejected from vehicle or death of another person or rollover; pedestrian hit by a moving vehicle; cyclist not wearing a helmet; fall from a height over 1.5 meter. The algorithm is adapted from PECARN decision rules.[14,36]
Abbreviations: GCS, Glasgow Coma Scale; CCT, Cranial Computer Tomography

© 2024 Bouvier D et al. *JAMA Network Open*.

**eFigure 3.** Decision Algorithm for CCT Scan or Hospitalization Indication for Children With Minor Head Trauma Management in the S100B Biomonitoring Group

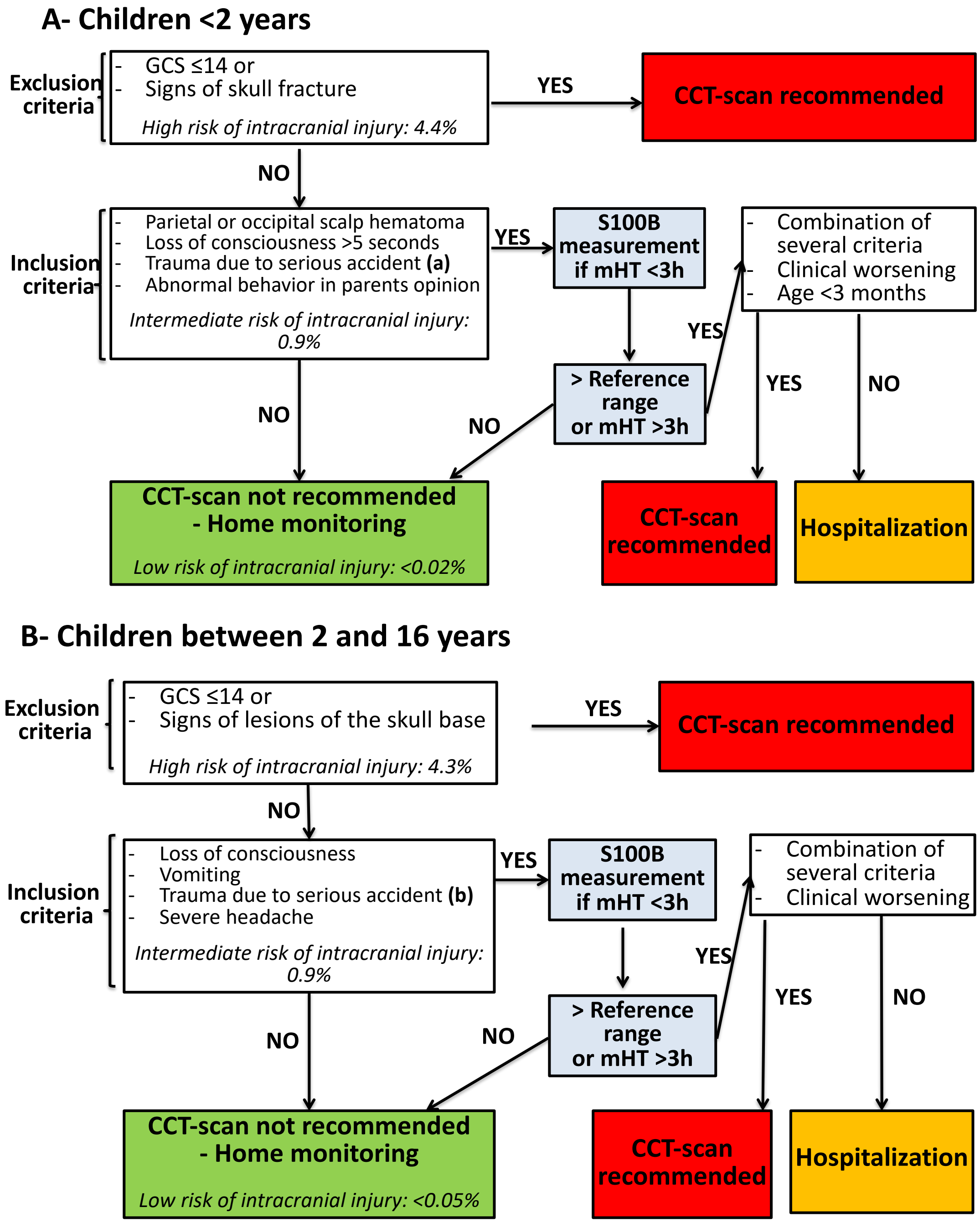


a = criteria of serious accident for children under 2 years old: road accident with passenger ejected from vehicle or death of another person or rollover; pedestrian hit by a moving vehicle; cyclist not wearing a helmet; fall from a height >0.9 meter). b = criteria of serious accident for children over 2 years old: road accident with passenger ejected from vehicle or death of another person or rollover; pedestrian hit by a moving vehicle; cyclist not wearing a helmet; fall from a height over 1.5 meter.

Abbreviations: GCS, Glasgow Coma Scale; CCT, Cranial Computer Tomography; mHT, minor head trauma.

© 2024 Bouvier D et al. *JAMA Network Open*.

**eTable 1.** Rationale for Exploratory Post Hoc Analysis

| Centre number | Centres with more than 60% fewer inclusions in the S100B biomonitoring group than in the conventional management control group. | Non respect of the decision algorithm for CCT-scan or hospitalisation indication > 20% | Centre retained in the post hoc analysis |
|---|---|---|---|
| 1 | | X | |
| 2 | | | X |
| 3 | X | X | |
| 4 | X | X | |
| 5 | | | X |
| 6 | | | X |
| 7 | X | NE | |
| 8 | X | X | |
| 9 | X | X | |
| 10 | X | X | |
| 11 | | | X |

Abbreviation: NE, Non-Evaluated

© 2024 Bouvier D et al. *JAMA Network Open*.

**eTable 2.** Characteristics of the Conventional Management Control and S100B Biomonitoring Groups: Exploratory Post Hoc Analysis on 4 Centers

| | | **Conventional management control group** | **S100B biomonitoring group** | **p** |
|---|---|---|---|---|
| n | | 261 | 757 | / |
| Age in years, median (IQR) | | 1.9 (0.8 - 6.2) | 2.2 (0.9 - 7.1) | 0.045 |
| Age group distribution, n (%) | 0-9 months | 63 (24.1) | 149 (19.7) | 0.31 |
| | 9-24 months | 72 (27.6) | 222 (29.3) | |
| | > 24 months | 126 (48.3) | 386 (51.0) | |
| Sex ratio Male/Female | | 150/111 | 462/295 | 0.50 |
| Skin pigmentation, n (%) | Clear | 210 on 256 (82) | 543 on 692 (78.5) | 0.40 |
| | Intermediate | 35 on 256 (13.7) | 120 on 692 (17.3) | |
| | Dark | 11 on 256 (4.3) | 29 on 692 (4.2) | |
| Weight in kg, median (IQR) | | 11.5 (8.6 - 20) | 12.1 (9.2 - 23) | 0.02 |
| Height in cm, median (IQR) | | 80 (72 - 108) | 80.5 (70 - 116) | 0.63 |
| Distance between home and hospital in km, median (IQR) | | 13 (6 - 25) | 12 (6 - 22) | 0.86 |
| Direct mechanism of injury, n (%) | | 252 (96.6) | 751 (99.2) | 0.005 |
| Causes of mHT, n (%) | Domestic accident | 197 (75.5) | 542 (71.6) | 0.02 |
| | School accident | 39 (14.9) | 82 (10.8) | |
| | Sports related | 14 (5.4) | 77 (10.2) | |
| | Road accident | 11 (4.2) | 54 (7.1) | |
| | Intoxication | 0 (0) | 2 (0.3) | |
| Inclusion criteria for children aged under 2 y, n (%) | Parietal or occipital scalp hematoma | 24 (18.1) | 50 (13.9) | 0.23 |
| | Loss of consciousness >5s | 10 (7.5) | 26 (7.2) | 0.93 |
| | Trauma due to serious accident | 116 (86.6) | 288 (80) | 0.21 |
| | Abnormal behaviour in parents' opinion | 7 (5.3) | 49 (13.6) | 0.03 |
| Inclusion criteria for children aged over 2 y, n (%) | Loss of consciousness | 40 (31.5) | 131 (33.6) | 0.89 |
| | Vomiting | 47 (37.0) | 113 (29.0) | 0.09 |
| | Trauma due to serious accident | 58 (45.7) | 184 (47.2) | 0.56 |
| | Severe headache | 19 (15.0) | 65 (16.7) | 0.58 |

Abbreviations: mHT, minor head trauma; IQR: interquartile range; y: years

© 2024 Bouvier D et al. *JAMA Network Open*.
