# Supplementary material for: Serum S100B Level in the Management of Pediatric Minor Head Trauma: A Randomized Clinical Trial
Source: JAMA Netw Open. 2024 Mar 19;7(3):e242366. doi: 10.1001/jamanetworkopen.2024.2366 (PMC10951739; doi:10.1001/jamanetworkopen.2024.2366)
Supplement: Supplement 3. — Data Sharing Statement [file jamanetwopen-e242366-s003.pdf]

Data Sharing Statement

Bouvier. Serum S100B Level in the Management of Pediatric Minor Head Trauma. *JAMA Netw Open*. Published March 19, 2024. doi:10.1001/jamanetworkopen.2024.2366

Data
Data available: Yes
Data types: Deidentified participant data, Data dictionary
How to access data: The French National Data Safety Authority (CNIL) forbids making data freely available without prior agreement. Thus, the data underlying study findings cannot be made freely available because of ethical and legal restrictions. However, individual participant data underlying the results reported in the manuscript (text, tables, figures, and appendices) can be obtained after deidentification. Data can be obtained upon request from the COMBINE steering committee. Readers may contact: dbouvier@chu-clermontferrand.fr to request the data.
When available: With publication

Supporting Documents
Document types: None

Additional Information
Who can access the data: Any researcher whose proposed use of the data has been approved
Types of analyses: Any purpose. Applications will be assessed for their scientific relevance.
Mechanisms of data availability: To gain access, data requestors will need to sign a data access agreement
